# Impact of drill bit wear on temperature increase in dental implant osteotomy: an in vitro study

**DOI:** 10.1371/journal.pone.0319492

**Published:** 2025-03-19

**Authors:** Marco Sorgato, Anna Bottin, Michele Stocchero, Marco Toia, Enrico Savio

**Affiliations:** 1 Department of Industrial Engineering, University of Padova, Padova, Italy; 2 Department of Neurosciences, School of Dentistry, University of Padova, Padova, Italy; 3 Department of Oral and Maxillofacial Surgery and Oral Medicine, Faculty of Odontology, Malmö University, Malmö, Sweden; University of Vigo, SPAIN

## Abstract

**Objectives:**

Dental implant surgery relies extensively on bone drilling, a critical procedure with intrinsic challenges. Drill bits show significant wear and are frequently utilized beyond the manufacturer’s recommended limits. Such practices can result in adverse effects, including friction and temperature rise in the surrounding bone area during interventions, with an increased risk of necrosis that can compromise the dental implant osseointegration. This study aimed to compare the quality of osteotomy obtained from two different protocols to determine a possible correlation between the drilling temperature and the tool wear and to evaluate their impact on potential health damage.

**Materials and methods:**

Experimental evaluations were conducted using synthetic bone that reproduced human bone characteristics. The drilling phase involved real-time temperature acquisition and scanning electron microscopy analysis of tool wear evolution. After the operation, actual hole size and geometry were characterized using a coordinate measuring machine, and temperatures and torques were measured during the subsequent implantation phase.

**Results:**

The findings revealed a direct correlation between tool wear and the temperature rise during the drilling phase, while a lower correlation was found with the hole profile geometry variation. The implantation phase demonstrated temperature and torque values within acceptable ranges.

**Conclusions:**

This study highlights the importance of adhering to proper tool maintenance and replacement protocols. By following recommended guidelines, practitioners can minimize adverse effects and enhance the success of dental implant procedures.

## Introduction

Bone drilling is an essential and common step in implant dental surgery to securely engage implants into the host bone. This operation aims to achieve an initial mechanical interlock between the bone and the implant, evolving into a process called osseointegration, which is the direct apposition of viable bone cells at the implant surface [1]. Adequate implant surgery is crucial for the implant’s short- and long-term healing and functionality. However, a significant challenge is the inevitable increase in temperature in the area surrounding the implant due to friction generated during the drilling process. This procedure poses a substantial risk by inducing cell death and compromising the implant’s stability in bone [2–4]. In vivo research has shown that irreversible tissue damage occurs when the bone is heated above 53 °C [5], even if this is not an absolute standard. Moreover, the threshold for cell viability has been established at 47 °C for 1 min [6].

Temperature increase during bone drilling is not unique to dental surgeries but is a common issue in all machining operations involving cutting tools. One primary factor contributing to this rise in temperature is the inherently low thermal conductivity of bone tissue [7], making effective temperature management crucial [6,8,9]. The repeated use of drill bits, leading to wear, further exacerbates heat generation at the cutting interface, increasing the risk of thermal damage to surrounding bone tissue [10–13].

The consequences of tool wear extend beyond temperature rise. Worn tools can cause excessive cutting forces and vibrations during drilling. Currently, the decision on when to replace a drill bit is often based on the surgeon’s subjective experience, which can lead to premature replacement or prolonged use of a worn drill bit. Both scenarios are suboptimal, resulting in unnecessary costs or increased risks of mechanical bone deterioration and post-operative complications [14,15].

The thermal dynamics of bone drilling are influenced by various parameters, including drilling speed, feed rate, and applied force [16]. Furthermore, the specific osteotomy required for each implant type affects drill wear, as each drill bit cuts the bone in specific regions, depending on the implant system. These variations can lead to differing levels of wear.

Notably, the existing scientific literature in dental implantology does not currently present conclusive evidence establishing a direct link between tool wear progression and temperature rise during osteotomy. Previous research on the relationship between drill bit wear and temperature rise during osteotomy did not integrate different analyses such as advanced imaging techniques, precise temperature acquisition, and comparative evaluation between distinct drilling protocols. In the current literature, there is a gap in the interplay between wear progression and thermal dynamics.

In particular, referred to distinct sets of drill bits designed for two implant systems. Understanding the impact of tool wear on cutting-edge geometry and its importance in achieving high-quality osteotomies is a complex but essential challenge.

The study emphasizes the clinical relevance of tool wear in reducing thermal risks and improving patient outcomes. By systematically examining these factors, the research contributes to optimizing surgical protocols and tool designs in dental implantology.

## Materials and methods

**Fig 1:**
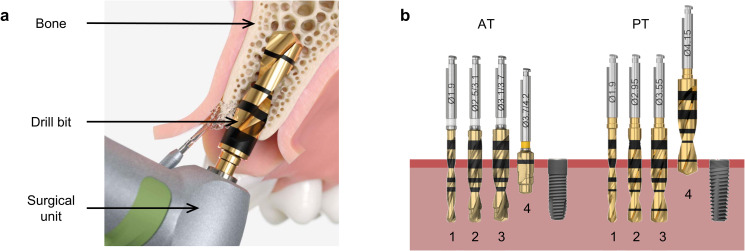
Dental osteotomy and drill bits. **A.** Schematic representation of the drilling process for dental implantation [17] with evidence of the surgical unit, drill bit, and bone. **B.** Sets of drill bits and dental prostheses of the two protocols (AT on the left and PT on the right). Each set includes four drill bits used in sequence to create the final hole geometry. After preparing the osteotomy site, the implant is inserted.

The in vitro analysis aimed to reproduce the clinical operation of dental implantation, as illustrated in Fig 1A. The tests provided a representative model for analyzing the procedure’s effectiveness under controlled conditions, allowing comparisons with clinical observations. The experimental procedure was performed into artificial bone blocks (Sawbones AB, Malmö, Sweden) composed of two layers. The upper layer comprised 1 mm of glass fiber-reinforced epoxy resin to replicate the high mechanical capacity of the cortical layer. The lower 40 mm layer was composed of compact polyurethane foam with a density of 20 pcf (pound per cubic foot), representing the more porous trabecular zone.

Two implant systems were compared: Astra Tech Implant System EV (AT) and PrimeTaper Implant System (PT), both from Dentsply Sirona Implants (Mölndal, Sweden). Two different sets of drilling bits were used for implant insertion. The drilling sequence for each implant type is depicted in Fig 1B, while the specific nomenclature of the instruments is detailed in Table 1. A total of 150 holes were prepared for each protocol. Subsequently, one AT implant (4.2 ×  13 mm) and one PT implant (4.2 ×  13 mm) were inserted in hole number 1 and progressively every ten holes from 10 to 150, for a total of sixteen implants for protocol.

**Table 1:** Drill bit nomenclature. The table lists the nomenclature used for the drilling experiments. Each protocol is characterized by 4 drill bits with progressively increasing diameters.

Protocol	Drill Bit Number	Drill Bit Name
AT	1	Twist Drill EV Ø 1.9, 6-17 mm
	2	Step Drill EV Ø 2.5/3.1, 6-17 mm
	3	Step Drill EV Ø 3.1/3.7, 6-17 mm
	4	Cortical Drill EV Ø 3.7/4.2
PT	1	Drill Ø 1.9 mm long
	2	Drill Ø 2.95 mm long
	3	Drill Ø 3.55 mm long
	4	Drill Ø 4.15 mm long

The same investigations were carried out for AT and PT protocols. This approach facilitated a comparative examination of drill wear and temperature rise between the two distinct protocols, providing valuable insights into the performance of the dental implantation technique under consideration.

### Drilling temperature acquisition

In drilling tests, a custom-made drilling setup with temperature control was employed. A CNC milling machine (Kugler Micromaster 5x, Kugler GmbH, Germany) was used to drive the drill bits. In this experiment, no lubrication or irrigation was used during drilling. The non-use of any coolant was made to minimize potential factors that could affect the results and to avoid its impact on the temperature. This means that only the two types of drilling sets could have affected the temperature changes. As a result, we collected more accurate data, which was more relevant for the statistical analyses [18].

An intermittent drilling strategy was implemented to limit chip clogging, which decreased the cutting forces and, consequently, the temperatures [19]. A study compared intermittent and continuous drilling and found no significant difference in temperature variation during the implant site creation [20]. This confirms that intermittent drilling is a viable alternative to continuous drilling. The operating parameters are described in Table 2. The drilling time of each drilling bit was 10-12 seconds.

**Table 2:** Drilling process parameters. The table presents the parameters used in the CNC drilling process for each drill bit.

Operation	Spindle speed (rpm)	Drilling depth (mm)	Feed rate (mm/min)	Peck feed (mm)	Peck retraction (mm)
AT, step 1-2-3 PT, step 1-2-3	3200	14	240	0.5	1
AT, step 4	3200	8.15	120	0.25	1
PT, step 4	3200	3.15	240	0.25	1

**Fig 2:**
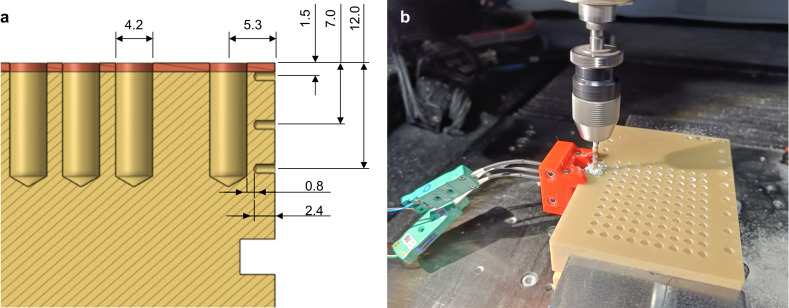
Bone block drilling and temperature acquisition. **A.** Schematic representation of the temperature acquisition set-up, with the distances of the thermocouple from the hole axis. **B.** Actual set-up for temperature acquisition during the drilling operation, with evidence of one drill bit, artificial bone block, and thermocouples.

Each set of bits was used to create 150 holes, surpassing the manufacturers’ set limit of about 25-30 holes. Temperature measurements were conducted at specific intervals during the drilling operations, in the initial drilling and subsequently after every ten holes with type-K thermocouples constructed of 0.25 mm diameter wire. Three thermocouples were placed at different depths from the surface of the cortical layer: 1.5 mm, 7 mm, and 12 mm. A Coordinate Measuring Machine (CMM) (Zeiss O-Inspect, Carl Zeiss AG, Germany) was used to verify the position of the hole’s axis and the other distances specified in Fig 2A. The overall deviation from the nominal value was lower than 0.05 mm, ensuring a reliable setup. Furthermore, by knowing the diameter of each hole, it was possible to determine the points at which to place the thermocouple. This allowed the collection of the temperature values in three critical processing zones: the surface zone near the cortical layer, the intermediate zone, and the zone near the bottom of the hole. To avoid interference with drilling operations, the thermocouples were placed radially 0.8 mm from the side of the largest hole in the protocols, equivalent to 2.9 mm from the hole’s axis. The thermocouples were embedded in a rigid sheath and fixed using a holder produced by additive manufacturing (Fig 2B).

**Fig 3:**
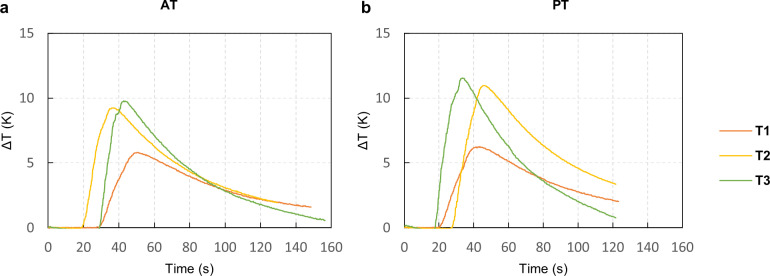
Temperature acquisition for the three different thermocouples (T1 at a depth of 1.5 mm, T2 at 7 mm, and T3 at 12 mm) during the drilling phase. **The temperature is represented as a temperature differential from the base temperature. A.** Temperature difference evolution in AT protocol during the drilling operation. **B.** Temperature difference evolution in PT protocol during the drilling operation.

The temperature profiles for both protocols are shown in Fig 3. The figure represents the differential temperatures recorded by the three thermocouples (T1 at a depth of 1.5 mm, T2 at 7 mm, and T3 at 12 mm). During the experimental campaign, the drillings were carried out 5 minutes apart, one from the other, to allow the artificial bone block to reach room temperature before the subsequent acquisition. The thermocouples were left on to verify that the ambient temperature was reached. The results will always be expressed as temperature differences from the base temperature of each thermocouple as detected before the operation. The base temperature was defined as the average of the values before the start of the operation, identified by exceeding the base temperature by 0.2 K. Moreover, the profiles of the different operations were normalized and time-synchronized, ensuring a common starting point for all various drillings to provide a more precise visualization of the temperature profiles. The normalized profiles calculated the maximum temperatures during each operation, and the graphs presented in the results section were produced.

### Tool wear assessment

**Fig 4:**
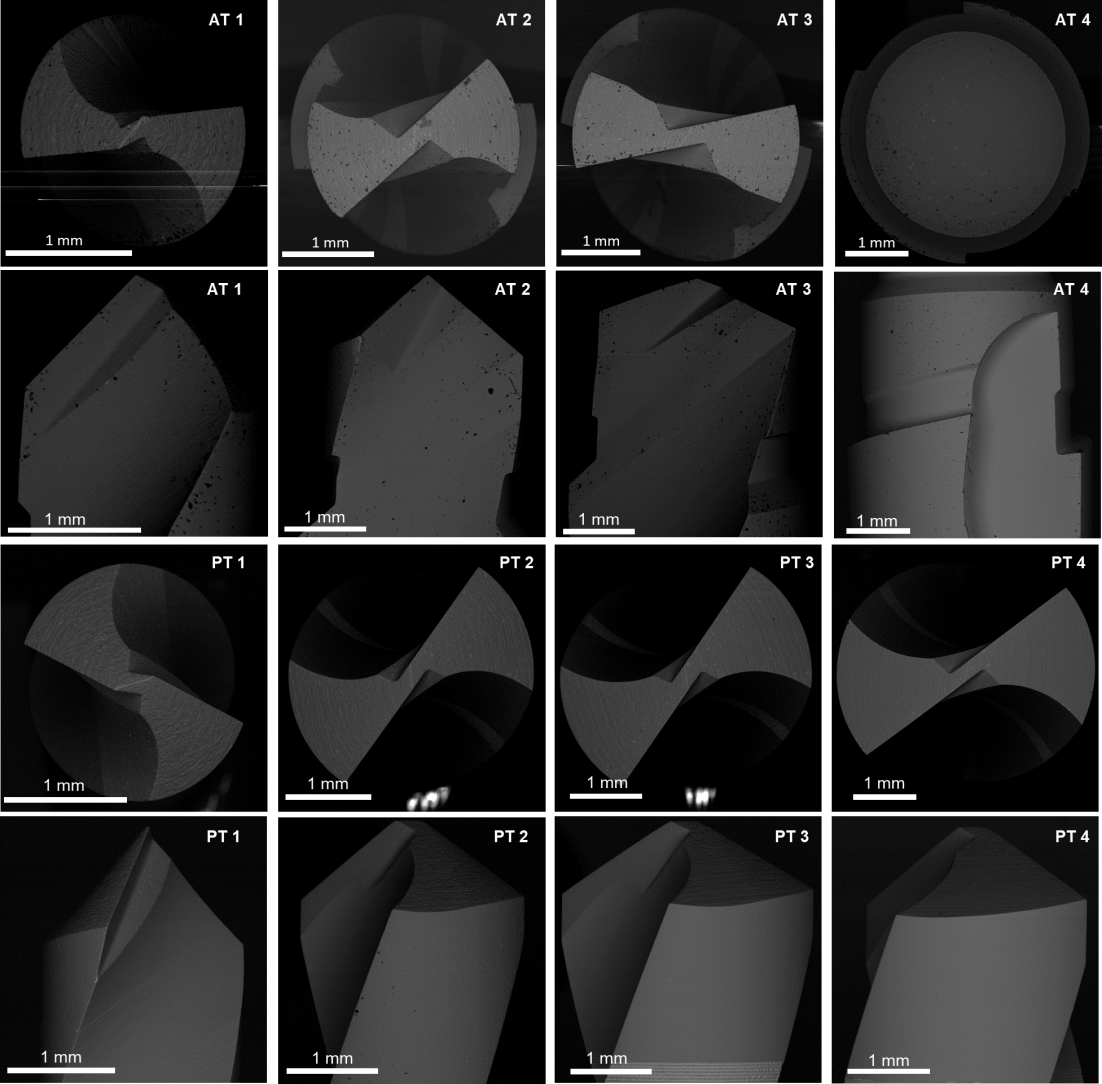
SEM images of the drill bits, taken before drilling operations, perpendicular and parallel to their axis.

A standard method for assessing the drills’ wear in surgical operations needs to be improved. In this study, SEM analysis was performed [21]. A scanning electron microscope (FEI Quanta 450, FEI Company, United States), operating at an accelerating voltage of 25 kV, was used to assess bits wear after the first drilling operation and every ten holes thereafter. The drill bits were cleaned in an ultrasonic bath with acetone for 10 minutes, then rinsed in deionized water and dried with compressed air to prepare for SEM examination. The tips of both protocols were steel coated with a thin layer of TiN, allowing the identification of the worn areas where the coating was no longer present by taking advantage of the backscattered electron detector (BSED). SEM images of each drill can be seen in Fig 4.

After a preliminary drill bit wear assessment, the main wear zone was located on the cutting edge and the main flank surface, so images of the tools were acquired in two orientations: parallel and perpendicular to the drill’s axis. To ensure the repeatability of the drilling experiments, a custom-made mounting device, consisting of an aluminum block with a PLA part in additive manufacturing, was made.

The images were aligned manually by adjusting their rotation and perspective. A preliminary alignment was implemented to facilitate this process by identifying three constant points shared between the images with the same orientation. A custom Python program employing the OpenCV library calculated a transformation matrix. In this way, the pictures were superimposable, simplifying subsequent measurements.

**Fig 5:**
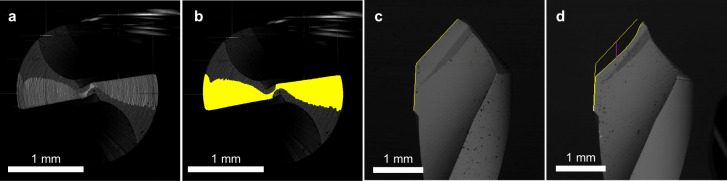
Example of drill bit wear assessment, after 150 holes, for the AT protocol. **A.** SEM image of the top view of the worn tool. **B.** Identification of the worn area in yellow. **C.** SEM image of the front view of the worn tool. **D.** Identification of the axial distance of the cutting-edge retreat from the fresh drill in yellow.

The wear measurement procedure was adapted from a previously described method [22], with the modifications needed from the different geometry of the drill bits. Two different methods for the flank wear assessment were employed for the two different image orientations acquired. In the top view, perpendicular to the drill’s axis, a worn area was measured (Fig 5A), while in the front view (parallel to the drill’s axis), the axial retreat of the cutting edge relative to the unworn tool was quantified to evaluate the effective reduction in drilling depth (Fig 5B). After verifying the scale of the SEM image by measuring a calibrated block of dimension similar to the drill bit, the worn area was initially measured in pixels, and then, using the image scale, it was evaluated in mm^2^. Similarly, the vertical distance between the fresh and worn cutting edges was measured in pixels and converted to mm. The described procedure was performed for each drill bit, and it was replicated three times.

Pearson’s correlation test verified the correlation between tool wear and temperature increase during the drilling operation. Before comparing the data, we first eliminated any outliers and checked if the variable was normally distributed. We visually inspected the data’s normality using a Quantile-Quantile (Q-Q) plot. Then, to calculate the Pearson’s correlation coefficient, the following formula was used (Eq 1):


$$r=\frac{n\sum_{}^{}\left(T_{i}W_{i}\right)-\sum_{}^{}T_{i}\sum_{}^{}W_{i}}{\sqrt{\left[n\sum_{}^{}T_{i}^{2}-{\left(\sum_{}^{}T_{i}\right)}^{2}\right]\left[n\sum_{}^{}W_{i}^{2}-{\left(\sum_{}^{}W_{i}\right)}^{2}\right]}}$$ (1)

where n is the number of data, ∆ T is the temperature increase acquired during the drilling phase, and *W* is the drill bit wear. Results were considered statistically significant when the p-value was less than 0.05.

### Drilled hole characterization

After the four-step protocols, a thorough examination of hole profiles was conducted to assess the potential impact of drill bit wear on the prosthesis implantation phase. This evaluation was performed after the first drilling and subsequently after every set of ten holes. The holes’ geometrical characterization was made through the Coordinate Measuring Machine, using a contact scanning probing system with a 1.5 mm tip diameter and 0.15 N measuring force. The measurement plan was repeated three times, observing micrometric differences among results, and the mean value was calculated.

For each hole, four profiles were measured along orthogonal directions in the inner cylindrical profile of the hole, adopting a continuous scanning strategy with a nominal distance between measured points of 0.05 mm. Subsequently, a Python program was developed to obtain a single-mediated profile from the four profiles for each hole. The Nelder-Mead algorithm from the Scipy library was utilized to make the data fusion [23]. In particular, the optimization algorithm proceeded to explore different values of profile translation and rotation to minimize the global standard deviation of the individual hole until convergence was achieved, ensuring a robust analysis of the geometric characteristics of the holes across the experimental conditions.

**Fig 6:**
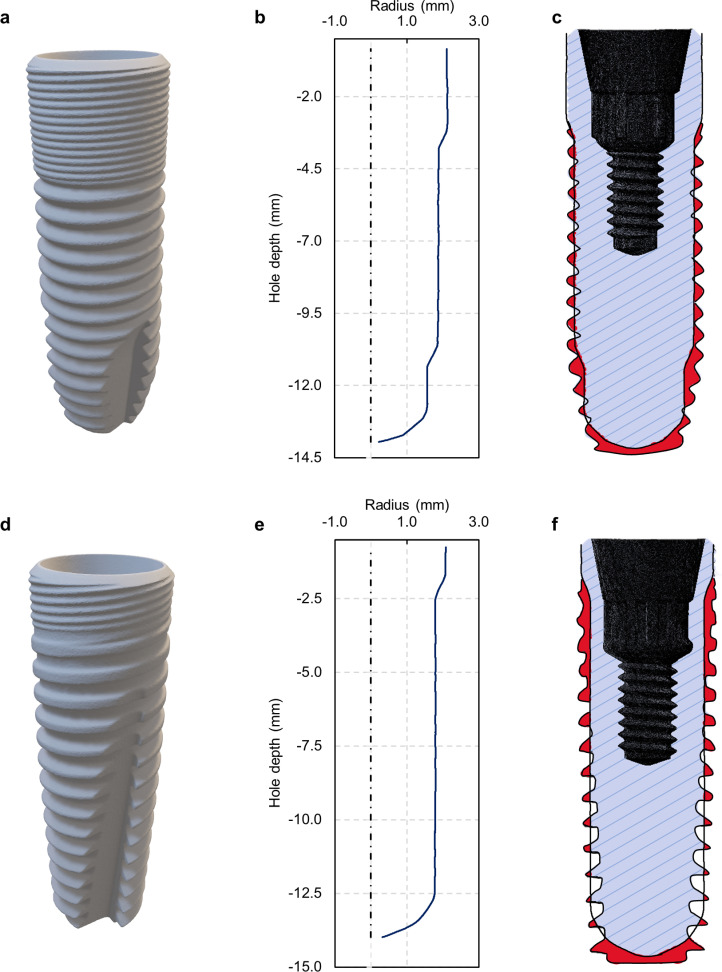
Volume intersection evaluation. **A.** CT scan of an AT implant. **B.** Average profile of the 10^th^ hole of the AT protocol. **C.** Section of the implant for calculating the volume intersection between the implant and the average 10^th^ hole profile for AT protocol. The areas highlighted in red were used to calculate the intersection volume between the drilled hole and the dental implant. **D.** CT scan of a PT implant. **E.** Average profile of the 10^th^ hole of the PT protocol. **F.** Section of the implant for calculating the volume intersection between the implant and the average 10^th^ hole profile for PT protocol. The areas highlighted in red were used to calculate the intersection volume between the drilled hole and the dental implant.

Further investigations were conducted to correlate the change in implant volume intersection, defined as the area of bone affected by the implant during screwing, with the increasing number of drillings (Fig 6). The geometry of the implant was obtained from a Computed Tomography scan (Fig 6A for AT implant and Fig 6D for PT implant), and the hole geometry corresponds to the average profile of hole 10 (Fig 6B for AT implant and Fig 6E for PT implant). The two values were then compared to obtain the final volume intersection (Fig 6C for AT implant and Fig 6F for PT implant).

### Implant insertion

For the implant insertion phase, two dedicated drivers from each protocol were employed: Implant Driver EV 4.2 short (AT) and Implant Driver EV (M) short (PT protocol). An operator made the insertion of the implants by hand to replicate the clinical condition. These drivers were mounted on a surgical unit (W&H Elcomed) equipped with the WI-75 E/KM handpiece (W&H Dentalwerk Bürmoos GmbH, Austria). The implants occupied the first hole and the holes number 10, 20, …, 140, 150 consistently with the temperature acquisitions made. The implant phase involved the implant screwing into the prepared hole with a slight pressure. The system parameters included a maximum torque of 450 Nmm and a rotational speed of 50 rpm. Like the drilling phase, irrigation was not used during implantation.

Throughout the implantation process, insertion torque and temperature measurements were conducted. As for the drilling operation, the temperature was registered in three different sections. Temperature results are computed as a temperature difference between the base temperature of individual thermocouples recorded before each operation. Torque measurements were carried out through the surgical unit, equipped with a documentation function, which acquired the torque exerted when it reached 10 Nmm and concluded 10 seconds after the motor was turned off.

The setup utilized in this study provided highly reproducible experimental conditions for an accurate investigation of the drilling and insertion phases of dental implantation. Three repetitions were performed for holes 1, 10, 20, 30, 40, 50, 100, and 150 for temperature acquisition, drill wear assessment, hole quality evaluation, and implantation temperature and force. These measurements showed very low standard deviations, indicating consistency among the data. The mean value will be presented in the following sections, and the maximum standard deviations of the measurements will be included in the figure legends.

## Results

### Tool wear assessment

This section delves into assessing tool wear in osteotomy drills, a key factor in this study on temperature increase and tissue damage during dental implant surgeries. As introduced earlier, the wear assessment is crucial to understanding its impact on temperature increase and subsequent tissue damage. Using SEM and image analysis, it is possible to compare a fresh drill with the same one after a certain number of holes to evaluate wear progression.

**Fig 7:**
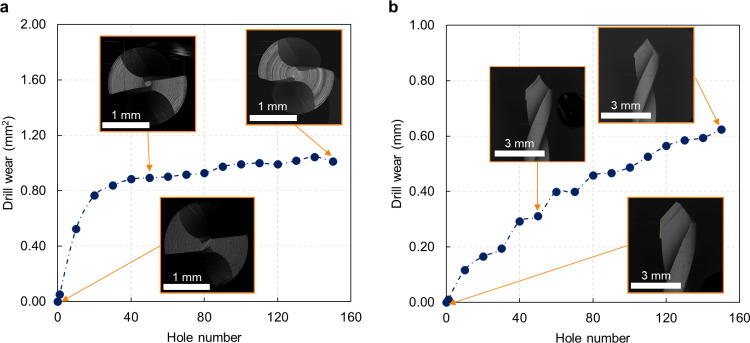
Tool wear evolution for the first drill bit in AT protocol. **A.** Worn area as a function of the hole number for the top view (SEM images represent the fresh and worn drill after 50 and 150 holes). The values represent the average of three repeats and the maximum calculated standard deviation is 0.02 mm², which is not visible in the graph. **B.** Worn cutting edge as a function of the hole number for the front view (SEM images represent the fresh and worn drill after 50 and 150 holes). The values represent the average of three repeats and the maximum calculated standard deviation is 0.01 mm, which is not visible in the graph.

As a first result, the wear evolution is represented for the AT protocol’s first step drill bit as a function of the number of holes produced (Fig 7). The whole cutting edge works to make the first 1.9 mm diameter hole. An evaluation of the worn tool, considering the fresh drill and the drill after a certain number of holes, can be seen in pictures obtained by SEM analysis. After the first few drillings, the worn area in the top view increases rapidly, and after drilling 40 holes, the entire main flank is worn, reaching 0.88 mm^2^ (Fig 7A). From there on, wear was stabilized and reached a plateau. In the front view (Fig 7B), wear increased with a fairly linear trend during the entire drilling campaign, getting a value of 0.62 mm after 150 holes.

**Fig 8:**
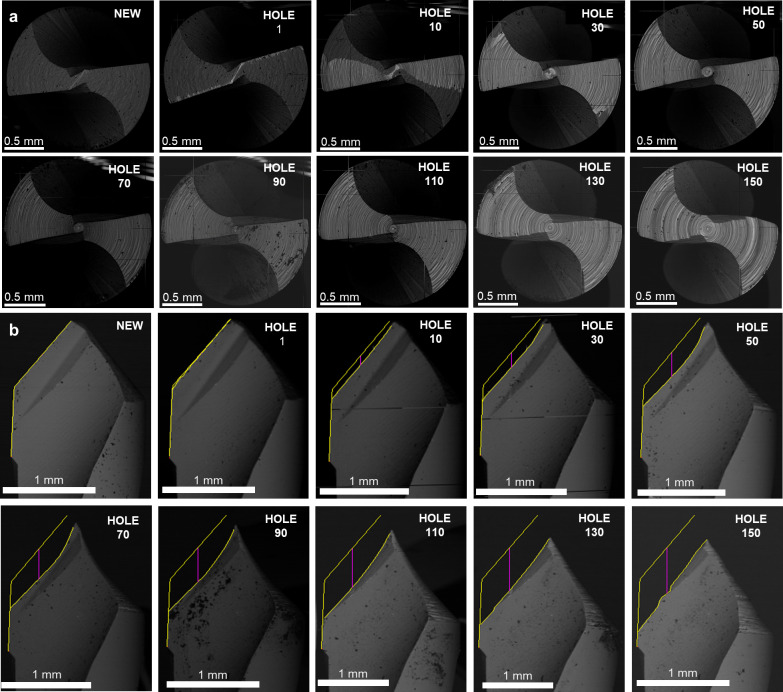
Tool wear assessment. **A.** SEM images of the worn area evolution for the first drill bit in AT protocol. The images are orthogonal to the drill axis, representing the wear evolution up to 150 drillings. In the panels, the drill bit is presented as new, after the 1st hole, and progressively every 20 holes from 10 to 150. **B.** SEM images of the worn cutting edge evolution for the first drill bit in AT protocol. The images are orthogonal to the drill axis, representing the wear evolution up to 150 drillings. In the panels, the drill bit is presented as new. after the 1st hole, and progressively every 20 holes from 10 to 150.

Fig 8 shows the wear progression from SEM images. The slight increase, from about the 50th hole, came from the deep wear developed on the central area (Fig 8A), and after drilling 100 holes, the bit becomes visibly shortened (Fig 8B). This discrepancy between the top and front views highlights the importance of considering multiple perspectives in tool wear assessment.

**Fig 9:**
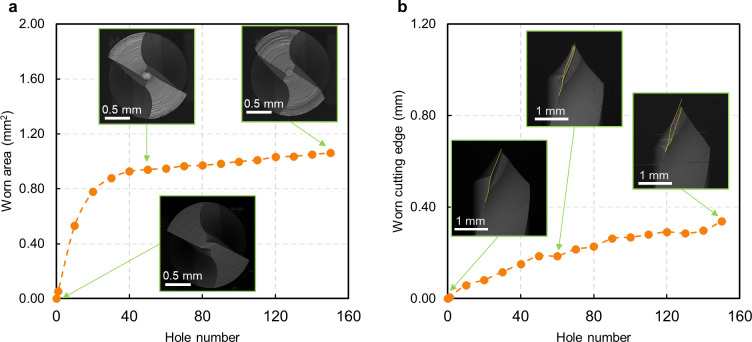
Tool wear evolution for the first drill bit in PT protocol. **A.** Worn area as a function of the hole number for the top view (SEM images represent the fresh and worn drill after 50 and 150 holes). The values represent the average of three repeats and the maximum calculated standard deviation is 0.01 mm², which is not visible in the graph. **B.** Worn cutting edge as a function of the hole number for the front view (SEM images represent the fresh and worn drill after 50 and 150 holes). The values represent the average of three repeats and the maximum calculated standard deviation is 0.01 mm, which is not visible in the graph.

**Fig 10:**
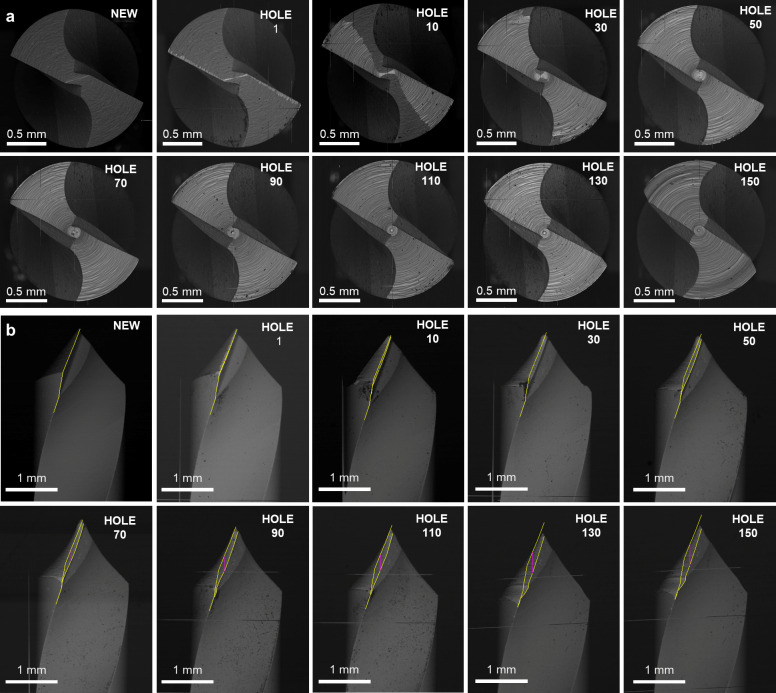
Tool wear assessment. **A.** SEM images of the worn area evolution for the first drill bit in PT protocol. The images are orthogonal to the drill axis, representing the wear evolution up to 150 drillings. In the panels, the drill bit is presented as new, after the 1st hole, and progressively every 20 holes from 10 to 150. **B.** SEM images of the worn cutting edge evolution for the first drill bit in PT protocol. The images are parallel to the drill axis, representing the wear evolution up to 150 drillings. In the panels, the drill bit is presented as new, after the 1st hole, and progressively every 20 holes from 10 to 150.

Similar considerations can be made for the first drill used for the PT protocol (Fig 9). After drilling 50 holes, the worn area was 0.94 mm^2^ (Fig 9A), then stabilized its increase and achieved 1.06 mm^2^ after 150 holes. The flank wear increased linearly, reaching 0.34 mm (Fig 9B). The evolution of the PT1 drill can be seen in Fig 10 from the SEM analysis.

The first tool geometry for both protocols and all PT drill bits is quite usual in drilling. However, in the AT protocol, bits number 2 and 3 have a stepped geometry with two cutting edges, and the AT 4^th^ bit has a distinctly different geometry from all the others. As an example, the evolution of the AT third bit will be presented (Fig 10).

**Fig 11:**
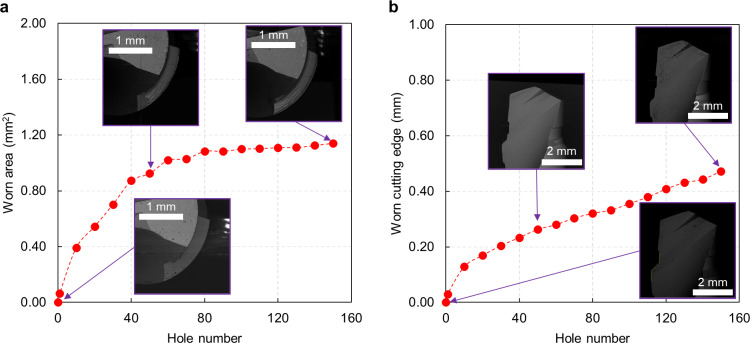
Tool wear evolution for the third drill bit in AT protocol. **A.** Worn area as a function of the hole number for the top view (SEM images represent the fresh and worn drill after 50 and 150 holes). The values represent the average of three repeats and the maximum calculated standard deviation is 0.03 mm², which is not visible in the graph. **B.** Worn cutting edge as a function of the hole number for the front view (SEM images represent the fresh and worn drill after 50 and 150 holes). The values represent the average of three repeats and the maximum calculated standard deviation is 0.01 mm, which is not visible in the graph.

**Fig 12:**
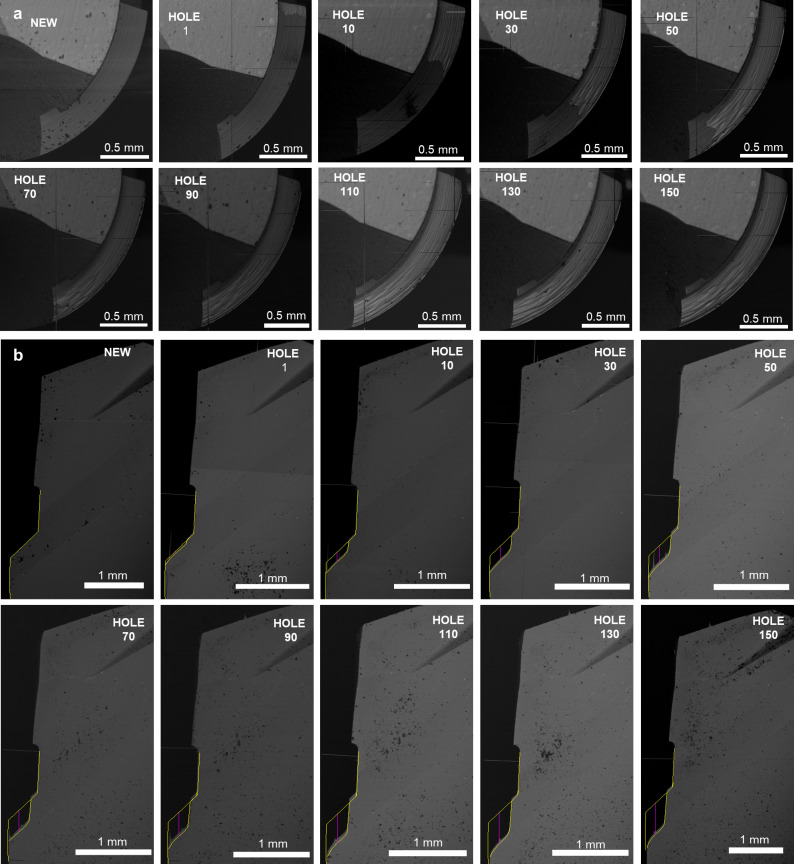
Tool wear assessment. **A.** SEM images of the worn area evolution for the third drill bit in AT protocol. The images are orthogonal to the drill axis, representing the wear evolution up to 150 drillings. In the panels, the drill bit is presented as new, after the 1st hole, and progressively every 20 holes from 10 to 150. **B.** SEM images of the worn cutting edge evolution for the third drill bit in AT protocol. The images are parallel to the drill axis, representing the wear evolution up to 150 drillings. In the panels, the drill bit is presented as new, after the 1st hole, and progressively every 20 holes from 10 to 150.

Observing the images produced by SEM (Fig 12), the top face of AT third bit did not show apparent effects of wear, even after the end of the study with 150 repeated drillings (Fig 11A). Therefore, attention was focused on the lower face, which was 3.7 mm in diameter. The wear area in the top view gradually increased until it stabilized after 60 drillings at 1.02 mm^2^ (Fig 12A). From here on, the entire area of the lower face with a diameter greater than 3.1 mm was worn, and the wear curve showed a plateau (Fig 11B).

The upper cutting edge of the AT third bit had the same diameter as the AT second bit. In the front view (Fig 12B), it did not experience apparent wear effects since it did not work the abrasive cortical layer of synthetic bone. As in the previous results, the worn area was evaluated only on the lower cutting edge. The wear increased linearly, slowing down after about 60 holes and reaching 0.47 mm after all 150 holes.

**Fig 13:**
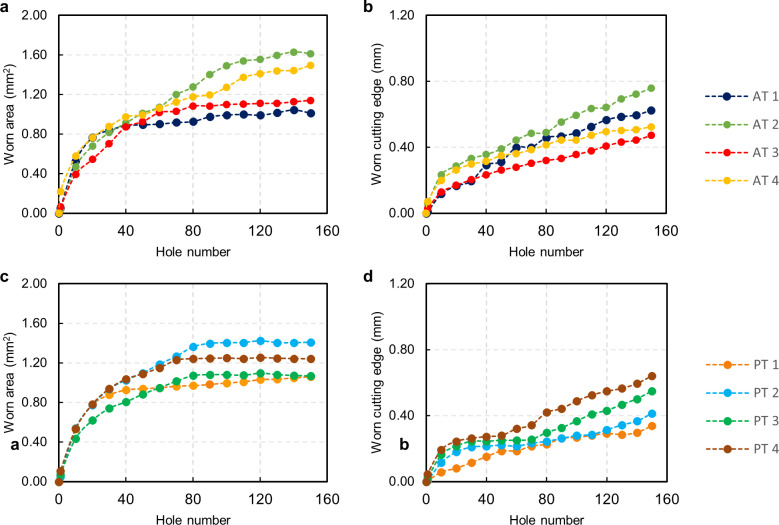
Tool wear evolution of all the drill bits. **A.** Wear curve of all the drill bits in AT protocol, as a function of the hole number, measured as worn area (the values represent the average of three repeats and the maximum calculated standard deviation is 0.03 mm², which is not visible in the graph) and **B.** as worn cutting edge (the values represent the average of three repeats and the maximum calculated standard deviation is 0.02 mm, which is not visible in the graph). **C.** Wear curve of all the drill bits in PT protocol, as a function of the hole number, measured as worn area and (the values represent the average of three repeats and the maximum calculated standard deviation is 0.02 mm², which is not visible in the graph) **D.** as worn cutting edge (the values represent the average of three repeats and the maximum calculated standard deviation is 0.01 mm, which is not visible in the graph).

Regardless of protocol or type of drill bit, wear increased quickly for the first few holes of every drill. After that, it either slowed down and increased linearly or reached a plateau (Fig 13). This might be because the drills were completely worn out after 40 to 80 drill holes. Therefore, only 50 holes will be considered for all upcoming evaluations in the study.

### Drilling temperature evaluation

**Fig 14:**
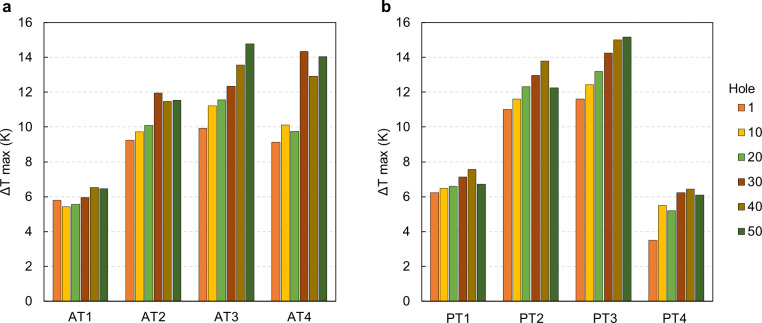
Maximum temperature differentials achieved for each drill bit, of the two protocols studied, with increasing hole number (1, 10, 20, 30, 40 and 50) during the drilling process. **A.** AT protocol. **B.** PT protocol. The maximum calculated standard deviation between the three repeats is 0.48 K.

In this section, we focus on evaluating drilling temperature, considering the results from the tool wear assessment. Temperature measurements are presented for drillings 1, 10, 20, 30, 40, and 50 to examine the impact of using drill bits beyond the manufacturer’s recommended number of uses. The results are summarized in Fig 14. For clarity, we present only the maximum temperature differences recorded by the three thermocouples, as the highest temperature poses the most significant health risk. The thermocouple positioned 1.5 mm from the surface recorded the highest temperatures in every drilling operation for both protocols.

In our experimental setup, the thermocouples were placed at a fixed distance from the hole axis to ensure consistency in data acquisition. Adjusting these distances during drilling would be difficult due to interference caused by previously drilled thermocouple holes. The maximum distance variation between the first three drill bits was 0.9 mm, while between the third and fourth bits, it was 0.25 mm, suggesting these differences won’t significantly impact temperature measurements. Additionally, the data show that temperature differentials for the second and third drill bits were higher than for the fourth drill bits, with a larger diameter, indicating that wear affects temperature increases.

Temperatures are expressed as ΔTs (temperature differentials) from the base temperature, allowing us to identify critical steps in both protocols and assess potential bone tissue damage during drilling. In particular, some critical temperature levels have been identified and should not be exceeded during bone drilling. A temperature differential of 8 K (corresponding to 45 °C) can produce cell damage. A ΔT of 10 K (47 ° C) can compromise osseointegration if maintained for more than 1 minute. Finally, 16 K (53 °C) can represent the limit for irreversible damage [8].

As reported in Fig 14A, the AT protocol’s first step showed temperature patterns similar to the PT protocol, with a maximum ΔT of 6.52 K. However, steps 2, 3, and 4 in the AT protocol exhibited critical conditions from the first use of the drill bits. The ΔTs increased with repeated use, particularly for steps 3 and 4. Notably, step 3 of the AT protocol demonstrated a significant increase in temperature difference with use, rising from 9.93 K at the first drilling to 14.78 K at the 50^th^ hole.

In the PT protocol (Fig 14B) for step 1, the maximum ΔT of 7.57 K was observed at drilling 40, while step 4 peaked at 6.64 K at drilling 30, with temperatures stabilizing thereafter. However, steps 2 and 3 displayed critical ΔTs from the first use, increasing up to 13 K difference as wear progressed. The highest ΔT of 15.16 K was recorded in step 3 at drilling 50. The varying temperature trends across different steps and protocols underscore the importance of considering drill wear and drilling parameters to prevent tissue damage during dental implant surgeries.

Table 3 reports the results obtained by the temperature acquisition and wear evaluation of all the drills of the two protocols. The value is calculated as the average of the three repetitions conducted.

**Table 3:** Wear and drilling temperature results. For each protocol and drill bit, the table presents the response variables (worn area, worn cutting edge, and ΔT max) at increasing hole numbers, ranging from 1 to 50.

Operation	Hole number	Worn area (mm^2^)	Worn cutting edge (mm)	ΔT max (K)
AT 1	1	0.05	0.01	5.8
	10	0.53	0.12	5.42
	20	0.77	0.17	5.56
	30	0.84	0.19	5.95
	40	0.88	0.29	6.52
	50	0.89	0.31	6.46
AT 2	1	0.04	0.06	9.25
	10	0.47	0.23	9.73
	20	0.68	0.29	10.09
	30	0.82	0.33	11.95
	40	0.91	0.36	11.48
	50	1.01	0.39	11.54
AT 3	1	0.07	0.03	9.93
	10	0.39	0.13	11.21
	20	0.54	0.17	11.56
	30	0.70	0.20	12.34
	40	0.87	0.23	13.57
	50	0.92	0.26	14.78
AT 4	1	0.22	0.07	9.13
	10	0.58	0.20	10.12
	20	0.76	0.26	9.74
	30	0.88	0.30	14.34
	40	0.97	0.31	12.91
	50	0.99	0.35	14.04
PT 1	1	0.05	0.01	6.24
	10	0.53	0.06	6.48
	20	0.78	0.08	6.61
	30	0.88	0.12	7.12
	40	0.93	0.15	7.57
	50	0.94	0.19	6.72
PT 2	1	0.05	0.01	11.00
	10	0.54	0.12	11.61
	20	0.78	0.18	12.31
	30	0.94	0.21	12.97
	40	1.03	0.22	13.80
	50	1.10	0.22	12.24
PT 3	1	0.07	0.02	11.60
	10	0.44	0.16	12.43
	20	0.62	0.22	13.19
	30	0.74	0.23	14.24
	40	0.81	0.24	15.00
	50	0.88	0.25	15.16
PT 4	1	0.11	0.05	3.50
	10	0.53	0.19	5.50
	20	0.78	0.24	5.20
	30	0.94	0.26	6.64
	40	1.04	0.27	6.43
	50	1.09	0.28	6.09

Pearson’s coefficient was evaluated for each drill. Almost all the measures had a strong correlation, showing *r* >  0.70. The correlation was nearly perfect for some quantity, with *r* near 1. For instance, the third drill bit, which reached the highest measured temperature, strongly correlates wear rise and temperature rise in both protocols (*r* =  0.96). The AT protocol third drill bit reached 0.92 mm^2^ of worn area after 50 drillings, correlating with a temperature differential of 14.78 K, the highest achieved in the protocol. The PT protocol exhibited a 15.16 K temperature difference after 50 drillings, with 0.88 mm^2^ of worn area.

### Drilled hole quality

**Fig 15:**
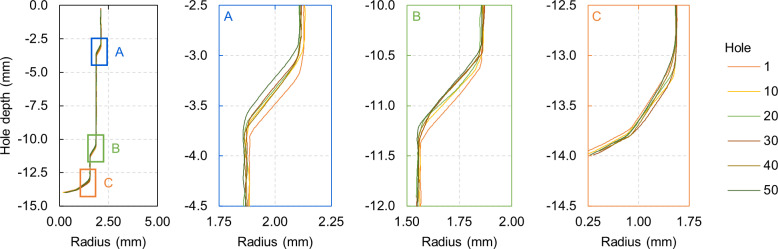
Average hole profiles for AT protocol after incremental drillings from 1 to 50 every ten holes. With details of the upper **A.**, middle **B.**, and lower zones detail **C.**. The maximum calculated standard deviation between the profiles of a specific hole is 0.06 mm.

In this section, we report the results of geometrical measurements performed on drilled holes using the CMM. These measurements include qualitative and quantitative evaluations for the PT and AT protocols. While similar analyses apply to both protocols, we focus primarily on the AT protocol due to its unique three-zone profile geometry (Fig 15), compared to the PT protocol’s two-zone profile. However, findings for the AT protocol also indicate trends observable in the PT protocol.

Throughout the drilling process, we noted that the diameter of the holes, as measured with four points extracted from the profile analysis, remained consistent, with a deviation from the first drill lower than 0.1 mm. However, variations in hole depth were observed, particularly in zones where the cutting edges of drill bits 3 and 4 had worn. This wear was most evident in the first “step” of the profile (worked by drill bit 4) and the second and third steps (worked by drill bit 3). As a result, we conducted a detailed analysis of these three zones.

The analysis revealed a clear progression in hole geometry in the upper and middle zones, with a reduction in drilling depth as bit wear increased. Specifically, after 50 drillings, the depth in the upper zone decreased by approximately 0.27 mm, while the middle zone saw a decrease of about 0.17 mm. The PT protocol’s main depth difference was 0.13 mm in the upper zone from drillings 1 to 50.

From the implant volume analysis for the AT protocol, 151 mm^3^ of bone was removed for each implantation, while for the PT protocol, the amount was 143 mm^3^. There was only a marginal variation in the volume intersection from the first to the fiftieth hole, specifically 1.9% for the first protocol and 0.9% for the second.

## Temperature and torque at implant placement

**Fig 16:**
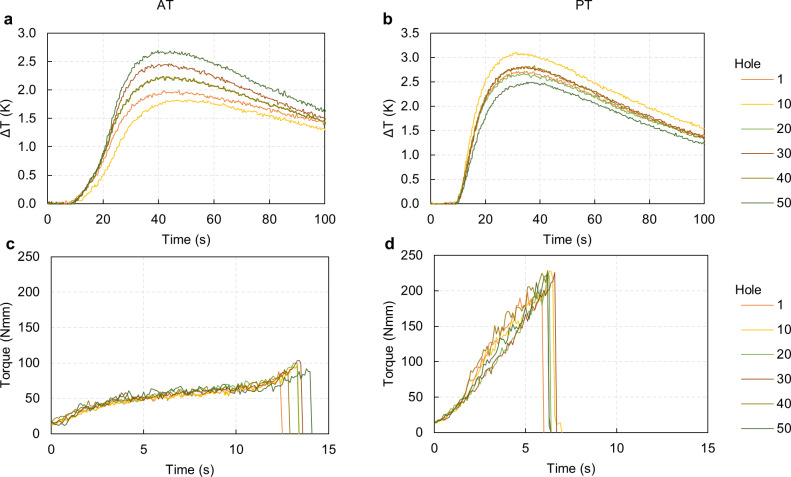
Temperature and torque insertion measurements. **A.** Curve of time-dependent temperature differential for AT protocol. The maximum calculated standard deviation between the three repetitions is 0.59 K. **B.** Curve of time-dependent temperature differential for PT protocol. The maximum calculated standard deviation between the three repetitions is 0.62 K. **C.** Curve of time-dependent torque for AT protocol. The maximum calculated standard deviation between the three repetitions is 4.1 Nmm. **D.** Curve of time-dependent torque for PT protocol. The maximum calculated standard deviation between the three repetitions is 5.3 Nmm.

In this final section of the results, we present the findings related to temperature increases and torque during prosthesis implantation. As observed in the drilling temperature measurements, the thermocouple placed 1.5 mm from the top consistently recorded the highest ΔTs values during implantation for both the AT and PT protocols. The thermocouples at 7 mm and 12 mm from the top surface measured maximum ΔTs slightly above 2.00 K and about 1.50 K, respectively. The first thermocouple showed more significant temperature variations. Therefore, we focus the discussion on the results of this first thermocouple (Fig 16).

In the AT protocol, ΔTs ranged between approximately 1.75 K and 2.75 K relative to the pre-operation synthetic bone temperature, with the highest value of 2.70 K occurring at hole number 50 (Fig 16A). In contrast, the PT protocol exhibited higher maximum ΔTs, reaching 3.10 K at the 10^th^ drilling (Fig 16B).

Regarding torque during prosthesis implantation, both protocols recorded values well below the maximum recommended limit of 450 Nmm. For the AT protocol, the average duration of the operation was about 13 seconds, with increasing torque values peaking towards the end of the engagement as the implant was almost entirely screwed into the hole. Torque values remained comparable across different drillings, but a notable difference was observed in the operation duration: the engagement in the first hole was the shortest, while the 50^th^ hole engagement took the longest (Fig 16C). The PT protocol exhibited torque values approximately twice as high as the AT protocol. Here, maximum torque values were reached at the end of the operation, which lasted around 7 seconds—about half the duration recorded for the AT protocol. Similar to the AT protocol, torque values in the PT protocol were consistent across different drillings. However, the duration of the operation was more uniform, with only about a 1 second difference between the shortest and the longest engagements (Fig 16D).

These findings on temperature increases and torque during prosthesis implantation highlight the differences in operational dynamics between the AT and PT protocols, as discussed later in the Discussion section.

## Discussion

The absence of a standardized surgical protocol for utilizing drill bits in dental implant surgeries often leads to their usage beyond the manufacturer’s recommended limits. Traditionally, the decision to discard surgical tools is based on the visual assessment of cutting edges or the surgeon’s perception of drilling forces [24]. This study underscores the necessity for real-time monitoring systems to evaluate biological damage to bone during drilling, which presents a challenge for dental clinics due to the complexity of drilling operations.

The drilling speed and feed rate have been found to impact the amount of heat generated during drilling significantly and, consequently, the potential for bone damage. Studies have shown that lower drilling speeds can reduce the increase in temperature, leading to better outcomes in terms of bone regeneration [25]. Similarly, the drill diameter and the feed rate have been observed to influence the temperature in the drilling zone, with higher feed rates resulting in lower temperature increases [26,27]. The applied drilling force is another parameter that affects temperature variation, with higher forces typically leading to lower increases in temperature. Additionally, the incorporation of vibrational drilling techniques has been explored as a means to modulate the rise in temperature during drilling. Specifically, the combination of low drilling speed and feed rate with controlled micro-vibrations has been identified as a favorable approach for reducing thermal risks during bone drilling [28,29]. In our experimental procedure, we employed input parameters commonly utilized in practical applications. These parameters were deliberately maintained at a constant level to prevent any potential influence on the data collected during the experiment.

Considering the critical importance of temperature management during bone drilling, this study focused on monitoring temperature and wear variations as key indicators of the quality of the drilling process. Various methods have been employed in past research for temperature acquisition, with thermocouples commonly used for reliable data collection in bone drilling and implantation operations [30–34]. On the other hand, wear monitoring involves different evaluation methods, as there is no standardized approach for assessing drill bit wear. In-situ measurements and visual evaluations have been used in previous studies to track the wear progression on drill bits. For example, a multi-sensor approach and an artificial neural network algorithm were applied in one study to assess drill bit wear during the operation [35]. Another study used scanning electron microscopy (SEM) images of drills to analyze wear evolution, observing degradation and corrosion on the cutting surfaces after repeated use [36]. One more employed a microscope to capture images of drill bit wear, noting increasing wear on the cutting edges with repeated use [37].

One of the primary objectives of this in vitro research was to explore the impact of drill wear on temperature elevation during the drilling process. We utilized parameters closely resembling those employed in actual dental implantations to assess the risk of reaching temperatures detrimental to health. The use of advanced techniques, including SEM analysis, CMM measurements, and continuous temperature monitoring, provided a comprehensive understanding of drill bits’ wear behavior, geometrical alterations of the obtained holes, and thermal dynamics associated with the drilling and dental implant processes. The chosen methodologies aimed to replicate real-world scenarios by introducing controlled variables, contributing to the reliability and relevance of the results obtained. The findings revealed that drilling is the most critical phase, producing the highest temperature increase compared to the subsequent implantation step. Crucially, surpassing the manufacturer’s recommended limits for drill bit use significantly increased temperature elevation, posing risks to patient health. The repeated use of drilling tools leads to significant wear on the cutting edges and a substantial increase in temperature in the cutting zone, which may cause tissue damage.

**Fig 17:**
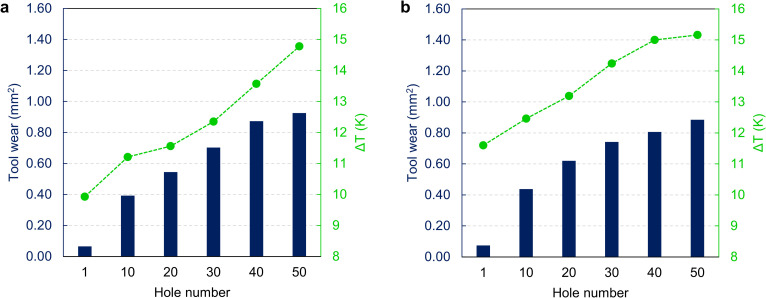
Correlation between temperature differentials and tool wear during drilling for the third drill bit for each protocol. **The graph illustrates the strong correlation between the increase in temperature and the rise in wear. A.** AT protocol. The maximum calculated standard deviation between the three repetitions is 0.03 mm² for the worn area and 0.41 K, which are not visible in the graph. **B.** PT protocol. The maximum calculated standard deviation between the three repetitions is 0.01 mm² for the worn area and 0.44 K, which are not visible in the graph.

A striking correlation was observed between the progression of drill wear and the rise in synthetic bone temperature before the drill was entirely worn out. During the initial series of 40 to 60 drillings—before the wear on tools stabilized at roughly 90% —the temperature increased by approximately 20% between the first and the 50th holes. In this in vitro study, the higher r value was obtained for both protocols in the third drill bits on the correlation between the worn area and the maximum temperature reached, with a value of 96. As shown in Fig 17, the two quantities arise linearly, reaching the peak in the last hole. The temperature increase and the wear for the AT protocol were 49% and 62%, respectively, from the first to the 50th drilling, while 31% and 81% were for the PT protocol. As drill bits were increasingly utilized, the blunting of their flank faces and cutting edges led to a distorted cutting area geometry. This alteration in geometry changed the nature of the cutting operation to more of a sliding action rather than a clean cutting, resulting in increased friction and heat generation between the drill and the synthetic bone [3].

The investigation into the geometry of the drilled holes was intended to explore any potential correlation with the drills’ wear or the implant’s subsequent insertion [38]. Despite the significant wear and temperature variations during the drilling phase, the geometry of the holes remained relatively consistent. There was only a slight variation in the evolution of the holes’ profile, suggesting a less evident, yet existent, relationship with worn tools. The most noticeable differences were observed in the depth of the holes, particularly in the areas where the cutting edges engaged in material removal while hole diameters remained unchanged. The holes’ depth decreased nearly linearly for both protocols. The most significant geometric changes were noted in the AT protocol, where, in alignment with the front view measurements of tool wear, the hole depth was reduced by 0.27 mm in the upper zone and 0.17 mm in the middle zone. In the PT protocol, the depth difference between the first and the fiftieth hole in the upper zone was 0.13 mm. Due to the geometry of the stylus tip used for the measure, the lower zone did not show a regular trend. The findings are consistent with other studies showing that wear progression, influenced by cutting speed and feed rate, can affect hole surface roughness. However, its impact on the circularity and shape of holes is smaller. Instead, wear influences the depth consistency of a hole more than its overall shape or size, aligning with our observations of minor changes in hole depth but stable diameters and profiles [39].

This minor change in hole geometry from the initial to the final drilling could explain the minimal variation in volume intersection between the drilled bone and the implant. The latter revealed a 1.9% intersection in the AT protocol and a 0.9% intersection in the PT protocol, indicating a precise fit with minimal discrepancy. This stability resulted in lower recorded torque and temperature levels during the implantation phase, suggesting a minimal impact on the implant’s structural integration.

The torque values consistently adhered to the limits set by the protocols. This indicates that the implantation process remains safe and effective within the defined operational parameters despite the challenges posed during the drilling phase.

Given these findings, it becomes evident that while the drilling phase poses significant risks, careful management of tool wear and operational parameters can mitigate these risks, ensuring the safety and effectiveness of the dental implantation process.

The study has some limitations. Firstly, it was conducted in vitro conditions using synthetic bone without irrigation. While this setup ensures consistency, it does not fully replicate clinical scenarios. Future studies incorporating irrigation would provide a more accurate assessment of temperatures and drill wear. Secondly, the study employed a machine-controlled drilling procedure to minimize variability and ensure repeatability. However, this approach does not account for human variability and diverges from the conditions in clinical reality.

Expanding this research to include in vivo studies incorporating irrigation and manual drilling would provide valuable insights into the clinical implications of these findings. This could potentially lead to improved protocols that enhance patient safety and treatment outcomes in dental implantation procedures. Exploring advanced drill bit designs and different synthetic bone block materials could further improve the study.

## Conclusions

The current study’s results, despite the limitations of the in vitro conditions, showed a strong correlation between tool wear and drilling temperature, outlining the importance of proper drill bit usage. Nevertheless, the hole geometry stayed relatively consistent, conducting to temperature and torque implantation values not higher than the limitations. The drilling phase introduced a higher risk of potential damage than the implantation phase in both examined protocols. Future research should investigate different combinations of operational parameters to understand their impact on both the drilling and implantation phases.

## Supporting information

S1 Table Drilling temperature profile.
The S1 Table provides the raw data used to generate the temperature profiles in Fig 3. The data represents the temperature increments recorded by the three thermocouples during the first drilling operation. The increments were automatically calculated by subtracting the room temperature from the acquired absolute values.
(PDF)

S2 Table Mean hole profile.
The data presented in S2 Table were used to generate the hole profiles in Fig 6. The table reports only the mean values calculated from three different measurements. For more information about measurement deviations, please refer to S5 Table.
(PDF)

S3 Table Wear measurements.
This table presents all the data obtained from the wear assessment. Specifically, it includes the worn area and cutting edge wear measurements for both the AT and PT protocols, recorded from hole 1 to hole 150 at intervals of every 10 holes.
(PDF)

S4 Table Maximum temperature during drilling operations.
S4 Table reports the maximum temperature variations recorded during the drilling experiments.
(PDF)

S5 Table Hole profiles – AT protocol.
S5 Table presents the measurements obtained using the CMM machine for holes numbered 1, 10, 20, 30, 40, and 50. Each measurement was repeated three times, and the mean value was calculated and reported.
(PDF)

S6 Table Insertion temperature - AT.
In this table, the average temperature increment during insertion for the AT protocol is presented.
(PDF)

S7 Table Insertion temperature - PT.
In this table, the average temperature increment during insertion for the PT protocol is presented.
(PDF)

S8 Table Insertion torque - AT.
In this table, the average torque values during insertion for the AT protocol are presented
(PDF)

S9 Table Insertion torque - PT.
In this table, the average torque values during insertion for the PT protocol are presented.
(PDF)
