# Supplementary material for: Impact of drill bit wear on temperature increase in dental implant osteotomy: an in vitro study
Source: PLoS One. 2025 Mar 19;20(3):e0319492. doi: 10.1371/journal.pone.0319492 (PMC11922234; doi:10.1371/journal.pone.0319492)
Supplement: S1 Table — The S1 Table provides the raw data used to generate the temperature profiles in Fig 3. The data represents the temperature increments recorded by the three thermocouples during the first drilling operation. The increments were automatically calculated by subtracting the room temperature from the acquired absolute values. (PDF) [file pone.0319492.s001.pdf]

Protocol	AT	
Drill bit	1	
Room temp	27.45	°C
Run	1	/3

Time (sec)	ΔT1 (K)	ΔT2 (K)	ΔT3 (K)
0.250	0.01	0.03	0.04
0.501	-0.03	0.01	0.06
0.750	-0.03	0.07	0.06
1.000	0.01	0.07	0.06
1.251	0.02	0.03	0.09
1.500	0.02	0.03	0.09
1.750	-0.01	0.02	0.03
2.000	-0.01	0.03	0.03
2.251	-0.02	0.03	0.02
2.500	0.02	0.03	0.02
2.750	0.02	0.03	-0.01
3.001	-0.01	0.00	0.03
3.250	-0.01	0.00	0.03
3.500	-0.02	0.00	-0.02
3.750	-0.02	0.03	-0.02
4.000	-0.02	0.03	-0.01
4.250	0.01	0.02	-0.01
4.500	0.01	0.02	-0.01
4.751	0.00	-0.01	-0.02
5.000	0.00	-0.01	-0.02
5.250	0.03	0.01	0.00
5.500	-0.01	0.00	0.00
5.751	-0.01	0.00	-0.06
6.000	0.02	0.00	-0.04
6.250	0.02	0.00	-0.04
6.501	-0.01	-0.02	-0.07
6.750	-0.01	-0.04	-0.07
7.000	0.00	-0.04	-0.07
7.250	0.00	0.02	-0.07
7.500	0.00	0.02	-0.07
7.750	0.00	0.00	-0.07
8.000	0.00	0.00	-0.07
8.251	0.01	0.00	-0.10
8.500	0.01	0.00	-0.10
8.750	0.02	0.00	-0.09
9.000	-0.02	-0.02	-0.11
9.250	-0.02	-0.02	-0.11
9.500	0.03	-0.04	-0.08
9.750	0.03	-0.02	-0.08
10.001	0.01	-0.02	-0.10
10.250	0.01	-0.02	-0.10
10.500	0.01	-0.02	-0.11
10.751	-0.02	-0.03	-0.13
11.000	-0.02	-0.03	-0.13
11.250	0.01	-0.03	-0.13
11.500	0.01	-0.03	-0.13
11.750	-0.02	-0.03	-0.11
12.000	0.00	-0.03	-0.14
12.250	0.00	-0.03	-0.14

Protocol	PT	
Drill bit	1	
Room temp	27.90	°C
Run	1	/3

Time (sec)	ΔT1 (K)	ΔT2 (K)	ΔT3 (K)
0.249	0.03	0.01	0.15
0.499	0.05	0.01	0.15
0.749	0.06	0.01	0.18
1.000	0.06	0.01	0.18
1.249	0.01	0.01	0.17
1.499	0.01	0.01	0.15
1.750	0.04	0.01	0.15
1.999	0.04	0.01	0.12
2.249	0.03	-0.03	0.12
2.499	0.03	-0.03	0.15
2.750	0.03	0.00	0.12
2.999	0.04	0.00	0.12
3.249	0.04	0.01	0.08
3.500	0.04	0.01	0.08
3.749	0.00	0.00	0.08
3.999	0.00	0.02	0.08
4.249	0.02	0.02	0.10
4.500	0.02	-0.02	0.07
4.749	0.01	-0.02	0.07
4.999	0.01	-0.02	0.02
5.250	0.01	0.02	0.02
5.499	0.01	0.02	0.00
5.749	0.01	-0.02	-0.01
5.999	0.03	-0.02	-0.01
6.250	0.03	-0.02	0.00
6.499	-0.01	-0.02	0.00
6.749	-0.01	-0.03	0.00
7.000	0.03	-0.01	0.00
7.249	0.03	-0.01	-0.05
7.499	0.03	-0.01	-0.05
7.749	0.02	-0.01	-0.05
8.000	0.02	0.00	-0.09
8.249	0.03	-0.03	-0.09
8.499	0.00	-0.03	-0.10
8.750	0.00	-0.04	-0.08
8.999	0.03	-0.04	-0.08
9.249	0.03	-0.02	-0.13
9.499	0.00	-0.02	-0.13
9.750	0.00	-0.05	-0.10
9.999	0.02	-0.05	-0.10
10.249	-0.01	-0.05	-0.14
10.500	-0.01	-0.04	-0.15
10.749	0.02	-0.04	-0.15
10.999	0.02	-0.05	-0.15
11.249	0.00	-0.05	-0.15
11.500	0.00	-0.05	-0.20
11.749	0.00	-0.05	-0.20
11.999	-0.01	-0.05	-0.16
12.249	-0.01	-0.05	-0.23

12.501	-0.02	-0.01	-0.14	12.500	-0.01	-0.05	-0.23
12.750	-0.02	-0.05	-0.14	12.749	-0.01	-0.06	-0.23
13.000	-0.01	-0.05	-0.17	12.999	0.03	-0.06	-0.23
13.251	0.02	-0.06	-0.17	13.250	-0.01	-0.06	-0.22
13.500	0.02	-0.06	-0.17	13.499	-0.01	-0.06	-0.26
13.750	0.00	-0.01	-0.17	13.749	0.00	-0.06	-0.26
14.000	0.00	-0.01	-0.17	13.999	0.00	-0.07	-0.27
14.251	0.03	-0.06	-0.18	14.250	0.02	-0.07	-0.27
14.500	0.03	-0.03	-0.18	14.499	0.00	-0.05	-0.25
14.750	-0.02	-0.03	-0.19	14.749	0.00	-0.05	-0.25
15.001	-0.02	-0.04	-0.16	14.999	0.01	-0.05	-0.29
15.250	-0.02	-0.04	-0.16	15.249	0.01	-0.07	-0.29
15.500	0.04	-0.04	-0.22	15.499	0.00	-0.07	-0.29
15.750	0.04	-0.04	-0.22	15.749	0.00	-0.06	-0.30
16.001	0.00	-0.07	-0.22	16.000	0.03	-0.06	-0.30
16.250	0.04	-0.05	-0.22	16.249	0.01	-0.06	-0.32
16.500	0.04	-0.05	-0.21	16.499	0.01	-0.07	-0.34
16.751	0.00	-0.03	-0.18	16.749	0.02	-0.07	-0.34
17.000	0.00	-0.03	-0.18	17.000	0.02	-0.06	-0.24
17.250	0.01	-0.07	-0.22	17.249	0.01	-0.06	-0.24
17.500	0.01	-0.05	-0.22	17.499	0.01	-0.06	-0.07
17.751	0.01	-0.05	-0.22	17.750	0.02	-0.06	-0.07
18.000	0.00	-0.06	-0.21	17.999	0.02	-0.06	0.17
18.250	0.00	-0.06	-0.21	18.249	0.02	-0.07	0.62
18.501	0.00	0.01	-0.22	18.499	0.03	-0.07	0.62
18.750	0.00	0.01	-0.22	18.750	0.03	-0.05	1.15
19.000	-0.02	0.03	-0.25	18.999	0.04	-0.05	1.15
19.250	-0.02	0.08	-0.25	19.249	0.02	-0.05	1.68
19.501	0.01	0.08	-0.26	19.499	0.02	-0.05	2.28
19.750	-0.02	0.23	-0.27	19.750	0.04	-0.05	2.28
20.000	-0.02	0.23	-0.27	19.999	0.04	-0.06	2.89
20.251	0.00	0.38	-0.28	20.249	0.09	-0.06	2.89
20.500	0.00	0.61	-0.28	20.500	0.09	-0.05	3.48
20.750	0.00	0.61	-0.26	20.749	0.12	-0.06	3.48
21.000	-0.01	0.85	-0.26	20.999	0.20	-0.06	4.02
21.250	-0.01	0.85	-0.27	21.249	0.20	-0.06	4.55
21.500	0.00	1.17	-0.29	21.500	0.32	-0.06	4.55
21.750	0.00	1.17	-0.29	21.749	0.32	-0.05	5.03
22.001	0.02	1.46	-0.28	21.999	0.45	-0.05	5.03
22.250	0.02	1.83	-0.28	22.250	0.56	-0.05	5.42
22.500	0.01	1.83	-0.27	22.499	0.56	-0.08	5.42
22.750	-0.02	2.18	-0.31	22.749	0.72	-0.08	5.85
23.000	-0.02	2.18	-0.31	22.999	0.72	-0.06	6.22
23.250	0.01	2.54	-0.27	23.250	0.88	-0.06	6.22
23.500	0.01	2.88	-0.27	23.499	0.88	-0.06	6.53
23.751	0.00	2.88	-0.30	23.749	1.06	-0.06	6.53
24.000	0.01	3.24	-0.30	24.000	1.23	-0.06	6.83
24.250	0.01	3.24	-0.32	24.249	1.23	-0.06	7.27
24.501	-0.01	3.59	-0.33	24.499	1.41	-0.06	7.27
24.750	-0.01	3.59	-0.33	24.749	1.41	-0.07	7.64
25.000	-0.01	3.96	-0.31	25.000	1.61	-0.07	7.64
25.250	-0.01	4.51	-0.31	25.249	1.76	-0.07	8.01
25.501	-0.01	4.51	-0.33	25.499	1.76	-0.07	8.01
25.750	0.02	4.94	-0.35	25.750	1.96	-0.07	8.33
26.000	0.02	4.94	-0.35	25.999	1.96	-0.07	8.69
26.250	0.00	5.32	-0.36	26.249	2.13	-0.07	8.69

26.500	0.00	5.32	-0.36
26.750	0.01	5.68	-0.38
27.000	0.00	5.95	-0.38
27.251	0.00	5.95	-0.36
27.500	0.05	6.29	-0.34
27.750	0.05	6.29	-0.34
28.001	0.04	6.56	-0.31
28.250	0.04	6.82	-0.31
28.500	0.03	6.82	-0.23
28.750	0.06	7.03	-0.07
29.001	0.06	7.03	-0.07
29.250	0.14	7.26	0.22
29.500	0.14	7.26	0.22
29.751	0.21	7.41	0.61
30.000	0.21	7.58	0.61
30.250	0.29	7.58	1.11
30.500	0.44	7.72	1.72
30.750	0.44	7.72	1.72
31.000	0.55	7.93	2.25
31.250	0.55	8.00	2.25
31.501	0.70	8.00	2.71
31.750	0.84	8.13	2.71
32.000	0.84	8.13	3.20
32.250	1.04	8.30	3.69
32.500	1.04	8.30	3.69
32.750	1.19	8.46	4.13
33.000	1.19	8.68	4.13
33.251	1.39	8.68	4.59
33.500	1.57	8.79	4.96
33.750	1.57	8.79	4.96
34.001	1.77	8.96	5.33
34.250	1.77	9.05	5.33
34.500	1.92	9.05	5.71
34.750	2.09	9.16	5.71
35.001	2.09	9.16	6.12
35.250	2.27	9.17	6.52
35.500	2.27	9.17	6.52
35.751	2.42	9.22	7.08
36.000	2.42	9.24	7.08
36.250	2.58	9.24	7.51
36.500	2.73	9.24	7.81
36.751	2.73	9.24	7.81
37.000	2.88	9.23	8.10
37.250	2.88	9.23	8.10
37.501	3.03	9.24	8.29
37.750	3.17	9.21	8.29
38.000	3.17	9.21	8.45
38.250	3.30	9.16	8.74
38.501	3.30	9.16	8.74
38.750	3.44	9.10	8.76
39.000	3.44	9.09	8.76
39.251	3.55	9.09	8.73
39.500	3.68	9.10	8.83
39.750	3.68	9.10	8.83
40.000	3.81	8.99	9.02
40.251	3.81	8.99	9.02

26.499	2.13	-0.05	9.02
26.750	2.29	-0.05	9.02
26.999	2.46	-0.05	9.32
27.249	2.46	0.01	9.55
27.500	2.64	0.01	9.55
27.749	2.64	0.20	9.71
27.999	2.79	0.20	9.71
28.249	2.79	0.51	9.88
28.500	2.95	0.56	9.88
28.749	3.10	0.56	10.01
28.999	3.10	0.88	10.13
29.250	3.26	0.88	10.13
29.499	3.26	1.51	10.34
29.749	3.43	1.56	10.34
29.999	3.52	1.56	10.38
30.250	3.52	2.02	10.45
30.499	3.69	2.02	10.45
30.749	3.69	2.61	10.63
31.000	3.85	2.61	10.63
31.249	3.85	3.11	10.91
31.499	3.97	3.22	10.91
31.749	4.11	3.22	11.16
32.000	4.11	3.60	11.37
32.249	4.21	3.60	11.37
32.499	4.21	4.18	11.48
32.750	4.33	4.28	11.48
32.999	4.46	4.28	11.51
33.249	4.46	4.68	11.51
33.499	4.54	4.68	11.55
33.750	4.54	5.05	11.51
33.999	4.69	5.05	11.51
34.249	4.69	5.56	11.50
34.500	4.84	5.61	11.50
34.749	5.01	5.61	11.40
34.999	5.01	5.96	11.36
35.249	5.17	5.96	11.36
35.500	5.17	6.41	11.30
35.749	5.32	6.41	11.30
35.999	5.45	6.82	11.20
36.250	5.45	6.86	11.20
36.499	5.60	6.86	11.13
36.749	5.60	7.20	11.04
36.999	5.72	7.20	11.04
37.250	5.72	7.62	10.94
37.499	5.81	7.66	10.94
37.749	5.88	7.66	10.86
38.000	5.88	7.96	10.75
38.249	5.95	7.96	10.75
38.499	5.95	8.32	10.66
38.749	6.04	8.32	10.66
39.000	6.04	8.62	10.55
39.249	6.07	8.66	10.55
39.499	6.14	8.66	10.48
39.750	6.14	8.88	10.36
39.999	6.17	8.88	10.36
40.249	6.17	9.21	10.28

40.500	3.92	8.93	9.18	40.499	6.16	9.24	10.28
40.750	3.92	8.91	9.18	40.750	6.20	9.24	10.15
41.001	4.04	8.91	9.40	40.999	6.20	9.48	10.06
41.250	4.14	8.84	9.58	41.249	6.22	9.48	10.06
41.500	4.14	8.84	9.58	41.500	6.22	9.82	9.96
41.751	4.26	8.77	9.68	41.749	6.18	9.82	9.96
42.000	4.26	8.70	9.68	41.999	6.18	10.13	9.84
42.250	4.34	8.70	9.74	42.249	6.23	10.19	9.84
42.500	4.43	8.66	9.74	42.500	6.24	10.19	9.76
42.751	4.43	8.66	9.78	42.749	6.24	10.40	9.62
43.000	4.61	8.61	9.74	42.999	6.21	10.40	9.62
43.250	4.61	8.61	9.74	43.250	6.21	10.66	9.53
43.501	4.75	8.52	9.76	43.499	6.26	10.69	9.53
43.750	4.75	8.46	9.76	43.749	6.21	10.69	9.42
44.000	4.87	8.46	9.73	43.999	6.21	10.80	9.42
44.250	5.00	8.39	9.71	44.250	6.19	10.80	9.34
44.501	5.00	8.39	9.71	44.499	6.19	10.88	9.20
44.750	5.08	8.32	9.66	44.749	6.20	10.88	9.20
45.000	5.08	8.27	9.66	45.000	6.20	10.94	9.09
45.250	5.19	8.27	9.57	45.249	6.16	10.95	9.09
45.500	5.29	8.21	9.57	45.499	6.15	10.95	9.03
45.750	5.29	8.21	9.51	45.749	6.15	10.96	8.91
46.000	5.38	8.14	9.46	45.999	6.14	10.96	8.91
46.251	5.38	8.14	9.46	46.249	6.14	10.95	8.84
46.500	5.47	8.11	9.38	46.499	6.13	10.95	8.84
46.750	5.47	8.03	9.38	46.750	6.08	10.92	8.75
47.000	5.49	8.03	9.31	46.999	6.08	10.92	8.75
47.250	5.57	7.94	9.24	47.249	6.05	10.92	8.67
47.500	5.57	7.94	9.24	47.500	6.05	10.88	8.60
47.750	5.63	7.89	9.17	47.749	6.02	10.88	8.60
48.001	5.63	7.89	9.17	47.999	6.02	10.82	8.50
48.250	5.69	7.81	9.09	48.249	6.01	10.81	8.50
48.500	5.73	7.78	9.09	48.500	6.00	10.81	8.41
48.750	5.73	7.78	9.03	48.749	6.00	10.76	8.30
49.000	5.78	7.68	8.94	48.999	6.00	10.76	8.30
49.250	5.78	7.68	8.94	49.250	6.00	10.66	8.21
49.500	5.78	7.64	8.85	49.499	5.96	10.66	8.21
49.751	5.78	7.55	8.85	49.749	5.96	10.59	8.12
50.000	5.76	7.55	8.76	49.999	5.87	10.58	8.12
50.250	5.80	7.53	8.71	50.250	5.86	10.58	8.05
50.501	5.80	7.53	8.71	50.499	5.86	10.53	7.95
50.750	5.79	7.47	8.66	50.749	5.83	10.53	7.95
51.000	5.79	7.47	8.66	51.000	5.83	10.42	7.86
51.250	5.78	7.38	8.55	51.249	5.80	10.42	7.86
51.501	5.78	7.32	8.55	51.499	5.77	10.42	7.76
51.750	5.75	7.32	8.49	51.749	5.77	10.34	7.69
52.000	5.74	7.24	8.42	52.000	5.73	10.34	7.69
52.251	5.74	7.24	8.42	52.249	5.73	10.25	7.60
52.500	5.72	7.20	8.33	52.499	5.67	10.25	7.60
52.750	5.72	7.17	8.33	52.750	5.67	10.16	7.49
53.000	5.70	7.17	8.26	52.999	5.67	10.15	7.49
53.251	5.71	7.10	8.26	53.249	5.62	10.15	7.44
53.500	5.71	7.10	8.13	53.499	5.62	10.07	7.35
53.750	5.66	7.05	8.11	53.750	5.63	10.07	7.35
54.001	5.66	7.05	8.11	53.999	5.63	9.96	7.24
54.250	5.63	6.99	8.02	54.249	5.55	9.94	7.24

54.500	5.63	6.94	8.02	54.500	5.56	9.94	7.17
54.750	5.65	6.94	7.93	54.749	5.56	9.88	7.17
55.001	5.59	6.87	7.89	54.999	5.48	9.88	7.10
55.250	5.59	6.87	7.89	55.249	5.48	9.80	7.01
55.500	5.61	6.83	7.77	55.500	5.47	9.80	7.01
55.751	5.61	6.80	7.77	55.749	5.47	9.69	6.95
56.000	5.56	6.80	7.74	55.999	5.40	9.68	6.95
56.250	5.54	6.71	7.74	56.250	5.39	9.68	6.86
56.500	5.54	6.71	7.65	56.499	5.39	9.60	6.81
56.751	5.52	6.67	7.61	56.749	5.34	9.60	6.81
57.000	5.52	6.67	7.61	56.999	5.34	9.51	6.73
57.250	5.49	6.61	7.53	57.250	5.34	9.51	6.73
57.501	5.49	6.56	7.53	57.499	5.28	9.42	6.64
57.750	5.46	6.56	7.45	57.749	5.28	9.41	6.64
58.000	5.42	6.53	7.39	58.000	5.24	9.41	6.55
58.250	5.42	6.53	7.39	58.249	5.24	9.32	6.50
58.501	5.40	6.48	7.31	58.499	5.23	9.32	6.50
58.750	5.40	6.48	7.31	58.749	5.23	9.22	6.45
59.000	5.38	6.40	7.26	59.000	5.23	9.21	6.45
59.251	5.34	6.36	7.26	59.249	5.16	9.21	6.37
59.500	5.34	6.36	7.18	59.499	5.16	9.12	6.35
59.750	5.30	6.30	7.12	59.750	5.11	9.12	6.35
60.000	5.30	6.30	7.12	59.999	5.11	9.02	6.25
60.251	5.31	6.24	7.07	60.249	5.12	9.02	6.25
60.500	5.31	6.20	7.07	60.499	5.12	8.92	6.16
60.750	5.28	6.20	6.96	60.750	5.06	8.91	6.16
61.001	5.25	6.09	6.91	60.999	5.03	8.91	6.09
61.250	5.25	6.09	6.91	61.249	5.03	8.83	6.03
61.500	5.23	6.06	6.83	61.500	5.02	8.83	6.03
61.750	5.23	6.06	6.83	61.749	5.02	8.73	5.92
62.001	5.16	6.01	6.79	61.999	4.96	8.72	5.92
62.250	5.16	5.96	6.79	62.249	4.93	8.72	5.79
62.500	5.16	5.96	6.71	62.500	4.93	8.64	5.71
62.751	5.10	5.91	6.65	62.749	4.89	8.64	5.71
63.000	5.10	5.91	6.65	62.999	4.89	8.54	5.62
63.250	5.07	5.86	6.60	63.250	4.87	8.54	5.62
63.500	5.07	5.84	6.60	63.499	4.87	8.46	5.57
63.751	5.04	5.84	6.56	63.749	4.82	8.43	5.57
64.000	5.02	5.78	6.56	63.999	4.79	8.43	5.50
64.250	5.02	5.78	6.45	64.250	4.79	8.37	5.39
64.501	5.03	5.74	6.40	64.499	4.78	8.37	5.39
64.750	5.03	5.74	6.40	64.749	4.78	8.27	5.36
65.000	4.96	5.72	6.35	65.000	4.73	8.25	5.36
65.250	4.96	5.64	6.35	65.249	4.69	8.25	5.32
65.501	4.93	5.64	6.26	65.499	4.69	8.17	5.32
65.750	4.90	5.60	6.22	65.749	4.65	8.17	5.23
66.000	4.90	5.60	6.22	66.000	4.65	8.11	5.17
66.251	4.85	5.56	6.17	66.249	4.61	8.11	5.17
66.500	4.85	5.51	6.17	66.499	4.61	8.02	5.11
66.750	4.83	5.51	6.13	66.750	4.60	8.01	5.11
67.000	4.79	5.49	6.13	66.999	4.58	8.01	5.08
67.251	4.79	5.49	6.05	67.249	4.58	7.94	5.03
67.500	4.80	5.40	5.99	67.500	4.55	7.94	5.03
67.750	4.80	5.40	5.99	67.749	4.55	7.85	4.95
68.001	4.76	5.38	5.94	67.999	4.52	7.85	4.95
68.250	4.76	5.34	5.94	68.249	4.46	7.76	4.90

68.500	4.73	5.34	5.86	68.500	4.46	7.75	4.90
68.750	4.68	5.32	5.79	68.749	4.46	7.75	4.86
69.001	4.68	5.32	5.79	68.999	4.46	7.68	4.80
69.250	4.69	5.27	5.75	69.250	4.43	7.68	4.80
69.500	4.69	5.27	5.75	69.499	4.43	7.59	4.74
69.751	4.65	5.21	5.68	69.749	4.39	7.59	4.74
70.000	4.61	5.18	5.68	69.999	4.36	7.59	4.69
70.250	4.61	5.18	5.63	70.250	4.36	7.51	4.65
70.501	4.60	5.13	5.58	70.499	4.32	7.51	4.65
70.750	4.60	5.13	5.58	70.749	4.32	7.42	4.55
71.000	4.57	5.11	5.52	71.000	4.29	7.42	4.55
71.250	4.57	5.07	5.52	71.249	4.29	7.34	4.49
71.501	4.53	5.07	5.48	71.499	4.27	7.34	4.49
71.750	4.52	5.03	5.38	71.749	4.23	7.34	4.46
72.000	4.52	5.03	5.38	72.000	4.23	7.29	4.42
72.251	4.49	4.97	5.37	72.249	4.22	7.29	4.42
72.500	4.49	4.97	5.37	72.499	4.22	7.20	4.38
72.750	4.39	4.99	5.29	72.750	4.20	7.18	4.38
73.000	4.39	4.92	5.29	72.999	4.14	7.18	4.32
73.250	4.40	4.92	5.23	73.249	4.14	7.13	4.29
73.500	4.40	4.86	5.18	73.499	4.14	7.13	4.29
73.750	4.40	4.86	5.18	73.750	4.14	7.05	4.23
74.001	4.31	4.84	5.16	73.999	4.10	7.05	4.23
74.250	4.31	4.81	5.16	74.249	4.10	6.97	4.20
74.500	4.31	4.81	5.11	74.500	4.07	6.96	4.20
74.751	4.28	4.77	5.11	74.749	4.08	6.96	4.12
75.000	4.28	4.77	5.03	74.999	4.08	6.91	4.11
75.250	4.25	4.73	5.00	75.249	4.02	6.91	4.11
75.500	4.25	4.73	5.00	75.500	4.02	6.83	4.06
75.751	4.24	4.66	4.95	75.749	4.01	6.82	4.06
76.000	4.24	4.64	4.95	75.999	3.97	6.82	4.00
76.250	4.21	4.64	4.91	76.250	3.97	6.76	4.00
76.501	4.15	4.63	4.87	76.499	3.98	6.76	3.97
76.750	4.15	4.63	4.87	76.749	3.98	6.70	3.89
77.000	4.15	4.60	4.80	76.999	3.91	6.70	3.89
77.250	4.15	4.54	4.80	77.250	3.91	6.62	3.85
77.501	4.08	4.54	4.77	77.499	3.93	6.62	3.85
77.750	4.08	4.50	4.77	77.749	3.89	6.62	3.84
78.000	4.08	4.50	4.71	78.000	3.89	6.56	3.76
78.251	4.04	4.48	4.69	78.249	3.88	6.56	3.76
78.500	4.04	4.48	4.69	78.499	3.88	6.49	3.74
78.750	4.01	4.43	4.66	78.749	3.83	6.49	3.74
79.000	4.01	4.38	4.66	79.000	3.80	6.42	3.68
79.250	4.00	4.38	4.61	79.249	3.80	6.42	3.68
79.500	3.97	4.37	4.53	79.499	3.78	6.42	3.67
79.750	3.97	4.37	4.53	79.750	3.78	6.35	3.64
80.001	3.98	4.31	4.52	79.999	3.75	6.35	3.64
80.250	3.98	4.31	4.52	80.249	3.75	6.29	3.56
80.500	3.94	4.28	4.47	80.499	3.74	6.28	3.56
80.751	3.90	4.27	4.47	80.750	3.70	6.28	3.51
81.000	3.90	4.27	4.43	80.999	3.70	6.23	3.46
81.250	3.89	4.23	4.39	81.249	3.63	6.23	3.46
81.500	3.89	4.23	4.39	81.500	3.63	6.17	3.44
81.751	3.82	4.16	4.33	81.749	3.60	6.17	3.44
82.000	3.82	4.16	4.33	81.999	3.60	6.12	3.37
82.250	3.86	4.16	4.31	82.249	3.58	6.11	3.37

82.501	3.81	4.17	4.24	82.500	3.53	6.11	3.36
82.750	3.81	4.17	4.24	82.749	3.53	6.06	3.30
83.000	3.76	4.12	4.20	82.999	3.54	6.06	3.30
83.250	3.76	4.12	4.20	83.250	3.54	5.98	3.29
83.501	3.79	4.07	4.18	83.499	3.49	5.98	3.29
83.750	3.79	4.05	4.18	83.749	3.46	5.98	3.24
84.000	3.75	4.05	4.10	83.999	3.46	5.92	3.21
84.251	3.70	4.01	4.08	84.250	3.49	5.92	3.21
84.500	3.70	4.01	4.08	84.499	3.49	5.86	3.21
84.750	3.64	3.96	4.00	84.749	3.44	5.86	3.21
85.000	3.64	3.97	4.00	85.000	3.44	5.80	3.15
85.250	3.64	3.97	3.97	85.249	3.42	5.79	3.15
85.500	3.63	3.92	3.97	85.499	3.42	5.79	3.13
85.750	3.63	3.92	3.93	85.749	3.42	5.75	3.08
86.001	3.57	3.93	3.88	86.000	3.38	5.75	3.08
86.250	3.57	3.93	3.88	86.249	3.38	5.69	3.05
86.500	3.56	3.89	3.85	86.499	3.37	5.67	3.05
86.751	3.56	3.84	3.85	86.750	3.32	5.67	3.01
87.000	3.53	3.84	3.83	86.999	3.32	5.64	3.01
87.250	3.53	3.79	3.77	87.249	3.33	5.64	2.99
87.500	3.53	3.79	3.77	87.499	3.33	5.58	2.92
87.751	3.48	3.81	3.75	87.750	3.30	5.58	2.92
88.000	3.48	3.76	3.75	87.999	3.30	5.53	2.92
88.250	3.47	3.76	3.68	88.249	3.29	5.53	2.92
88.501	3.43	3.74	3.68	88.500	3.26	5.53	2.87
88.750	3.43	3.74	3.65	88.749	3.26	5.47	2.85
89.000	3.41	3.71	3.63	88.999	3.25	5.47	2.85
89.250	3.41	3.71	3.63	89.249	3.25	5.42	2.79
89.501	3.41	3.68	3.61	89.499	3.21	5.42	2.79
89.750	3.41	3.65	3.61	89.749	3.18	5.36	2.77
90.000	3.39	3.65	3.56	89.999	3.18	5.35	2.77
90.251	3.37	3.62	3.50	90.250	3.18	5.35	2.73
90.500	3.37	3.62	3.50	90.499	3.18	5.31	2.73
90.750	3.32	3.58	3.48	90.749	3.14	5.31	2.73
91.000	3.32	3.58	3.48	90.999	3.14	5.25	2.66
91.251	3.33	3.55	3.43	91.250	3.14	5.24	2.66
91.500	3.31	3.54	3.43	91.499	3.12	5.24	2.65
91.750	3.31	3.54	3.37	91.749	3.12	5.21	2.61
92.001	3.27	3.52	3.38	92.000	3.08	5.21	2.61
92.250	3.27	3.52	3.38	92.249	3.08	5.15	2.57
92.500	3.27	3.49	3.35	92.499	3.06	5.15	2.57
92.750	3.27	3.46	3.35	92.749	3.06	5.12	2.54
93.000	3.25	3.46	3.31	93.000	3.06	5.11	2.54
93.250	3.20	3.46	3.27	93.249	3.04	5.11	2.49
93.500	3.20	3.46	3.27	93.499	3.04	5.07	2.47
93.751	3.18	3.41	3.27	93.750	3.02	5.07	2.47
94.000	3.18	3.41	3.27	93.999	3.02	5.01	2.46
94.250	3.18	3.38	3.24	94.249	2.96	5.00	2.46
94.501	3.18	3.37	3.24	94.499	2.99	5.00	2.42
94.750	3.19	3.37	3.21	94.750	2.99	4.96	2.41
95.000	3.14	3.38	3.14	94.999	2.97	4.96	2.41
95.250	3.14	3.38	3.14	95.249	2.97	4.92	2.35
95.501	3.13	3.31	3.12	95.500	2.97	4.92	2.35
95.750	3.13	3.30	3.12	95.749	2.97	4.88	2.32
96.000	3.07	3.30	3.09	95.999	2.91	4.87	2.32
96.251	3.03	3.31	3.09	96.249	2.95	4.87	2.29

96.500	3.03	3.31	3.08	96.500	2.95	4.84	2.28
96.750	3.02	3.24	3.07	96.749	2.90	4.84	2.28
97.000	3.02	3.24	3.07	96.999	2.90	4.79	2.22
97.251	3.02	3.23	3.02	97.250	2.89	4.79	2.22
97.500	3.02	3.20	3.02	97.499	2.88	4.79	2.19
97.750	2.98	3.20	2.99	97.749	2.88	4.73	2.19
98.001	2.96	3.19	2.97	97.999	2.86	4.73	2.18
98.250	2.96	3.19	2.97	98.250	2.86	4.70	2.15
98.500	2.94	3.16	2.92	98.499	2.82	4.70	2.15
98.750	2.94	3.15	2.92	98.749	2.82	4.67	2.11
99.001	2.94	3.15	2.88	99.000	2.79	4.66	2.11
99.250	2.90	3.12	2.88	99.249	2.77	4.66	2.08
99.500	2.90	3.12	2.84	99.499	2.77	4.63	2.02
99.751	2.90	3.09	2.83	99.749	2.77	4.63	2.02
100.000	2.90	3.09	2.83	100.000	2.77	4.59	2.03
100.250	2.86	3.09	2.81	100.249	2.77	4.59	2.03
100.500	2.86	3.02	2.81	100.499	2.74	4.54	1.97
100.750	2.88	3.02	2.78	100.750	2.74	4.53	1.97
101.000	2.84	3.05	2.77	100.999	2.71	4.53	1.95
101.250	2.84	3.05	2.77	101.249	2.71	4.51	1.93
101.501	2.85	3.03	2.74	101.499	2.68	4.51	1.93
101.750	2.85	3.03	2.74	101.749	2.68	4.45	1.90
102.000	2.81	2.99	2.74	101.999	2.70	4.44	1.90
102.250	2.80	2.98	2.74	102.249	2.65	4.44	1.88
102.501	2.80	2.98	2.67	102.500	2.65	4.42	1.85
102.750	2.77	2.97	2.66	102.749	2.66	4.42	1.85
103.000	2.77	2.97	2.66	102.999	2.66	4.36	1.79
103.251	2.78	2.95	2.64	103.250	2.68	4.36	1.79
103.500	2.78	2.90	2.64	103.499	2.68	4.33	1.81
103.750	2.74	2.90	2.62	103.749	2.63	4.32	1.81
104.000	2.71	2.88	2.56	103.999	2.60	4.32	1.78
104.250	2.71	2.88	2.56	104.250	2.60	4.30	1.76
104.500	2.69	2.88	2.52	104.499	2.59	4.30	1.76
104.750	2.69	2.88	2.52	104.749	2.59	4.26	1.72
105.001	2.66	2.82	2.50	105.000	2.56	4.25	1.72
105.250	2.66	2.83	2.50	105.249	2.57	4.25	1.68
105.500	2.67	2.83	2.49	105.499	2.57	4.23	1.66
105.751	2.64	2.82	2.45	105.749	2.55	4.23	1.66
106.000	2.64	2.82	2.45	106.000	2.55	4.19	1.63
106.250	2.65	2.77	2.40	106.249	2.54	4.19	1.63
106.500	2.65	2.82	2.40	106.499	2.54	4.15	1.62
106.751	2.60	2.82	2.39	106.750	2.52	4.14	1.62
107.000	2.58	2.73	2.39	106.999	2.52	4.14	1.61
107.250	2.58	2.73	2.39	107.249	2.52	4.11	1.59
107.501	2.59	2.77	2.35	107.499	2.52	4.11	1.59
107.750	2.59	2.77	2.35	107.750	2.52	4.08	1.54
108.000	2.55	2.73	2.38	107.999	2.46	4.07	1.54
108.250	2.55	2.70	2.38	108.249	2.44	4.07	1.55
108.500	2.56	2.70	2.29	108.500	2.44	4.03	1.55
108.750	2.55	2.70	2.29	108.749	2.45	4.03	1.50
109.000	2.55	2.70	2.29	108.999	2.45	4.01	1.44
109.251	2.54	2.69	2.27	109.249	2.42	4.01	1.44
109.500	2.54	2.63	2.27	109.500	2.42	3.96	1.47
109.750	2.49	2.63	2.24	109.749	2.42	3.96	1.47
110.001	2.47	2.62	2.24	109.999	2.40	3.96	1.41
110.250	2.47	2.62	2.25	110.249	2.40	3.93	1.40

110.500	2.44	2.60	2.21	110.499	2.36	3.93	1.40
110.750	2.44	2.60	2.21	110.749	2.36	3.90	1.39
111.001	2.45	2.62	2.19	110.999	2.35	3.90	1.39
111.250	2.45	2.56	2.19	111.250	2.38	3.87	1.33
111.500	2.42	2.56	2.18	111.499	2.38	3.86	1.33
111.751	2.42	2.54	2.17	111.749	2.36	3.86	1.33
112.000	2.42	2.54	2.17	112.000	2.36	3.83	1.29
112.250	2.40	2.54	2.12	112.249	2.33	3.83	1.29
112.500	2.40	2.54	2.12	112.499	2.33	3.78	1.24
112.751	2.38	2.54	2.12	112.749	2.30	3.78	1.24
113.000	2.39	2.51	2.12	113.000	2.32	3.78	1.22
113.250	2.39	2.51	2.11	113.249	2.32	3.78	1.23
113.500	2.35	2.51	2.08	113.499	2.30	3.78	1.23
113.750	2.35	2.51	2.08	113.750	2.30	3.74	1.20
114.000	2.31	2.46	2.05	113.999	2.30	3.74	1.20
114.250	2.31	2.47	2.05	114.249	2.30	3.71	1.17
114.501	2.33	2.47	2.01	114.499	2.27	3.71	1.17
114.750	2.30	2.44	1.99	114.750	2.23	3.71	1.16
115.000	2.30	2.44	1.99	114.999	2.23	3.68	1.13
115.251	2.30	2.43	1.99	115.249	2.22	3.68	1.13
115.500	2.30	2.43	1.99	115.500	2.22	3.64	1.09
115.750	2.31	2.42	2.02	115.749	2.23	3.64	1.09
116.000	2.31	2.37	2.02	115.999	2.19	3.64	1.07
116.251	2.28	2.37	1.94	116.249	2.19	3.62	1.02
116.500	2.29	2.37	1.94	116.499	2.21	3.62	1.02
116.750	2.29	2.37	1.94	116.749	2.21	3.60	1.01
117.001	2.24	2.35	1.94	116.999	2.21	3.60	1.01
117.250	2.24	2.37	1.94	117.250	2.21	3.57	0.97
117.500	2.24	2.37	1.88	117.499	2.17	3.56	0.97
117.750	2.22	2.31	1.88	117.749	2.17	3.56	0.95
118.001	2.22	2.31	1.88	117.999	2.17	3.55	0.93
118.250	2.24	2.32	1.85	118.250	2.19	3.55	0.93
118.500	2.24	2.32	1.85	118.499	2.19	3.50	0.91
118.751	2.23	2.31	1.86	118.749	2.17	3.49	0.91
119.000	2.23	2.26	1.86	119.000	2.14	3.49	0.88
119.250	2.22	2.26	1.83	119.249	2.14	3.48	0.88
119.500	2.19	2.28	1.77	119.499	2.15	3.48	0.86
119.751	2.19	2.28	1.77	119.749	2.15	3.46	0.86
120.000	2.21	2.23	1.80	120.000	2.09	3.46	0.86
120.250	2.21	2.21	1.80	120.249	2.09	3.42	0.84
120.501	2.17	2.21	1.78	120.499	2.09	3.41	0.84
120.750	2.20	2.21	1.78	120.750	2.09	3.41	0.82
121.000	2.20	2.21	1.76	120.999	2.09	3.40	0.80
121.250	2.11	2.17	1.72	121.249	2.05	3.40	0.80
121.501	2.11	2.17	1.72	121.499	2.05	3.37	0.77
121.750	2.15	2.17	1.71	121.749	2.06	3.37	0.77
122.000	2.15	2.20	1.71	121.999	2.03		
122.251	2.15	2.20	1.69	122.249	2.03		
122.500	2.10	2.17	1.65	122.500	2.06		
122.750	2.10	2.17	1.65	122.749	2.06		
123.000	2.13	2.14	1.66	122.999	2.03		
123.251	2.13	2.14	1.66	123.249	2.03		
123.500	2.09	2.15	1.67				
123.750	2.05	2.10	1.67				
124.001	2.05	2.10	1.61				
124.250	2.10	2.10	1.64				

124.500	2.10	2.10	1.64
124.750	2.07	2.07	1.60
125.001	2.07	2.09	1.60
125.250	2.09	2.09	1.55
125.500	2.02	2.08	1.54
125.751	2.02	2.08	1.54
126.000	2.03	2.06	1.57
126.250	2.03	2.06	1.57
126.500	2.05	2.03	1.50
126.751	2.05	1.99	1.50
127.000	2.00	1.99	1.48
127.250	2.04	2.01	1.50
127.501	2.04	2.01	1.50
127.750	1.98	1.97	1.47
128.000	1.98	2.00	1.47
128.250	2.00	2.00	1.45
128.501	2.00	1.96	1.45
128.750	2.00	1.96	1.42
129.000	1.98	1.93	1.44
129.251	1.98	1.93	1.44
129.500	1.97	1.94	1.40
129.750	1.97		1.40
130.001	1.94		1.39
130.250	1.96		1.37
130.500	1.96		1.37
130.750	1.97		1.34
131.001	1.97		1.34
131.250	1.90		1.35
131.500	1.94		1.35
131.751	1.94		1.32
132.000	1.90		1.33
132.250	1.90		1.33
132.500	1.91		1.30
132.751	1.91		1.30
133.000	1.84		1.28
133.250	1.89		1.27
133.501	1.89		1.27
133.750	1.86		1.23
134.000	1.86		1.23
134.250	1.88		1.23
134.501	1.84		1.23
134.750	1.84		1.22
135.000	1.82		1.20
135.251	1.82		1.20
135.500	1.83		1.20
135.750	1.83		1.20
136.000	1.80		1.17
136.250	1.83		1.16
136.500	1.83		1.16
136.750	1.80		1.17
137.001	1.80		1.17
137.250	1.79		1.15
137.500	1.79		1.15
137.750	1.79		1.15
138.001	1.78		1.12
138.250	1.78		1.12

138.500	1.77	1.09
138.751	1.77	1.09
139.000	1.75	1.10
139.250	1.72	1.10
139.500	1.72	1.07
139.750	1.73	1.04
140.000	1.73	1.04
140.250	1.74	1.03
140.501	1.74	1.03
140.750	1.70	1.03
141.000	1.75	1.05
141.250	1.75	1.05
141.500	1.69	0.99
141.750	1.69	0.99
142.000	1.72	0.97
142.251	1.69	0.97
142.500	1.69	0.98
142.750	1.66	0.95
143.000	1.66	0.95
143.251	1.67	0.96
143.500	1.67	0.96
143.750	1.64	0.98
144.001	1.68	0.91
144.250	1.68	0.91
144.500	1.66	0.92
144.750	1.66	0.92
145.000	1.68	0.90
145.250	1.69	0.90
145.500	1.69	0.86
145.751	1.63	0.86
146.000	1.63	0.86
146.250	1.62	0.85
146.500	1.62	0.85
146.750	1.62	0.84
147.000	1.62	0.83
147.250	1.62	0.83
147.501	1.59	0.80
147.750	1.59	0.80
148.000	1.61	0.83
148.251	1.61	0.83
148.500	1.60	0.77
148.751		0.78
149.001		0.78
149.251		0.75
149.501		0.75
149.751		0.72
150.001		0.72
150.251		0.70
150.501		0.71
150.751		0.71
151.001		0.72
151.252		0.72
151.502		0.70
151.752		0.65
152.002		0.65
152.252		0.67

152.502	0.67
152.752	0.65
153.002	0.65
153.252	0.65
153.502	0.69
153.753	0.69
154.003	0.64
154.253	0.64
154.503	0.62
154.753	0.63
155.003	0.63
155.253	0.63
155.503	0.63
155.753	0.58
156.003	0.58
156.254	0.53
156.504	0.58
