# Supplementary material for: Impact of drill bit wear on temperature increase in dental implant osteotomy: an in vitro study
Source: PLoS One. 2025 Mar 19;20(3):e0319492. doi: 10.1371/journal.pone.0319492 (PMC11922234; doi:10.1371/journal.pone.0319492)
Supplement: S2 Table — The data presented in S2 Table were used to generate the hole profiles in Fig 6. The table reports only the mean values calculated from three different measurements. For more information about measurement deviations, please refer to S5 Table. (PDF) [file pone.0319492.s002.pdf]

Protocol	AT
Hole nr.	10
	**Mean Values**
Hole depth (mm)	Radius (mm)
-13.960	0.218
-13.950	0.252
-13.940	0.281
-13.930	0.313
-13.920	0.344
-13.910	0.376
-13.900	0.410
-13.890	0.445
-13.880	0.477
-13.870	0.505
-13.860	0.532
-13.850	0.558
-13.840	0.585
-13.830	0.611
-13.820	0.638
-13.810	0.666
-13.800	0.696
-13.790	0.725
-13.780	0.755
-13.770	0.782
-13.760	0.804
-13.750	0.826
-13.740	0.848
-13.730	0.870
-13.720	0.889
-13.710	0.901
-13.700	0.912
-13.690	0.922
-13.680	0.933
-13.670	0.943
-13.660	0.953
-13.650	0.964
-13.640	0.974
-13.630	0.984
-13.620	0.994
-13.610	1.004
-13.600	1.014
-13.590	1.025
-13.580	1.035
-13.570	1.045
-13.560	1.055
-13.550	1.065
-13.540	1.075
-13.530	1.084
-13.520	1.094
-13.510	1.103
-13.500	1.113
-13.490	1.122
-13.480	1.132
-13.470	1.141
-13.460	1.151

Protocol	PT
Hole nr.	10
	**Mean Values**
Hole depth (mm)	Radius (mm)
-13.990	0.322
-13.980	0.350
-13.970	0.374
-13.960	0.398
-13.950	0.420
-13.940	0.442
-13.930	0.464
-13.920	0.485
-13.910	0.506
-13.900	0.527
-13.890	0.548
-13.880	0.569
-13.870	0.589
-13.860	0.610
-13.850	0.629
-13.840	0.648
-13.830	0.667
-13.820	0.687
-13.810	0.708
-13.800	0.730
-13.790	0.752
-13.780	0.774
-13.770	0.795
-13.760	0.817
-13.750	0.838
-13.740	0.860
-13.730	0.881
-13.720	0.902
-13.710	0.921
-13.700	0.939
-13.690	0.957
-13.680	0.973
-13.670	0.989
-13.660	1.005
-13.650	1.022
-13.640	1.037
-13.630	1.053
-13.620	1.069
-13.610	1.084
-13.600	1.100
-13.590	1.116
-13.580	1.131
-13.570	1.145
-13.560	1.159
-13.550	1.172
-13.540	1.186
-13.530	1.198
-13.520	1.209
-13.510	1.221
-13.500	1.232
-13.490	1.243

-13.450	1.160	-13.480	1.254
-13.440	1.170	-13.470	1.265
-13.430	1.179	-13.460	1.275
-13.420	1.189	-13.450	1.285
-13.410	1.198	-13.440	1.295
-13.400	1.208	-13.430	1.305
-13.390	1.217	-13.420	1.315
-13.380	1.227	-13.410	1.324
-13.370	1.236	-13.400	1.332
-13.360	1.246	-13.390	1.341
-13.350	1.255	-13.380	1.348
-13.340	1.265	-13.370	1.355
-13.330	1.274	-13.360	1.362
-13.320	1.284	-13.350	1.369
-13.310	1.293	-13.340	1.376
-13.300	1.302	-13.330	1.383
-13.290	1.312	-13.320	1.390
-13.280	1.321	-13.310	1.397
-13.270	1.331	-13.300	1.404
-13.260	1.340	-13.290	1.410
-13.250	1.350	-13.280	1.417
-13.240	1.359	-13.270	1.424
-13.230	1.369	-13.260	1.431
-13.220	1.378	-13.250	1.437
-13.210	1.388	-13.240	1.444
-13.200	1.397	-13.230	1.450
-13.190	1.407	-13.220	1.456
-13.180	1.416	-13.210	1.463
-13.170	1.424	-13.200	1.469
-13.160	1.431	-13.190	1.475
-13.150	1.438	-13.180	1.481
-13.140	1.445	-13.170	1.487
-13.130	1.451	-13.160	1.494
-13.120	1.456	-13.150	1.500
-13.110	1.460	-13.140	1.506
-13.100	1.465	-13.130	1.512
-13.090	1.469	-13.120	1.519
-13.080	1.474	-13.110	1.525
-13.070	1.478	-13.100	1.531
-13.060	1.482	-13.090	1.537
-13.050	1.486	-13.080	1.544
-13.040	1.490	-13.070	1.550
-13.030	1.494	-13.060	1.556
-13.020	1.498	-13.050	1.562
-13.010	1.502	-13.040	1.568
-13.000	1.506	-13.030	1.575
-12.990	1.511	-13.020	1.581
-12.980	1.515	-13.010	1.586
-12.970	1.519	-13.000	1.592
-12.960	1.523	-12.990	1.597
-12.950	1.527	-12.980	1.602
-12.940	1.530	-12.970	1.607
-12.930	1.534	-12.960	1.612
-12.920	1.537	-12.950	1.618
-12.910	1.539	-12.940	1.623
-12.900	1.541	-12.930	1.628

-12.890	1.543	-12.920	1.633
-12.880	1.545	-12.910	1.639
-12.870	1.546	-12.900	1.644
-12.860	1.547	-12.890	1.649
-12.850	1.548	-12.880	1.654
-12.840	1.550	-12.870	1.659
-12.830	1.551	-12.860	1.665
-12.820	1.552	-12.850	1.670
-12.810	1.554	-12.840	1.675
-12.800	1.555	-12.830	1.680
-12.790	1.557	-12.820	1.685
-12.780	1.558	-12.810	1.690
-12.770	1.559	-12.800	1.694
-12.760	1.561	-12.790	1.699
-12.750	1.562	-12.780	1.704
-12.740	1.564	-12.770	1.708
-12.730	1.565	-12.760	1.713
-12.720	1.566	-12.750	1.718
-12.710	1.568	-12.740	1.722
-12.700	1.569	-12.730	1.726
-12.690	1.570	-12.720	1.730
-12.680	1.571	-12.710	1.733
-12.670	1.572	-12.700	1.737
-12.660	1.572	-12.690	1.740
-12.650	1.572	-12.680	1.742
-12.640	1.572	-12.670	1.744
-12.630	1.572	-12.660	1.747
-12.620	1.572	-12.650	1.749
-12.610	1.572	-12.640	1.751
-12.600	1.571	-12.630	1.754
-12.590	1.571	-12.620	1.756
-12.580	1.571	-12.610	1.758
-12.570	1.571	-12.600	1.760
-12.560	1.570	-12.590	1.761
-12.550	1.570	-12.580	1.763
-12.540	1.570	-12.570	1.765
-12.530	1.569	-12.560	1.767
-12.520	1.569	-12.550	1.769
-12.510	1.569	-12.540	1.770
-12.500	1.569	-12.530	1.771
-12.490	1.569	-12.520	1.772
-12.480	1.570	-12.510	1.773
-12.470	1.569	-12.500	1.774
-12.460	1.569	-12.490	1.774
-12.450	1.568	-12.480	1.775
-12.440	1.568	-12.470	1.775
-12.430	1.568	-12.460	1.776
-12.420	1.568	-12.450	1.776
-12.410	1.567	-12.440	1.776
-12.400	1.567	-12.430	1.777
-12.390	1.567	-12.420	1.777
-12.380	1.567	-12.410	1.777
-12.370	1.566	-12.400	1.778
-12.360	1.566	-12.390	1.778
-12.350	1.566	-12.380	1.778
-12.340	1.565	-12.370	1.779

-12.330	1.565	-12.360	1.779
-12.320	1.565	-12.350	1.779
-12.310	1.564	-12.340	1.779
-12.300	1.564	-12.330	1.778
-12.290	1.563	-12.320	1.778
-12.280	1.563	-12.310	1.778
-12.270	1.563	-12.300	1.778
-12.260	1.563	-12.290	1.778
-12.250	1.562	-12.280	1.778
-12.240	1.562	-12.270	1.777
-12.230	1.562	-12.260	1.777
-12.220	1.562	-12.250	1.777
-12.210	1.562	-12.240	1.776
-12.200	1.562	-12.230	1.776
-12.190	1.562	-12.220	1.775
-12.180	1.562	-12.210	1.775
-12.170	1.562	-12.200	1.775
-12.160	1.562	-12.190	1.775
-12.150	1.562	-12.180	1.774
-12.140	1.562	-12.170	1.774
-12.130	1.562	-12.160	1.774
-12.120	1.562	-12.150	1.774
-12.110	1.562	-12.140	1.774
-12.100	1.562	-12.130	1.774
-12.090	1.562	-12.120	1.775
-12.080	1.563	-12.110	1.775
-12.070	1.563	-12.100	1.775
-12.060	1.563	-12.090	1.776
-12.050	1.563	-12.080	1.776
-12.040	1.564	-12.070	1.776
-12.030	1.564	-12.060	1.777
-12.020	1.565	-12.050	1.777
-12.010	1.565	-12.040	1.777
-12.000	1.566	-12.030	1.778
-11.990	1.566	-12.020	1.778
-11.980	1.566	-12.010	1.778
-11.970	1.567	-12.000	1.779
-11.960	1.567	-11.990	1.779
-11.950	1.567	-11.980	1.779
-11.940	1.568	-11.970	1.780
-11.930	1.568	-11.960	1.781
-11.920	1.568	-11.950	1.781
-11.910	1.568	-11.940	1.782
-11.900	1.568	-11.930	1.782
-11.890	1.568	-11.920	1.783
-11.880	1.567	-11.910	1.783
-11.870	1.567	-11.900	1.784
-11.860	1.567	-11.890	1.785
-11.850	1.567	-11.880	1.785
-11.840	1.566	-11.870	1.786
-11.830	1.566	-11.860	1.786
-11.820	1.566	-11.850	1.787
-11.810	1.565	-11.840	1.787
-11.800	1.565	-11.830	1.787
-11.790	1.565	-11.820	1.787
-11.780	1.565	-11.810	1.788

-11.770	1.565	-11.800	1.788
-11.760	1.565	-11.790	1.788
-11.750	1.564	-11.780	1.788
-11.740	1.564	-11.770	1.788
-11.730	1.564	-11.760	1.788
-11.720	1.564	-11.750	1.787
-11.710	1.564	-11.740	1.787
-11.700	1.564	-11.730	1.786
-11.690	1.564	-11.720	1.786
-11.680	1.564	-11.710	1.785
-11.670	1.564	-11.700	1.785
-11.660	1.564	-11.690	1.784
-11.650	1.565	-11.680	1.784
-11.640	1.565	-11.670	1.783
-11.630	1.565	-11.660	1.783
-11.620	1.565	-11.650	1.783
-11.610	1.565	-11.640	1.783
-11.600	1.565	-11.630	1.782
-11.590	1.565	-11.620	1.782
-11.580	1.564	-11.610	1.782
-11.570	1.564	-11.600	1.782
-11.560	1.564	-11.590	1.781
-11.550	1.564	-11.580	1.781
-11.540	1.564	-11.570	1.781
-11.530	1.564	-11.560	1.781
-11.520	1.563	-11.550	1.781
-11.510	1.563	-11.540	1.781
-11.500	1.563	-11.530	1.781
-11.490	1.563	-11.520	1.781
-11.480	1.563	-11.510	1.781
-11.470	1.563	-11.500	1.781
-11.460	1.564	-11.490	1.780
-11.450	1.564	-11.480	1.780
-11.440	1.564	-11.470	1.780
-11.430	1.565	-11.460	1.780
-11.420	1.565	-11.450	1.779
-11.410	1.565	-11.440	1.779
-11.400	1.565	-11.430	1.780
-11.390	1.565	-11.420	1.779
-11.380	1.566	-11.410	1.779
-11.370	1.566	-11.400	1.779
-11.360	1.567	-11.390	1.779
-11.350	1.569	-11.380	1.778
-11.340	1.572	-11.370	1.779
-11.330	1.575	-11.360	1.779
-11.320	1.578	-11.350	1.779
-11.310	1.581	-11.340	1.780
-11.300	1.584	-11.330	1.780
-11.290	1.587	-11.320	1.781
-11.280	1.590	-11.310	1.781
-11.270	1.593	-11.300	1.781
-11.260	1.596	-11.290	1.782
-11.250	1.599	-11.280	1.782
-11.240	1.602	-11.270	1.782
-11.230	1.605	-11.260	1.783
-11.220	1.610	-11.250	1.783

-11.210	1.614	-11.240	1.784
-11.200	1.618	-11.230	1.784
-11.190	1.623	-11.220	1.784
-11.180	1.627	-11.210	1.785
-11.170	1.631	-11.200	1.785
-11.160	1.635	-11.190	1.785
-11.150	1.640	-11.180	1.786
-11.140	1.644	-11.170	1.786
-11.130	1.648	-11.160	1.786
-11.120	1.652	-11.150	1.786
-11.110	1.657	-11.140	1.786
-11.100	1.661	-11.130	1.786
-11.090	1.665	-11.120	1.786
-11.080	1.670	-11.110	1.786
-11.070	1.674	-11.100	1.786
-11.060	1.678	-11.090	1.786
-11.050	1.682	-11.080	1.785
-11.040	1.687	-11.070	1.785
-11.030	1.691	-11.060	1.785
-11.020	1.695	-11.050	1.785
-11.010	1.699	-11.040	1.784
-11.000	1.704	-11.030	1.784
-10.990	1.708	-11.020	1.784
-10.980	1.712	-11.010	1.783
-10.970	1.716	-11.000	1.783
-10.960	1.721	-10.990	1.782
-10.950	1.725	-10.980	1.782
-10.940	1.729	-10.970	1.781
-10.930	1.734	-10.960	1.781
-10.920	1.738	-10.950	1.781
-10.910	1.742	-10.940	1.781
-10.900	1.746	-10.930	1.780
-10.890	1.750	-10.920	1.780
-10.880	1.753	-10.910	1.780
-10.870	1.757	-10.900	1.780
-10.860	1.761	-10.890	1.780
-10.850	1.765	-10.880	1.780
-10.840	1.769	-10.870	1.780
-10.830	1.773	-10.860	1.781
-10.820	1.777	-10.850	1.781
-10.810	1.781	-10.840	1.781
-10.800	1.785	-10.830	1.781
-10.790	1.788	-10.820	1.781
-10.780	1.792	-10.810	1.781
-10.770	1.796	-10.800	1.781
-10.760	1.799	-10.790	1.781
-10.750	1.803	-10.780	1.781
-10.740	1.806	-10.770	1.780
-10.730	1.810	-10.760	1.780
-10.720	1.813	-10.750	1.780
-10.710	1.817	-10.740	1.780
-10.700	1.821	-10.730	1.780
-10.690	1.824	-10.720	1.781
-10.680	1.828	-10.710	1.781
-10.670	1.831	-10.700	1.781
-10.660	1.835	-10.690	1.781

-10.650	1.839	-10.680	1.781
-10.640	1.842	-10.670	1.781
-10.630	1.845	-10.660	1.781
-10.620	1.847	-10.650	1.781
-10.610	1.849	-10.640	1.781
-10.600	1.850	-10.630	1.781
-10.590	1.851	-10.620	1.781
-10.580	1.852	-10.610	1.781
-10.570	1.854	-10.600	1.781
-10.560	1.855	-10.590	1.781
-10.550	1.856	-10.580	1.781
-10.540	1.856	-10.570	1.781
-10.530	1.856	-10.560	1.781
-10.520	1.856	-10.550	1.781
-10.510	1.856	-10.540	1.781
-10.500	1.856	-10.530	1.781
-10.490	1.856	-10.520	1.781
-10.480	1.855	-10.510	1.782
-10.470	1.855	-10.500	1.782
-10.460	1.855	-10.490	1.782
-10.450	1.855	-10.480	1.782
-10.440	1.854	-10.470	1.782
-10.430	1.854	-10.460	1.783
-10.420	1.854	-10.450	1.783
-10.410	1.854	-10.440	1.783
-10.400	1.854	-10.430	1.783
-10.390	1.854	-10.420	1.783
-10.380	1.855	-10.410	1.783
-10.370	1.855	-10.400	1.783
-10.360	1.855	-10.390	1.783
-10.350	1.855	-10.380	1.783
-10.340	1.855	-10.370	1.783
-10.330	1.855	-10.360	1.783
-10.320	1.856	-10.350	1.784
-10.310	1.857	-10.340	1.784
-10.300	1.857	-10.330	1.784
-10.290	1.858	-10.320	1.784
-10.280	1.859	-10.310	1.784
-10.270	1.859	-10.300	1.785
-10.260	1.860	-10.290	1.785
-10.250	1.860	-10.280	1.785
-10.240	1.861	-10.270	1.785
-10.230	1.861	-10.260	1.786
-10.220	1.862	-10.250	1.786
-10.210	1.862	-10.240	1.786
-10.200	1.863	-10.230	1.786
-10.190	1.863	-10.220	1.786
-10.180	1.864	-10.210	1.786
-10.170	1.864	-10.200	1.787
-10.160	1.865	-10.190	1.786
-10.150	1.865	-10.180	1.786
-10.140	1.866	-10.170	1.786
-10.130	1.866	-10.160	1.786
-10.120	1.866	-10.150	1.786
-10.110	1.867	-10.140	1.786
-10.100	1.867	-10.130	1.786

-10.090	1.867	-10.120	1.786
-10.080	1.867	-10.110	1.786
-10.070	1.867	-10.100	1.786
-10.060	1.867	-10.090	1.786
-10.050	1.867	-10.080	1.786
-10.040	1.867	-10.070	1.786
-10.030	1.867	-10.060	1.786
-10.020	1.868	-10.050	1.787
-10.010	1.868	-10.040	1.787
-10.000	1.868	-10.030	1.787
-9.990	1.867	-10.020	1.788
-9.980	1.867	-10.010	1.788
-9.970	1.867	-10.000	1.788
-9.960	1.866	-9.990	1.788
-9.950	1.866	-9.980	1.789
-9.940	1.865	-9.970	1.789
-9.930	1.865	-9.960	1.789
-9.920	1.865	-9.950	1.789
-9.910	1.864	-9.940	1.789
-9.900	1.864	-9.930	1.789
-9.890	1.864	-9.920	1.789
-9.880	1.865	-9.910	1.789
-9.870	1.865	-9.900	1.789
-9.860	1.866	-9.890	1.788
-9.850	1.866	-9.880	1.788
-9.840	1.866	-9.870	1.788
-9.830	1.867	-9.860	1.787
-9.820	1.867	-9.850	1.787
-9.810	1.867	-9.840	1.787
-9.800	1.868	-9.830	1.786
-9.790	1.868	-9.820	1.787
-9.780	1.868	-9.810	1.787
-9.770	1.869	-9.800	1.787
-9.760	1.869	-9.790	1.788
-9.750	1.869	-9.780	1.788
-9.740	1.870	-9.770	1.788
-9.730	1.870	-9.760	1.789
-9.720	1.870	-9.750	1.789
-9.710	1.870	-9.740	1.789
-9.700	1.870	-9.730	1.789
-9.690	1.871	-9.720	1.789
-9.680	1.871	-9.710	1.789
-9.670	1.871	-9.700	1.789
-9.660	1.871	-9.690	1.789
-9.650	1.871	-9.680	1.789
-9.640	1.871	-9.670	1.789
-9.630	1.871	-9.660	1.789
-9.620	1.871	-9.650	1.789
-9.610	1.871	-9.640	1.789
-9.600	1.872	-9.630	1.789
-9.590	1.872	-9.620	1.790
-9.580	1.872	-9.610	1.790
-9.570	1.872	-9.600	1.790
-9.560	1.872	-9.590	1.790
-9.550	1.872	-9.580	1.790
-9.540	1.872	-9.570	1.790

-9.530	1.871	-9.560	1.790
-9.520	1.871	-9.550	1.790
-9.510	1.871	-9.540	1.790
-9.500	1.871	-9.530	1.790
-9.490	1.871	-9.520	1.790
-9.480	1.870	-9.510	1.790
-9.470	1.870	-9.500	1.790
-9.460	1.870	-9.490	1.791
-9.450	1.869	-9.480	1.791
-9.440	1.869	-9.470	1.791
-9.430	1.869	-9.460	1.792
-9.420	1.868	-9.450	1.792
-9.410	1.868	-9.440	1.792
-9.400	1.867	-9.430	1.792
-9.390	1.866	-9.420	1.792
-9.380	1.866	-9.410	1.792
-9.370	1.865	-9.400	1.792
-9.360	1.865	-9.390	1.792
-9.350	1.864	-9.380	1.793
-9.340	1.864	-9.370	1.793
-9.330	1.864	-9.360	1.792
-9.320	1.863	-9.350	1.792
-9.310	1.863	-9.340	1.792
-9.300	1.863	-9.330	1.791
-9.290	1.863	-9.320	1.791
-9.280	1.863	-9.310	1.791
-9.270	1.863	-9.300	1.791
-9.260	1.863	-9.290	1.791
-9.250	1.863	-9.280	1.790
-9.240	1.863	-9.270	1.790
-9.230	1.862	-9.260	1.790
-9.220	1.862	-9.250	1.790
-9.210	1.863	-9.240	1.790
-9.200	1.863	-9.230	1.790
-9.190	1.863	-9.220	1.790
-9.180	1.863	-9.210	1.790
-9.170	1.863	-9.200	1.790
-9.160	1.864	-9.190	1.790
-9.150	1.864	-9.180	1.790
-9.140	1.864	-9.170	1.790
-9.130	1.864	-9.160	1.790
-9.120	1.865	-9.150	1.790
-9.110	1.865	-9.140	1.791
-9.100	1.865	-9.130	1.791
-9.090	1.865	-9.120	1.791
-9.080	1.866	-9.110	1.791
-9.070	1.866	-9.100	1.792
-9.060	1.866	-9.090	1.792
-9.050	1.867	-9.080	1.793
-9.040	1.867	-9.070	1.793
-9.030	1.868	-9.060	1.794
-9.020	1.868	-9.050	1.794
-9.010	1.869	-9.040	1.795
-9.000	1.869	-9.030	1.795
-8.990	1.869	-9.020	1.796
-8.980	1.870	-9.010	1.796

-8.970	1.870	-9.000	1.797
-8.960	1.871	-8.990	1.797
-8.950	1.871	-8.980	1.798
-8.940	1.872	-8.970	1.798
-8.930	1.872	-8.960	1.798
-8.920	1.873	-8.950	1.798
-8.910	1.873	-8.940	1.798
-8.900	1.874	-8.930	1.799
-8.890	1.874	-8.920	1.799
-8.880	1.875	-8.910	1.799
-8.870	1.876	-8.900	1.799
-8.860	1.876	-8.890	1.799
-8.850	1.877	-8.880	1.799
-8.840	1.877	-8.870	1.799
-8.830	1.878	-8.860	1.799
-8.820	1.878	-8.850	1.799
-8.810	1.879	-8.840	1.799
-8.800	1.879	-8.830	1.799
-8.790	1.879	-8.820	1.798
-8.780	1.880	-8.810	1.799
-8.770	1.880	-8.800	1.799
-8.760	1.880	-8.790	1.800
-8.750	1.880	-8.780	1.800
-8.740	1.881	-8.770	1.801
-8.730	1.881	-8.760	1.801
-8.720	1.880	-8.750	1.801
-8.710	1.880	-8.740	1.802
-8.700	1.880	-8.730	1.802
-8.690	1.880	-8.720	1.802
-8.680	1.880	-8.710	1.802
-8.670	1.880	-8.700	1.802
-8.660	1.879	-8.690	1.802
-8.650	1.879	-8.680	1.802
-8.640	1.879	-8.670	1.802
-8.630	1.879	-8.660	1.802
-8.620	1.879	-8.650	1.802
-8.610	1.879	-8.640	1.801
-8.600	1.878	-8.630	1.801
-8.590	1.878	-8.620	1.801
-8.580	1.878	-8.610	1.801
-8.570	1.878	-8.600	1.800
-8.560	1.878	-8.590	1.800
-8.550	1.878	-8.580	1.800
-8.540	1.878	-8.570	1.799
-8.530	1.878	-8.560	1.799
-8.520	1.877	-8.550	1.799
-8.510	1.877	-8.540	1.799
-8.500	1.877	-8.530	1.798
-8.490	1.876	-8.520	1.798
-8.480	1.876	-8.510	1.798
-8.470	1.876	-8.500	1.798
-8.460	1.876	-8.490	1.798
-8.450	1.876	-8.480	1.798
-8.440	1.876	-8.470	1.798
-8.430	1.876	-8.460	1.798
-8.420	1.876	-8.450	1.798

-8.410	1.876	-8.440	1.799
-8.400	1.876	-8.430	1.799
-8.390	1.876	-8.420	1.799
-8.380	1.876	-8.410	1.799
-8.370	1.876	-8.400	1.799
-8.360	1.876	-8.390	1.799
-8.350	1.876	-8.380	1.799
-8.340	1.876	-8.370	1.799
-8.330	1.875	-8.360	1.799
-8.320	1.875	-8.350	1.798
-8.310	1.874	-8.340	1.798
-8.300	1.874	-8.330	1.798
-8.290	1.874	-8.320	1.798
-8.280	1.874	-8.310	1.797
-8.270	1.874	-8.300	1.797
-8.260	1.874	-8.290	1.797
-8.250	1.875	-8.280	1.796
-8.240	1.875	-8.270	1.796
-8.230	1.875	-8.260	1.795
-8.220	1.875	-8.250	1.795
-8.210	1.875	-8.240	1.795
-8.200	1.875	-8.230	1.794
-8.190	1.875	-8.220	1.794
-8.180	1.874	-8.210	1.793
-8.170	1.874	-8.200	1.793
-8.160	1.874	-8.190	1.792
-8.150	1.875	-8.180	1.792
-8.140	1.875	-8.170	1.791
-8.130	1.875	-8.160	1.791
-8.120	1.875	-8.150	1.791
-8.110	1.875	-8.140	1.791
-8.100	1.875	-8.130	1.792
-8.090	1.875	-8.120	1.792
-8.080	1.875	-8.110	1.792
-8.070	1.875	-8.100	1.792
-8.060	1.875	-8.090	1.792
-8.050	1.875	-8.080	1.792
-8.040	1.875	-8.070	1.792
-8.030	1.875	-8.060	1.793
-8.020	1.875	-8.050	1.793
-8.010	1.876	-8.040	1.793
-8.000	1.876	-8.030	1.793
-7.990	1.876	-8.020	1.793
-7.980	1.876	-8.010	1.793
-7.970	1.876	-8.000	1.793
-7.960	1.876	-7.990	1.794
-7.950	1.876	-7.980	1.794
-7.940	1.876	-7.970	1.794
-7.930	1.876	-7.960	1.794
-7.920	1.876	-7.950	1.794
-7.910	1.876	-7.940	1.794
-7.900	1.876	-7.930	1.795
-7.890	1.876	-7.920	1.795
-7.880	1.876	-7.910	1.795
-7.870	1.875	-7.900	1.795
-7.860	1.875	-7.890	1.795

-7.850	1.875	-7.880	1.796
-7.840	1.874	-7.870	1.796
-7.830	1.873	-7.860	1.796
-7.820	1.873	-7.850	1.796
-7.810	1.872	-7.840	1.796
-7.800	1.871	-7.830	1.796
-7.790	1.871	-7.820	1.796
-7.780	1.870	-7.810	1.796
-7.770	1.869	-7.800	1.796
-7.760	1.869	-7.790	1.796
-7.750	1.868	-7.780	1.796
-7.740	1.868	-7.770	1.796
-7.730	1.868	-7.760	1.796
-7.720	1.868	-7.750	1.796
-7.710	1.868	-7.740	1.797
-7.700	1.869	-7.730	1.797
-7.690	1.869	-7.720	1.797
-7.680	1.869	-7.710	1.797
-7.670	1.869	-7.700	1.797
-7.660	1.869	-7.690	1.797
-7.650	1.869	-7.680	1.797
-7.640	1.869	-7.670	1.797
-7.630	1.868	-7.660	1.797
-7.620	1.868	-7.650	1.797
-7.610	1.868	-7.640	1.797
-7.600	1.867	-7.630	1.797
-7.590	1.867	-7.620	1.797
-7.580	1.867	-7.610	1.797
-7.570	1.867	-7.600	1.796
-7.560	1.867	-7.590	1.796
-7.550	1.867	-7.580	1.796
-7.540	1.868	-7.570	1.796
-7.530	1.868	-7.560	1.796
-7.520	1.868	-7.550	1.796
-7.510	1.868	-7.540	1.795
-7.500	1.868	-7.530	1.795
-7.490	1.869	-7.520	1.795
-7.480	1.869	-7.510	1.795
-7.470	1.869	-7.500	1.794
-7.460	1.869	-7.490	1.794
-7.450	1.869	-7.480	1.794
-7.440	1.870	-7.470	1.794
-7.430	1.870	-7.460	1.794
-7.420	1.870	-7.450	1.794
-7.410	1.870	-7.440	1.794
-7.400	1.870	-7.430	1.794
-7.390	1.871	-7.420	1.794
-7.380	1.871	-7.410	1.794
-7.370	1.871	-7.400	1.794
-7.360	1.871	-7.390	1.794
-7.350	1.871	-7.380	1.794
-7.340	1.871	-7.370	1.794
-7.330	1.870	-7.360	1.794
-7.320	1.871	-7.350	1.794
-7.310	1.871	-7.340	1.794
-7.300	1.872	-7.330	1.794

-7.290	1.872	-7.320	1.794
-7.280	1.873	-7.310	1.794
-7.270	1.873	-7.300	1.794
-7.260	1.874	-7.290	1.794
-7.250	1.874	-7.280	1.794
-7.240	1.875	-7.270	1.794
-7.230	1.875	-7.260	1.794
-7.220	1.876	-7.250	1.794
-7.210	1.876	-7.240	1.794
-7.200	1.877	-7.230	1.794
-7.190	1.878	-7.220	1.794
-7.180	1.878	-7.210	1.794
-7.170	1.879	-7.200	1.794
-7.160	1.880	-7.190	1.794
-7.150	1.880	-7.180	1.794
-7.140	1.881	-7.170	1.794
-7.130	1.881	-7.160	1.794
-7.120	1.882	-7.150	1.795
-7.110	1.882	-7.140	1.795
-7.100	1.883	-7.130	1.795
-7.090	1.883	-7.120	1.795
-7.080	1.884	-7.110	1.795
-7.070	1.884	-7.100	1.795
-7.060	1.885	-7.090	1.796
-7.050	1.885	-7.080	1.796
-7.040	1.886	-7.070	1.796
-7.030	1.886	-7.060	1.795
-7.020	1.887	-7.050	1.795
-7.010	1.887	-7.040	1.795
-7.000	1.888	-7.030	1.795
-6.990	1.888	-7.020	1.795
-6.980	1.888	-7.010	1.796
-6.970	1.889	-7.000	1.796
-6.960	1.889	-6.990	1.796
-6.950	1.889	-6.980	1.797
-6.940	1.889	-6.970	1.797
-6.930	1.889	-6.960	1.798
-6.920	1.889	-6.950	1.798
-6.910	1.889	-6.940	1.798
-6.900	1.889	-6.930	1.799
-6.890	1.888	-6.920	1.799
-6.880	1.888	-6.910	1.799
-6.870	1.887	-6.900	1.799
-6.860	1.887	-6.890	1.799
-6.850	1.886	-6.880	1.799
-6.840	1.886	-6.870	1.799
-6.830	1.886	-6.860	1.798
-6.820	1.886	-6.850	1.798
-6.810	1.886	-6.840	1.798
-6.800	1.886	-6.830	1.798
-6.790	1.886	-6.820	1.798
-6.780	1.885	-6.810	1.797
-6.770	1.885	-6.800	1.797
-6.760	1.884	-6.790	1.796
-6.750	1.883	-6.780	1.796
-6.740	1.883	-6.770	1.795

-6.730	1.882	-6.760	1.795
-6.720	1.881	-6.750	1.795
-6.710	1.881	-6.740	1.795
-6.700	1.880	-6.730	1.794
-6.690	1.880	-6.720	1.794
-6.680	1.880	-6.710	1.794
-6.670	1.880	-6.700	1.794
-6.660	1.880	-6.690	1.794
-6.650	1.880	-6.680	1.794
-6.640	1.880	-6.670	1.794
-6.630	1.879	-6.660	1.794
-6.620	1.879	-6.650	1.794
-6.610	1.878	-6.640	1.794
-6.600	1.878	-6.630	1.793
-6.590	1.878	-6.620	1.793
-6.580	1.877	-6.610	1.793
-6.570	1.878	-6.600	1.793
-6.560	1.878	-6.590	1.793
-6.550	1.879	-6.580	1.793
-6.540	1.879	-6.570	1.793
-6.530	1.880	-6.560	1.794
-6.520	1.880	-6.550	1.794
-6.510	1.880	-6.540	1.794
-6.500	1.881	-6.530	1.794
-6.490	1.881	-6.520	1.794
-6.480	1.881	-6.510	1.794
-6.470	1.881	-6.500	1.795
-6.460	1.881	-6.490	1.795
-6.450	1.880	-6.480	1.795
-6.440	1.880	-6.470	1.795
-6.430	1.880	-6.460	1.795
-6.420	1.879	-6.450	1.795
-6.410	1.879	-6.440	1.796
-6.400	1.879	-6.430	1.796
-6.390	1.879	-6.420	1.796
-6.380	1.879	-6.410	1.797
-6.370	1.879	-6.400	1.797
-6.360	1.878	-6.390	1.798
-6.350	1.878	-6.380	1.798
-6.340	1.878	-6.370	1.798
-6.330	1.878	-6.360	1.799
-6.320	1.877	-6.350	1.799
-6.310	1.877	-6.340	1.799
-6.300	1.877	-6.330	1.799
-6.290	1.877	-6.320	1.799
-6.280	1.877	-6.310	1.799
-6.270	1.877	-6.300	1.800
-6.260	1.877	-6.290	1.800
-6.250	1.876	-6.280	1.800
-6.240	1.876	-6.270	1.800
-6.230	1.876	-6.260	1.799
-6.220	1.876	-6.250	1.799
-6.210	1.876	-6.240	1.799
-6.200	1.876	-6.230	1.799
-6.190	1.875	-6.220	1.799
-6.180	1.875	-6.210	1.799

-6.170	1.875	-6.200	1.799
-6.160	1.875	-6.190	1.799
-6.150	1.875	-6.180	1.799
-6.140	1.875	-6.170	1.799
-6.130	1.874	-6.160	1.799
-6.120	1.874	-6.150	1.799
-6.110	1.874	-6.140	1.799
-6.100	1.874	-6.130	1.799
-6.090	1.874	-6.120	1.799
-6.080	1.874	-6.110	1.799
-6.070	1.874	-6.100	1.800
-6.060	1.874	-6.090	1.800
-6.050	1.874	-6.080	1.800
-6.040	1.874	-6.070	1.800
-6.030	1.874	-6.060	1.800
-6.020	1.874	-6.050	1.800
-6.010	1.873	-6.040	1.800
-6.000	1.873	-6.030	1.800
-5.990	1.873	-6.020	1.800
-5.980	1.873	-6.010	1.800
-5.970	1.873	-6.000	1.799
-5.960	1.874	-5.990	1.799
-5.950	1.874	-5.980	1.799
-5.940	1.874	-5.970	1.798
-5.930	1.874	-5.960	1.798
-5.920	1.874	-5.950	1.798
-5.910	1.874	-5.940	1.797
-5.900	1.874	-5.930	1.797
-5.890	1.873	-5.920	1.797
-5.880	1.873	-5.910	1.797
-5.870	1.873	-5.900	1.797
-5.860	1.873	-5.890	1.796
-5.850	1.872	-5.880	1.796
-5.840	1.872	-5.870	1.796
-5.830	1.871	-5.860	1.796
-5.820	1.870	-5.850	1.796
-5.810	1.870	-5.840	1.796
-5.800	1.869	-5.830	1.796
-5.790	1.868	-5.820	1.795
-5.780	1.868	-5.810	1.795
-5.770	1.868	-5.800	1.795
-5.760	1.868	-5.790	1.796
-5.750	1.868	-5.780	1.796
-5.740	1.868	-5.770	1.796
-5.730	1.868	-5.760	1.796
-5.720	1.868	-5.750	1.796
-5.710	1.868	-5.740	1.796
-5.700	1.868	-5.730	1.796
-5.690	1.868	-5.720	1.796
-5.680	1.868	-5.710	1.796
-5.670	1.868	-5.700	1.796
-5.660	1.868	-5.690	1.795
-5.650	1.868	-5.680	1.795
-5.640	1.868	-5.670	1.795
-5.630	1.868	-5.660	1.795
-5.620	1.868	-5.650	1.795

-5.610	1.868	-5.640	1.795
-5.600	1.868	-5.630	1.795
-5.590	1.868	-5.620	1.794
-5.580	1.869	-5.610	1.793
-5.570	1.870	-5.600	1.793
-5.560	1.870	-5.590	1.792
-5.550	1.871	-5.580	1.792
-5.540	1.871	-5.570	1.791
-5.530	1.872	-5.560	1.790
-5.520	1.873	-5.550	1.790
-5.510	1.873	-5.540	1.789
-5.500	1.874	-5.530	1.789
-5.490	1.875	-5.520	1.789
-5.480	1.875	-5.510	1.788
-5.470	1.876	-5.500	1.789
-5.460	1.877	-5.490	1.789
-5.450	1.877	-5.480	1.789
-5.440	1.878	-5.470	1.789
-5.430	1.878	-5.460	1.789
-5.420	1.879	-5.450	1.789
-5.410	1.879	-5.440	1.790
-5.400	1.880	-5.430	1.790
-5.390	1.880	-5.420	1.791
-5.380	1.880	-5.410	1.791
-5.370	1.881	-5.400	1.791
-5.360	1.881	-5.390	1.792
-5.350	1.881	-5.380	1.792
-5.340	1.882	-5.370	1.792
-5.330	1.882	-5.360	1.793
-5.320	1.882	-5.350	1.793
-5.310	1.882	-5.340	1.793
-5.300	1.883	-5.330	1.794
-5.290	1.883	-5.320	1.794
-5.280	1.883	-5.310	1.795
-5.270	1.883	-5.300	1.795
-5.260	1.882	-5.290	1.796
-5.250	1.882	-5.280	1.796
-5.240	1.882	-5.270	1.796
-5.230	1.881	-5.260	1.797
-5.220	1.881	-5.250	1.797
-5.210	1.880	-5.240	1.797
-5.200	1.879	-5.230	1.797
-5.190	1.879	-5.220	1.798
-5.180	1.878	-5.210	1.798
-5.170	1.881	-5.200	1.798
-5.160	1.883	-5.190	1.798
-5.150	1.885	-5.180	1.798
-5.140	1.885	-5.170	1.798
-5.130	1.885	-5.160	1.798
-5.120	1.885	-5.150	1.797
-5.110	1.885	-5.140	1.797
-5.100	1.885	-5.130	1.797
-5.090	1.885	-5.120	1.797
-5.080	1.885	-5.110	1.796
-5.070	1.884	-5.100	1.796
-5.060	1.884	-5.090	1.795

-5.050	1.884	-5.080	1.795
-5.040	1.883	-5.070	1.795
-5.030	1.883	-5.060	1.795
-5.020	1.882	-5.050	1.795
-5.010	1.881	-5.040	1.795
-5.000	1.880	-5.030	1.795
-4.990	1.879	-5.020	1.795
-4.980	1.879	-5.010	1.795
-4.970	1.878	-5.000	1.795
-4.960	1.878	-4.990	1.795
-4.950	1.878	-4.980	1.795
-4.940	1.878	-4.970	1.795
-4.930	1.878	-4.960	1.795
-4.920	1.878	-4.950	1.795
-4.910	1.878	-4.940	1.796
-4.900	1.878	-4.930	1.796
-4.890	1.877	-4.920	1.796
-4.880	1.877	-4.910	1.797
-4.870	1.876	-4.900	1.797
-4.860	1.876	-4.890	1.797
-4.850	1.876	-4.880	1.797
-4.840	1.875	-4.870	1.797
-4.830	1.875	-4.860	1.797
-4.820	1.874	-4.850	1.797
-4.810	1.874	-4.840	1.797
-4.800	1.874	-4.830	1.797
-4.790	1.874	-4.820	1.797
-4.780	1.873	-4.810	1.797
-4.770	1.873	-4.800	1.797
-4.760	1.873	-4.790	1.797
-4.750	1.873	-4.780	1.797
-4.740	1.873	-4.770	1.797
-4.730	1.874	-4.760	1.797
-4.720	1.874	-4.750	1.797
-4.710	1.875	-4.740	1.797
-4.700	1.876	-4.730	1.797
-4.690	1.876	-4.720	1.797
-4.680	1.876	-4.710	1.797
-4.670	1.877	-4.700	1.797
-4.660	1.877	-4.690	1.797
-4.650	1.878	-4.680	1.797
-4.640	1.878	-4.670	1.797
-4.630	1.878	-4.660	1.797
-4.620	1.879	-4.650	1.797
-4.610	1.879	-4.640	1.797
-4.600	1.879	-4.630	1.797
-4.590	1.880	-4.620	1.798
-4.580	1.880	-4.610	1.798
-4.570	1.880	-4.600	1.798
-4.560	1.880	-4.590	1.798
-4.550	1.880	-4.580	1.797
-4.540	1.880	-4.570	1.797
-4.530	1.880	-4.560	1.797
-4.520	1.880	-4.550	1.797
-4.510	1.880	-4.540	1.797
-4.500	1.881	-4.530	1.796

-4.490	1.881	-4.520	1.796
-4.480	1.881	-4.510	1.795
-4.470	1.881	-4.500	1.795
-4.460	1.881	-4.490	1.794
-4.450	1.881	-4.480	1.794
-4.440	1.881	-4.470	1.793
-4.430	1.882	-4.460	1.792
-4.420	1.882	-4.450	1.792
-4.410	1.882	-4.440	1.791
-4.400	1.883	-4.430	1.791
-4.390	1.883	-4.420	1.790
-4.380	1.883	-4.410	1.790
-4.370	1.884	-4.400	1.790
-4.360	1.884	-4.390	1.789
-4.350	1.884	-4.380	1.789
-4.340	1.884	-4.370	1.789
-4.330	1.885	-4.360	1.788
-4.320	1.885	-4.350	1.789
-4.310	1.885	-4.340	1.789
-4.300	1.885	-4.330	1.789
-4.290	1.885	-4.320	1.789
-4.280	1.886	-4.310	1.789
-4.270	1.886	-4.300	1.789
-4.260	1.886	-4.290	1.789
-4.250	1.886	-4.280	1.790
-4.240	1.886	-4.270	1.790
-4.230	1.887	-4.260	1.790
-4.220	1.887	-4.250	1.790
-4.210	1.887	-4.240	1.790
-4.200	1.887	-4.230	1.790
-4.190	1.887	-4.220	1.790
-4.180	1.887	-4.210	1.790
-4.170	1.887	-4.200	1.791
-4.160	1.887	-4.190	1.791
-4.150	1.887	-4.180	1.791
-4.140	1.887	-4.170	1.791
-4.130	1.887	-4.160	1.791
-4.120	1.886	-4.150	1.791
-4.110	1.886	-4.140	1.791
-4.100	1.886	-4.130	1.791
-4.090	1.886	-4.120	1.792
-4.080	1.886	-4.110	1.792
-4.070	1.886	-4.100	1.792
-4.060	1.885	-4.090	1.792
-4.050	1.885	-4.080	1.792
-4.040	1.885	-4.070	1.793
-4.030	1.885	-4.060	1.793
-4.020	1.885	-4.050	1.794
-4.010	1.885	-4.040	1.794
-4.000	1.885	-4.030	1.795
-3.990	1.885	-4.020	1.796
-3.980	1.885	-4.010	1.796
-3.970	1.886	-4.000	1.797
-3.960	1.886	-3.990	1.797
-3.950	1.886	-3.980	1.797
-3.940	1.886	-3.970	1.797

-3.930	1.887	-3.960	1.797
-3.920	1.887	-3.950	1.797
-3.910	1.887	-3.940	1.797
-3.900	1.887	-3.930	1.797
-3.890	1.887	-3.920	1.797
-3.880	1.887	-3.910	1.797
-3.870	1.887	-3.900	1.797
-3.860	1.887	-3.890	1.797
-3.850	1.887	-3.880	1.796
-3.840	1.887	-3.870	1.796
-3.830	1.887	-3.860	1.796
-3.820	1.887	-3.850	1.797
-3.810	1.887	-3.840	1.797
-3.800	1.888	-3.830	1.797
-3.790	1.890	-3.820	1.797
-3.780	1.892	-3.810	1.797
-3.770	1.894	-3.800	1.797
-3.760	1.897	-3.790	1.797
-3.750	1.900	-3.780	1.797
-3.740	1.902	-3.770	1.797
-3.730	1.904	-3.760	1.796
-3.720	1.908	-3.750	1.796
-3.710	1.912	-3.740	1.796
-3.700	1.916	-3.730	1.796
-3.690	1.920	-3.720	1.795
-3.680	1.924	-3.710	1.795
-3.670	1.928	-3.700	1.795
-3.660	1.931	-3.690	1.795
-3.650	1.935	-3.680	1.794
-3.640	1.939	-3.670	1.794
-3.630	1.943	-3.660	1.794
-3.620	1.947	-3.650	1.794
-3.610	1.951	-3.640	1.794
-3.600	1.955	-3.630	1.794
-3.590	1.958	-3.620	1.794
-3.580	1.962	-3.610	1.794
-3.570	1.966	-3.600	1.794
-3.560	1.970	-3.590	1.793
-3.550	1.974	-3.580	1.793
-3.540	1.978	-3.570	1.793
-3.530	1.982	-3.560	1.793
-3.520	1.985	-3.550	1.792
-3.510	1.989	-3.540	1.792
-3.500	1.993	-3.530	1.792
-3.490	1.997	-3.520	1.792
-3.480	2.001	-3.510	1.792
-3.470	2.005	-3.500	1.792
-3.460	2.009	-3.490	1.792
-3.450	2.012	-3.480	1.792
-3.440	2.016	-3.470	1.792
-3.430	2.020	-3.460	1.792
-3.420	2.024	-3.450	1.791
-3.410	2.028	-3.440	1.791
-3.400	2.032	-3.430	1.791
-3.390	2.035	-3.420	1.791
-3.380	2.039	-3.410	1.790

-3.370	2.043	-3.400	1.790
-3.360	2.047	-3.390	1.790
-3.350	2.051	-3.380	1.790
-3.340	2.055	-3.370	1.790
-3.330	2.059	-3.360	1.790
-3.320	2.062	-3.350	1.790
-3.310	2.066	-3.340	1.790
-3.300	2.070	-3.330	1.790
-3.290	2.074	-3.320	1.790
-3.280	2.078	-3.310	1.790
-3.270	2.082	-3.300	1.790
-3.260	2.085	-3.290	1.790
-3.250	2.088	-3.280	1.791
-3.240	2.091	-3.270	1.791
-3.230	2.094	-3.260	1.791
-3.220	2.096	-3.250	1.791
-3.210	2.099	-3.240	1.792
-3.200	2.102	-3.230	1.792
-3.190	2.105	-3.220	1.791
-3.180	2.107	-3.210	1.791
-3.170	2.108	-3.200	1.791
-3.160	2.110	-3.190	1.791
-3.150	2.111	-3.180	1.790
-3.140	2.112	-3.170	1.790
-3.130	2.113	-3.160	1.790
-3.120	2.114	-3.150	1.790
-3.110	2.115	-3.140	1.790
-3.100	2.116	-3.130	1.790
-3.090	2.117	-3.120	1.790
-3.080	2.118	-3.110	1.790
-3.070	2.119	-3.100	1.790
-3.060	2.119	-3.090	1.790
-3.050	2.120	-3.080	1.790
-3.040	2.121	-3.070	1.790
-3.030	2.122	-3.060	1.789
-3.020	2.122	-3.050	1.789
-3.010	2.123	-3.040	1.789
-3.000	2.124	-3.030	1.789
-2.990	2.124	-3.020	1.788
-2.980	2.125	-3.010	1.788
-2.970	2.126	-3.000	1.788
-2.960	2.126	-2.990	1.788
-2.950	2.127	-2.980	1.787
-2.940	2.127	-2.970	1.787
-2.930	2.127	-2.960	1.786
-2.920	2.127	-2.950	1.786
-2.910	2.127	-2.940	1.786
-2.900	2.128	-2.930	1.786
-2.890	2.128	-2.920	1.786
-2.880	2.128	-2.910	1.785
-2.870	2.128	-2.900	1.785
-2.860	2.128	-2.890	1.785
-2.850	2.128	-2.880	1.785
-2.840	2.128	-2.870	1.785
-2.830	2.128	-2.860	1.785
-2.820	2.128	-2.850	1.785

-2.810	2.128	-2.840	1.785
-2.800	2.128	-2.830	1.785
-2.790	2.128	-2.820	1.786
-2.780	2.128	-2.810	1.786
-2.770	2.128	-2.800	1.786
-2.760	2.128	-2.790	1.786
-2.750	2.128	-2.780	1.787
-2.740	2.128	-2.770	1.787
-2.730	2.128	-2.760	1.787
-2.720	2.128	-2.750	1.787
-2.710	2.128	-2.740	1.787
-2.700	2.128	-2.730	1.788
-2.690	2.128	-2.720	1.788
-2.680	2.128	-2.710	1.788
-2.670	2.128	-2.700	1.788
-2.660	2.128	-2.690	1.788
-2.650	2.128	-2.680	1.788
-2.640	2.128	-2.670	1.789
-2.630	2.128	-2.660	1.789
-2.620	2.128	-2.650	1.789
-2.610	2.127	-2.640	1.789
-2.600	2.127	-2.630	1.789
-2.590	2.127	-2.620	1.789
-2.580	2.127	-2.610	1.790
-2.570	2.127	-2.600	1.790
-2.560	2.127	-2.590	1.791
-2.550	2.127	-2.580	1.791
-2.540	2.128	-2.570	1.791
-2.530	2.128	-2.560	1.791
-2.520	2.128	-2.550	1.792
-2.510	2.128	-2.540	1.793
-2.500	2.128	-2.530	1.794
-2.490	2.128	-2.520	1.795
-2.480	2.128	-2.510	1.796
-2.470	2.127	-2.500	1.797
-2.460	2.127	-2.490	1.799
-2.450	2.127	-2.480	1.802
-2.440	2.127	-2.470	1.804
-2.430	2.127	-2.460	1.807
-2.420	2.127	-2.450	1.809
-2.410	2.126	-2.440	1.811
-2.400	2.126	-2.430	1.814
-2.390	2.126	-2.420	1.816
-2.380	2.126	-2.410	1.819
-2.370	2.126	-2.400	1.822
-2.360	2.126	-2.390	1.824
-2.350	2.126	-2.380	1.827
-2.340	2.126	-2.370	1.830
-2.330	2.126	-2.360	1.833
-2.320	2.126	-2.350	1.836
-2.310	2.126	-2.340	1.838
-2.300	2.126	-2.330	1.841
-2.290	2.126	-2.320	1.844
-2.280	2.126	-2.310	1.847
-2.270	2.126	-2.300	1.850
-2.260	2.126	-2.290	1.852

-2.250	2.126	-2.280	1.855
-2.240	2.126	-2.270	1.858
-2.230	2.125	-2.260	1.861
-2.220	2.125	-2.250	1.864
-2.210	2.125	-2.240	1.866
-2.200	2.125	-2.230	1.869
-2.190	2.125	-2.220	1.873
-2.180	2.125	-2.210	1.877
-2.170	2.125	-2.200	1.880
-2.160	2.125	-2.190	1.884
-2.150	2.125	-2.180	1.888
-2.140	2.125	-2.170	1.892
-2.130	2.125	-2.160	1.895
-2.120	2.125	-2.150	1.899
-2.110	2.125	-2.140	1.903
-2.100	2.125	-2.130	1.907
-2.090	2.126	-2.120	1.912
-2.080	2.126	-2.110	1.916
-2.070	2.126	-2.100	1.920
-2.060	2.126	-2.090	1.925
-2.050	2.127	-2.080	1.929
-2.040	2.127	-2.070	1.933
-2.030	2.127	-2.060	1.938
-2.020	2.126	-2.050	1.942
-2.010	2.126	-2.040	1.946
-2.000	2.126	-2.030	1.951
-1.990	2.126	-2.020	1.955
-1.980	2.126	-2.010	1.960
-1.970	2.126	-2.000	1.964
-1.960	2.126	-1.990	1.968
-1.950	2.126	-1.980	1.973
-1.940	2.126	-1.970	1.977
-1.930	2.126	-1.960	1.981
-1.920	2.127	-1.950	1.986
-1.910	2.127	-1.940	1.990
-1.900	2.127	-1.930	1.995
-1.890	2.127	-1.920	1.999
-1.880	2.127	-1.910	2.003
-1.870	2.127	-1.900	2.008
-1.860	2.127	-1.890	2.012
-1.850	2.127	-1.880	2.015
-1.840	2.127	-1.870	2.018
-1.830	2.127	-1.860	2.022
-1.820	2.128	-1.850	2.025
-1.810	2.128	-1.840	2.028
-1.800	2.128	-1.830	2.032
-1.790	2.128	-1.820	2.035
-1.780	2.129	-1.810	2.038
-1.770	2.129	-1.800	2.042
-1.760	2.129	-1.790	2.045
-1.750	2.129	-1.780	2.048
-1.740	2.129	-1.770	2.051
-1.730	2.129	-1.760	2.054
-1.720	2.128	-1.750	2.057
-1.710	2.128	-1.740	2.059
-1.700	2.127	-1.730	2.061

-1.690	2.127	-1.720	2.064
-1.680	2.126	-1.710	2.066
-1.670	2.126	-1.700	2.067
-1.660	2.125	-1.690	2.069
-1.650	2.125	-1.680	2.070
-1.640	2.124	-1.670	2.072
-1.630	2.124	-1.660	2.073
-1.620	2.124	-1.650	2.074
-1.610	2.123	-1.640	2.074
-1.600	2.123	-1.630	2.075
-1.590	2.122	-1.620	2.075
-1.580	2.122	-1.610	2.075
-1.570	2.122	-1.600	2.075
-1.560	2.122	-1.590	2.075
-1.550	2.121	-1.580	2.075
-1.540	2.121	-1.570	2.075
-1.530	2.121	-1.560	2.075
-1.520	2.120	-1.550	2.075
-1.510	2.120	-1.540	2.075
-1.500	2.120	-1.530	2.074
-1.490	2.120	-1.520	2.074
-1.480	2.119	-1.510	2.074
-1.470	2.119	-1.500	2.073
-1.460	2.118	-1.490	2.073
-1.450	2.118	-1.480	2.073
-1.440	2.118	-1.470	2.073
-1.430	2.117	-1.460	2.073
-1.420	2.117	-1.450	2.073
-1.410	2.116	-1.440	2.072
-1.400	2.115	-1.430	2.072
-1.390	2.115	-1.420	2.072
-1.380	2.114	-1.410	2.072
-1.370	2.114	-1.400	2.072
-1.360	2.114	-1.390	2.071
-1.350	2.113	-1.380	2.071
-1.340	2.113	-1.370	2.071
-1.330	2.113	-1.360	2.071
-1.320	2.113	-1.350	2.070
-1.310	2.112	-1.340	2.070
-1.300	2.112	-1.330	2.070
-1.290	2.112	-1.320	2.070
-1.280	2.112	-1.310	2.070
-1.270	2.111	-1.300	2.069
-1.260	2.111	-1.290	2.069
-1.250	2.111	-1.280	2.069
-1.240	2.111	-1.270	2.069
-1.230	2.111	-1.260	2.069
-1.220	2.110	-1.250	2.069
-1.210	2.110	-1.240	2.069
-1.200	2.110	-1.230	2.070
-1.190	2.110	-1.220	2.070
-1.180	2.109	-1.210	2.070
-1.170	2.109	-1.200	2.070
-1.160	2.109	-1.190	2.070
-1.150	2.110	-1.180	2.070
-1.140	2.110	-1.170	2.070

-1.130	2.110
-1.120	2.110
-1.110	2.110
-1.100	2.110
-1.090	2.110
-1.080	2.110
-1.070	2.110
-1.060	2.110
-1.050	2.110
-1.040	2.110
-1.030	2.110
-1.020	2.110
-1.010	2.110
-1.000	2.111
-0.990	2.111
-0.980	2.111
-0.970	2.111
-0.960	2.111
-0.950	2.111
-0.940	2.111
-0.930	2.111
-0.920	2.111
-0.910	2.111
-0.900	2.111
-0.890	2.111
-0.880	2.111
-0.870	2.111
-0.860	2.111
-0.850	2.111
-0.840	2.111
-0.830	2.111
-0.820	2.111
-0.810	2.110
-0.800	2.110
-0.790	2.110
-0.780	2.110
-0.770	2.110
-0.760	2.110
-0.750	2.110
-0.740	2.110
-0.730	2.110
-0.720	2.110
-0.710	2.110
-0.700	2.110
-0.690	2.110
-0.680	2.110
-0.670	2.110
-0.660	2.110
-0.650	2.110
-0.640	2.110
-0.630	2.110
-0.620	2.111
-0.610	2.111
-0.600	2.111
-0.590	2.111
-0.580	2.111

-1.160	2.070
-1.150	2.071
-1.140	2.071
-1.130	2.071
-1.120	2.072
-1.110	2.072
-1.100	2.072
-1.090	2.072
-1.080	2.072
-1.070	2.073
-1.060	2.073
-1.050	2.073
-1.040	2.073
-1.030	2.073
-1.020	2.074
-1.010	2.074
-1.000	2.074
-0.990	2.075
-0.980	2.076
-0.970	2.077
-0.960	2.077
-0.950	2.078
-0.940	2.078
-0.930	2.079
-0.920	2.079
-0.910	2.080
-0.900	2.080
-0.890	2.080
-0.880	2.081
-0.870	2.081
-0.860	2.081
-0.850	2.081
-0.840	2.081
-0.830	2.081
-0.820	2.081
-0.810	2.081
-0.800	2.081
-0.790	2.081
-0.780	2.081
-0.770	2.080
-0.760	2.080
-0.750	2.080

-0.570	2.111
-0.560	2.111
-0.550	2.112
-0.540	2.112
-0.530	2.112
-0.520	2.112
-0.510	2.111
-0.500	2.111
-0.490	2.111
-0.480	2.111
-0.470	2.111
-0.460	2.111
-0.450	2.111
-0.440	2.111
-0.430	2.111
-0.420	2.111
-0.410	2.111
-0.400	2.111
-0.390	2.111
-0.380	2.111
-0.370	2.111
-0.360	2.111
-0.350	2.110
