# Supplementary material for: Impact of drill bit wear on temperature increase in dental implant osteotomy: an in vitro study
Source: PLoS One. 2025 Mar 19;20(3):e0319492. doi: 10.1371/journal.pone.0319492 (PMC11922234; doi:10.1371/journal.pone.0319492)
Supplement: S3 Table — This table presents all the data obtained from the wear assessment. Specifically, it includes the worn area and cutting edge wear measurements for both the AT and PT protocols, recorded from hole 1 to hole 150 at intervals of every 10 holes. (PDF) [file pone.0319492.s003.pdf]

Prot.	hole nr.	Worn area (mm^2)				Cutting edge (mm)			
		Run1	Run2	Run3	**AVG**	Run1	Run2	Run3	**AVG**
**AT 1**	0	0.01	0.02	0.01	0.01	0.00	0.01	0.00	0.00
	1	0.04	0.05	0.06	0.05	0.01	0.01	0.02	0.01
	10	0.52	0.53	0.54	0.53	0.12	0.13	0.11	0.12
	20	0.76	0.77	0.78	0.77	0.17	0.18	0.16	0.17
	30	0.83	0.84	0.85	0.84	0.19	0.20	0.18	0.19
	40	0.87	0.88	0.89	0.88	0.29	0.30	0.28	0.29
	50	0.88	0.89	0.90	0.89	0.31	0.32	0.30	0.31
	100	0.98	0.99	1.00	0.99	0.49	0.50	0.48	0.49
	150	1.00	1.01	1.02	1.01	0.62	0.63	0.61	0.62
**AT 2**	0	0.00	0.01	0.00	0.00	0.00	0.01	0.00	0.00
	1	0.04	0.03	0.05	0.04	0.06	0.05	0.07	0.06
	10	0.47	0.46	0.48	0.47	0.23	0.22	0.24	0.23
	20	0.68	0.67	0.69	0.68	0.29	0.28	0.30	0.29
	30	0.82	0.83	0.81	0.82	0.33	0.34	0.32	0.33
	40	0.91	0.92	0.90	0.91	0.36	0.35	0.37	0.36
	50	1.01	1.00	1.02	1.01	0.39	0.38	0.40	0.39
	100	1.49	1.50	1.48	1.49	0.59	0.58	0.60	0.59
	150	1.61	1.60	1.62	1.61	0.76	0.75	0.77	0.76
**AT 3**	0	0.00	0.01	0.00	0.00	0.00	0.01	0.00	0.00
	1	0.07	0.06	0.08	0.07	0.03	0.02	0.04	0.03
	10	0.39	0.40	0.38	0.39	0.13	0.12	0.14	0.13
	20	0.54	0.53	0.55	0.54	0.17	0.16	0.18	0.17
	30	0.70	0.71	0.69	0.70	0.20	0.21	0.19	0.20
	40	0.87	0.86	0.88	0.87	0.23	0.22	0.24	0.23
	50	0.92	0.93	0.91	0.92	0.26	0.27	0.25	0.26
	100	1.10	1.11	1.09	1.10	0.36	0.37	0.35	0.36
	150	1.14	1.15	1.13	1.14	0.47	0.46	0.48	0.47
**AT 4**	0	0.00	0.01	0.00	0.00	0.00	0.01	0.00	0.00
	1	0.22	0.23	0.21	0.22	0.07	0.08	0.06	0.07
	10	0.58	0.59	0.57	0.58	0.20	0.21	0.19	0.20
	20	0.76	0.75	0.77	0.76	0.26	0.25	0.27	0.26
	30	0.88	0.87	0.89	0.88	0.30	0.29	0.31	0.30
	40	0.97	0.96	0.98	0.97	0.31	0.30	0.32	0.31
	50	0.99	1.00	0.98	0.99	0.35	0.36	0.34	0.35
	100	1.27	1.28	1.26	1.27	0.44	0.45	0.43	0.44
	150	1.50	1.49	1.51	1.50	0.52	0.53	0.51	0.52
**Prot.**	**hole nr.**	**Worn area (mm^2)**				**Cutting edge (mm)**			
		Run1	Run2	Run3	**AVG**	Run1	Run2	Run3	**AVG**
**PT1**	0	0.00	0.01	0.00	0.00	0.00	0.01	0.00	0.00
	1	0.05	0.06	0.04	0.05	0.01	0.02	0.00	0.01
	10	0.53	0.54	0.52	0.53	0.06	0.05	0.07	0.06
	20	0.78	0.79	0.77	0.78	0.08	0.09	0.07	0.08
	30	0.88	0.89	0.87	0.88	0.12	0.11	0.13	0.12
	40	0.93	0.92	0.94	0.93	0.15	0.14	0.16	0.15
	50	0.94	0.95	0.93	0.94	0.19	0.18	0.20	0.19
	100	1.00	1.01	0.99	1.00	0.27	0.26	0.28	0.27
	150	1.06	1.07	1.05	1.06	0.34	0.33	0.35	0.34
**PT2**	0	0.00	0.01	0.00	0.00	0.00	0.01	0.00	0.00
	1	0.05	0.04	0.06	0.05	0.01	0.02	0.01	0.01
	10	0.54	0.55	0.53	0.54	0.12	0.13	0.11	0.12
	20	0.78	0.77	0.79	0.78	0.18	0.19	0.17	0.18
	30	0.94	0.93	0.95	0.94	0.21	0.22	0.20	0.21

	40	1.03	1.02	1.04	1.03	0.22	0.23	0.21	0.22
	50	1.10	1.11	1.09	1.10	0.22	0.23	0.21	0.22
	100	1.40	1.39	1.41	1.40	0.28	0.29	0.27	0.28
	150	1.41	1.42	1.40	1.41	0.41	0.42	0.40	0.41
**PT3**	0	0.00	0.01	0.00	0.00	0.00	0.01	0.00	0.00
	1	0.07	0.08	0.06	0.07	0.02	0.03	0.01	0.02
	10	0.44	0.45	0.43	0.44	0.16	0.17	0.15	0.16
	20	0.62	0.63	0.61	0.62	0.22	0.23	0.21	0.22
	30	0.74	0.75	0.73	0.74	0.24	0.25	0.23	0.24
	40	0.81	0.82	0.80	0.81	0.24	0.25	0.23	0.24
	50	0.88	0.89	0.87	0.88	0.25	0.26	0.24	0.25
	100	1.08	1.09	1.07	1.08	0.37	0.38	0.36	0.37
	150	1.07	1.08	1.06	1.07	0.55	0.56	0.54	0.55
**PT4**	0	0.00	0.01	0.00	0.00	0.00	0.01	0.00	0.00
	1	0.10	0.11	0.12	0.11	0.05	0.06	0.04	0.05
	10	0.52	0.53	0.54	0.53	0.18	0.19	0.20	0.19
	20	0.77	0.78	0.79	0.78	0.23	0.24	0.25	0.24
	30	0.93	0.94	0.95	0.94	0.25	0.26	0.27	0.26
	40	1.03	1.04	1.05	1.04	0.26	0.27	0.28	0.27
	50	1.08	1.09	1.10	1.09	0.27	0.28	0.29	0.28
	100	1.24	1.25	1.26	1.25	0.48	0.49	0.50	0.49
	150	1.23	1.24	1.25	1.24	0.63	0.64	0.65	0.64
