# Supplementary material for: Impact of drill bit wear on temperature increase in dental implant osteotomy: an in vitro study
Source: PLoS One. 2025 Mar 19;20(3):e0319492. doi: 10.1371/journal.pone.0319492 (PMC11922234; doi:10.1371/journal.pone.0319492)
Supplement: S4 Table — reports the maximum temperature variations recorded during the drilling experiments. (PDF) [file pone.0319492.s004.pdf]

Prot.	Hole nr.	ΔT (K)				
		Run 1	Run 2	Run 3	**AVG**	**St. dev.**
**AT 1**	1	5.80	6.32	5.28	5.80	0.52
	10	5.42	5.99	4.85	5.42	0.57
	20	5.56	5.92	5.20	5.56	0.36
	30	5.95	6.46	5.44	5.95	0.51
	40	6.52	6.99	6.05	6.52	0.47
	50	6.46	6.82	6.10	6.46	0.36
**AT 2**	1	9.25	9.66	8.84	9.25	0.41
	10	9.73	10.20	9.26	9.73	0.47
	20	10.09	10.68	9.50	10.09	0.59
	30	11.95	12.24	11.66	11.95	0.29
	40	11.48	11.85	11.11	11.48	0.37
	50	11.54	12.02	11.06	11.54	0.48
**AT 3**	1	9.93	10.26	9.60	9.93	0.33
	10	11.21	11.78	10.64	11.21	0.57
	20	11.56	12.02	11.10	11.56	0.46
	30	14.78	15.16	14.40	14.78	0.38
	40	13.57	14.01	13.13	13.57	0.44
	50	12.34	12.55	12.13	12.34	0.21
**AT 4**	1	9.13	9.49	8.77	9.13	0.36
	10	10.12	10.53	9.71	10.12	0.41
	20	9.74	9.99	9.49	9.74	0.25
	30	14.34	14.76	13.92	14.34	0.42
	40	12.91	13.19	12.63	12.91	0.28
	50	14.04	14.56	13.52	14.04	0.52
**PT 1**	1	6.24	6.75	5.73	6.24	0.51
	10	6.48	6.95	6.01	6.48	0.47
	20	6.61	7.10	6.12	6.61	0.49
	30	7.12	7.73	6.51	7.12	0.61
	40	7.57	8.11	7.03	7.57	0.54
	50	6.72	7.27	6.17	6.72	0.55
**PT 2**	1	11.00	11.53	10.47	11.00	0.53
	10	11.61	12.08	11.14	11.61	0.47
	20	12.31	12.85	11.77	12.31	0.54
	30	12.97	13.36	12.58	12.97	0.39
	40	13.80	14.25	13.35	13.80	0.45
	50	12.24	12.71	11.77	12.24	0.47
**PT 3**	1	11.60	12.19	11.01	11.60	0.59
	10	12.43	12.94	11.92	12.43	0.51
	20	13.19	13.81	12.57	13.19	0.62
	30	14.24	14.60	13.88	14.24	0.36
	40	15.16	15.64	14.68	15.16	0.48
	50	15.00	15.51	14.49	15.00	0.51
**PT 4**	1	3.50	3.87	3.13	3.50	0.37
	10	5.50	6.11	4.89	5.50	0.61
	20	5.20	5.74	4.66	5.20	0.54
	30	6.64	7.10	6.18	6.64	0.46
	40	6.43	6.76	6.10	6.43	0.33
	50	6.09	6.69	5.49	6.09	0.60
