# Supplementary material for: Impact of drill bit wear on temperature increase in dental implant osteotomy: an in vitro study
Source: PLoS One. 2025 Mar 19;20(3):e0319492. doi: 10.1371/journal.pone.0319492 (PMC11922234; doi:10.1371/journal.pone.0319492)
Supplement: S5 Table — presents the measurements obtained using the CMM machine for holes numbered 1, 10, 20, 30, 40, and 50. Each measurement was repeated three times, and the mean value was calculated and reported. (PDF) [file pone.0319492.s005.pdf]

Protocol	AT			
Hole nr.	1			
Hole depth (mm)	Meas_1 Radius (mm)	Meas_2 Radius (mm)	Meas_3 Radius (mm)	**Mean Radius (mm)**
-13.990	0.188	0.235	0.220	**0.200**
-13.980	0.268	0.244	0.270	**0.248**
-13.970	0.332	0.347	0.343	**0.283**
-13.960	0.276	0.289	0.314	**0.324**
-13.950	0.352	0.346	0.381	**0.354**
-13.940	0.380	0.421	0.395	**0.384**
-13.930	0.421	0.391	0.428	**0.412**
-13.920	0.476	0.494	0.463	**0.439**
-13.910	0.423	0.442	0.456	**0.467**
-13.900	0.462	0.526	0.491	**0.497**
-13.890	0.563	0.559	0.540	**0.530**
-13.880	0.570	0.550	0.588	**0.559**
-13.870	0.554	0.584	0.585	**0.586**
-13.860	0.593	0.594	0.588	**0.613**
-13.850	0.662	0.698	0.684	**0.640**
-13.840	0.617	0.675	0.686	**0.666**
-13.830	0.656	0.684	0.717	**0.693**
-13.820	0.681	0.719	0.757	**0.719**
-13.810	0.736	0.782	0.759	**0.745**
-13.800	0.715	0.739	0.729	**0.773**
-13.790	0.820	0.819	0.818	**0.803**
-13.780	0.799	0.786	0.843	**0.827**
-13.770	0.814	0.845	0.817	**0.844**
-13.760	0.851	0.903	0.836	**0.857**
-13.750	0.915	0.857	0.869	**0.869**
-13.740	0.901	0.905	0.924	**0.882**
-13.730	0.855	0.912	0.867	**0.894**

Protocol	AT			
Hole nr.	10			
Hole depth (mm)	Meas_1 Radius (mm)	Meas_2 Radius (mm)	Meas_3 Radius (mm)	**Mean Radius (mm)**
-13.960	0.213	0.168	0.217	**0.218**
-13.950	0.266	0.298	0.276	**0.252**
-13.940	0.300	0.238	0.278	**0.281**
-13.930	0.278	0.356	0.299	**0.313**
-13.920	0.355	0.257	0.305	**0.344**
-13.910	0.328	0.368	0.378	**0.376**
-13.900	0.452	0.454	0.417	**0.410**
-13.890	0.472	0.380	0.424	**0.445**
-13.880	0.450	0.545	0.512	**0.477**
-13.870	0.535	0.551	0.550	**0.505**
-13.860	0.534	0.461	0.448	**0.532**
-13.850	0.560	0.641	0.600	**0.558**
-13.840	0.566	0.611	0.621	**0.585**
-13.830	0.640	0.618	0.565	**0.611**
-13.820	0.640	0.651	0.673	**0.638**
-13.810	0.698	0.665	0.688	**0.666**
-13.800	0.696	0.678	0.697	**0.696**
-13.790	0.753	0.758	0.739	**0.725**
-13.780	0.809	0.754	0.787	**0.755**
-13.770	0.778	0.801	0.765	**0.782**
-13.760	0.829	0.743	0.805	**0.804**
-13.750	0.782	0.816	0.860	**0.826**
-13.740	0.857	0.818	0.898	**0.848**
-13.730	0.848	0.857	0.875	**0.870**
-13.720	0.861	0.911	0.913	**0.889**
-13.710	0.859	0.920	0.845	**0.901**
-13.700	0.953	0.901	0.948	**0.912**

-13.720	0.914	0.962	0.890	**0.907**	-13.690	0.872	0.961	0.908	**0.922**
-13.710	0.947	0.880	0.902	**0.919**	-13.680	0.929	0.919	0.967	**0.933**
-13.700	0.916	0.895	0.955	**0.932**	-13.670	1.033	0.988	0.944	**0.943**
-13.690	0.979	0.914	0.971	**0.944**	-13.660	0.916	0.957	0.903	**0.953**
-13.680	0.919	0.982	0.926	**0.957**	-13.650	0.960	1.011	0.960	**0.964**
-13.670	0.936	1.012	0.954	**0.969**	-13.640	1.034	0.949	0.996	**0.974**
-13.660	0.933	0.982	0.940	**0.982**	-13.630	1.001	0.914	0.958	**0.984**
-13.650	0.916	0.977	0.920	**0.994**	-13.620	0.945	0.945	0.948	**0.994**
-13.640	0.996	0.996	1.013	**1.007**	-13.610	1.045	1.004	1.023	**1.004**
-13.630	0.972	1.026	1.027	**1.019**	-13.600	0.986	1.008	1.056	**1.014**
-13.620	0.996	1.006	1.040	**1.032**	-13.590	0.963	1.058	1.032	**1.025**
-13.610	1.008	1.049	1.072	**1.045**	-13.580	1.027	0.990	0.979	**1.035**
-13.600	1.047	1.009	1.066	**1.057**	-13.570	0.983	1.065	1.062	**1.045**
-13.590	1.068	1.042	1.052	**1.070**	-13.560	1.016	1.093	1.002	**1.055**
-13.580	1.117	1.080	1.075	**1.082**	-13.550	1.103	1.066	1.068	**1.065**
-13.570	1.063	1.102	1.049	**1.095**	-13.540	1.110	1.136	1.058	**1.075**
-13.560	1.085	1.142	1.081	**1.107**	-13.530	1.060	1.077	1.116	**1.084**
-13.550	1.095	1.110	1.115	**1.120**	-13.520	1.115	1.171	1.186	**1.094**
-13.540	1.139	1.150	1.134	**1.132**	-13.510	1.064	1.105	1.125	**1.103**
-13.530	1.104	1.127	1.129	**1.145**	-13.500	1.119	1.123	1.123	**1.113**
-13.520	1.136	1.163	1.177	**1.157**	-13.490	1.110	1.099	1.129	**1.122**
-13.510	1.224	1.178	1.164	**1.170**	-13.480	1.159	1.118	1.133	**1.132**
-13.500	1.198	1.127	1.190	**1.182**	-13.470	1.059	1.085	1.122	**1.141**
-13.490	1.173	1.122	1.165	**1.195**	-13.460	1.143	1.098	1.092	**1.151**
-13.480	1.198	1.206	1.185	**1.207**	-13.450	1.191	1.167	1.229	**1.160**
-13.470	1.188	1.180	1.210	**1.220**	-13.440	1.177	1.159	1.198	**1.170**
-13.460	1.225	1.233	1.290	**1.232**	-13.430	1.225	1.169	1.186	**1.179**
-13.450	1.286	1.285	1.275	**1.245**	-13.420	1.153	1.249	1.192	**1.189**
-13.440	1.278	1.246	1.234	**1.258**	-13.410	1.223	1.219	1.215	**1.198**
-13.430	1.281	1.295	1.296	**1.270**	-13.400	1.231	1.232	1.208	**1.208**
-13.420	1.220	1.262	1.299	**1.283**	-13.390	1.196	1.276	1.256	**1.217**
-13.410	1.300	1.286	1.291	**1.295**	-13.380	1.286	1.216	1.235	**1.227**

-13.400	1.266	1.316	1.271	**1.308**	-13.370	1.239	1.287	1.222	**1.236**
-13.390	1.329	1.312	1.355	**1.320**	-13.360	1.268	1.210	1.219	**1.246**
-13.380	1.275	1.325	1.340	**1.333**	-13.350	1.228	1.196	1.277	**1.255**
-13.370	1.319	1.350	1.372	**1.345**	-13.340	1.240	1.296	1.282	**1.265**
-13.360	1.361	1.288	1.345	**1.358**	-13.330	1.293	1.258	1.268	**1.274**
-13.350	1.356	1.338	1.372	**1.370**	-13.320	1.277	1.278	1.211	**1.284**
-13.340	1.367	1.412	1.367	**1.383**	-13.310	1.292	1.234	1.299	**1.293**
-13.330	1.343	1.383	1.352	**1.395**	-13.300	1.267	1.286	1.236	**1.302**
-13.320	1.473	1.437	1.432	**1.408**	-13.290	1.345	1.286	1.304	**1.312**
-13.310	1.462	1.422	1.405	**1.420**	-13.280	1.230	1.273	1.298	**1.321**
-13.300	1.448	1.428	1.448	**1.431**	-13.270	1.359	1.280	1.305	**1.331**
-13.290	1.431	1.413	1.467	**1.441**	-13.260	1.357	1.369	1.339	**1.340**
-13.280	1.526	1.480	1.489	**1.452**	-13.250	1.300	1.270	1.358	**1.350**
-13.270	1.424	1.490	1.476	**1.462**	-13.240	1.395	1.395	1.355	**1.359**
-13.260	1.522	1.514	1.469	**1.472**	-13.230	1.374	1.338	1.403	**1.369**
-13.250	1.503	1.509	1.445	**1.482**	-13.220	1.456	1.430	1.390	**1.378**
-13.240	1.522	1.484	1.493	**1.492**	-13.210	1.320	1.356	1.408	**1.388**
-13.230	1.537	1.520	1.551	**1.502**	-13.200	1.393	1.466	1.430	**1.397**
-13.220	1.485	1.489	1.496	**1.510**	-13.190	1.495	1.411	1.420	**1.407**
-13.210	1.512	1.527	1.492	**1.516**	-13.180	1.337	1.393	1.341	**1.416**
-13.200	1.612	1.537	1.585	**1.521**	-13.170	1.417	1.385	1.360	**1.424**
-13.190	1.494	1.482	1.479	**1.523**	-13.160	1.451	1.421	1.448	**1.431**
-13.180	1.552	1.550	1.497	**1.526**	-13.150	1.434	1.445	1.385	**1.438**
-13.170	1.560	1.600	1.558	**1.528**	-13.140	1.452	1.371	1.440	**1.445**
-13.160	1.560	1.523	1.502	**1.530**	-13.130	1.460	1.437	1.467	**1.451**
-13.150	1.546	1.484	1.521	**1.532**	-13.120	1.425	1.477	1.473	**1.456**
-13.140	1.502	1.518	1.485	**1.534**	-13.110	1.406	1.418	1.369	**1.460**
-13.130	1.584	1.524	1.593	**1.535**	-13.100	1.472	1.505	1.446	**1.465**
-13.120	1.528	1.536	1.497	**1.537**	-13.090	1.524	1.457	1.481	**1.469**
-13.110	1.537	1.587	1.551	**1.538**	-13.080	1.525	1.503	1.490	**1.474**
-13.100	1.485	1.503	1.509	**1.540**	-13.070	1.493	1.460	1.450	**1.478**
-13.090	1.525	1.548	1.547	**1.541**	-13.060	1.518	1.457	1.502	**1.482**

-13.080	1.514	1.553	1.499	**1.543**	-13.050	1.477	1.464	1.509	**1.486**
-13.070	1.576	1.546	1.596	**1.544**	-13.040	1.436	1.496	1.454	**1.490**
-13.060	1.540	1.558	1.532	**1.545**	-13.030	1.493	1.557	1.579	**1.494**
-13.050	1.539	1.578	1.566	**1.546**	-13.020	1.478	1.439	1.526	**1.498**
-13.040	1.530	1.509	1.559	**1.547**	-13.010	1.520	1.469	1.432	**1.502**
-13.030	1.578	1.590	1.649	**1.548**	-13.000	1.371	1.458	1.434	**1.506**
-13.020	1.594	1.569	1.573	**1.550**	-12.990	1.478	1.459	1.539	**1.511**
-13.010	1.583	1.574	1.539	**1.551**	-12.980	1.488	1.545	1.572	**1.515**
-13.000	1.480	1.551	1.491	**1.552**	-12.970	1.579	1.543	1.498	**1.519**
-12.990	1.543	1.530	1.543	**1.553**	-12.960	1.478	1.426	1.482	**1.523**
-12.980	1.614	1.598	1.629	**1.554**	-12.950	1.600	1.540	1.509	**1.527**
-12.970	1.579	1.567	1.509	**1.555**	-12.940	1.540	1.549	1.552	**1.530**
-12.960	1.563	1.525	1.552	**1.556**	-12.930	1.559	1.520	1.532	**1.534**
-12.950	1.529	1.514	1.579	**1.557**	-12.920	1.524	1.472	1.494	**1.537**
-12.940	1.611	1.545	1.584	**1.558**	-12.910	1.584	1.577	1.495	**1.539**
-12.930	1.568	1.582	1.595	**1.559**	-12.900	1.549	1.519	1.582	**1.541**
-12.920	1.599	1.596	1.614	**1.560**	-12.890	1.558	1.467	1.507	**1.543**
-12.910	1.578	1.591	1.543	**1.561**	-12.880	1.561	1.473	1.497	**1.545**
-12.900	1.542	1.565	1.603	**1.561**	-12.870	1.563	1.541	1.579	**1.546**
-12.890	1.568	1.547	1.556	**1.561**	-12.860	1.570	1.541	1.548	**1.547**
-12.880	1.550	1.545	1.523	**1.561**	-12.850	1.504	1.501	1.570	**1.548**
-12.870	1.588	1.546	1.575	**1.561**	-12.840	1.628	1.533	1.605	**1.550**
-12.860	1.598	1.574	1.559	**1.561**	-12.830	1.620	1.605	1.627	**1.551**
-12.850	1.537	1.546	1.537	**1.561**	-12.820	1.570	1.471	1.511	**1.552**
-12.840	1.540	1.567	1.562	**1.561**	-12.810	1.546	1.481	1.528	**1.554**
-12.830	1.590	1.512	1.546	**1.562**	-12.800	1.587	1.493	1.511	**1.555**
-12.820	1.576	1.527	1.581	**1.562**	-12.790	1.573	1.583	1.533	**1.557**
-12.810	1.539	1.554	1.580	**1.562**	-12.780	1.572	1.524	1.587	**1.558**
-12.800	1.495	1.524	1.542	**1.562**	-12.770	1.524	1.572	1.577	**1.559**
-12.790	1.571	1.551	1.554	**1.563**	-12.760	1.644	1.561	1.635	**1.561**
-12.780	1.493	1.551	1.508	**1.563**	-12.750	1.554	1.518	1.557	**1.562**
-12.770	1.569	1.533	1.542	**1.563**	-12.740	1.477	1.523	1.533	**1.564**

-12.760	1.579	1.578	1.606	**1.563**	-12.730	1.634	1.576	1.667	**1.565**
-12.750	1.537	1.553	1.576	**1.563**	-12.720	1.588	1.584	1.555	**1.566**
-12.740	1.524	1.510	1.498	**1.563**	-12.710	1.523	1.577	1.549	**1.568**
-12.730	1.584	1.542	1.518	**1.563**	-12.700	1.582	1.543	1.603	**1.569**
-12.720	1.552	1.491	1.511	**1.563**	-12.690	1.540	1.588	1.603	**1.570**
-12.710	1.507	1.558	1.531	**1.563**	-12.680	1.565	1.525	1.583	**1.571**
-12.700	1.553	1.577	1.609	**1.563**	-12.670	1.579	1.590	1.553	**1.572**
-12.690	1.609	1.543	1.553	**1.563**	-12.660	1.589	1.544	1.585	**1.572**
-12.680	1.570	1.626	1.596	**1.563**	-12.650	1.567	1.594	1.559	**1.572**
-12.670	1.494	1.531	1.515	**1.563**	-12.640	1.654	1.587	1.599	**1.572**
-12.660	1.522	1.589	1.541	**1.563**	-12.630	1.616	1.532	1.584	**1.572**
-12.650	1.624	1.581	1.573	**1.563**	-12.620	1.525	1.553	1.570	**1.572**
-12.640	1.585	1.534	1.568	**1.563**	-12.610	1.602	1.518	1.524	**1.572**
-12.630	1.509	1.509	1.526	**1.564**	-12.600	1.594	1.604	1.518	**1.571**
-12.620	1.523	1.595	1.587	**1.564**	-12.590	1.570	1.551	1.555	**1.571**
-12.610	1.589	1.516	1.549	**1.564**	-12.580	1.462	1.497	1.532	**1.571**
-12.600	1.556	1.586	1.599	**1.564**	-12.570	1.483	1.485	1.557	**1.571**
-12.590	1.600	1.599	1.572	**1.564**	-12.560	1.549	1.593	1.580	**1.570**
-12.580	1.587	1.618	1.551	**1.564**	-12.550	1.537	1.562	1.590	**1.570**
-12.570	1.529	1.582	1.579	**1.564**	-12.540	1.601	1.603	1.553	**1.570**
-12.560	1.580	1.568	1.587	**1.564**	-12.530	1.527	1.533	1.504	**1.569**
-12.550	1.580	1.529	1.505	**1.564**	-12.520	1.559	1.604	1.579	**1.569**
-12.540	1.578	1.567	1.580	**1.564**	-12.510	1.592	1.550	1.560	**1.569**
-12.530	1.501	1.534	1.568	**1.564**	-12.500	1.530	1.565	1.540	**1.569**
-12.520	1.569	1.571	1.528	**1.565**	-12.490	1.533	1.470	1.492	**1.569**
-12.510	1.527	1.538	1.563	**1.565**	-12.480	1.594	1.548	1.520	**1.570**
-12.500	1.610	1.612	1.566	**1.565**	-12.470	1.606	1.560	1.573	**1.569**
-12.490	1.532	1.556	1.557	**1.566**	-12.460	1.573	1.578	1.608	**1.569**
-12.480	1.593	1.580	1.599	**1.566**	-12.450	1.552	1.528	1.535	**1.568**
-12.470	1.567	1.593	1.566	**1.566**	-12.440	1.562	1.573	1.533	**1.568**
-12.460	1.601	1.574	1.608	**1.566**	-12.430	1.550	1.613	1.565	**1.568**
-12.450	1.582	1.625	1.561	**1.567**	-12.420	1.594	1.601	1.619	**1.568**

-12.440	1.525	1.537	1.551	**1.567**	-12.410	1.540	1.530	1.586	**1.567**
-12.430	1.595	1.589	1.573	**1.567**	-12.400	1.626	1.593	1.573	**1.567**
-12.420	1.592	1.633	1.575	**1.567**	-12.390	1.555	1.558	1.611	**1.567**
-12.410	1.554	1.594	1.540	**1.567**	-12.380	1.647	1.621	1.615	**1.567**
-12.400	1.578	1.532	1.554	**1.567**	-12.370	1.565	1.479	1.556	**1.566**
-12.390	1.611	1.577	1.562	**1.566**	-12.360	1.604	1.600	1.646	**1.566**
-12.380	1.547	1.556	1.571	**1.566**	-12.350	1.627	1.589	1.538	**1.566**
-12.370	1.582	1.542	1.530	**1.566**	-12.340	1.551	1.591	1.541	**1.565**
-12.360	1.572	1.577	1.571	**1.566**	-12.330	1.541	1.545	1.558	**1.565**
-12.350	1.545	1.504	1.563	**1.566**	-12.320	1.567	1.514	1.564	**1.565**
-12.340	1.605	1.642	1.591	**1.566**	-12.310	1.454	1.517	1.544	**1.564**
-12.330	1.583	1.589	1.574	**1.566**	-12.300	1.578	1.578	1.627	**1.564**
-12.320	1.538	1.546	1.524	**1.566**	-12.290	1.590	1.626	1.619	**1.563**
-12.310	1.564	1.577	1.604	**1.565**	-12.280	1.568	1.490	1.499	**1.563**
-12.300	1.554	1.532	1.583	**1.565**	-12.270	1.545	1.589	1.534	**1.563**
-12.290	1.513	1.528	1.506	**1.565**	-12.260	1.566	1.597	1.529	**1.563**
-12.280	1.572	1.583	1.530	**1.565**	-12.250	1.527	1.578	1.510	**1.562**
-12.270	1.551	1.586	1.569	**1.565**	-12.240	1.585	1.538	1.485	**1.562**
-12.260	1.549	1.563	1.553	**1.565**	-12.230	1.538	1.534	1.572	**1.562**
-12.250	1.570	1.574	1.576	**1.564**	-12.220	1.581	1.535	1.610	**1.562**
-12.240	1.574	1.542	1.576	**1.564**	-12.210	1.546	1.586	1.615	**1.562**
-12.230	1.573	1.512	1.537	**1.564**	-12.200	1.574	1.638	1.549	**1.562**
-12.220	1.601	1.609	1.588	**1.564**	-12.190	1.567	1.596	1.531	**1.562**
-12.210	1.569	1.555	1.590	**1.564**	-12.180	1.511	1.551	1.473	**1.562**
-12.200	1.555	1.519	1.548	**1.564**	-12.170	1.565	1.586	1.550	**1.562**
-12.190	1.555	1.500	1.572	**1.564**	-12.160	1.589	1.600	1.596	**1.562**
-12.180	1.540	1.515	1.493	**1.563**	-12.150	1.552	1.575	1.534	**1.562**
-12.170	1.596	1.613	1.571	**1.563**	-12.140	1.531	1.527	1.498	**1.562**
-12.160	1.536	1.579	1.562	**1.562**	-12.130	1.628	1.534	1.562	**1.562**
-12.150	1.565	1.513	1.531	**1.562**	-12.120	1.598	1.577	1.530	**1.562**
-12.140	1.583	1.583	1.581	**1.563**	-12.110	1.616	1.574	1.608	**1.562**
-12.130	1.593	1.533	1.574	**1.563**	-12.100	1.578	1.635	1.637	**1.562**

-12.120	1.550	1.578	1.525	**1.563**	-12.090	1.533	1.563	1.592	**1.562**
-12.110	1.555	1.577	1.578	**1.563**	-12.080	1.627	1.570	1.537	**1.563**
-12.100	1.540	1.607	1.576	**1.563**	-12.070	1.562	1.496	1.536	**1.563**
-12.090	1.582	1.587	1.585	**1.564**	-12.060	1.520	1.572	1.551	**1.563**
-12.080	1.599	1.563	1.556	**1.564**	-12.050	1.585	1.513	1.573	**1.563**
-12.070	1.518	1.564	1.583	**1.563**	-12.040	1.544	1.606	1.537	**1.564**
-12.060	1.610	1.531	1.579	**1.563**	-12.030	1.490	1.562	1.534	**1.564**
-12.050	1.590	1.604	1.555	**1.563**	-12.020	1.612	1.533	1.605	**1.565**
-12.040	1.517	1.543	1.528	**1.562**	-12.010	1.527	1.619	1.584	**1.565**
-12.030	1.559	1.494	1.534	**1.562**	-12.000	1.507	1.508	1.569	**1.566**
-12.020	1.536	1.591	1.599	**1.562**	-11.990	1.599	1.509	1.578	**1.566**
-12.010	1.581	1.534	1.537	**1.562**	-11.980	1.678	1.629	1.593	**1.566**
-12.000	1.593	1.514	1.562	**1.562**	-11.970	1.611	1.634	1.577	**1.567**
-11.990	1.551	1.592	1.616	**1.562**	-11.960	1.562	1.554	1.575	**1.567**
-11.980	1.609	1.566	1.553	**1.562**	-11.950	1.555	1.529	1.521	**1.567**
-11.970	1.564	1.560	1.583	**1.562**	-11.940	1.598	1.540	1.521	**1.568**
-11.960	1.529	1.544	1.510	**1.562**	-11.930	1.550	1.599	1.629	**1.568**
-11.950	1.543	1.596	1.526	**1.561**	-11.920	1.551	1.590	1.553	**1.568**
-11.940	1.556	1.530	1.578	**1.561**	-11.910	1.624	1.548	1.547	**1.568**
-11.930	1.583	1.538	1.526	**1.561**	-11.900	1.601	1.567	1.534	**1.568**
-11.920	1.567	1.526	1.560	**1.561**	-11.890	1.460	1.498	1.541	**1.568**
-11.910	1.563	1.564	1.598	**1.560**	-11.880	1.606	1.602	1.594	**1.567**
-11.900	1.565	1.565	1.618	**1.560**	-11.870	1.618	1.557	1.586	**1.567**
-11.890	1.564	1.529	1.593	**1.560**	-11.860	1.564	1.590	1.558	**1.567**
-11.880	1.573	1.512	1.585	**1.560**	-11.850	1.554	1.505	1.510	**1.567**
-11.870	1.592	1.522	1.537	**1.559**	-11.840	1.587	1.525	1.614	**1.566**
-11.860	1.616	1.546	1.596	**1.559**	-11.830	1.580	1.631	1.614	**1.566**
-11.850	1.620	1.598	1.616	**1.559**	-11.820	1.577	1.634	1.560	**1.566**
-11.840	1.592	1.568	1.570	**1.558**	-11.810	1.603	1.547	1.545	**1.565**
-11.830	1.607	1.561	1.585	**1.558**	-11.800	1.511	1.589	1.567	**1.565**
-11.820	1.525	1.567	1.583	**1.558**	-11.790	1.488	1.560	1.554	**1.565**
-11.810	1.562	1.542	1.600	**1.557**	-11.780	1.591	1.534	1.498	**1.565**

-11.800	1.549	1.585	1.588	**1.558**	-11.770	1.561	1.590	1.583	**1.565**
-11.790	1.535	1.587	1.519	**1.558**	-11.760	1.598	1.518	1.522	**1.565**
-11.780	1.598	1.616	1.593	**1.558**	-11.750	1.531	1.595	1.578	**1.564**
-11.770	1.553	1.558	1.534	**1.559**	-11.740	1.582	1.615	1.598	**1.564**
-11.760	1.543	1.585	1.563	**1.559**	-11.730	1.629	1.635	1.563	**1.564**
-11.750	1.566	1.595	1.583	**1.560**	-11.720	1.544	1.602	1.601	**1.564**
-11.740	1.569	1.599	1.563	**1.561**	-11.710	1.558	1.536	1.577	**1.564**
-11.730	1.551	1.513	1.568	**1.561**	-11.700	1.581	1.563	1.600	**1.564**
-11.720	1.542	1.594	1.608	**1.561**	-11.690	1.564	1.560	1.602	**1.564**
-11.710	1.557	1.600	1.547	**1.562**	-11.680	1.574	1.537	1.504	**1.564**
-11.700	1.540	1.502	1.542	**1.562**	-11.670	1.597	1.603	1.574	**1.564**
-11.690	1.536	1.541	1.595	**1.562**	-11.660	1.569	1.560	1.558	**1.564**
-11.680	1.561	1.568	1.567	**1.563**	-11.650	1.555	1.637	1.623	**1.565**
-11.670	1.589	1.559	1.550	**1.563**	-11.640	1.546	1.602	1.536	**1.565**
-11.660	1.554	1.576	1.576	**1.563**	-11.630	1.529	1.559	1.575	**1.565**
-11.650	1.591	1.530	1.522	**1.563**	-11.620	1.543	1.566	1.594	**1.565**
-11.640	1.593	1.548	1.532	**1.563**	-11.610	1.569	1.566	1.583	**1.565**
-11.630	1.514	1.519	1.545	**1.563**	-11.600	1.592	1.536	1.583	**1.565**
-11.620	1.620	1.610	1.564	**1.563**	-11.590	1.532	1.554	1.620	**1.565**
-11.610	1.543	1.602	1.561	**1.563**	-11.580	1.591	1.528	1.587	**1.564**
-11.600	1.549	1.523	1.525	**1.563**	-11.570	1.515	1.579	1.536	**1.564**
-11.590	1.522	1.585	1.530	**1.563**	-11.560	1.574	1.523	1.585	**1.564**
-11.580	1.537	1.566	1.505	**1.563**	-11.550	1.539	1.521	1.454	**1.564**
-11.570	1.536	1.588	1.576	**1.563**	-11.540	1.568	1.544	1.599	**1.564**
-11.560	1.541	1.560	1.532	**1.563**	-11.530	1.521	1.571	1.578	**1.564**
-11.550	1.530	1.560	1.569	**1.563**	-11.520	1.569	1.577	1.542	**1.563**
-11.540	1.618	1.570	1.565	**1.563**	-11.510	1.489	1.580	1.516	**1.563**
-11.530	1.594	1.546	1.536	**1.563**	-11.500	1.619	1.548	1.606	**1.563**
-11.520	1.571	1.589	1.602	**1.563**	-11.490	1.619	1.579	1.565	**1.563**
-11.510	1.515	1.550	1.485	**1.563**	-11.480	1.570	1.526	1.616	**1.563**
-11.500	1.552	1.530	1.577	**1.563**	-11.470	1.540	1.619	1.539	**1.563**
-11.490	1.578	1.599	1.614	**1.562**	-11.460	1.555	1.627	1.548	**1.564**

-11.480	1.534	1.537	1.523	**1.562**	-11.450	1.601	1.570	1.590	**1.564**
-11.470	1.545	1.604	1.590	**1.562**	-11.440	1.590	1.555	1.584	**1.564**
-11.460	1.535	1.574	1.577	**1.562**	-11.430	1.564	1.600	1.596	**1.565**
-11.450	1.572	1.517	1.556	**1.562**	-11.420	1.494	1.538	1.504	**1.565**
-11.440	1.596	1.555	1.586	**1.561**	-11.410	1.618	1.587	1.636	**1.565**
-11.430	1.545	1.585	1.557	**1.561**	-11.400	1.564	1.538	1.473	**1.565**
-11.420	1.584	1.532	1.570	**1.561**	-11.390	1.592	1.546	1.549	**1.565**
-11.410	1.533	1.507	1.533	**1.561**	-11.380	1.533	1.602	1.589	**1.566**
-11.400	1.541	1.590	1.517	**1.561**	-11.370	1.541	1.507	1.605	**1.566**
-11.390	1.564	1.554	1.512	**1.561**	-11.360	1.563	1.584	1.584	**1.567**
-11.380	1.562	1.520	1.596	**1.561**	-11.350	1.563	1.605	1.580	**1.569**
-11.370	1.556	1.595	1.538	**1.560**	-11.340	1.574	1.519	1.574	**1.572**
-11.360	1.568	1.504	1.527	**1.560**	-11.330	1.621	1.555	1.595	**1.575**
-11.350	1.588	1.581	1.608	**1.561**	-11.320	1.568	1.557	1.501	**1.578**
-11.340	1.580	1.567	1.589	**1.561**	-11.310	1.590	1.597	1.563	**1.581**
-11.330	1.592	1.605	1.623	**1.562**	-11.300	1.582	1.579	1.569	**1.584**
-11.320	1.534	1.527	1.538	**1.563**	-11.290	1.544	1.551	1.541	**1.587**
-11.310	1.501	1.548	1.557	**1.564**	-11.280	1.625	1.589	1.606	**1.590**
-11.300	1.548	1.611	1.557	**1.565**	-11.270	1.608	1.601	1.595	**1.593**
-11.290	1.594	1.646	1.624	**1.567**	-11.260	1.618	1.669	1.582	**1.596**
-11.280	1.613	1.596	1.637	**1.569**	-11.250	1.584	1.526	1.614	**1.599**
-11.270	1.625	1.557	1.576	**1.570**	-11.240	1.572	1.607	1.658	**1.602**
-11.260	1.535	1.564	1.602	**1.572**	-11.230	1.633	1.601	1.563	**1.605**
-11.250	1.610	1.617	1.583	**1.574**	-11.220	1.613	1.637	1.574	**1.610**
-11.240	1.619	1.601	1.602	**1.576**	-11.210	1.626	1.628	1.680	**1.614**
-11.230	1.578	1.565	1.525	**1.579**	-11.200	1.586	1.552	1.588	**1.618**
-11.220	1.575	1.554	1.551	**1.581**	-11.190	1.599	1.639	1.612	**1.623**
-11.210	1.566	1.597	1.525	**1.583**	-11.180	1.559	1.610	1.532	**1.627**
-11.200	1.597	1.577	1.521	**1.585**	-11.170	1.577	1.671	1.641	**1.631**
-11.190	1.617	1.601	1.568	**1.588**	-11.160	1.646	1.633	1.597	**1.635**
-11.180	1.623	1.623	1.596	**1.591**	-11.150	1.612	1.597	1.643	**1.640**
-11.170	1.630	1.605	1.620	**1.594**	-11.140	1.617	1.566	1.588	**1.644**

-11.160	1.580	1.625	1.582	**1.598**	-11.130	1.675	1.650	1.646	**1.648**
-11.150	1.619	1.623	1.575	**1.601**	-11.120	1.695	1.756	1.700	**1.652**
-11.140	1.631	1.611	1.602	**1.605**	-11.110	1.640	1.672	1.704	**1.657**
-11.130	1.647	1.603	1.569	**1.608**	-11.100	1.665	1.735	1.723	**1.661**
-11.120	1.634	1.640	1.578	**1.613**	-11.090	1.654	1.729	1.718	**1.665**
-11.110	1.605	1.667	1.676	**1.617**	-11.080	1.679	1.645	1.651	**1.670**
-11.100	1.587	1.593	1.635	**1.622**	-11.070	1.656	1.708	1.725	**1.674**
-11.090	1.662	1.613	1.590	**1.626**	-11.060	1.728	1.684	1.643	**1.678**
-11.080	1.635	1.641	1.598	**1.631**	-11.050	1.672	1.675	1.682	**1.682**
-11.070	1.687	1.697	1.656	**1.635**	-11.040	1.694	1.680	1.665	**1.687**
-11.060	1.588	1.656	1.626	**1.640**	-11.030	1.753	1.763	1.718	**1.691**
-11.050	1.658	1.683	1.662	**1.644**	-11.020	1.699	1.766	1.677	**1.695**
-11.040	1.641	1.712	1.658	**1.649**	-11.010	1.684	1.702	1.749	**1.699**
-11.030	1.651	1.631	1.698	**1.653**	-11.000	1.699	1.732	1.699	**1.704**
-11.020	1.621	1.606	1.648	**1.658**	-10.990	1.791	1.711	1.755	**1.708**
-11.010	1.662	1.645	1.632	**1.662**	-10.980	1.742	1.733	1.771	**1.712**
-11.000	1.693	1.639	1.681	**1.667**	-10.970	1.699	1.726	1.727	**1.716**
-10.990	1.679	1.622	1.606	**1.671**	-10.960	1.738	1.693	1.692	**1.721**
-10.980	1.727	1.710	1.679	**1.676**	-10.950	1.668	1.716	1.760	**1.725**
-10.970	1.659	1.630	1.658	**1.680**	-10.940	1.686	1.691	1.756	**1.729**
-10.960	1.740	1.670	1.703	**1.685**	-10.930	1.750	1.762	1.769	**1.734**
-10.950	1.694	1.637	1.622	**1.689**	-10.920	1.709	1.773	1.682	**1.738**
-10.940	1.684	1.716	1.696	**1.694**	-10.910	1.811	1.785	1.773	**1.742**
-10.930	1.674	1.667	1.660	**1.698**	-10.900	1.752	1.680	1.746	**1.746**
-10.920	1.693	1.673	1.696	**1.703**	-10.890	1.760	1.775	1.704	**1.750**
-10.910	1.635	1.662	1.712	**1.707**	-10.880	1.758	1.762	1.707	**1.753**
-10.900	1.721	1.711	1.755	**1.712**	-10.870	1.868	1.826	1.818	**1.757**
-10.890	1.689	1.690	1.649	**1.716**	-10.860	1.739	1.742	1.772	**1.761**
-10.880	1.659	1.731	1.717	**1.721**	-10.850	1.782	1.870	1.836	**1.765**
-10.870	1.761	1.710	1.748	**1.725**	-10.840	1.817	1.736	1.823	**1.769**
-10.860	1.723	1.734	1.733	**1.730**	-10.830	1.763	1.759	1.800	**1.773**
-10.850	1.709	1.763	1.780	**1.734**	-10.820	1.735	1.820	1.764	**1.777**

-10.840	1.703	1.697	1.726	**1.739**	-10.810	1.853	1.755	1.812	**1.781**
-10.830	1.749	1.745	1.736	**1.743**	-10.800	1.810	1.805	1.737	**1.785**
-10.820	1.758	1.712	1.715	**1.748**	-10.790	1.791	1.782	1.759	**1.788**
-10.810	1.764	1.742	1.733	**1.752**	-10.780	1.777	1.768	1.777	**1.792**
-10.800	1.704	1.752	1.759	**1.757**	-10.770	1.753	1.735	1.764	**1.796**
-10.790	1.792	1.730	1.734	**1.761**	-10.760	1.785	1.849	1.770	**1.799**
-10.780	1.737	1.699	1.773	**1.766**	-10.750	1.795	1.827	1.837	**1.803**
-10.770	1.783	1.738	1.732	**1.770**	-10.740	1.787	1.847	1.787	**1.806**
-10.760	1.741	1.767	1.817	**1.775**	-10.730	1.854	1.760	1.814	**1.810**
-10.750	1.841	1.781	1.806	**1.779**	-10.720	1.799	1.774	1.817	**1.813**
-10.740	1.788	1.799	1.765	**1.784**	-10.710	1.743	1.735	1.744	**1.817**
-10.730	1.774	1.767	1.732	**1.788**	-10.700	1.781	1.863	1.824	**1.821**
-10.720	1.769	1.766	1.775	**1.793**	-10.690	1.886	1.873	1.800	**1.824**
-10.710	1.812	1.812	1.847	**1.798**	-10.680	1.760	1.829	1.811	**1.828**
-10.700	1.835	1.840	1.807	**1.802**	-10.670	1.844	1.854	1.795	**1.831**
-10.690	1.825	1.853	1.814	**1.807**	-10.660	1.784	1.826	1.836	**1.835**
-10.680	1.748	1.817	1.772	**1.811**	-10.650	1.766	1.845	1.801	**1.839**
-10.670	1.817	1.801	1.792	**1.816**	-10.640	1.851	1.883	1.886	**1.842**
-10.660	1.865	1.805	1.820	**1.820**	-10.630	1.890	1.854	1.848	**1.845**
-10.650	1.814	1.852	1.831	**1.823**	-10.620	1.847	1.780	1.783	**1.847**
-10.640	1.809	1.804	1.781	**1.826**	-10.610	1.784	1.864	1.871	**1.849**
-10.630	1.876	1.868	1.863	**1.829**	-10.600	1.875	1.820	1.832	**1.850**
-10.620	1.833	1.851	1.816	**1.831**	-10.590	1.829	1.859	1.877	**1.851**
-10.610	1.761	1.828	1.819	**1.833**	-10.580	1.843	1.910	1.832	**1.852**
-10.600	1.791	1.853	1.789	**1.835**	-10.570	1.900	1.817	1.811	**1.854**
-10.590	1.797	1.849	1.813	**1.837**	-10.560	1.946	1.898	1.883	**1.855**
-10.580	1.799	1.803	1.845	**1.839**	-10.550	1.856	1.826	1.915	**1.856**
-10.570	1.868	1.823	1.811	**1.841**	-10.540	1.838	1.930	1.876	**1.856**
-10.560	1.879	1.846	1.853	**1.843**	-10.530	1.921	1.827	1.880	**1.856**
-10.550	1.854	1.849	1.806	**1.845**	-10.520	1.858	1.818	1.829	**1.856**
-10.540	1.804	1.871	1.828	**1.847**	-10.510	1.817	1.882	1.804	**1.856**
-10.530	1.842	1.911	1.860	**1.849**	-10.500	1.886	1.841	1.844	**1.856**

-10.520	1.849	1.898	1.880	**1.851**	-10.490	1.812	1.814	1.822	**1.856**
-10.510	1.841	1.850	1.813	**1.853**	-10.480	1.809	1.820	1.823	**1.855**
-10.500	1.878	1.818	1.863	**1.854**	-10.470	1.779	1.768	1.699	**1.855**
-10.490	1.865	1.876	1.849	**1.856**	-10.460	1.882	1.806	1.866	**1.855**
-10.480	1.874	1.875	1.873	**1.858**	-10.450	1.835	1.897	1.874	**1.855**
-10.470	1.856	1.825	1.847	**1.859**	-10.440	1.928	1.866	1.881	**1.854**
-10.460	1.899	1.824	1.877	**1.861**	-10.430	1.835	1.808	1.768	**1.854**
-10.450	1.852	1.869	1.843	**1.862**	-10.420	1.880	1.857	1.889	**1.854**
-10.440	1.909	1.921	1.863	**1.863**	-10.410	1.843	1.840	1.886	**1.854**
-10.430	1.866	1.846	1.856	**1.863**	-10.400	1.838	1.866	1.925	**1.854**
-10.420	1.844	1.858	1.805	**1.863**	-10.390	1.885	1.824	1.840	**1.854**
-10.410	1.841	1.888	1.847	**1.863**	-10.380	1.932	1.856	1.865	**1.855**
-10.400	1.888	1.869	1.848	**1.863**	-10.370	1.903	1.970	1.903	**1.855**
-10.390	1.855	1.820	1.885	**1.864**	-10.360	1.827	1.855	1.821	**1.855**
-10.380	1.901	1.888	1.875	**1.864**	-10.350	1.881	1.865	1.907	**1.855**
-10.370	1.832	1.794	1.840	**1.864**	-10.340	1.865	1.808	1.810	**1.855**
-10.360	1.910	1.885	1.843	**1.864**	-10.330	1.834	1.839	1.828	**1.855**
-10.350	1.821	1.818	1.824	**1.864**	-10.320	1.746	1.793	1.835	**1.856**
-10.340	1.850	1.844	1.828	**1.864**	-10.310	1.873	1.815	1.796	**1.857**
-10.330	1.816	1.857	1.869	**1.864**	-10.300	1.802	1.897	1.871	**1.857**
-10.320	1.853	1.786	1.794	**1.865**	-10.290	1.865	1.897	1.901	**1.858**
-10.310	1.877	1.845	1.815	**1.865**	-10.280	1.828	1.773	1.786	**1.859**
-10.300	1.956	1.904	1.885	**1.865**	-10.270	1.838	1.856	1.865	**1.859**
-10.290	1.823	1.893	1.881	**1.865**	-10.260	1.857	1.818	1.915	**1.860**
-10.280	1.884	1.857	1.932	**1.865**	-10.250	1.835	1.813	1.762	**1.860**
-10.270	1.883	1.853	1.868	**1.865**	-10.240	1.831	1.825	1.879	**1.861**
-10.260	1.912	1.869	1.928	**1.865**	-10.230	1.869	1.896	1.836	**1.861**
-10.250	1.824	1.842	1.850	**1.865**	-10.220	1.890	1.827	1.903	**1.862**
-10.240	1.879	1.877	1.898	**1.864**	-10.210	1.895	1.877	1.809	**1.862**
-10.230	1.884	1.873	1.888	**1.864**	-10.200	1.781	1.833	1.869	**1.863**
-10.220	1.847	1.857	1.789	**1.864**	-10.190	1.840	1.856	1.812	**1.863**
-10.210	1.880	1.812	1.840	**1.864**	-10.180	1.861	1.820	1.916	**1.864**

-10.200	1.841	1.910	1.866	**1.864**	-10.170	1.903	1.876	1.850	**1.864**
-10.190	1.836	1.863	1.845	**1.864**	-10.160	1.899	1.867	1.931	**1.865**
-10.180	1.776	1.851	1.789	**1.864**	-10.150	1.854	1.936	1.893	**1.865**
-10.170	1.835	1.840	1.846	**1.864**	-10.140	1.866	1.879	1.821	**1.866**
-10.160	1.871	1.863	1.892	**1.863**	-10.130	1.891	1.880	1.874	**1.866**
-10.150	1.854	1.904	1.893	**1.863**	-10.120	1.847	1.929	1.908	**1.866**
-10.140	1.880	1.865	1.913	**1.863**	-10.110	1.805	1.841	1.888	**1.867**
-10.130	1.808	1.824	1.848	**1.863**	-10.100	1.893	1.903	1.884	**1.867**
-10.120	1.855	1.853	1.840	**1.863**	-10.090	1.860	1.804	1.818	**1.867**
-10.110	1.826	1.799	1.795	**1.862**	-10.080	1.851	1.877	1.907	**1.867**
-10.100	1.863	1.879	1.817	**1.862**	-10.070	1.834	1.905	1.858	**1.867**
-10.090	1.870	1.860	1.893	**1.862**	-10.060	1.852	1.884	1.858	**1.867**
-10.080	1.868	1.861	1.822	**1.862**	-10.050	1.868	1.847	1.794	**1.867**
-10.070	1.884	1.882	1.894	**1.862**	-10.040	1.870	1.924	1.854	**1.867**
-10.060	1.859	1.896	1.836	**1.861**	-10.030	1.826	1.839	1.874	**1.867**
-10.050	1.886	1.810	1.850	**1.861**	-10.020	1.855	1.872	1.879	**1.868**
-10.040	1.874	1.849	1.923	**1.861**	-10.010	1.817	1.834	1.883	**1.868**
-10.030	1.858	1.847	1.838	**1.860**	-10.000	1.849	1.896	1.799	**1.868**
-10.020	1.895	1.853	1.926	**1.860**	-9.990	1.925	1.880	1.884	**1.867**
-10.010	1.817	1.819	1.846	**1.860**	-9.980	1.806	1.837	1.875	**1.867**
-10.000	1.801	1.814	1.866	**1.859**	-9.970	1.829	1.908	1.835	**1.867**
-9.990	1.902	1.905	1.881	**1.859**	-9.960	1.827	1.889	1.821	**1.866**
-9.980	1.857	1.913	1.927	**1.859**	-9.950	1.936	1.841	1.899	**1.866**
-9.970	1.796	1.837	1.839	**1.859**	-9.940	1.850	1.800	1.894	**1.865**
-9.960	1.880	1.889	1.832	**1.859**	-9.930	1.928	1.886	1.857	**1.865**
-9.950	1.846	1.847	1.881	**1.859**	-9.920	1.876	1.841	1.797	**1.865**
-9.940	1.850	1.826	1.810	**1.860**	-9.910	1.758	1.778	1.841	**1.864**
-9.930	1.873	1.874	1.828	**1.860**	-9.900	1.898	1.859	1.871	**1.864**
-9.920	1.875	1.867	1.872	**1.860**	-9.890	1.841	1.880	1.905	**1.864**
-9.910	1.860	1.876	1.816	**1.861**	-9.880	1.828	1.802	1.892	**1.865**
-9.900	1.880	1.868	1.846	**1.861**	-9.870	1.895	1.900	1.884	**1.865**
-9.890	1.880	1.839	1.841	**1.862**	-9.860	1.868	1.848	1.936	**1.866**

-9.880	1.843	1.853	1.825	**1.862**	-9.850	1.924	1.951	1.914	**1.866**
-9.870	1.886	1.823	1.889	**1.863**	-9.840	1.894	1.936	1.858	**1.866**
-9.860	1.820	1.871	1.841	**1.863**	-9.830	1.807	1.822	1.870	**1.867**
-9.850	1.890	1.831	1.831	**1.864**	-9.820	1.803	1.790	1.792	**1.867**
-9.840	1.820	1.855	1.840	**1.864**	-9.810	1.888	1.814	1.820	**1.867**
-9.830	1.838	1.897	1.912	**1.865**	-9.800	1.839	1.836	1.910	**1.868**
-9.820	1.907	1.897	1.924	**1.865**	-9.790	1.861	1.825	1.893	**1.868**
-9.810	1.884	1.885	1.857	**1.866**	-9.780	1.925	1.929	1.848	**1.868**
-9.800	1.848	1.896	1.878	**1.866**	-9.770	1.904	1.863	1.859	**1.869**
-9.790	1.869	1.890	1.840	**1.867**	-9.760	1.927	1.881	1.917	**1.869**
-9.780	1.830	1.897	1.881	**1.867**	-9.750	1.894	1.837	1.897	**1.869**
-9.770	1.800	1.840	1.790	**1.867**	-9.740	1.862	1.809	1.880	**1.870**
-9.760	1.897	1.889	1.876	**1.868**	-9.730	1.870	1.844	1.876	**1.870**
-9.750	1.889	1.843	1.851	**1.868**	-9.720	1.838	1.838	1.915	**1.870**
-9.740	1.872	1.913	1.892	**1.868**	-9.710	1.965	1.886	1.935	**1.870**
-9.730	1.868	1.915	1.868	**1.868**	-9.700	1.834	1.844	1.854	**1.870**
-9.720	1.846	1.906	1.883	**1.868**	-9.690	1.896	1.836	1.867	**1.871**
-9.710	1.826	1.826	1.841	**1.868**	-9.680	1.866	1.887	1.806	**1.871**
-9.700	1.906	1.894	1.872	**1.867**	-9.670	1.883	1.850	1.797	**1.871**
-9.690	1.863	1.878	1.924	**1.867**	-9.660	1.874	1.866	1.860	**1.871**
-9.680	1.900	1.849	1.863	**1.867**	-9.650	1.833	1.922	1.895	**1.871**
-9.670	1.891	1.855	1.915	**1.866**	-9.640	1.861	1.939	1.866	**1.871**
-9.660	1.884	1.927	1.861	**1.866**	-9.630	1.919	1.871	1.961	**1.871**
-9.650	1.923	1.901	1.884	**1.865**	-9.620	1.912	1.904	1.828	**1.871**
-9.640	1.823	1.886	1.830	**1.865**	-9.610	1.849	1.890	1.886	**1.871**
-9.630	1.807	1.843	1.862	**1.865**	-9.600	1.845	1.815	1.854	**1.872**
-9.620	1.907	1.851	1.865	**1.864**	-9.590	1.844	1.792	1.841	**1.872**
-9.610	1.874	1.813	1.848	**1.864**	-9.580	1.914	1.922	1.934	**1.872**
-9.600	1.847	1.831	1.812	**1.864**	-9.570	1.831	1.847	1.803	**1.872**
-9.590	1.816	1.847	1.834	**1.863**	-9.560	1.774	1.825	1.806	**1.872**
-9.580	1.838	1.889	1.834	**1.863**	-9.550	1.875	1.853	1.894	**1.872**
-9.570	1.883	1.894	1.851	**1.862**	-9.540	1.825	1.889	1.882	**1.872**

-9.560	1.824	1.836	1.835	**1.862**
-9.550	1.825	1.861	1.849	**1.862**
-9.540	1.904	1.840	1.892	**1.862**
-9.530	1.859	1.844	1.839	**1.863**
-9.520	1.841	1.846	1.912	**1.863**
-9.510	1.881	1.860	1.820	**1.863**
-9.500	1.854	1.815	1.885	**1.863**
-9.490	1.877	1.867	1.880	**1.863**
-9.480	1.898	1.919	1.942	**1.863**
-9.470	1.863	1.839	1.872	**1.863**
-9.460	1.869	1.853	1.853	**1.863**
-9.450	1.819	1.862	1.806	**1.863**
-9.440	1.871	1.885	1.887	**1.863**
-9.430	1.896	1.881	1.895	**1.863**
-9.420	1.860	1.845	1.916	**1.863**
-9.410	1.786	1.816	1.828	**1.863**
-9.400	1.884	1.805	1.858	**1.863**
-9.390	1.880	1.847	1.887	**1.863**
-9.380	1.839	1.899	1.854	**1.863**
-9.370	1.850	1.832	1.907	**1.863**
-9.360	1.891	1.895	1.868	**1.863**
-9.350	1.882	1.924	1.866	**1.863**
-9.340	1.855	1.889	1.846	**1.863**
-9.330	1.866	1.921	1.888	**1.864**
-9.320	1.902	1.851	1.916	**1.864**
-9.310	1.897	1.846	1.864	**1.863**
-9.300	1.802	1.826	1.874	**1.863**
-9.290	1.832	1.856	1.817	**1.863**
-9.280	1.809	1.829	1.882	**1.864**
-9.270	1.851	1.815	1.850	**1.864**
-9.260	1.778	1.799	1.841	**1.864**
-9.250	1.821	1.874	1.838	**1.864**

-9.530	1.789	1.773	1.754	**1.871**
-9.520	1.838	1.875	1.897	**1.871**
-9.510	1.821	1.850	1.758	**1.871**
-9.500	1.810	1.896	1.812	**1.871**
-9.490	1.903	1.896	1.865	**1.871**
-9.480	1.959	1.886	1.905	**1.870**
-9.470	1.813	1.816	1.813	**1.870**
-9.460	1.855	1.885	1.930	**1.870**
-9.450	1.900	1.868	1.931	**1.869**
-9.440	1.811	1.855	1.820	**1.869**
-9.430	1.868	1.906	1.883	**1.869**
-9.420	1.828	1.818	1.793	**1.868**
-9.410	1.849	1.854	1.861	**1.868**
-9.400	1.848	1.892	1.860	**1.867**
-9.390	1.869	1.919	1.847	**1.866**
-9.380	1.863	1.864	1.858	**1.866**
-9.370	1.888	1.815	1.908	**1.865**
-9.360	1.830	1.818	1.879	**1.865**
-9.350	1.846	1.838	1.836	**1.864**
-9.340	1.906	1.839	1.858	**1.864**
-9.330	1.834	1.855	1.825	**1.864**
-9.320	1.889	1.876	1.905	**1.863**
-9.310	1.876	1.879	1.873	**1.863**
-9.300	1.877	1.843	1.797	**1.863**
-9.290	1.833	1.835	1.887	**1.863**
-9.280	1.890	1.853	1.875	**1.863**
-9.270	1.819	1.858	1.848	**1.863**
-9.260	1.898	1.906	1.836	**1.863**
-9.250	1.919	1.894	1.913	**1.863**
-9.240	1.816	1.800	1.886	**1.863**
-9.230	1.854	1.803	1.897	**1.862**
-9.220	1.931	1.875	1.876	**1.862**

-9.240	1.877	1.906	1.931	**1.864**	-9.210	1.869	1.828	1.864	**1.863**
-9.230	1.884	1.864	1.817	**1.864**	-9.200	1.926	1.859	1.857	**1.863**
-9.220	1.956	1.912	1.928	**1.864**	-9.190	1.932	1.887	1.885	**1.863**
-9.210	1.880	1.844	1.908	**1.865**	-9.180	1.856	1.822	1.840	**1.863**
-9.200	1.805	1.878	1.831	**1.865**	-9.170	1.884	1.817	1.862	**1.863**
-9.190	1.861	1.887	1.897	**1.865**	-9.160	1.908	1.854	1.921	**1.864**
-9.180	1.892	1.819	1.854	**1.865**	-9.150	1.868	1.888	1.886	**1.864**
-9.170	1.789	1.806	1.808	**1.865**	-9.140	1.879	1.865	1.864	**1.864**
-9.160	1.794	1.814	1.820	**1.865**	-9.130	1.828	1.923	1.849	**1.864**
-9.150	1.838	1.882	1.863	**1.865**	-9.120	1.863	1.811	1.867	**1.865**
-9.140	1.823	1.829	1.795	**1.866**	-9.110	1.813	1.831	1.887	**1.865**
-9.130	1.905	1.869	1.913	**1.866**	-9.100	1.884	1.861	1.878	**1.865**
-9.120	1.840	1.854	1.891	**1.866**	-9.090	1.887	1.857	1.878	**1.865**
-9.110	1.867	1.876	1.867	**1.866**	-9.080	1.848	1.840	1.841	**1.866**
-9.100	1.817	1.830	1.887	**1.866**	-9.070	1.870	1.816	1.831	**1.866**
-9.090	1.859	1.877	1.897	**1.866**	-9.060	1.860	1.884	1.856	**1.866**
-9.080	1.876	1.853	1.867	**1.866**	-9.050	1.867	1.836	1.791	**1.867**
-9.070	1.925	1.897	1.902	**1.866**	-9.040	1.918	1.893	1.928	**1.867**
-9.060	1.906	1.908	1.839	**1.866**	-9.030	1.872	1.840	1.911	**1.868**
-9.050	1.878	1.895	1.905	**1.866**	-9.020	1.834	1.829	1.895	**1.868**
-9.040	1.863	1.887	1.858	**1.865**	-9.010	1.840	1.817	1.830	**1.869**
-9.030	1.876	1.874	1.863	**1.865**	-9.000	1.837	1.800	1.832	**1.869**
-9.020	1.897	1.873	1.905	**1.865**	-8.990	1.899	1.958	1.868	**1.869**
-9.010	1.859	1.811	1.844	**1.865**	-8.980	1.851	1.881	1.880	**1.870**
-9.000	1.868	1.864	1.894	**1.865**	-8.970	1.829	1.876	1.840	**1.870**
-8.990	1.835	1.873	1.887	**1.865**	-8.960	1.901	1.871	1.906	**1.871**
-8.980	1.878	1.895	1.852	**1.865**	-8.950	1.925	1.864	1.855	**1.871**
-8.970	1.830	1.858	1.783	**1.864**	-8.940	1.908	1.872	1.965	**1.872**
-8.960	1.875	1.881	1.876	**1.864**	-8.930	1.807	1.797	1.819	**1.872**
-8.950	1.863	1.834	1.875	**1.864**	-8.920	1.880	1.870	1.861	**1.873**
-8.940	1.870	1.867	1.885	**1.864**	-8.910	1.920	1.865	1.899	**1.873**
-8.930	1.853	1.812	1.829	**1.863**	-8.900	1.868	1.876	1.885	**1.874**

-8.920	1.889	1.902	1.835	**1.863**	-8.890	1.851	1.844	1.868	**1.874**
-8.910	1.898	1.892	1.900	**1.863**	-8.880	1.872	1.916	1.840	**1.875**
-8.900	1.859	1.867	1.889	**1.863**	-8.870	1.926	1.861	1.902	**1.876**
-8.890	1.865	1.878	1.813	**1.863**	-8.860	1.968	1.883	1.901	**1.876**
-8.880	1.799	1.867	1.831	**1.863**	-8.850	1.842	1.889	1.838	**1.877**
-8.870	1.860	1.821	1.856	**1.863**	-8.840	1.868	1.842	1.882	**1.877**
-8.860	1.841	1.882	1.874	**1.863**	-8.830	1.834	1.921	1.891	**1.878**
-8.850	1.876	1.942	1.931	**1.862**	-8.820	1.814	1.780	1.803	**1.878**
-8.840	1.852	1.891	1.929	**1.862**	-8.810	1.832	1.878	1.898	**1.879**
-8.830	1.901	1.911	1.860	**1.862**	-8.800	1.819	1.789	1.854	**1.879**
-8.820	1.863	1.894	1.819	**1.862**	-8.790	1.794	1.867	1.821	**1.879**
-8.810	1.872	1.902	1.856	**1.862**	-8.780	1.902	1.848	1.883	**1.880**
-8.800	1.787	1.855	1.833	**1.862**	-8.770	1.866	1.814	1.897	**1.880**
-8.790	1.911	1.874	1.877	**1.862**	-8.760	1.825	1.822	1.845	**1.880**
-8.780	1.845	1.824	1.871	**1.862**	-8.750	1.866	1.873	1.864	**1.880**
-8.770	1.828	1.851	1.903	**1.862**	-8.740	1.932	1.998	1.909	**1.881**
-8.760	1.837	1.856	1.801	**1.862**	-8.730	1.891	1.943	1.888	**1.881**
-8.750	1.865	1.845	1.842	**1.863**	-8.720	1.879	1.833	1.841	**1.880**
-8.740	1.866	1.861	1.860	**1.863**	-8.710	1.878	1.913	1.929	**1.880**
-8.730	1.980	1.902	1.928	**1.863**	-8.700	1.847	1.856	1.836	**1.880**
-8.720	1.793	1.821	1.849	**1.863**	-8.690	1.818	1.895	1.865	**1.880**
-8.710	1.856	1.873	1.866	**1.864**	-8.680	1.947	1.858	1.924	**1.880**
-8.700	1.848	1.823	1.850	**1.864**	-8.670	1.854	1.903	1.942	**1.880**
-8.690	1.874	1.844	1.843	**1.865**	-8.660	1.855	1.889	1.885	**1.879**
-8.680	1.846	1.908	1.894	**1.865**	-8.650	1.962	1.896	1.879	**1.879**
-8.670	1.840	1.840	1.800	**1.865**	-8.640	1.905	1.814	1.856	**1.879**
-8.660	1.915	1.866	1.860	**1.866**	-8.630	1.900	1.867	1.840	**1.879**
-8.650	1.877	1.818	1.833	**1.866**	-8.620	1.800	1.848	1.770	**1.879**
-8.640	1.839	1.881	1.913	**1.866**	-8.610	1.897	1.875	1.814	**1.879**
-8.630	1.874	1.876	1.851	**1.866**	-8.600	1.898	1.959	1.897	**1.878**
-8.620	1.879	1.892	1.834	**1.866**	-8.590	1.879	1.882	1.894	**1.878**
-8.610	1.876	1.830	1.811	**1.867**	-8.580	1.932	1.893	1.937	**1.878**

-8.600	1.844	1.873	1.916	**1.867**	-8.570	1.873	1.919	1.852	**1.878**
-8.590	1.919	1.889	1.918	**1.867**	-8.560	1.816	1.891	1.818	**1.878**
-8.580	1.857	1.835	1.848	**1.867**	-8.550	1.826	1.901	1.892	**1.878**
-8.570	1.912	1.857	1.883	**1.868**	-8.540	1.945	1.897	1.950	**1.878**
-8.560	1.892	1.835	1.856	**1.869**	-8.530	1.918	1.959	1.885	**1.878**
-8.550	1.948	1.876	1.907	**1.870**	-8.520	1.773	1.845	1.864	**1.877**
-8.540	1.922	1.893	1.875	**1.871**	-8.510	1.887	1.854	1.904	**1.877**
-8.530	1.785	1.819	1.853	**1.872**	-8.500	1.877	1.859	1.940	**1.877**
-8.520	1.867	1.826	1.822	**1.872**	-8.490	1.903	1.916	1.870	**1.876**
-8.510	1.831	1.836	1.865	**1.873**	-8.480	1.831	1.880	1.842	**1.876**
-8.500	1.868	1.846	1.867	**1.873**	-8.470	1.880	1.837	1.803	**1.876**
-8.490	1.905	1.854	1.876	**1.873**	-8.460	1.896	1.848	1.895	**1.876**
-8.480	1.864	1.853	1.857	**1.874**	-8.450	1.935	1.874	1.844	**1.876**
-8.470	1.915	1.848	1.878	**1.874**	-8.440	1.893	1.920	1.897	**1.876**
-8.460	1.901	1.886	1.848	**1.874**	-8.430	1.874	1.873	1.825	**1.876**
-8.450	1.916	1.883	1.854	**1.874**	-8.420	1.869	1.874	1.906	**1.876**
-8.440	1.852	1.850	1.826	**1.875**	-8.410	1.915	1.887	1.836	**1.876**
-8.430	1.870	1.818	1.864	**1.875**	-8.400	1.807	1.881	1.865	**1.876**
-8.420	1.886	1.912	1.875	**1.874**	-8.390	1.892	1.872	1.856	**1.876**
-8.410	1.894	1.941	1.866	**1.874**	-8.380	1.906	1.887	1.840	**1.876**
-8.400	1.865	1.847	1.849	**1.874**	-8.370	1.877	1.828	1.837	**1.876**
-8.390	1.819	1.894	1.853	**1.874**	-8.360	1.898	1.833	1.844	**1.876**
-8.380	1.833	1.807	1.861	**1.874**	-8.350	1.850	1.869	1.844	**1.876**
-8.370	1.895	1.912	1.920	**1.873**	-8.340	1.906	1.881	1.945	**1.876**
-8.360	1.848	1.842	1.889	**1.873**	-8.330	1.837	1.923	1.902	**1.875**
-8.350	1.827	1.843	1.809	**1.873**	-8.320	1.907	1.870	1.871	**1.875**
-8.340	1.862	1.825	1.886	**1.873**	-8.310	1.894	1.911	1.862	**1.874**
-8.330	1.878	1.841	1.855	**1.874**	-8.300	1.882	1.852	1.933	**1.874**
-8.320	1.847	1.877	1.844	**1.874**	-8.290	1.948	1.888	1.946	**1.874**
-8.310	1.881	1.836	1.894	**1.874**	-8.280	1.871	1.866	1.853	**1.874**
-8.300	1.908	1.921	1.879	**1.874**	-8.270	1.897	1.853	1.871	**1.874**
-8.290	1.930	1.898	1.955	**1.874**	-8.260	1.911	1.839	1.909	**1.874**

-8.280	1.881	1.889	1.888	**1.874**	-8.250	1.896	1.876	1.963	**1.875**
-8.270	1.892	1.903	1.913	**1.874**	-8.240	1.914	1.873	1.910	**1.875**
-8.260	1.909	1.836	1.868	**1.874**	-8.230	1.912	1.885	1.830	**1.875**
-8.250	1.814	1.877	1.882	**1.874**	-8.220	1.792	1.831	1.870	**1.875**
-8.240	1.863	1.908	1.865	**1.875**	-8.210	1.895	1.909	1.859	**1.875**
-8.230	1.918	1.914	1.859	**1.875**	-8.200	1.863	1.909	1.917	**1.875**
-8.220	1.881	1.879	1.909	**1.875**	-8.190	1.889	1.928	1.830	**1.875**
-8.210	1.930	1.912	1.881	**1.875**	-8.180	1.860	1.851	1.871	**1.874**
-8.200	1.920	1.854	1.887	**1.876**	-8.170	1.932	1.866	1.883	**1.874**
-8.190	1.918	1.903	1.847	**1.877**	-8.160	1.852	1.913	1.890	**1.874**
-8.180	1.870	1.871	1.920	**1.877**	-8.150	1.887	1.918	1.871	**1.875**
-8.170	1.885	1.880	1.888	**1.878**	-8.140	1.935	1.874	1.910	**1.875**
-8.160	1.838	1.826	1.877	**1.878**	-8.130	1.879	1.901	1.884	**1.875**
-8.150	1.930	1.905	1.875	**1.879**	-8.120	1.904	1.854	1.935	**1.875**
-8.140	1.914	1.873	1.896	**1.879**	-8.110	1.858	1.893	1.818	**1.875**
-8.130	1.891	1.917	1.946	**1.879**	-8.100	1.944	1.917	1.860	**1.875**
-8.120	1.809	1.868	1.877	**1.879**	-8.090	1.831	1.856	1.894	**1.875**
-8.110	1.850	1.796	1.839	**1.879**	-8.080	1.897	1.909	1.847	**1.875**
-8.100	1.892	1.849	1.918	**1.880**	-8.070	1.877	1.867	1.844	**1.875**
-8.090	1.858	1.889	1.856	**1.880**	-8.060	1.917	1.893	1.858	**1.875**
-8.080	1.871	1.875	1.823	**1.880**	-8.050	1.855	1.874	1.840	**1.875**
-8.070	1.880	1.877	1.832	**1.880**	-8.040	1.830	1.872	1.855	**1.875**
-8.060	1.904	1.961	1.928	**1.880**	-8.030	1.841	1.908	1.895	**1.875**
-8.050	1.878	1.902	1.889	**1.880**	-8.020	1.848	1.881	1.803	**1.875**
-8.040	1.869	1.906	1.895	**1.879**	-8.010	1.908	1.906	1.927	**1.876**
-8.030	1.934	1.886	1.881	**1.879**	-8.000	1.908	1.915	1.862	**1.876**
-8.020	1.874	1.902	1.933	**1.879**	-7.990	1.843	1.777	1.830	**1.876**
-8.010	1.875	1.919	1.920	**1.879**	-7.980	1.884	1.899	1.892	**1.876**
-8.000	1.880	1.860	1.806	**1.880**	-7.970	1.898	1.866	1.877	**1.876**
-7.990	1.890	1.904	1.888	**1.880**	-7.960	1.924	1.860	1.918	**1.876**
-7.980	1.871	1.850	1.893	**1.881**	-7.950	1.836	1.841	1.836	**1.876**
-7.970	1.842	1.849	1.907	**1.881**	-7.940	1.833	1.827	1.855	**1.876**

-7.960	1.871	1.901	1.860	**1.881**	-7.930	1.925	1.890	1.868	**1.876**
-7.950	1.904	1.894	1.901	**1.882**	-7.920	1.877	1.895	1.892	**1.876**
-7.940	1.878	1.954	1.916	**1.882**	-7.910	1.829	1.856	1.863	**1.876**
-7.930	1.908	1.837	1.848	**1.883**	-7.900	1.886	1.884	1.887	**1.876**
-7.920	1.917	1.933	1.925	**1.883**	-7.890	1.839	1.904	1.929	**1.876**
-7.910	1.902	1.839	1.873	**1.883**	-7.880	1.866	1.859	1.901	**1.876**
-7.900	1.929	1.937	1.896	**1.883**	-7.870	1.876	1.836	1.853	**1.875**
-7.890	1.885	1.894	1.861	**1.884**	-7.860	1.867	1.926	1.855	**1.875**
-7.880	1.915	1.889	1.899	**1.884**	-7.850	1.873	1.867	1.935	**1.875**
-7.870	1.874	1.893	1.856	**1.884**	-7.840	1.801	1.875	1.870	**1.874**
-7.860	1.945	1.869	1.909	**1.884**	-7.830	1.872	1.904	1.874	**1.873**
-7.850	1.905	1.863	1.908	**1.884**	-7.820	1.908	1.959	1.908	**1.873**
-7.840	1.859	1.863	1.842	**1.884**	-7.810	1.853	1.925	1.915	**1.872**
-7.830	1.887	1.854	1.834	**1.884**	-7.800	1.905	1.891	1.843	**1.871**
-7.820	1.837	1.835	1.850	**1.884**	-7.790	1.910	1.865	1.872	**1.871**
-7.810	1.830	1.851	1.841	**1.884**	-7.780	1.868	1.847	1.810	**1.870**
-7.800	1.935	1.961	1.943	**1.884**	-7.770	1.867	1.901	1.891	**1.869**
-7.790	1.867	1.929	1.879	**1.883**	-7.760	1.786	1.878	1.823	**1.869**
-7.780	1.875	1.837	1.870	**1.883**	-7.750	1.873	1.814	1.904	**1.868**
-7.770	1.849	1.871	1.855	**1.883**	-7.740	1.893	1.853	1.896	**1.868**
-7.760	1.829	1.894	1.855	**1.883**	-7.730	1.854	1.804	1.805	**1.868**
-7.750	1.847	1.806	1.868	**1.882**	-7.720	1.861	1.820	1.842	**1.868**
-7.740	1.917	1.948	1.902	**1.882**	-7.710	1.871	1.875	1.790	**1.868**
-7.730	1.901	1.855	1.889	**1.882**	-7.700	1.885	1.864	1.901	**1.869**
-7.720	1.916	1.865	1.850	**1.881**	-7.690	1.877	1.934	1.878	**1.869**
-7.710	1.848	1.901	1.900	**1.880**	-7.680	1.832	1.767	1.818	**1.869**
-7.700	1.861	1.820	1.885	**1.879**	-7.670	1.809	1.877	1.802	**1.869**
-7.690	1.869	1.883	1.937	**1.879**	-7.660	1.826	1.789	1.753	**1.869**
-7.680	1.853	1.860	1.902	**1.878**	-7.650	1.825	1.896	1.860	**1.869**
-7.670	1.825	1.886	1.878	**1.877**	-7.640	1.870	1.903	1.942	**1.869**
-7.660	1.963	1.930	1.896	**1.876**	-7.630	1.772	1.847	1.832	**1.868**
-7.650	1.837	1.901	1.866	**1.875**	-7.620	1.834	1.739	1.803	**1.868**

-7.640	1.849	1.796	1.778	**1.875**	-7.610	1.928	1.902	1.873	**1.868**
-7.630	1.884	1.888	1.855	**1.874**	-7.600	1.848	1.905	1.941	**1.867**
-7.620	1.857	1.828	1.880	**1.873**	-7.590	1.823	1.921	1.870	**1.867**
-7.610	1.872	1.883	1.817	**1.873**	-7.580	1.848	1.875	1.896	**1.867**
-7.600	1.862	1.845	1.887	**1.873**	-7.570	1.945	1.954	1.894	**1.867**
-7.590	1.902	1.885	1.889	**1.873**	-7.560	1.833	1.886	1.901	**1.867**
-7.580	1.891	1.827	1.899	**1.873**	-7.550	1.919	1.905	1.837	**1.867**
-7.570	1.835	1.895	1.826	**1.873**	-7.540	1.803	1.800	1.841	**1.868**
-7.560	1.914	1.887	1.902	**1.873**	-7.530	1.893	1.945	1.863	**1.868**
-7.550	1.843	1.901	1.840	**1.873**	-7.520	1.872	1.930	1.900	**1.868**
-7.540	1.855	1.859	1.890	**1.873**	-7.510	1.836	1.819	1.753	**1.868**
-7.530	1.921	1.861	1.862	**1.873**	-7.500	1.862	1.861	1.848	**1.868**
-7.520	1.912	1.908	1.851	**1.873**	-7.490	1.915	1.868	1.903	**1.869**
-7.510	1.845	1.882	1.843	**1.873**	-7.480	1.907	1.883	1.825	**1.869**
-7.500	1.878	1.863	1.886	**1.872**	-7.470	1.864	1.878	1.874	**1.869**
-7.490	1.885	1.944	1.870	**1.872**	-7.460	1.952	1.875	1.949	**1.869**
-7.480	1.916	1.857	1.911	**1.872**	-7.450	1.909	1.902	1.834	**1.869**
-7.470	1.889	1.870	1.859	**1.871**	-7.440	1.889	1.893	1.898	**1.870**
-7.460	1.892	1.833	1.833	**1.871**	-7.430	1.906	1.929	1.874	**1.870**
-7.450	1.909	1.896	1.893	**1.872**	-7.420	1.897	1.871	1.919	**1.870**
-7.440	1.854	1.870	1.812	**1.872**	-7.410	1.914	1.863	1.885	**1.870**
-7.430	1.818	1.887	1.827	**1.872**	-7.400	1.853	1.864	1.886	**1.870**
-7.420	1.876	1.808	1.852	**1.873**	-7.390	1.914	1.915	1.889	**1.871**
-7.410	1.833	1.892	1.870	**1.873**	-7.380	1.934	1.890	1.932	**1.871**
-7.400	1.881	1.824	1.823	**1.873**	-7.370	1.934	1.863	1.895	**1.871**
-7.390	1.878	1.838	1.827	**1.873**	-7.360	1.876	1.880	1.897	**1.871**
-7.380	1.897	1.876	1.831	**1.873**	-7.350	1.896	1.800	1.861	**1.871**
-7.370	1.880	1.862	1.878	**1.873**	-7.340	1.910	1.816	1.883	**1.871**
-7.360	1.857	1.891	1.850	**1.873**	-7.330	1.909	1.868	1.873	**1.870**
-7.350	1.886	1.933	1.871	**1.873**	-7.320	1.847	1.875	1.905	**1.871**
-7.340	1.871	1.842	1.914	**1.873**	-7.310	1.938	1.899	1.850	**1.871**
-7.330	1.837	1.872	1.878	**1.872**	-7.300	1.922	1.901	1.864	**1.872**

-7.320	1.832	1.885	1.859	**1.872**	-7.290	1.902	1.882	1.963	**1.872**
-7.310	1.921	1.872	1.881	**1.872**	-7.280	1.861	1.936	1.932	**1.873**
-7.300	1.834	1.803	1.777	**1.871**	-7.270	1.846	1.907	1.918	**1.873**
-7.290	1.950	1.894	1.929	**1.871**	-7.260	1.866	1.858	1.915	**1.874**
-7.280	1.864	1.808	1.825	**1.871**	-7.250	1.933	1.947	1.943	**1.874**
-7.270	1.832	1.882	1.876	**1.871**	-7.240	1.930	1.874	1.874	**1.875**
-7.260	1.843	1.921	1.883	**1.872**	-7.230	1.831	1.868	1.895	**1.875**
-7.250	1.809	1.867	1.882	**1.872**	-7.220	1.806	1.856	1.856	**1.876**
-7.240	1.850	1.883	1.921	**1.873**	-7.210	1.881	1.860	1.860	**1.876**
-7.230	1.888	1.914	1.947	**1.873**	-7.200	1.812	1.819	1.812	**1.877**
-7.220	1.937	1.884	1.935	**1.874**	-7.190	1.822	1.860	1.905	**1.878**
-7.210	1.872	1.820	1.845	**1.874**	-7.180	1.845	1.810	1.883	**1.878**
-7.200	1.903	1.836	1.904	**1.875**	-7.170	1.875	1.899	1.896	**1.879**
-7.190	1.894	1.915	1.888	**1.875**	-7.160	1.840	1.931	1.852	**1.880**
-7.180	1.880	1.893	1.837	**1.876**	-7.150	1.838	1.889	1.913	**1.880**
-7.170	1.840	1.911	1.858	**1.876**	-7.140	1.904	1.896	1.911	**1.881**
-7.160	1.895	1.909	1.888	**1.877**	-7.130	1.866	1.915	1.912	**1.881**
-7.150	1.883	1.901	1.945	**1.877**	-7.120	1.864	1.851	1.869	**1.882**
-7.140	1.862	1.846	1.886	**1.878**	-7.110	1.922	1.908	1.849	**1.882**
-7.130	1.793	1.846	1.844	**1.879**	-7.100	1.889	1.892	1.903	**1.883**
-7.120	1.866	1.841	1.909	**1.879**	-7.090	1.901	1.943	1.916	**1.883**
-7.110	1.896	1.911	1.856	**1.880**	-7.080	1.888	1.933	1.979	**1.884**
-7.100	1.825	1.891	1.878	**1.880**	-7.070	1.942	1.856	1.867	**1.884**
-7.090	1.878	1.861	1.894	**1.881**	-7.060	1.860	1.868	1.831	**1.885**
-7.080	1.884	1.873	1.866	**1.882**	-7.050	1.898	1.806	1.859	**1.885**
-7.070	1.859	1.816	1.837	**1.883**	-7.040	1.902	1.925	1.938	**1.886**
-7.060	1.834	1.901	1.883	**1.884**	-7.030	1.910	1.829	1.878	**1.886**
-7.050	1.898	1.874	1.840	**1.885**	-7.020	1.861	1.855	1.921	**1.887**
-7.040	1.931	1.971	1.925	**1.886**	-7.010	1.848	1.861	1.863	**1.887**
-7.030	1.950	1.925	1.896	**1.887**	-7.000	1.882	1.833	1.899	**1.888**
-7.020	1.879	1.913	1.849	**1.888**	-6.990	1.902	1.929	1.863	**1.888**
-7.010	1.834	1.822	1.878	**1.889**	-6.980	1.967	1.871	1.915	**1.888**

-7.000	1.852	1.922	1.910	**1.889**	-6.970	1.935	1.871	1.936	**1.889**
-6.990	2.006	1.969	1.949	**1.890**	-6.960	1.944	1.881	1.872	**1.889**
-6.980	1.909	1.927	1.934	**1.890**	-6.950	1.861	1.880	1.867	**1.889**
-6.970	1.944	1.993	1.920	**1.890**	-6.940	1.851	1.857	1.859	**1.889**
-6.960	1.918	1.905	1.914	**1.890**	-6.930	1.887	1.848	1.827	**1.889**
-6.950	1.889	1.890	1.900	**1.890**	-6.920	1.947	1.928	1.889	**1.889**
-6.940	1.903	1.923	1.868	**1.890**	-6.910	1.905	1.858	1.895	**1.889**
-6.930	1.927	1.914	1.945	**1.890**	-6.900	1.932	1.862	1.861	**1.889**
-6.920	1.911	1.875	1.909	**1.890**	-6.890	1.878	1.926	1.839	**1.888**
-6.910	1.899	1.833	1.874	**1.890**	-6.880	1.835	1.874	1.875	**1.888**
-6.900	1.865	1.870	1.879	**1.890**	-6.870	1.792	1.809	1.827	**1.887**
-6.890	1.853	1.900	1.903	**1.890**	-6.860	1.823	1.883	1.866	**1.887**
-6.880	1.871	1.875	1.860	**1.890**	-6.850	1.966	1.923	1.885	**1.886**
-6.870	1.924	1.898	1.883	**1.890**	-6.840	1.824	1.894	1.903	**1.886**
-6.860	1.869	1.878	1.916	**1.890**	-6.830	1.908	1.868	1.836	**1.886**
-6.850	1.886	1.935	1.932	**1.890**	-6.820	1.921	1.887	1.848	**1.886**
-6.840	1.854	1.902	1.839	**1.890**	-6.810	1.961	1.879	1.880	**1.886**
-6.830	1.961	1.916	1.926	**1.890**	-6.800	1.884	1.873	1.845	**1.886**
-6.820	1.902	1.888	1.937	**1.889**	-6.790	1.864	1.832	1.825	**1.886**
-6.810	1.844	1.822	1.882	**1.889**	-6.780	1.920	1.882	1.861	**1.885**
-6.800	1.888	1.850	1.899	**1.889**	-6.770	1.872	1.880	1.838	**1.885**
-6.790	1.918	1.929	1.943	**1.888**	-6.760	1.873	1.841	1.924	**1.884**
-6.780	1.883	1.871	1.884	**1.888**	-6.750	1.908	1.866	1.900	**1.883**
-6.770	1.873	1.887	1.931	**1.887**	-6.740	1.840	1.853	1.852	**1.883**
-6.760	1.887	1.890	1.833	**1.887**	-6.730	1.833	1.858	1.871	**1.882**
-6.750	1.943	1.910	1.880	**1.886**	-6.720	1.855	1.914	1.946	**1.881**
-6.740	1.879	1.904	1.872	**1.886**	-6.710	1.931	1.900	1.935	**1.881**
-6.730	1.917	1.890	1.895	**1.885**	-6.700	1.939	1.908	1.980	**1.880**
-6.720	1.917	1.899	1.901	**1.884**	-6.690	1.867	1.897	1.857	**1.880**
-6.710	1.902	1.913	1.929	**1.883**	-6.680	1.848	1.831	1.842	**1.880**
-6.700	1.884	1.852	1.932	**1.883**	-6.670	1.816	1.865	1.845	**1.880**
-6.690	1.863	1.844	1.916	**1.882**	-6.660	1.907	1.941	1.895	**1.880**

-6.680	1.920	1.860	1.924	**1.881**	-6.650	1.900	1.840	1.873	**1.880**
-6.670	1.861	1.871	1.893	**1.880**	-6.640	1.875	1.868	1.917	**1.880**
-6.660	1.900	1.836	1.859	**1.879**	-6.630	1.840	1.863	1.857	**1.879**
-6.650	1.815	1.849	1.874	**1.878**	-6.620	1.900	1.942	1.968	**1.879**
-6.640	1.833	1.839	1.881	**1.878**	-6.610	1.915	1.953	1.956	**1.878**
-6.630	1.871	1.876	1.886	**1.877**	-6.600	1.913	1.853	1.923	**1.878**
-6.620	1.887	1.857	1.923	**1.876**	-6.590	1.895	1.822	1.850	**1.878**
-6.610	1.789	1.843	1.828	**1.875**	-6.580	1.839	1.905	1.828	**1.877**
-6.600	1.881	1.855	1.884	**1.875**	-6.570	1.815	1.889	1.834	**1.878**
-6.590	1.917	1.861	1.922	**1.874**	-6.560	1.944	1.904	1.861	**1.878**
-6.580	1.916	1.882	1.853	**1.874**	-6.550	1.905	1.950	1.890	**1.879**
-6.570	1.833	1.884	1.888	**1.874**	-6.540	1.864	1.828	1.883	**1.879**
-6.560	1.902	1.893	1.829	**1.874**	-6.530	1.830	1.863	1.859	**1.880**
-6.550	1.957	1.900	1.886	**1.874**	-6.520	1.878	1.903	1.859	**1.880**
-6.540	1.891	1.892	1.905	**1.874**	-6.510	1.830	1.866	1.889	**1.880**
-6.530	1.894	1.852	1.838	**1.874**	-6.500	1.926	1.841	1.882	**1.881**
-6.520	1.810	1.830	1.841	**1.874**	-6.490	1.852	1.827	1.879	**1.881**
-6.510	1.933	1.908	1.897	**1.874**	-6.480	1.873	1.879	1.830	**1.881**
-6.500	1.924	1.905	1.854	**1.874**	-6.470	1.855	1.898	1.916	**1.881**
-6.490	1.803	1.829	1.801	**1.874**	-6.460	1.872	1.831	1.822	**1.881**
-6.480	1.842	1.831	1.898	**1.874**	-6.450	1.841	1.872	1.858	**1.880**
-6.470	1.844	1.854	1.866	**1.875**	-6.440	1.923	1.859	1.944	**1.880**
-6.460	1.862	1.867	1.878	**1.875**	-6.430	1.839	1.802	1.895	**1.880**
-6.450	1.883	1.881	1.911	**1.875**	-6.420	1.945	1.867	1.941	**1.879**
-6.440	1.858	1.888	1.883	**1.875**	-6.410	1.867	1.847	1.817	**1.879**
-6.430	1.886	1.859	1.922	**1.876**	-6.400	1.840	1.840	1.865	**1.879**
-6.420	1.843	1.840	1.856	**1.876**	-6.390	1.886	1.891	1.837	**1.879**
-6.410	1.863	1.902	1.869	**1.876**	-6.380	1.896	1.905	1.872	**1.879**
-6.400	1.880	1.860	1.896	**1.876**	-6.370	1.868	1.819	1.895	**1.879**
-6.390	1.869	1.850	1.859	**1.876**	-6.360	1.960	1.916	1.872	**1.878**
-6.380	1.855	1.897	1.867	**1.877**	-6.350	1.907	1.887	1.907	**1.878**
-6.370	1.904	1.892	1.873	**1.877**	-6.340	1.884	1.879	1.958	**1.878**

-6.360	1.843	1.915	1.910	**1.877**	-6.330	1.884	1.910	1.917	**1.878**
-6.350	1.933	1.923	1.875	**1.877**	-6.320	1.943	1.869	1.908	**1.877**
-6.340	1.934	1.928	1.890	**1.877**	-6.310	1.880	1.853	1.872	**1.877**
-6.330	1.881	1.859	1.860	**1.877**	-6.300	1.900	1.897	1.946	**1.877**
-6.320	1.874	1.894	1.924	**1.877**	-6.290	1.881	1.893	1.829	**1.877**
-6.310	1.838	1.863	1.869	**1.878**	-6.280	1.880	1.864	1.920	**1.877**
-6.300	1.908	1.860	1.869	**1.878**	-6.270	1.904	1.970	1.927	**1.877**
-6.290	1.886	1.898	1.845	**1.878**	-6.260	1.896	1.897	1.871	**1.877**
-6.280	1.857	1.841	1.822	**1.878**	-6.250	1.884	1.885	1.859	**1.876**
-6.270	1.900	1.877	1.902	**1.878**	-6.240	1.800	1.841	1.833	**1.876**
-6.260	1.871	1.939	1.922	**1.879**	-6.230	1.880	1.884	1.867	**1.876**
-6.250	1.887	1.869	1.893	**1.879**	-6.220	1.913	1.859	1.888	**1.876**
-6.240	1.872	1.909	1.899	**1.879**	-6.210	1.833	1.827	1.861	**1.876**
-6.230	1.863	1.872	1.852	**1.879**	-6.200	1.917	1.886	1.891	**1.876**
-6.220	1.866	1.857	1.863	**1.879**	-6.190	1.840	1.841	1.904	**1.875**
-6.210	1.912	1.878	1.855	**1.879**	-6.180	1.926	1.874	1.867	**1.875**
-6.200	1.868	1.906	1.907	**1.879**	-6.170	1.894	1.870	1.886	**1.875**
-6.190	1.907	1.904	1.863	**1.879**	-6.160	1.873	1.895	1.890	**1.875**
-6.180	1.887	1.921	1.873	**1.879**	-6.150	1.813	1.882	1.825	**1.875**
-6.170	1.876	1.899	1.944	**1.879**	-6.140	1.857	1.880	1.923	**1.875**
-6.160	1.854	1.877	1.895	**1.879**	-6.130	1.884	1.922	1.898	**1.874**
-6.150	1.901	1.889	1.908	**1.879**	-6.120	1.852	1.890	1.873	**1.874**
-6.140	1.831	1.873	1.860	**1.879**	-6.110	1.895	1.880	1.868	**1.874**
-6.130	1.901	1.884	1.900	**1.879**	-6.100	1.838	1.916	1.846	**1.874**
-6.120	1.854	1.842	1.907	**1.879**	-6.090	1.824	1.906	1.861	**1.874**
-6.110	1.882	1.866	1.832	**1.879**	-6.080	1.835	1.806	1.858	**1.874**
-6.100	1.843	1.919	1.881	**1.879**	-6.070	1.864	1.917	1.952	**1.874**
-6.090	1.869	1.833	1.909	**1.879**	-6.060	1.868	1.833	1.888	**1.874**
-6.080	1.831	1.888	1.854	**1.879**	-6.050	1.843	1.774	1.826	**1.874**
-6.070	1.880	1.856	1.818	**1.879**	-6.040	1.887	1.934	1.929	**1.874**
-6.060	1.812	1.862	1.862	**1.879**	-6.030	1.793	1.856	1.874	**1.874**
-6.050	1.862	1.886	1.819	**1.879**	-6.020	1.873	1.885	1.883	**1.874**

-6.040	1.870	1.866	1.893	**1.879**	-6.010	1.847	1.861	1.838	**1.873**
-6.030	1.921	1.909	1.929	**1.879**	-6.000	1.855	1.814	1.905	**1.873**
-6.020	1.885	1.881	1.903	**1.878**	-5.990	1.819	1.788	1.809	**1.873**
-6.010	1.917	1.868	1.914	**1.878**	-5.980	1.914	1.954	1.934	**1.873**
-6.000	1.851	1.866	1.893	**1.877**	-5.970	1.861	1.799	1.855	**1.873**
-5.990	1.914	1.909	1.879	**1.877**	-5.960	1.892	1.966	1.919	**1.874**
-5.980	1.884	1.837	1.890	**1.876**	-5.950	1.887	1.923	1.848	**1.874**
-5.970	1.901	1.901	1.851	**1.876**	-5.940	1.809	1.840	1.815	**1.874**
-5.960	1.879	1.885	1.876	**1.876**	-5.930	1.864	1.832	1.858	**1.874**
-5.950	1.899	1.860	1.911	**1.876**	-5.920	1.855	1.895	1.880	**1.874**
-5.940	1.865	1.841	1.843	**1.876**	-5.910	1.876	1.886	1.880	**1.874**
-5.930	1.869	1.848	1.919	**1.876**	-5.900	1.909	1.961	1.907	**1.874**
-5.920	1.887	1.911	1.847	**1.876**	-5.890	1.907	1.935	1.918	**1.873**
-5.910	1.869	1.879	1.901	**1.876**	-5.880	1.816	1.860	1.792	**1.873**
-5.900	1.895	1.862	1.886	**1.876**	-5.870	1.902	1.968	1.952	**1.873**
-5.890	1.923	1.878	1.887	**1.875**	-5.860	1.882	1.866	1.916	**1.873**
-5.880	1.927	1.917	1.906	**1.875**	-5.850	1.938	1.859	1.868	**1.872**
-5.870	1.847	1.874	1.858	**1.875**	-5.840	1.831	1.834	1.806	**1.872**
-5.860	1.869	1.890	1.892	**1.875**	-5.830	1.862	1.878	1.941	**1.871**
-5.850	1.883	1.811	1.868	**1.876**	-5.820	1.894	1.825	1.846	**1.870**
-5.840	1.910	1.959	1.910	**1.876**	-5.810	1.837	1.845	1.855	**1.870**
-5.830	1.902	1.842	1.859	**1.876**	-5.800	1.827	1.838	1.894	**1.869**
-5.820	1.894	1.933	1.877	**1.877**	-5.790	1.885	1.892	1.916	**1.868**
-5.810	1.880	1.926	1.926	**1.877**	-5.780	1.913	1.841	1.852	**1.868**
-5.800	1.894	1.834	1.859	**1.877**	-5.770	1.839	1.860	1.821	**1.868**
-5.790	1.905	1.918	1.902	**1.878**	-5.760	1.934	1.887	1.850	**1.868**
-5.780	1.863	1.890	1.825	**1.878**	-5.750	1.847	1.887	1.913	**1.868**
-5.770	1.863	1.866	1.862	**1.879**	-5.740	1.915	1.859	1.864	**1.868**
-5.760	1.907	1.833	1.853	**1.880**	-5.730	1.838	1.896	1.860	**1.868**
-5.750	1.898	1.841	1.867	**1.881**	-5.720	1.815	1.852	1.867	**1.868**
-5.740	1.895	1.886	1.836	**1.881**	-5.710	1.861	1.890	1.888	**1.868**
-5.730	1.917	1.859	1.905	**1.882**	-5.700	1.892	1.902	1.895	**1.868**

-5.720	1.830	1.879	1.908	**1.883**	-5.690	1.917	1.894	1.912	**1.868**
-5.710	1.899	1.860	1.902	**1.884**	-5.680	1.876	1.946	1.936	**1.868**
-5.700	1.850	1.835	1.893	**1.885**	-5.670	1.873	1.907	1.924	**1.868**
-5.690	1.920	1.907	1.851	**1.886**	-5.660	1.943	1.861	1.931	**1.868**
-5.680	1.914	1.896	1.891	**1.887**	-5.650	1.923	1.917	1.928	**1.868**
-5.670	1.878	1.880	1.930	**1.887**	-5.640	1.863	1.899	1.923	**1.868**
-5.660	1.868	1.860	1.897	**1.888**	-5.630	1.846	1.804	1.901	**1.868**
-5.650	1.910	1.883	1.954	**1.888**	-5.620	1.878	1.891	1.867	**1.868**
-5.640	1.926	1.937	1.936	**1.889**	-5.610	1.826	1.892	1.915	**1.868**
-5.630	1.845	1.837	1.868	**1.889**	-5.600	1.883	1.857	1.866	**1.868**
-5.620	1.884	1.910	1.853	**1.889**	-5.590	1.857	1.799	1.881	**1.868**
-5.610	1.946	1.926	1.874	**1.890**	-5.580	1.922	1.872	1.841	**1.869**
-5.600	1.923	1.869	1.859	**1.890**	-5.570	1.903	1.931	1.893	**1.870**
-5.590	1.918	1.926	1.872	**1.889**	-5.560	1.895	1.881	1.841	**1.870**
-5.580	1.909	1.885	1.894	**1.889**	-5.550	1.878	1.927	1.868	**1.871**
-5.570	1.892	1.854	1.852	**1.889**	-5.540	1.906	1.868	1.901	**1.871**
-5.560	1.896	1.906	1.840	**1.889**	-5.530	1.841	1.813	1.750	**1.872**
-5.550	1.881	1.867	1.928	**1.889**	-5.520	1.910	1.890	1.909	**1.873**
-5.540	1.831	1.908	1.859	**1.888**	-5.510	1.850	1.855	1.886	**1.873**
-5.530	1.919	1.914	1.871	**1.888**	-5.500	1.883	1.829	1.851	**1.874**
-5.520	1.882	1.814	1.834	**1.888**	-5.490	1.861	1.930	1.926	**1.875**
-5.510	1.865	1.905	1.842	**1.888**	-5.480	1.890	1.812	1.887	**1.875**
-5.500	1.876	1.922	1.866	**1.887**	-5.470	1.838	1.915	1.873	**1.876**
-5.490	1.898	1.901	1.902	**1.887**	-5.460	1.896	1.893	1.915	**1.877**
-5.480	1.874	1.871	1.851	**1.887**	-5.450	1.925	1.899	1.905	**1.877**
-5.470	1.909	1.937	1.901	**1.886**	-5.440	1.930	1.895	1.866	**1.878**
-5.460	1.856	1.855	1.833	**1.885**	-5.430	1.850	1.800	1.880	**1.878**
-5.450	1.853	1.918	1.857	**1.885**	-5.420	1.909	1.927	1.873	**1.879**
-5.440	1.858	1.864	1.828	**1.884**	-5.410	1.887	1.899	1.913	**1.879**
-5.430	1.845	1.880	1.865	**1.883**	-5.400	1.855	1.870	1.865	**1.880**
-5.420	1.870	1.897	1.909	**1.883**	-5.390	1.859	1.922	1.910	**1.880**
-5.410	1.896	1.844	1.914	**1.882**	-5.380	1.895	1.911	1.983	**1.880**

-5.400	1.900	1.918	1.915	**1.881**	-5.370	1.815	1.891	1.828	**1.881**
-5.390	1.870	1.837	1.853	**1.880**	-5.360	1.789	1.844	1.868	**1.881**
-5.380	1.865	1.864	1.919	**1.880**	-5.350	1.915	1.887	1.863	**1.881**
-5.370	1.878	1.884	1.880	**1.878**	-5.340	1.894	1.825	1.897	**1.882**
-5.360	1.901	1.912	1.861	**1.877**	-5.330	1.934	1.881	1.941	**1.882**
-5.350	1.835	1.876	1.872	**1.877**	-5.320	1.884	1.911	1.830	**1.882**
-5.340	1.878	1.876	1.863	**1.876**	-5.310	1.808	1.852	1.869	**1.882**
-5.330	1.812	1.846	1.863	**1.876**	-5.300	1.870	1.852	1.865	**1.883**
-5.320	1.852	1.819	1.870	**1.875**	-5.290	1.887	1.858	1.918	**1.883**
-5.310	1.855	1.901	1.890	**1.875**	-5.280	1.889	1.879	1.907	**1.883**
-5.300	1.876	1.847	1.858	**1.874**	-5.270	1.882	1.929	1.886	**1.883**
-5.290	1.845	1.845	1.848	**1.874**	-5.260	1.895	1.857	1.859	**1.882**
-5.280	1.779	1.852	1.828	**1.873**	-5.250	1.905	1.915	1.851	**1.882**
-5.270	1.875	1.877	1.890	**1.873**	-5.240	1.881	1.830	1.909	**1.882**
-5.260	1.871	1.853	1.867	**1.873**	-5.230	1.878	1.863	1.812	**1.881**
-5.250	1.848	1.893	1.916	**1.872**	-5.220	1.924	1.924	1.876	**1.881**
-5.240	1.840	1.866	1.849	**1.872**	-5.210	1.985	1.910	1.910	**1.880**
-5.230	1.817	1.839	1.822	**1.873**	-5.200	1.880	1.917	1.842	**1.879**
-5.220	1.810	1.870	1.869	**1.873**	-5.190	1.874	1.829	1.817	**1.879**
-5.210	1.883	1.876	1.814	**1.874**	-5.180	1.874	1.922	1.844	**1.878**
-5.200	1.891	1.905	1.870	**1.874**	-5.170	1.921	1.829	1.886	**1.881**
-5.190	1.874	1.820	1.867	**1.875**	-5.160	1.959	1.902	1.945	**1.883**
-5.180	1.864	1.891	1.880	**1.876**	-5.150	1.888	1.851	1.911	**1.885**
-5.170	1.909	1.912	1.870	**1.876**	-5.140	1.918	1.956	1.872	**1.885**
-5.160	1.882	1.884	1.881	**1.877**	-5.130	1.908	1.950	1.870	**1.885**
-5.150	1.917	1.905	1.859	**1.878**	-5.120	1.914	1.815	1.870	**1.885**
-5.140	1.876	1.882	1.857	**1.879**	-5.110	1.879	1.850	1.806	**1.885**
-5.130	1.910	1.831	1.879	**1.880**	-5.100	1.899	1.897	1.916	**1.885**
-5.120	1.845	1.885	1.873	**1.880**	-5.090	1.933	1.841	1.898	**1.885**
-5.110	1.832	1.884	1.856	**1.881**	-5.080	1.892	1.945	1.931	**1.885**
-5.100	1.886	1.913	1.922	**1.882**	-5.070	1.845	1.793	1.843	**1.884**
-5.090	1.881	1.885	1.893	**1.883**	-5.060	1.933	1.884	1.874	**1.884**

-5.080	1.888	1.940	1.883	**1.884**	-5.050	1.849	1.853	1.907	**1.884**
-5.070	1.914	1.896	1.864	**1.885**	-5.040	1.921	1.908	1.892	**1.883**
-5.060	1.907	1.883	1.940	**1.885**	-5.030	1.942	1.874	1.882	**1.883**
-5.050	1.899	1.839	1.861	**1.886**	-5.020	1.833	1.892	1.875	**1.882**
-5.040	1.893	1.868	1.891	**1.887**	-5.010	1.830	1.909	1.895	**1.881**
-5.030	1.872	1.879	1.944	**1.888**	-5.000	1.983	1.906	1.906	**1.880**
-5.020	1.905	1.850	1.866	**1.889**	-4.990	1.870	1.824	1.820	**1.879**
-5.010	1.861	1.874	1.928	**1.889**	-4.980	1.896	1.956	1.898	**1.879**
-5.000	1.896	1.870	1.916	**1.890**	-4.970	1.926	1.854	1.894	**1.878**
-4.990	1.877	1.933	1.876	**1.891**	-4.960	1.865	1.838	1.915	**1.878**
-4.980	1.914	1.885	1.889	**1.892**	-4.950	1.863	1.897	1.855	**1.878**
-4.970	1.878	1.876	1.886	**1.892**	-4.940	1.886	1.865	1.875	**1.878**
-4.960	1.920	1.930	1.861	**1.892**	-4.930	1.856	1.823	1.855	**1.878**
-4.950	1.886	1.873	1.856	**1.893**	-4.920	1.893	1.868	1.958	**1.878**
-4.940	1.952	1.897	1.886	**1.893**	-4.910	1.825	1.829	1.872	**1.878**
-4.930	1.882	1.880	1.847	**1.893**	-4.900	1.895	1.886	1.813	**1.878**
-4.920	1.933	1.923	1.943	**1.893**	-4.890	1.865	1.853	1.874	**1.877**
-4.910	1.906	1.899	1.926	**1.893**	-4.880	1.845	1.841	1.886	**1.877**
-4.900	1.842	1.889	1.902	**1.892**	-4.870	1.861	1.876	1.858	**1.876**
-4.890	1.923	1.908	1.926	**1.892**	-4.860	1.869	1.887	1.843	**1.876**
-4.880	1.892	1.889	1.923	**1.892**	-4.850	1.843	1.908	1.923	**1.876**
-4.870	1.928	1.901	1.875	**1.891**	-4.840	1.800	1.854	1.843	**1.875**
-4.860	1.900	1.930	1.935	**1.890**	-4.830	1.858	1.917	1.903	**1.875**
-4.850	1.870	1.864	1.817	**1.890**	-4.820	1.874	1.918	1.847	**1.874**
-4.840	1.867	1.912	1.847	**1.889**	-4.810	1.829	1.825	1.818	**1.874**
-4.830	1.845	1.925	1.883	**1.888**	-4.800	1.907	1.814	1.874	**1.874**
-4.820	1.897	1.912	1.968	**1.887**	-4.790	1.868	1.835	1.879	**1.874**
-4.810	1.856	1.833	1.862	**1.886**	-4.780	1.886	1.848	1.863	**1.873**
-4.800	1.928	1.981	1.904	**1.885**	-4.770	1.928	1.904	1.833	**1.873**
-4.790	1.855	1.892	1.888	**1.884**	-4.760	1.812	1.848	1.899	**1.873**
-4.780	1.937	1.924	1.893	**1.884**	-4.750	1.818	1.861	1.764	**1.873**
-4.770	1.886	1.929	1.854	**1.883**	-4.740	1.824	1.897	1.914	**1.873**

-4.760	1.919	1.867	1.910	**1.882**	-4.730	1.944	1.895	1.892	**1.874**
-4.750	1.876	1.895	1.904	**1.882**	-4.720	1.901	1.896	1.907	**1.874**
-4.740	1.849	1.886	1.857	**1.881**	-4.710	1.853	1.931	1.923	**1.875**
-4.730	1.917	1.883	1.891	**1.881**	-4.700	1.862	1.845	1.874	**1.876**
-4.720	1.911	1.913	1.897	**1.881**	-4.690	1.910	1.934	1.926	**1.876**
-4.710	1.890	1.867	1.850	**1.881**	-4.680	1.796	1.834	1.878	**1.876**
-4.700	1.828	1.893	1.875	**1.881**	-4.670	1.874	1.871	1.929	**1.877**
-4.690	1.872	1.904	1.842	**1.881**	-4.660	1.883	1.921	1.873	**1.877**
-4.680	1.883	1.959	1.908	**1.881**	-4.650	1.857	1.902	1.878	**1.878**
-4.670	1.858	1.865	1.920	**1.881**	-4.640	1.852	1.901	1.836	**1.878**
-4.660	1.873	1.941	1.910	**1.881**	-4.630	1.924	1.942	1.969	**1.878**
-4.650	1.935	1.867	1.895	**1.881**	-4.620	1.913	1.926	1.918	**1.879**
-4.640	1.862	1.890	1.849	**1.882**	-4.610	1.829	1.873	1.900	**1.879**
-4.630	1.874	1.889	1.884	**1.882**	-4.600	1.822	1.868	1.808	**1.879**
-4.620	1.929	1.932	1.917	**1.882**	-4.590	1.867	1.891	1.897	**1.880**
-4.610	1.878	1.873	1.938	**1.882**	-4.580	1.914	1.929	1.869	**1.880**
-4.600	1.849	1.896	1.862	**1.882**	-4.570	1.928	1.932	1.872	**1.880**
-4.590	1.921	1.899	1.875	**1.882**	-4.560	1.886	1.926	1.877	**1.880**
-4.580	1.926	1.919	1.891	**1.883**	-4.550	1.844	1.855	1.848	**1.880**
-4.570	1.794	1.850	1.833	**1.883**	-4.540	1.875	1.887	1.890	**1.880**
-4.560	1.865	1.818	1.868	**1.884**	-4.530	1.869	1.804	1.853	**1.880**
-4.550	1.874	1.935	1.872	**1.884**	-4.520	1.829	1.914	1.860	**1.880**
-4.540	1.858	1.925	1.862	**1.884**	-4.510	1.904	1.877	1.852	**1.880**
-4.530	1.886	1.914	1.926	**1.885**	-4.500	1.844	1.812	1.841	**1.881**
-4.520	1.925	1.860	1.874	**1.885**	-4.490	1.850	1.876	1.902	**1.881**
-4.510	1.914	1.940	1.902	**1.886**	-4.480	1.893	1.924	1.883	**1.881**
-4.500	1.924	1.855	1.881	**1.886**	-4.470	1.932	1.872	1.867	**1.881**
-4.490	1.865	1.861	1.871	**1.886**	-4.460	1.922	1.897	1.888	**1.881**
-4.480	1.833	1.878	1.886	**1.887**	-4.450	1.858	1.873	1.837	**1.881**
-4.470	1.849	1.891	1.905	**1.887**	-4.440	1.871	1.891	1.961	**1.881**
-4.460	1.890	1.884	1.846	**1.886**	-4.430	1.860	1.861	1.893	**1.882**
-4.450	1.868	1.918	1.934	**1.886**	-4.420	1.889	1.883	1.887	**1.882**

-4.440	1.896	1.905	1.912	**1.886**	-4.410	1.861	1.849	1.848	**1.882**
-4.430	1.841	1.854	1.896	**1.886**	-4.400	1.805	1.796	1.882	**1.883**
-4.420	1.910	1.912	1.908	**1.886**	-4.390	1.816	1.869	1.845	**1.883**
-4.410	1.852	1.915	1.870	**1.886**	-4.380	1.905	1.861	1.889	**1.883**
-4.400	1.878	1.799	1.848	**1.886**	-4.370	1.905	1.904	1.908	**1.884**
-4.390	1.816	1.876	1.853	**1.886**	-4.360	1.838	1.911	1.818	**1.884**
-4.380	1.872	1.901	1.834	**1.886**	-4.350	1.922	1.895	1.861	**1.884**
-4.370	1.920	1.927	1.883	**1.886**	-4.340	1.911	1.897	1.867	**1.884**
-4.360	1.882	1.883	1.914	**1.886**	-4.330	1.938	1.865	1.916	**1.885**
-4.350	1.893	1.860	1.858	**1.886**	-4.320	1.827	1.875	1.899	**1.885**
-4.340	1.870	1.866	1.872	**1.886**	-4.310	1.921	1.887	1.902	**1.885**
-4.330	1.896	1.866	1.924	**1.886**	-4.300	1.897	1.962	1.893	**1.885**
-4.320	1.878	1.905	1.874	**1.886**	-4.290	1.887	1.956	1.930	**1.885**
-4.310	1.919	1.883	1.871	**1.886**	-4.280	1.853	1.886	1.830	**1.886**
-4.300	1.880	1.900	1.950	**1.885**	-4.270	1.937	1.867	1.851	**1.886**
-4.290	1.802	1.876	1.840	**1.885**	-4.260	1.828	1.802	1.886	**1.886**
-4.280	1.900	1.835	1.907	**1.885**	-4.250	1.857	1.866	1.892	**1.886**
-4.270	1.870	1.903	1.883	**1.885**	-4.240	1.894	1.869	1.872	**1.886**
-4.260	1.877	1.893	1.865	**1.885**	-4.230	1.867	1.874	1.840	**1.887**
-4.250	1.902	1.882	1.896	**1.885**	-4.220	1.887	1.932	1.946	**1.887**
-4.240	1.920	1.898	1.855	**1.885**	-4.210	1.849	1.834	1.794	**1.887**
-4.230	1.913	1.936	1.929	**1.885**	-4.200	1.876	1.922	1.962	**1.887**
-4.220	1.866	1.878	1.882	**1.885**	-4.190	1.891	1.864	1.898	**1.887**
-4.210	1.865	1.871	1.805	**1.885**	-4.180	1.846	1.892	1.883	**1.887**
-4.200	1.882	1.874	1.815	**1.885**	-4.170	1.825	1.870	1.910	**1.887**
-4.190	1.857	1.827	1.887	**1.884**	-4.160	1.958	1.915	1.984	**1.887**
-4.180	1.838	1.897	1.874	**1.884**	-4.150	1.852	1.896	1.879	**1.887**
-4.170	1.900	1.909	1.836	**1.883**	-4.140	1.894	1.833	1.882	**1.887**
-4.160	1.873	1.812	1.845	**1.883**	-4.130	1.871	1.868	1.869	**1.887**
-4.150	1.880	1.857	1.914	**1.882**	-4.120	1.873	1.886	1.951	**1.886**
-4.140	1.852	1.911	1.921	**1.882**	-4.110	1.867	1.886	1.804	**1.886**
-4.130	1.901	1.887	1.903	**1.881**	-4.100	1.895	1.890	1.854	**1.886**

-4.120	1.839	1.839	1.867	**1.881**
-4.110	1.894	1.891	1.872	**1.880**
-4.100	1.865	1.893	1.844	**1.879**
-4.090	1.845	1.856	1.854	**1.878**
-4.080	1.820	1.878	1.822	**1.877**
-4.070	1.883	1.900	1.908	**1.876**
-4.060	1.852	1.843	1.850	**1.875**
-4.050	1.884	1.951	1.905	**1.874**
-4.040	1.863	1.873	1.875	**1.873**
-4.030	1.875	1.883	1.828	**1.872**
-4.020	1.853	1.888	1.872	**1.871**
-4.010	1.854	1.898	1.906	**1.870**
-4.000	1.860	1.806	1.844	**1.869**
-3.990	1.848	1.896	1.868	**1.868**
-3.980	1.871	1.909	1.844	**1.867**
-3.970	1.837	1.859	1.816	**1.866**
-3.960	1.823	1.861	1.839	**1.866**
-3.950	1.920	1.880	1.879	**1.865**
-3.940	1.840	1.829	1.843	**1.866**
-3.930	1.915	1.908	1.904	**1.867**
-3.920	1.855	1.910	1.872	**1.868**
-3.910	1.821	1.882	1.877	**1.869**
-3.900	1.898	1.894	1.911	**1.869**
-3.890	1.912	1.855	1.913	**1.869**
-3.880	1.874	1.919	1.924	**1.869**
-3.870	1.906	1.856	1.880	**1.869**
-3.860	1.873	1.879	1.844	**1.869**
-3.850	1.873	1.870	1.924	**1.869**
-3.840	1.879	1.801	1.834	**1.870**
-3.830	1.880	1.823	1.857	**1.871**
-3.820	1.914	1.895	1.854	**1.872**
-3.810	1.900	1.846	1.833	**1.873**

-4.090	1.905	1.872	1.872	**1.886**
-4.080	1.891	1.892	1.922	**1.886**
-4.070	1.915	1.839	1.837	**1.886**
-4.060	1.865	1.858	1.933	**1.885**
-4.050	1.936	1.896	1.936	**1.885**
-4.040	1.891	1.951	1.946	**1.885**
-4.030	1.874	1.927	1.863	**1.885**
-4.020	1.857	1.913	1.880	**1.885**
-4.010	1.890	1.942	1.860	**1.885**
-4.000	1.897	1.923	1.827	**1.885**
-3.990	1.890	1.877	1.908	**1.885**
-3.980	1.898	1.887	1.844	**1.885**
-3.970	1.932	1.961	1.911	**1.886**
-3.960	1.928	1.888	1.866	**1.886**
-3.950	1.915	1.878	1.885	**1.886**
-3.940	1.953	1.905	1.939	**1.886**
-3.930	1.935	1.949	1.917	**1.887**
-3.920	1.829	1.792	1.883	**1.887**
-3.910	1.836	1.815	1.880	**1.887**
-3.900	1.900	1.874	1.953	**1.887**
-3.890	1.893	1.951	1.908	**1.887**
-3.880	1.939	1.978	1.922	**1.887**
-3.870	1.846	1.865	1.922	**1.887**
-3.860	1.873	1.868	1.835	**1.887**
-3.850	1.868	1.927	1.840	**1.887**
-3.840	1.931	1.932	1.923	**1.887**
-3.830	1.947	1.889	1.955	**1.887**
-3.820	1.870	1.949	1.895	**1.887**
-3.810	1.864	1.869	1.844	**1.887**
-3.800	1.826	1.899	1.905	**1.888**
-3.790	1.849	1.904	1.863	**1.890**
-3.780	1.940	1.958	1.914	**1.892**

-3.800	1.936	1.879	1.904	**1.874**	-3.770	1.894	1.809	1.856	**1.894**
-3.790	1.856	1.842	1.817	**1.875**	-3.760	1.905	1.835	1.858	**1.897**
-3.780	1.915	1.861	1.918	**1.876**	-3.750	1.915	1.910	1.906	**1.900**
-3.770	1.898	1.883	1.846	**1.877**	-3.740	1.904	1.960	1.992	**1.902**
-3.760	1.898	1.898	1.947	**1.877**	-3.730	1.938	1.865	1.938	**1.904**
-3.750	1.845	1.861	1.834	**1.878**	-3.720	1.936	1.934	1.905	**1.908**
-3.740	1.928	1.952	1.925	**1.879**	-3.710	1.901	1.917	1.882	**1.912**
-3.730	1.888	1.857	1.914	**1.880**	-3.700	1.946	1.914	1.983	**1.916**
-3.720	1.948	1.902	1.919	**1.880**	-3.690	1.957	1.931	1.987	**1.920**
-3.710	1.877	1.853	1.859	**1.881**	-3.680	1.967	1.941	1.879	**1.924**
-3.700	1.932	1.856	1.891	**1.882**	-3.670	1.995	1.922	1.910	**1.928**
-3.690	1.928	1.901	1.937	**1.883**	-3.660	1.933	1.984	1.937	**1.931**
-3.680	1.901	1.897	1.864	**1.884**	-3.650	1.878	1.921	1.895	**1.935**
-3.670	1.853	1.907	1.915	**1.886**	-3.640	1.925	1.876	1.957	**1.939**
-3.660	1.825	1.871	1.824	**1.888**	-3.630	1.926	1.899	1.914	**1.943**
-3.650	1.921	1.893	1.939	**1.891**	-3.620	1.955	1.887	1.949	**1.947**
-3.640	1.850	1.926	1.900	**1.894**	-3.610	1.905	1.993	1.979	**1.951**
-3.630	1.878	1.884	1.864	**1.898**	-3.600	1.949	1.906	1.929	**1.955**
-3.620	1.911	1.908	1.890	**1.901**	-3.590	1.933	1.975	2.020	**1.958**
-3.610	1.914	1.938	1.905	**1.905**	-3.580	1.925	1.980	1.909	**1.962**
-3.600	1.920	1.939	1.891	**1.909**	-3.570	1.966	2.040	2.047	**1.966**
-3.590	1.929	1.980	1.915	**1.913**	-3.560	1.979	2.059	1.999	**1.970**
-3.580	1.915	1.970	1.896	**1.916**	-3.550	2.009	2.002	1.949	**1.974**
-3.570	1.987	1.996	1.929	**1.920**	-3.540	1.988	2.004	2.037	**1.978**
-3.560	1.944	1.880	1.917	**1.924**	-3.530	2.015	1.993	2.008	**1.982**
-3.550	2.000	1.942	1.924	**1.927**	-3.520	2.033	1.950	2.001	**1.985**
-3.540	1.929	1.991	1.958	**1.931**	-3.510	1.991	1.980	1.964	**1.989**
-3.530	1.913	1.960	1.910	**1.935**	-3.500	1.960	1.935	1.970	**1.993**
-3.520	1.977	1.919	1.970	**1.939**	-3.490	1.982	2.046	2.021	**1.997**
-3.510	1.930	1.964	1.933	**1.942**	-3.480	2.069	2.001	1.996	**2.001**
-3.500	2.001	1.981	1.924	**1.946**	-3.470	1.934	1.999	1.987	**2.005**
-3.490	1.980	2.006	1.943	**1.950**	-3.460	2.007	1.998	2.080	**2.009**

-3.480	1.931	1.960	1.933	**1.953**	-3.450	2.046	2.098	2.078	**2.012**
-3.470	1.932	1.947	1.997	**1.957**	-3.440	1.989	2.003	2.078	**2.016**
-3.460	1.977	1.921	1.969	**1.961**	-3.430	1.999	2.016	1.998	**2.020**
-3.450	1.966	1.962	1.996	**1.965**	-3.420	2.045	2.078	1.994	**2.024**
-3.440	1.956	1.985	1.948	**1.968**	-3.410	2.044	2.032	2.002	**2.028**
-3.430	1.951	1.946	1.987	**1.972**	-3.400	2.061	1.989	2.020	**2.032**
-3.420	1.993	2.024	1.959	**1.976**	-3.390	2.098	2.083	2.019	**2.035**
-3.410	1.908	1.954	1.942	**1.979**	-3.380	2.057	2.006	2.000	**2.039**
-3.400	1.977	1.978	2.029	**1.983**	-3.370	2.068	2.005	2.043	**2.043**
-3.390	1.999	1.988	1.984	**1.987**	-3.360	2.095	2.038	2.036	**2.047**
-3.380	2.044	2.008	1.979	**1.991**	-3.350	2.090	2.125	2.094	**2.051**
-3.370	1.975	1.979	2.011	**1.994**	-3.340	2.062	2.082	2.056	**2.055**
-3.360	1.964	1.990	1.956	**1.998**	-3.330	2.037	2.026	2.020	**2.059**
-3.350	1.925	1.983	1.995	**2.002**	-3.320	2.020	2.063	1.983	**2.062**
-3.340	2.035	1.980	1.960	**2.005**	-3.310	2.060	1.990	2.053	**2.066**
-3.330	2.063	2.029	2.012	**2.009**	-3.300	2.024	2.077	2.119	**2.070**
-3.320	2.031	2.029	1.969	**2.013**	-3.290	1.990	2.037	2.037	**2.074**
-3.310	2.005	2.028	2.042	**2.017**	-3.280	2.055	2.068	2.032	**2.078**
-3.300	2.005	1.944	1.982	**2.020**	-3.270	2.069	2.132	2.089	**2.082**
-3.290	2.029	2.012	2.082	**2.024**	-3.260	2.060	2.100	2.112	**2.085**
-3.280	2.022	1.978	2.035	**2.028**	-3.250	2.091	2.097	2.167	**2.088**
-3.270	2.058	2.044	2.015	**2.031**	-3.240	2.103	2.121	2.081	**2.091**
-3.260	2.023	2.081	2.071	**2.035**	-3.230	2.062	2.126	2.088	**2.094**
-3.250	2.004	2.003	2.022	**2.038**	-3.220	2.101	2.126	2.141	**2.096**
-3.240	2.015	2.065	2.013	**2.042**	-3.210	2.087	2.050	2.120	**2.099**
-3.230	2.117	2.045	2.052	**2.045**	-3.200	2.168	2.071	2.127	**2.102**
-3.220	1.970	2.015	1.951	**2.049**	-3.190	2.176	2.101	2.122	**2.105**
-3.210	2.033	2.078	2.105	**2.052**	-3.180	2.102	2.061	2.067	**2.107**
-3.200	2.066	2.021	2.085	**2.056**	-3.170	2.120	2.111	2.164	**2.108**
-3.190	2.055	2.076	2.043	**2.059**	-3.160	2.143	2.148	2.182	**2.110**
-3.180	2.074	2.036	2.076	**2.062**	-3.150	2.130	2.118	2.082	**2.111**
-3.170	2.041	2.062	2.013	**2.065**	-3.140	2.120	2.181	2.129	**2.112**

-3.160	2.038	2.066	2.056	**2.068**	-3.130	2.099	2.118	2.030	**2.113**
-3.150	2.122	2.053	2.120	**2.072**	-3.120	2.040	2.061	2.057	**2.114**
-3.140	2.040	2.035	1.995	**2.075**	-3.110	2.225	2.144	2.164	**2.115**
-3.130	2.096	2.095	2.144	**2.078**	-3.100	2.102	2.079	2.156	**2.116**
-3.120	2.173	2.134	2.093	**2.082**	-3.090	2.179	2.102	2.086	**2.117**
-3.110	2.121	2.084	2.075	**2.085**	-3.080	2.066	2.095	2.126	**2.118**
-3.100	2.067	2.119	2.103	**2.088**	-3.070	2.047	2.125	2.042	**2.119**
-3.090	2.090	2.095	2.107	**2.091**	-3.060	2.084	2.108	2.140	**2.119**
-3.080	2.132	2.128	2.125	**2.094**	-3.050	2.110	2.091	2.032	**2.120**
-3.070	2.096	2.117	2.112	**2.096**	-3.040	2.101	2.178	2.142	**2.121**
-3.060	2.043	2.073	2.060	**2.099**	-3.030	2.091	2.136	2.151	**2.122**
-3.050	2.136	2.085	2.103	**2.101**	-3.020	2.086	2.137	2.059	**2.122**
-3.040	2.150	2.113	2.154	**2.103**	-3.010	2.089	2.092	2.132	**2.123**
-3.030	2.102	2.105	2.127	**2.106**	-3.000	2.082	2.122	2.089	**2.124**
-3.020	2.094	2.124	2.133	**2.108**	-2.990	2.123	2.090	2.087	**2.124**
-3.010	2.095	2.070	2.093	**2.110**	-2.980	2.176	2.102	2.153	**2.125**
-3.000	2.098	2.136	2.111	**2.112**	-2.970	2.113	2.075	2.124	**2.126**
-2.990	2.148	2.080	2.128	**2.114**	-2.960	2.121	2.141	2.146	**2.126**
-2.980	2.153	2.143	2.090	**2.117**	-2.950	2.112	2.164	2.142	**2.127**
-2.970	2.141	2.097	2.120	**2.119**	-2.940	2.192	2.127	2.138	**2.127**
-2.960	2.128	2.114	2.078	**2.120**	-2.930	2.101	2.130	2.144	**2.127**
-2.950	2.125	2.105	2.162	**2.121**	-2.920	2.107	2.076	2.110	**2.127**
-2.940	2.150	2.187	2.146	**2.123**	-2.910	2.111	2.068	2.060	**2.127**
-2.930	2.109	2.076	2.035	**2.124**	-2.900	2.138	2.163	2.155	**2.128**
-2.920	2.072	2.092	2.143	**2.125**	-2.890	2.128	2.097	2.045	**2.128**
-2.910	2.155	2.196	2.170	**2.126**	-2.880	2.078	2.097	2.113	**2.128**
-2.900	2.100	2.134	2.145	**2.126**	-2.870	2.140	2.139	2.169	**2.128**
-2.890	2.152	2.081	2.100	**2.127**	-2.860	2.074	2.104	2.091	**2.128**
-2.880	2.146	2.090	2.134	**2.128**	-2.850	2.090	2.084	2.060	**2.128**
-2.870	2.131	2.124	2.187	**2.129**	-2.840	2.085	2.064	2.081	**2.128**
-2.860	2.140	2.141	2.124	**2.130**	-2.830	2.185	2.156	2.145	**2.128**
-2.850	2.190	2.217	2.169	**2.130**	-2.820	2.098	2.159	2.093	**2.128**

-2.840	2.135	2.138	2.138	**2.131**	-2.810	2.086	2.095	2.103	**2.128**
-2.830	2.167	2.118	2.143	**2.132**	-2.800	2.154	2.146	2.173	**2.128**
-2.820	2.098	2.171	2.110	**2.132**	-2.790	2.164	2.138	2.090	**2.128**
-2.810	2.077	2.119	2.109	**2.132**	-2.780	2.110	2.178	2.187	**2.128**
-2.800	2.096	2.107	2.155	**2.132**	-2.770	2.146	2.128	2.096	**2.128**
-2.790	2.139	2.161	2.085	**2.132**	-2.760	2.137	2.112	2.066	**2.128**
-2.780	2.186	2.117	2.121	**2.132**	-2.750	2.088	2.080	2.083	**2.128**
-2.770	2.124	2.063	2.122	**2.132**	-2.740	2.121	2.138	2.079	**2.128**
-2.760	2.201	2.153	2.133	**2.132**	-2.730	2.105	2.081	2.142	**2.128**
-2.750	2.129	2.122	2.120	**2.132**	-2.720	2.058	2.059	2.109	**2.128**
-2.740	2.170	2.155	2.135	**2.132**	-2.710	2.012	2.087	2.061	**2.128**
-2.730	2.148	2.094	2.151	**2.132**	-2.700	2.039	2.083	2.130	**2.128**
-2.720	2.197	2.179	2.144	**2.132**	-2.690	2.083	2.163	2.159	**2.128**
-2.710	2.150	2.129	2.091	**2.132**	-2.680	2.080	2.122	2.069	**2.128**
-2.700	2.102	2.095	2.111	**2.132**	-2.670	2.097	2.119	2.048	**2.128**
-2.690	2.170	2.173	2.185	**2.132**	-2.660	2.188	2.157	2.172	**2.128**
-2.680	2.104	2.171	2.165	**2.132**	-2.650	2.125	2.097	2.105	**2.128**
-2.670	2.112	2.096	2.118	**2.132**	-2.640	2.182	2.223	2.179	**2.128**
-2.660	2.122	2.135	2.119	**2.132**	-2.630	2.108	2.074	2.072	**2.128**
-2.650	2.148	2.087	2.125	**2.133**	-2.620	2.109	2.053	2.083	**2.128**
-2.640	2.116	2.123	2.103	**2.133**	-2.610	2.121	2.133	2.087	**2.127**
-2.630	2.109	2.137	2.143	**2.133**	-2.600	2.114	2.125	2.109	**2.127**
-2.620	2.157	2.123	2.156	**2.134**	-2.590	2.162	2.082	2.150	**2.127**
-2.610	2.147	2.113	2.111	**2.134**	-2.580	2.152	2.078	2.150	**2.127**
-2.600	2.109	2.137	2.120	**2.134**	-2.570	2.043	2.041	2.081	**2.127**
-2.590	2.094	2.108	2.109	**2.134**	-2.560	2.176	2.142	2.160	**2.127**
-2.580	2.134	2.096	2.108	**2.134**	-2.550	2.078	2.170	2.126	**2.127**
-2.570	2.160	2.133	2.163	**2.134**	-2.540	2.095	2.185	2.165	**2.128**
-2.560	2.072	2.085	2.092	**2.134**	-2.530	2.119	2.142	2.109	**2.128**
-2.550	2.115	2.130	2.159	**2.134**	-2.520	2.086	2.139	2.114	**2.128**
-2.540	2.189	2.138	2.133	**2.133**	-2.510	2.109	2.146	2.104	**2.128**
-2.530	2.097	2.136	2.141	**2.133**	-2.500	2.115	2.210	2.137	**2.128**

-2.520	2.136	2.114	2.076	**2.132**	-2.490	2.131	2.069	2.074	**2.128**
-2.510	2.093	2.052	2.104	**2.132**	-2.480	2.111	2.076	2.091	**2.128**
-2.500	2.101	2.145	2.076	**2.131**	-2.470	2.086	2.066	2.128	**2.127**
-2.490	2.087	2.089	2.150	**2.131**	-2.460	2.128	2.058	2.103	**2.127**
-2.480	2.132	2.104	2.083	**2.130**	-2.450	2.139	2.191	2.109	**2.127**
-2.470	2.051	2.105	2.076	**2.129**	-2.440	2.142	2.089	2.109	**2.127**
-2.460	2.113	2.148	2.140	**2.128**	-2.430	2.144	2.100	2.092	**2.127**
-2.450	2.154	2.102	2.083	**2.127**	-2.420	2.176	2.227	2.141	**2.127**
-2.440	2.134	2.113	2.123	**2.126**	-2.410	2.175	2.091	2.113	**2.126**
-2.430	2.091	2.130	2.101	**2.126**	-2.400	2.109	2.192	2.104	**2.126**
-2.420	2.159	2.134	2.117	**2.125**	-2.390	2.127	2.156	2.105	**2.126**
-2.410	2.185	2.169	2.127	**2.124**	-2.380	2.080	2.115	2.090	**2.126**
-2.400	2.144	2.119	2.136	**2.124**	-2.370	2.099	2.170	2.120	**2.126**
-2.390	2.116	2.094	2.108	**2.123**	-2.360	2.064	2.125	2.067	**2.126**
-2.380	2.131	2.091	2.153	**2.123**	-2.350	2.142	2.168	2.105	**2.126**
-2.370	2.126	2.182	2.117	**2.122**	-2.340	2.134	2.169	2.159	**2.126**
-2.360	2.109	2.129	2.143	**2.122**	-2.330	2.128	2.043	2.104	**2.126**
-2.350	2.120	2.182	2.126	**2.121**	-2.320	2.161	2.131	2.079	**2.126**
-2.340	2.093	2.097	2.123	**2.120**	-2.310	2.128	2.098	2.080	**2.126**
-2.330	2.141	2.090	2.137	**2.121**	-2.300	2.100	2.091	2.090	**2.126**
-2.320	2.092	2.121	2.131	**2.121**	-2.290	2.154	2.168	2.161	**2.126**
-2.310	2.108	2.118	2.153	**2.122**	-2.280	2.135	2.193	2.146	**2.126**
-2.300	2.076	2.136	2.098	**2.122**	-2.270	2.164	2.124	2.096	**2.126**
-2.290	2.135	2.147	2.117	**2.123**	-2.260	2.176	2.156	2.180	**2.126**
-2.280	2.100	2.074	2.126	**2.123**	-2.250	2.103	2.129	2.178	**2.126**
-2.270	2.142	2.144	2.153	**2.124**	-2.240	2.073	2.154	2.150	**2.126**
-2.260	2.128	2.130	2.166	**2.124**	-2.230	2.203	2.223	2.191	**2.125**
-2.250	2.084	2.050	2.063	**2.124**	-2.220	2.069	2.043	2.058	**2.125**
-2.240	2.145	2.094	2.118	**2.125**	-2.210	2.161	2.099	2.071	**2.125**
-2.230	2.168	2.140	2.102	**2.125**	-2.200	2.116	2.120	2.057	**2.125**
-2.220	2.196	2.140	2.201	**2.125**	-2.190	2.126	2.071	2.083	**2.125**
-2.210	2.178	2.138	2.119	**2.126**	-2.180	2.170	2.098	2.090	**2.125**

-2.200	2.132	2.108	2.135	**2.126**	-2.170	2.200	2.176	2.147	**2.125**
-2.190	2.101	2.115	2.118	**2.126**	-2.160	2.141	2.080	2.091	**2.125**
-2.180	2.135	2.128	2.173	**2.126**	-2.150	2.164	2.128	2.098	**2.125**
-2.170	2.153	2.142	2.079	**2.127**	-2.140	2.077	2.098	2.091	**2.125**
-2.160	2.161	2.133	2.113	**2.127**	-2.130	2.188	2.165	2.135	**2.125**
-2.150	2.156	2.158	2.214	**2.127**	-2.120	2.147	2.128	2.128	**2.125**
-2.140	2.080	2.079	2.108	**2.128**	-2.110	2.079	2.160	2.150	**2.125**
-2.130	2.168	2.106	2.101	**2.128**	-2.100	2.113	2.061	2.126	**2.125**
-2.120	2.116	2.130	2.111	**2.128**	-2.090	2.116	2.130	2.143	**2.126**
-2.110	2.154	2.215	2.147	**2.128**	-2.080	2.148	2.200	2.162	**2.126**
-2.100	2.176	2.150	2.134	**2.128**	-2.070	2.147	2.201	2.204	**2.126**
-2.090	2.185	2.120	2.152	**2.128**	-2.060	2.076	2.117	2.087	**2.126**
-2.080	2.113	2.188	2.166	**2.128**	-2.050	2.120	2.119	2.063	**2.127**
-2.070	2.109	2.119	2.105	**2.128**	-2.040	2.146	2.148	2.117	**2.127**
-2.060	2.064	2.046	2.119	**2.128**	-2.030	2.068	2.082	2.135	**2.127**
-2.050	2.182	2.197	2.185	**2.127**	-2.020	2.137	2.105	2.155	**2.126**
-2.040	2.080	2.061	2.117	**2.127**	-2.010	2.139	2.169	2.124	**2.126**
-2.030	2.108	2.122	2.150	**2.127**	-2.000	2.131	2.157	2.128	**2.126**
-2.020	2.119	2.115	2.141	**2.127**	-1.990	2.180	2.095	2.149	**2.126**
-2.010	2.147	2.094	2.147	**2.127**	-1.980	2.119	2.148	2.084	**2.126**
-2.000	2.123	2.132	2.149	**2.127**	-1.970	2.076	2.097	2.092	**2.126**
-1.990	2.136	2.119	2.073	**2.127**	-1.960	2.154	2.208	2.119	**2.126**
-1.980	2.146	2.152	2.152	**2.128**	-1.950	2.123	2.118	2.189	**2.126**
-1.970	2.155	2.170	2.099	**2.128**	-1.940	2.112	2.142	2.076	**2.126**
-1.960	2.152	2.154	2.112	**2.128**	-1.930	2.165	2.088	2.146	**2.126**
-1.950	2.129	2.098	2.146	**2.128**	-1.920	2.121	2.116	2.075	**2.127**
-1.940	2.139	2.099	2.139	**2.129**	-1.910	2.194	2.151	2.125	**2.127**
-1.930	2.146	2.099	2.127	**2.129**	-1.900	2.137	2.198	2.116	**2.127**
-1.920	2.178	2.133	2.143	**2.129**	-1.890	2.093	2.147	2.172	**2.127**
-1.910	2.180	2.112	2.148	**2.129**	-1.880	2.108	2.063	2.041	**2.127**
-1.900	2.173	2.176	2.155	**2.129**	-1.870	2.069	2.118	2.143	**2.127**
-1.890	2.142	2.112	2.150	**2.129**	-1.860	2.094	2.144	2.091	**2.127**

-1.880	2.090	2.116	2.049	**2.129**	-1.850	2.071	2.079	2.158	**2.127**
-1.870	2.180	2.140	2.106	**2.129**	-1.840	2.105	2.108	2.125	**2.127**
-1.860	2.108	2.120	2.133	**2.129**	-1.830	2.078	2.100	2.160	**2.127**
-1.850	2.099	2.103	2.119	**2.129**	-1.820	2.178	2.127	2.140	**2.128**
-1.840	2.107	2.186	2.139	**2.129**	-1.810	2.089	2.104	2.108	**2.128**
-1.830	2.118	2.077	2.149	**2.129**	-1.800	2.154	2.116	2.185	**2.128**
-1.820	2.111	2.100	2.156	**2.129**	-1.790	2.077	2.143	2.096	**2.128**
-1.810	2.101	2.108	2.116	**2.129**	-1.780	2.181	2.121	2.100	**2.129**
-1.800	2.169	2.145	2.191	**2.129**	-1.770	2.118	2.141	2.180	**2.129**
-1.790	2.092	2.132	2.134	**2.129**	-1.760	2.125	2.171	2.114	**2.129**
-1.780	2.083	2.157	2.122	**2.129**	-1.750	2.186	2.176	2.103	**2.129**
-1.770	2.181	2.158	2.107	**2.129**	-1.740	2.139	2.187	2.131	**2.129**
-1.760	2.187	2.176	2.166	**2.129**	-1.730	2.094	2.186	2.121	**2.129**
-1.750	2.157	2.165	2.170	**2.129**	-1.720	2.188	2.121	2.149	**2.128**
-1.740	2.088	2.107	2.162	**2.129**	-1.710	2.132	2.150	2.078	**2.128**
-1.730	2.159	2.164	2.095	**2.129**	-1.700	2.145	2.088	2.151	**2.127**
-1.720	2.163	2.146	2.159	**2.129**	-1.690	2.169	2.104	2.115	**2.127**
-1.710	2.102	2.095	2.096	**2.128**	-1.680	2.184	2.094	2.144	**2.126**
-1.700	2.139	2.175	2.151	**2.128**	-1.670	2.141	2.129	2.091	**2.126**
-1.690	2.125	2.117	2.076	**2.128**	-1.660	2.089	2.125	2.104	**2.125**
-1.680	2.105	2.123	2.116	**2.128**	-1.650	2.104	2.152	2.161	**2.125**
-1.670	2.057	2.096	2.086	**2.127**	-1.640	2.121	2.140	2.186	**2.124**
-1.660	2.143	2.127	2.108	**2.127**	-1.630	2.095	2.143	2.149	**2.124**
-1.650	2.088	2.089	2.039	**2.127**	-1.620	2.112	2.112	2.123	**2.124**
-1.640	2.115	2.153	2.095	**2.126**	-1.610	2.108	2.138	2.092	**2.123**
-1.630	2.119	2.134	2.133	**2.126**	-1.600	2.121	2.054	2.105	**2.123**
-1.620	2.081	2.111	2.113	**2.126**	-1.590	2.082	2.108	2.139	**2.122**
-1.610	2.142	2.167	2.168	**2.125**	-1.580	2.125	2.170	2.127	**2.122**
-1.600	2.161	2.118	2.114	**2.125**	-1.570	2.189	2.131	2.157	**2.122**
-1.590	2.126	2.138	2.080	**2.124**	-1.560	2.138	2.144	2.133	**2.122**
-1.580	2.096	2.123	2.138	**2.124**	-1.550	2.099	2.002	2.059	**2.121**
-1.570	2.145	2.134	2.151	**2.123**	-1.540	2.044	2.097	1.998	**2.121**

-1.560	2.129	2.104	2.162	**2.123**	-1.530	2.115	2.096	2.097	**2.121**
-1.550	2.122	2.117	2.074	**2.122**	-1.520	2.160	2.109	2.091	**2.120**
-1.540	2.152	2.089	2.155	**2.122**	-1.510	2.065	2.125	2.135	**2.120**
-1.530	2.069	2.129	2.104	**2.121**	-1.500	2.125	2.138	2.157	**2.120**
-1.520	2.116	2.064	2.098	**2.121**	-1.490	2.171	2.091	2.124	**2.120**
-1.510	2.158	2.212	2.148	**2.120**	-1.480	2.142	2.122	2.136	**2.119**
-1.500	2.171	2.131	2.123	**2.120**	-1.470	2.068	2.150	2.095	**2.119**
-1.490	2.168	2.147	2.172	**2.119**	-1.460	2.081	2.018	2.050	**2.118**
-1.480	2.096	2.056	2.040	**2.118**	-1.450	2.116	2.119	2.153	**2.118**
-1.470	2.201	2.165	2.151	**2.118**	-1.440	2.082	2.078	2.143	**2.118**
-1.460	2.141	2.081	2.126	**2.117**	-1.430	2.145	2.115	2.118	**2.117**
-1.450	2.109	2.138	2.097	**2.116**	-1.420	2.083	2.178	2.127	**2.117**
-1.440	2.137	2.142	2.098	**2.116**	-1.410	2.154	2.172	2.152	**2.116**
-1.430	2.067	2.065	2.073	**2.115**	-1.400	2.191	2.131	2.150	**2.115**
-1.420	2.120	2.119	2.061	**2.114**	-1.390	2.108	2.141	2.113	**2.115**
-1.410	2.156	2.122	2.140	**2.114**	-1.380	2.100	2.167	2.111	**2.114**
-1.400	2.089	2.092	2.075	**2.113**	-1.370	2.059	2.142	2.075	**2.114**
-1.390	2.079	2.090	2.062	**2.113**	-1.360	2.083	2.087	2.170	**2.114**
-1.380	2.112	2.078	2.080	**2.113**	-1.350	2.120	2.058	2.097	**2.113**
-1.370	2.142	2.110	2.135	**2.112**	-1.340	2.143	2.104	2.089	**2.113**
-1.360	2.084	2.132	2.148	**2.112**	-1.330	2.023	2.089	2.087	**2.113**
-1.350	2.119	2.117	2.111	**2.112**	-1.320	2.079	2.066	2.155	**2.113**
-1.340	2.105	2.117	2.096	**2.112**	-1.310	2.098	2.144	2.137	**2.112**
-1.330	2.106	2.103	2.135	**2.111**	-1.300	2.167	2.146	2.122	**2.112**
-1.320	2.094	2.055	2.096	**2.111**	-1.290	2.131	2.159	2.095	**2.112**
-1.310	2.187	2.111	2.143	**2.111**	-1.280	2.115	2.108	2.038	**2.112**
-1.300	2.093	2.068	2.136	**2.111**	-1.270	2.074	2.148	2.115	**2.111**
-1.290	2.141	2.147	2.101	**2.111**	-1.260	2.094	2.161	2.165	**2.111**
-1.280	2.150	2.102	2.163	**2.111**	-1.250	2.134	2.116	2.183	**2.111**
-1.270	2.116	2.100	2.042	**2.110**	-1.240	2.039	2.078	2.047	**2.111**
-1.260	2.118	2.097	2.085	**2.110**	-1.230	2.120	2.138	2.141	**2.111**
-1.250	2.145	2.102	2.095	**2.110**	-1.220	2.054	2.137	2.119	**2.110**

-1.240	2.076	2.041	2.049	**2.109**	-1.210	2.124	2.121	2.095	**2.110**
-1.230	2.193	2.132	2.140	**2.109**	-1.200	2.068	2.109	2.123	**2.110**
-1.220	2.084	2.069	2.107	**2.109**	-1.190	2.165	2.165	2.116	**2.110**
-1.210	2.071	2.071	2.065	**2.109**	-1.180	2.116	2.082	2.119	**2.109**
-1.200	2.136	2.125	2.116	**2.108**	-1.170	2.034	2.054	2.119	**2.109**
-1.190	2.129	2.106	2.089	**2.108**	-1.160	2.076	2.146	2.154	**2.109**
-1.180	2.128	2.134	2.069	**2.108**	-1.150	2.114	2.183	2.104	**2.110**
-1.170	2.017	2.084	2.064	**2.107**	-1.140	2.141	2.063	2.124	**2.110**
-1.160	2.070	2.119	2.052	**2.107**	-1.130	2.122	2.087	2.092	**2.110**
-1.150	2.080	2.088	2.111	**2.107**	-1.120	2.083	2.179	2.140	**2.110**
-1.140	2.158	2.116	2.102	**2.108**	-1.110	2.100	2.102	2.168	**2.110**
-1.130	2.119	2.062	2.126	**2.108**	-1.100	2.075	2.094	2.106	**2.110**
-1.120	2.085	2.130	2.096	**2.108**	-1.090	2.088	2.019	2.111	**2.110**
-1.110	2.074	2.041	2.116	**2.108**	-1.080	2.117	2.101	2.128	**2.110**
-1.100	2.129	2.087	2.055	**2.108**	-1.070	2.083	2.055	2.102	**2.110**
-1.090	2.121	2.109	2.150	**2.108**	-1.060	2.121	2.065	2.055	**2.110**
-1.080	2.138	2.154	2.149	**2.109**	-1.050	2.088	2.025	2.019	**2.110**
-1.070	2.138	2.139	2.156	**2.109**	-1.040	2.176	2.168	2.133	**2.110**
-1.060	2.098	2.107	2.119	**2.109**	-1.030	2.063	2.095	2.088	**2.110**
-1.050	2.152	2.130	2.089	**2.109**	-1.020	2.101	2.099	2.021	**2.110**
-1.040	2.148	2.121	2.070	**2.110**	-1.010	2.119	2.085	2.079	**2.110**
-1.030	2.111	2.065	2.080	**2.110**	-1.000	2.170	2.144	2.075	**2.111**
-1.020	2.166	2.127	2.136	**2.110**	-0.990	2.154	2.093	2.080	**2.111**
-1.010	2.152	2.160	2.122	**2.111**	-0.980	2.110	2.060	2.091	**2.111**
-1.000	2.144	2.080	2.098	**2.111**	-0.970	2.085	2.097	2.105	**2.111**
-0.990	2.097	2.083	2.089	**2.111**	-0.960	2.158	2.152	2.159	**2.111**
-0.980	2.128	2.101	2.116	**2.112**	-0.950	2.094	2.153	2.060	**2.111**
-0.970	2.094	2.113	2.148	**2.112**	-0.940	2.154	2.099	2.196	**2.111**
-0.960	2.105	2.091	2.053	**2.112**	-0.930	2.081	2.069	2.047	**2.111**
-0.950	2.147	2.105	2.105	**2.113**	-0.920	2.049	2.132	2.090	**2.111**
-0.940	2.128	2.136	2.143	**2.113**	-0.910	2.149	2.126	2.079	**2.111**
-0.930	2.134	2.143	2.094	**2.113**	-0.900	2.089	2.068	2.122	**2.111**

-0.920	2.173	2.172	2.146	**2.113**	-0.890	2.071	2.092	2.113	**2.111**
-0.910	2.137	2.118	2.103	**2.113**	-0.880	2.112	2.102	2.044	**2.111**
-0.900	2.093	2.085	2.087	**2.114**	-0.870	2.053	2.133	2.132	**2.111**
-0.890	2.094	2.147	2.121	**2.114**	-0.860	2.115	2.158	2.146	**2.111**
-0.880	2.121	2.115	2.125	**2.114**	-0.850	2.107	2.126	2.148	**2.111**
-0.870	2.117	2.124	2.132	**2.114**	-0.840	2.122	2.115	2.168	**2.111**
-0.860	2.137	2.138	2.117	**2.114**	-0.830	2.108	2.127	2.062	**2.111**
-0.850	2.122	2.076	2.110	**2.114**	-0.820	2.129	2.086	2.124	**2.111**
-0.840	2.162	2.095	2.132	**2.115**	-0.810	2.190	2.142	2.180	**2.110**
-0.830	2.134	2.151	2.120	**2.115**	-0.800	2.169	2.115	2.102	**2.110**
-0.820	2.090	2.083	2.043	**2.115**	-0.790	2.146	2.052	2.104	**2.110**
-0.810	2.114	2.113	2.124	**2.115**	-0.780	2.137	2.138	2.080	**2.110**
-0.800	2.136	2.098	2.153	**2.115**	-0.770	2.121	2.110	2.052	**2.110**
-0.790	2.092	2.062	2.094	**2.115**	-0.760	2.146	2.176	2.154	**2.110**
-0.780	2.082	2.116	2.127	**2.115**	-0.750	2.127	2.112	2.120	**2.110**
-0.770	2.065	2.122	2.090	**2.115**	-0.740	2.175	2.123	2.161	**2.110**
-0.760	2.146	2.081	2.107	**2.115**	-0.730	2.117	2.077	2.108	**2.110**
-0.750	2.090	2.100	2.131	**2.115**	-0.720	2.035	2.076	2.070	**2.110**
-0.740	2.081	2.055	2.071	**2.115**	-0.710	2.099	2.133	2.138	**2.110**
-0.730	2.056	2.129	2.118	**2.115**	-0.700	2.162	2.160	2.088	**2.110**
-0.720	2.140	2.079	2.124	**2.115**	-0.690	2.063	2.062	2.046	**2.110**
-0.710	2.097	2.144	2.143	**2.115**	-0.680	2.175	2.097	2.161	**2.110**
-0.700	2.111	2.120	2.128	**2.115**	-0.670	2.084	2.181	2.127	**2.110**
-0.690	2.125	2.119	2.095	**2.115**	-0.660	2.063	2.150	2.140	**2.110**
-0.680	2.134	2.087	2.118	**2.115**	-0.650	2.032	2.040	2.117	**2.110**
-0.670	2.129	2.152	2.114	**2.115**	-0.640	2.075	2.084	2.148	**2.110**
-0.660	2.074	2.126	2.138	**2.115**	-0.630	2.125	2.163	2.125	**2.110**
-0.650	2.101	2.107	2.120	**2.115**	-0.620	2.184	2.093	2.118	**2.111**
-0.640	2.138	2.134	2.146	**2.115**	-0.610	2.108	2.118	2.056	**2.111**
-0.630	2.108	2.085	2.152	**2.115**	-0.600	2.146	2.079	2.060	**2.111**
-0.620	2.147	2.084	2.140	**2.115**	-0.590	2.136	2.125	2.091	**2.111**
-0.610	2.126	2.064	2.133	**2.115**	-0.580	2.108	2.021	2.078	**2.111**

-0.600	2.166	2.091	2.110	**2.115**
-0.590	2.175	2.151	2.110	**2.116**
-0.580	2.103	2.138	2.139	**2.116**
-0.570	2.133	2.092	2.123	**2.116**
-0.560	2.140	2.179	2.174	**2.116**
-0.550	2.114	2.109	2.107	**2.116**
-0.540	2.197	2.164	2.193	**2.116**
-0.530	2.069	2.126	2.083	**2.116**
-0.520	2.135	2.174	2.143	**2.116**
-0.510	2.064	2.055	2.127	**2.116**
-0.500	2.095	2.082	2.030	**2.116**
-0.490	2.091	2.154	2.094	**2.116**
-0.480	2.120	2.095	2.129	**2.117**
-0.470	2.133	2.078	2.066	**2.117**
-0.460	2.117	2.167	2.140	**2.117**
-0.450	2.140	2.155	2.093	**2.117**
-0.440	2.103	2.116	2.117	**2.117**
-0.430	2.132	2.136	2.105	**2.117**
-0.420	2.160	2.100	2.100	**2.117**
-0.410	2.169	2.137	2.169	**2.117**
-0.400	2.123	2.172	2.117	**2.117**
-0.390	2.090	2.097	2.128	**2.117**
-0.380	2.088	2.054	2.127	**2.117**
-0.370	2.116	2.084	2.143	**2.117**
-0.360	2.063	2.110	2.131	**2.116**
-0.350	2.111	2.123	2.103	**2.116**
-0.340	2.097	2.109	2.121	**2.116**
-0.330	2.098	2.123	2.154	**2.116**
-0.320	2.117	2.194	2.156	**2.116**
-0.310	2.143	2.149	2.079	**2.116**
-0.300	2.119	2.107	2.078	**2.116**
-0.290	2.132	2.084	2.156	**2.116**

-0.570	2.137	2.127	2.150	**2.111**
-0.560	2.104	2.155	2.104	**2.111**
-0.550	2.097	2.038	2.126	**2.112**
-0.540	2.081	2.133	2.128	**2.112**
-0.530	2.079	2.116	2.053	**2.112**
-0.520	2.146	2.161	2.111	**2.112**
-0.510	2.183	2.155	2.167	**2.111**
-0.500	2.113	2.113	2.075	**2.111**
-0.490	2.114	2.141	2.150	**2.111**
-0.480	2.055	2.110	2.136	**2.111**
-0.470	2.092	2.116	2.142	**2.111**
-0.460	2.137	2.109	2.140	**2.111**
-0.450	2.116	2.081	2.025	**2.111**
-0.440	2.090	2.128	2.062	**2.111**
-0.430	2.155	2.116	2.164	**2.111**
-0.420	2.114	2.154	2.091	**2.111**
-0.410	2.091	2.121	2.181	**2.111**
-0.400	2.088	2.075	2.039	**2.111**
-0.390	2.191	2.147	2.132	**2.111**
-0.380	2.146	2.098	2.111	**2.111**
-0.370	2.132	2.097	2.034	**2.111**
-0.360	2.074	2.076	2.057	**2.111**
-0.350	2.084	2.113	2.062	**2.110**

-0.280	2.118	2.114	2.081	**2.117**
-0.270	2.161	2.162	2.124	**2.117**
-0.260	2.140	2.104	2.099	**2.117**
-0.250	2.118	2.141	2.102	**2.117**

Protocol	AT			
Hole nr.	20			
Hole depth (mm)	Meas_1 Radius (mm)	Meas_2 Radius (mm)	Meas_3 Radius (mm)	**Mean Radius (mm)**
-13.870	0.671	0.613	0.680	**0.642**
-13.860	0.684	0.651	0.668	**0.672**
-13.850	0.728	0.746	0.702	**0.704**
-13.840	0.726	0.778	0.802	**0.735**
-13.830	0.735	0.737	0.708	**0.763**
-13.820	0.791	0.755	0.748	**0.789**
-13.810	0.852	0.812	0.854	**0.808**
-13.800	0.834	0.896	0.847	**0.822**
-13.790	0.870	0.881	0.809	**0.844**
-13.780	0.823	0.852	0.855	**0.856**
-13.770	0.905	0.871	0.875	**0.867**
-13.760	0.889	0.885	0.866	**0.877**
-13.750	0.848	0.876	0.871	**0.888**
-13.740	0.933	0.916	0.905	**0.899**
-13.730	0.940	0.929	0.915	**0.910**
-13.720	0.953	0.940	0.974	**0.921**
-13.710	0.950	0.935	0.920	**0.932**
-13.700	0.923	0.940	0.883	**0.943**
-13.690	0.942	1.008	0.946	**0.953**
-13.680	0.997	0.942	0.927	**0.964**
-13.670	0.967	0.946	0.995	**0.975**
-13.660	0.947	0.956	0.995	**0.986**
-13.650	0.972	0.943	0.989	**0.997**
-13.640	0.978	0.991	0.941	**1.008**
-13.630	1.055	1.010	1.024	**1.019**
-13.620	1.026	1.074	1.052	**1.029**
-13.610	1.047	1.041	1.029	**1.040**

Protocol	AT			
Hole nr.	30			
Hole depth (mm)	Meas_1 Radius (mm)	Meas_2 Radius (mm)	Meas_3 Radius (mm)	**Mean Radius (mm)**
-13.990	0.313	0.339	0.322	**0.328**
-13.980	0.355	0.343	0.361	**0.370**
-13.970	0.459	0.485	0.418	**0.406**
-13.960	0.374	0.458	0.404	**0.438**
-13.950	0.421	0.485	0.425	**0.467**
-13.940	0.447	0.529	0.500	**0.494**
-13.930	0.473	0.391	0.491	**0.519**
-13.920	0.510	0.595	0.547	**0.544**
-13.910	0.527	0.596	0.593	**0.569**
-13.900	0.582	0.661	0.627	**0.594**
-13.890	0.695	0.736	0.628	**0.619**
-13.880	0.633	0.729	0.660	**0.642**
-13.870	0.626	0.660	0.671	**0.665**
-13.860	0.638	0.686	0.633	**0.692**
-13.850	0.730	0.776	0.741	**0.718**
-13.840	0.698	0.784	0.782	**0.746**
-13.830	0.734	0.709	0.748	**0.778**
-13.820	0.917	0.868	0.884	**0.811**
-13.810	0.939	0.855	0.860	**0.841**
-13.800	0.834	0.800	0.900	**0.866**
-13.790	0.890	0.891	0.977	**0.887**
-13.780	0.926	1.010	0.902	**0.905**
-13.770	0.850	0.877	0.941	**0.920**
-13.760	1.035	1.019	0.943	**0.935**
-13.750	0.903	0.942	0.922	**0.950**
-13.740	1.012	0.977	0.984	**0.965**
-13.730	1.055	0.982	1.064	**0.981**

-13.600	1.107	1.052	1.080	**1.051**	-13.720	1.034	1.025	1.023	**0.994**
-13.590	1.055	1.050	1.121	**1.062**	-13.710	0.987	1.033	0.985	**1.007**
-13.580	1.017	1.068	1.036	**1.073**	-13.700	1.110	1.019	1.085	**1.020**
-13.570	1.100	1.124	1.046	**1.084**	-13.690	1.064	1.091	1.056	**1.033**
-13.560	1.031	1.094	1.061	**1.095**	-13.680	1.011	1.042	1.017	**1.046**
-13.550	1.107	1.104	1.083	**1.105**	-13.670	1.074	1.137	1.126	**1.059**
-13.540	1.075	1.104	1.136	**1.116**	-13.660	1.121	1.075	1.074	**1.072**
-13.530	1.140	1.169	1.102	**1.127**	-13.650	0.989	1.055	1.073	**1.085**
-13.520	1.101	1.145	1.120	**1.138**	-13.640	1.155	1.129	1.079	**1.098**
-13.510	1.153	1.126	1.110	**1.149**	-13.630	1.084	1.063	1.102	**1.108**
-13.500	1.203	1.196	1.228	**1.160**	-13.620	1.215	1.204	1.128	**1.118**
-13.490	1.197	1.149	1.160	**1.171**	-13.610	1.178	1.181	1.080	**1.128**
-13.480	1.195	1.228	1.173	**1.181**	-13.600	1.189	1.162	1.146	**1.138**
-13.470	1.192	1.229	1.209	**1.192**	-13.590	1.209	1.164	1.213	**1.149**
-13.460	1.200	1.208	1.220	**1.203**	-13.580	1.182	1.127	1.204	**1.159**
-13.450	1.221	1.212	1.238	**1.214**	-13.570	1.194	1.185	1.195	**1.169**
-13.440	1.224	1.210	1.234	**1.225**	-13.560	1.278	1.218	1.173	**1.179**
-13.430	1.242	1.200	1.273	**1.236**	-13.550	1.233	1.173	1.164	**1.189**
-13.420	1.220	1.212	1.266	**1.247**	-13.540	1.277	1.173	1.239	**1.199**
-13.410	1.238	1.245	1.199	**1.257**	-13.530	1.189	1.239	1.139	**1.209**
-13.400	1.243	1.293	1.259	**1.268**	-13.520	1.208	1.184	1.202	**1.219**
-13.390	1.277	1.239	1.212	**1.279**	-13.510	1.242	1.240	1.198	**1.229**
-13.380	1.257	1.269	1.273	**1.290**	-13.500	1.214	1.168	1.232	**1.239**
-13.370	1.344	1.289	1.353	**1.301**	-13.490	1.201	1.290	1.257	**1.249**
-13.360	1.316	1.295	1.303	**1.312**	-13.480	1.233	1.306	1.242	**1.259**
-13.350	1.305	1.328	1.289	**1.323**	-13.470	1.334	1.262	1.304	**1.269**
-13.340	1.339	1.321	1.352	**1.333**	-13.460	1.242	1.342	1.295	**1.279**
-13.330	1.298	1.298	1.307	**1.344**	-13.450	1.328	1.218	1.275	**1.289**
-13.320	1.325	1.321	1.333	**1.355**	-13.440	1.310	1.299	1.241	**1.299**
-13.310	1.302	1.330	1.283	**1.366**	-13.430	1.289	1.382	1.313	**1.309**
-13.300	1.372	1.347	1.409	**1.377**	-13.420	1.335	1.341	1.347	**1.319**
-13.290	1.424	1.415	1.372	**1.388**	-13.410	1.457	1.387	1.350	**1.329**

-13.280	1.385	1.359	1.428	**1.399**	-13.400	1.361	1.359	1.376	**1.339**
-13.270	1.453	1.401	1.426	**1.409**	-13.390	1.401	1.374	1.304	**1.349**
-13.260	1.413	1.338	1.399	**1.420**	-13.380	1.465	1.411	1.373	**1.359**
-13.250	1.422	1.370	1.354	**1.430**	-13.370	1.406	1.410	1.462	**1.369**
-13.240	1.470	1.456	1.409	**1.440**	-13.360	1.373	1.340	1.379	**1.379**
-13.230	1.426	1.447	1.469	**1.450**	-13.350	1.422	1.345	1.373	**1.390**
-13.220	1.460	1.511	1.496	**1.460**	-13.340	1.439	1.415	1.399	**1.400**
-13.210	1.487	1.468	1.455	**1.469**	-13.330	1.426	1.337	1.343	**1.410**
-13.200	1.522	1.497	1.471	**1.477**	-13.320	1.396	1.423	1.387	**1.420**
-13.190	1.518	1.510	1.514	**1.484**	-13.310	1.377	1.399	1.477	**1.427**
-13.180	1.539	1.474	1.467	**1.492**	-13.300	1.337	1.348	1.362	**1.434**
-13.170	1.495	1.520	1.513	**1.499**	-13.290	1.489	1.401	1.461	**1.441**
-13.160	1.548	1.472	1.500	**1.506**	-13.280	1.345	1.400	1.448	**1.448**
-13.150	1.491	1.474	1.495	**1.514**	-13.270	1.479	1.426	1.488	**1.455**
-13.140	1.508	1.493	1.441	**1.521**	-13.260	1.439	1.493	1.404	**1.462**
-13.130	1.496	1.525	1.489	**1.528**	-13.250	1.572	1.469	1.485	**1.468**
-13.120	1.512	1.521	1.541	**1.535**	-13.240	1.480	1.461	1.450	**1.474**
-13.110	1.541	1.514	1.491	**1.538**	-13.230	1.438	1.442	1.491	**1.481**
-13.100	1.493	1.558	1.523	**1.542**	-13.220	1.473	1.412	1.415	**1.487**
-13.090	1.567	1.565	1.580	**1.543**	-13.210	1.487	1.475	1.470	**1.492**
-13.080	1.570	1.533	1.548	**1.545**	-13.200	1.523	1.512	1.483	**1.496**
-13.070	1.558	1.543	1.506	**1.546**	-13.190	1.462	1.451	1.490	**1.500**
-13.060	1.501	1.552	1.532	**1.547**	-13.180	1.480	1.479	1.486	**1.503**
-13.050	1.612	1.572	1.568	**1.548**	-13.170	1.452	1.460	1.455	**1.507**
-13.040	1.523	1.589	1.517	**1.549**	-13.160	1.515	1.578	1.608	**1.511**
-13.030	1.522	1.543	1.544	**1.550**	-13.150	1.533	1.476	1.555	**1.515**
-13.020	1.563	1.562	1.615	**1.550**	-13.140	1.532	1.566	1.530	**1.518**
-13.010	1.548	1.561	1.574	**1.551**	-13.130	1.487	1.484	1.500	**1.522**
-13.000	1.561	1.541	1.575	**1.551**	-13.120	1.567	1.514	1.572	**1.525**
-12.990	1.515	1.537	1.572	**1.552**	-13.110	1.591	1.497	1.584	**1.528**
-12.980	1.553	1.578	1.532	**1.552**	-13.100	1.453	1.460	1.383	**1.530**
-12.970	1.520	1.554	1.545	**1.553**	-13.090	1.519	1.609	1.572	**1.533**

-12.960	1.495	1.552	1.550	**1.553**	-13.080	1.534	1.511	1.578	**1.535**
-12.950	1.491	1.470	1.532	**1.553**	-13.070	1.492	1.491	1.562	**1.538**
-12.940	1.618	1.570	1.633	**1.553**	-13.060	1.514	1.477	1.554	**1.540**
-12.930	1.514	1.556	1.508	**1.553**	-13.050	1.557	1.491	1.553	**1.543**
-12.920	1.528	1.501	1.559	**1.554**	-13.040	1.581	1.540	1.526	**1.545**
-12.910	1.545	1.556	1.535	**1.554**	-13.030	1.591	1.551	1.505	**1.547**
-12.900	1.598	1.557	1.578	**1.554**	-13.020	1.496	1.482	1.498	**1.548**
-12.890	1.565	1.600	1.573	**1.554**	-13.010	1.541	1.562	1.462	**1.550**
-12.880	1.504	1.571	1.552	**1.554**	-13.000	1.500	1.508	1.572	**1.551**
-12.870	1.626	1.575	1.593	**1.554**	-12.990	1.433	1.438	1.487	**1.552**
-12.860	1.574	1.536	1.583	**1.554**	-12.980	1.596	1.563	1.514	**1.553**
-12.850	1.587	1.592	1.546	**1.555**	-12.970	1.488	1.461	1.518	**1.555**
-12.840	1.595	1.569	1.518	**1.555**	-12.960	1.570	1.522	1.614	**1.556**
-12.830	1.530	1.594	1.594	**1.555**	-12.950	1.552	1.530	1.530	**1.557**
-12.820	1.593	1.537	1.593	**1.555**	-12.940	1.575	1.649	1.589	**1.558**
-12.810	1.503	1.515	1.560	**1.555**	-12.930	1.524	1.596	1.494	**1.559**
-12.800	1.549	1.537	1.593	**1.555**	-12.920	1.572	1.509	1.483	**1.560**
-12.790	1.588	1.532	1.532	**1.556**	-12.910	1.633	1.619	1.536	**1.560**
-12.780	1.608	1.577	1.537	**1.556**	-12.900	1.515	1.537	1.549	**1.561**
-12.770	1.504	1.565	1.535	**1.556**	-12.890	1.545	1.440	1.517	**1.562**
-12.760	1.512	1.552	1.556	**1.556**	-12.880	1.573	1.507	1.473	**1.562**
-12.750	1.560	1.531	1.560	**1.556**	-12.870	1.503	1.573	1.539	**1.563**
-12.740	1.558	1.552	1.560	**1.556**	-12.860	1.581	1.537	1.580	**1.563**
-12.730	1.542	1.610	1.578	**1.556**	-12.850	1.605	1.540	1.494	**1.563**
-12.720	1.594	1.549	1.558	**1.556**	-12.840	1.562	1.485	1.545	**1.563**
-12.710	1.627	1.611	1.568	**1.555**	-12.830	1.645	1.624	1.562	**1.563**
-12.700	1.563	1.617	1.581	**1.555**	-12.820	1.617	1.585	1.510	**1.564**
-12.690	1.556	1.561	1.576	**1.554**	-12.810	1.599	1.597	1.512	**1.564**
-12.680	1.531	1.537	1.523	**1.554**	-12.800	1.556	1.557	1.604	**1.564**
-12.670	1.567	1.546	1.572	**1.553**	-12.790	1.579	1.604	1.631	**1.564**
-12.660	1.564	1.611	1.594	**1.552**	-12.780	1.570	1.550	1.482	**1.564**
-12.650	1.552	1.573	1.549	**1.552**	-12.770	1.534	1.505	1.458	**1.564**

-12.640	1.539	1.513	1.576	**1.551**	-12.760	1.586	1.671	1.630	**1.564**
-12.630	1.579	1.541	1.544	**1.550**	-12.750	1.601	1.518	1.619	**1.564**
-12.620	1.545	1.571	1.572	**1.549**	-12.740	1.568	1.562	1.557	**1.564**
-12.610	1.581	1.561	1.537	**1.549**	-12.730	1.540	1.529	1.548	**1.564**
-12.600	1.525	1.601	1.574	**1.548**	-12.720	1.593	1.478	1.533	**1.564**
-12.590	1.585	1.603	1.552	**1.547**	-12.710	1.586	1.564	1.666	**1.564**
-12.580	1.534	1.556	1.571	**1.546**	-12.700	1.545	1.592	1.615	**1.564**
-12.570	1.528	1.566	1.579	**1.546**	-12.690	1.541	1.579	1.593	**1.563**
-12.560	1.548	1.520	1.532	**1.545**	-12.680	1.603	1.576	1.561	**1.563**
-12.550	1.519	1.555	1.571	**1.544**	-12.670	1.584	1.620	1.555	**1.564**
-12.540	1.528	1.600	1.535	**1.544**	-12.660	1.552	1.564	1.540	**1.564**
-12.530	1.588	1.530	1.528	**1.543**	-12.650	1.555	1.578	1.592	**1.564**
-12.520	1.572	1.495	1.525	**1.543**	-12.640	1.538	1.483	1.578	**1.564**
-12.510	1.542	1.569	1.501	**1.544**	-12.630	1.573	1.550	1.653	**1.564**
-12.500	1.514	1.551	1.526	**1.544**	-12.620	1.530	1.538	1.622	**1.564**
-12.490	1.518	1.529	1.573	**1.545**	-12.610	1.604	1.611	1.580	**1.564**
-12.480	1.564	1.505	1.580	**1.546**	-12.600	1.547	1.509	1.522	**1.564**
-12.470	1.574	1.502	1.531	**1.547**	-12.590	1.517	1.596	1.582	**1.564**
-12.460	1.519	1.530	1.556	**1.548**	-12.580	1.587	1.598	1.512	**1.564**
-12.450	1.526	1.531	1.562	**1.549**	-12.570	1.500	1.589	1.567	**1.564**
-12.440	1.580	1.552	1.509	**1.549**	-12.560	1.582	1.645	1.541	**1.564**
-12.430	1.520	1.582	1.546	**1.550**	-12.550	1.551	1.625	1.530	**1.564**
-12.420	1.565	1.590	1.579	**1.551**	-12.540	1.588	1.594	1.491	**1.564**
-12.410	1.576	1.527	1.565	**1.552**	-12.530	1.522	1.494	1.547	**1.564**
-12.400	1.494	1.511	1.551	**1.553**	-12.520	1.485	1.558	1.582	**1.563**
-12.390	1.589	1.554	1.565	**1.554**	-12.510	1.552	1.566	1.495	**1.563**
-12.380	1.516	1.541	1.575	**1.555**	-12.500	1.586	1.549	1.643	**1.563**
-12.370	1.609	1.600	1.568	**1.556**	-12.490	1.584	1.514	1.619	**1.562**
-12.360	1.605	1.585	1.572	**1.556**	-12.480	1.632	1.577	1.569	**1.562**
-12.350	1.558	1.540	1.529	**1.557**	-12.470	1.589	1.538	1.520	**1.561**
-12.340	1.604	1.542	1.587	**1.558**	-12.460	1.489	1.488	1.497	**1.561**
-12.330	1.599	1.575	1.537	**1.558**	-12.450	1.639	1.644	1.630	**1.560**

-12.320	1.567	1.549	1.556	**1.559**	-12.440	1.606	1.630	1.536	**1.560**
-12.310	1.611	1.600	1.556	**1.560**	-12.430	1.545	1.612	1.536	**1.559**
-12.300	1.524	1.552	1.568	**1.560**	-12.420	1.599	1.606	1.551	**1.558**
-12.290	1.502	1.545	1.561	**1.561**	-12.410	1.604	1.501	1.517	**1.558**
-12.280	1.585	1.599	1.609	**1.560**	-12.400	1.593	1.536	1.542	**1.558**
-12.270	1.489	1.534	1.559	**1.560**	-12.390	1.603	1.636	1.608	**1.558**
-12.260	1.548	1.574	1.566	**1.560**	-12.380	1.522	1.584	1.536	**1.558**
-12.250	1.617	1.578	1.570	**1.560**	-12.370	1.531	1.526	1.543	**1.558**
-12.240	1.541	1.549	1.610	**1.559**	-12.360	1.634	1.536	1.536	**1.558**
-12.230	1.641	1.578	1.596	**1.559**	-12.350	1.604	1.610	1.546	**1.557**
-12.220	1.565	1.581	1.511	**1.559**	-12.340	1.548	1.624	1.588	**1.557**
-12.210	1.589	1.550	1.534	**1.558**	-12.330	1.547	1.537	1.633	**1.557**
-12.200	1.599	1.563	1.545	**1.558**	-12.320	1.514	1.487	1.506	**1.557**
-12.190	1.544	1.543	1.597	**1.557**	-12.310	1.531	1.588	1.597	**1.557**
-12.180	1.486	1.538	1.479	**1.557**	-12.300	1.650	1.607	1.630	**1.558**
-12.170	1.547	1.532	1.590	**1.556**	-12.290	1.584	1.645	1.529	**1.558**
-12.160	1.491	1.557	1.537	**1.556**	-12.280	1.571	1.542	1.532	**1.559**
-12.150	1.625	1.600	1.556	**1.555**	-12.270	1.594	1.594	1.531	**1.559**
-12.140	1.546	1.473	1.530	**1.554**	-12.260	1.548	1.505	1.536	**1.559**
-12.130	1.536	1.490	1.565	**1.554**	-12.250	1.675	1.656	1.612	**1.559**
-12.120	1.524	1.566	1.548	**1.553**	-12.240	1.535	1.602	1.614	**1.559**
-12.110	1.575	1.535	1.588	**1.552**	-12.230	1.636	1.539	1.550	**1.559**
-12.100	1.556	1.579	1.516	**1.552**	-12.220	1.599	1.593	1.605	**1.559**
-12.090	1.506	1.511	1.515	**1.551**	-12.210	1.560	1.605	1.566	**1.559**
-12.080	1.540	1.569	1.592	**1.551**	-12.200	1.624	1.578	1.574	**1.559**
-12.070	1.590	1.574	1.574	**1.550**	-12.190	1.578	1.578	1.540	**1.559**
-12.060	1.539	1.525	1.546	**1.549**	-12.180	1.558	1.594	1.586	**1.559**
-12.050	1.611	1.579	1.569	**1.548**	-12.170	1.519	1.560	1.467	**1.559**
-12.040	1.511	1.449	1.514	**1.546**	-12.160	1.496	1.540	1.576	**1.558**
-12.030	1.557	1.529	1.505	**1.545**	-12.150	1.621	1.608	1.623	**1.558**
-12.020	1.522	1.572	1.543	**1.544**	-12.140	1.533	1.532	1.549	**1.557**
-12.010	1.548	1.501	1.563	**1.542**	-12.130	1.552	1.594	1.553	**1.556**

-12.000	1.565	1.522	1.529	**1.542**
-11.990	1.608	1.571	1.533	**1.543**
-11.980	1.582	1.504	1.545	**1.543**
-11.970	1.578	1.529	1.533	**1.543**
-11.960	1.508	1.555	1.531	**1.543**
-11.950	1.535	1.612	1.563	**1.543**
-11.940	1.471	1.518	1.503	**1.543**
-11.930	1.526	1.578	1.557	**1.544**
-11.920	1.495	1.572	1.519	**1.544**
-11.910	1.553	1.536	1.525	**1.544**
-11.900	1.532	1.582	1.591	**1.545**
-11.890	1.533	1.467	1.529	**1.545**
-11.880	1.542	1.515	1.561	**1.546**
-11.870	1.498	1.539	1.474	**1.546**
-11.860	1.549	1.537	1.476	**1.546**
-11.850	1.539	1.551	1.511	**1.547**
-11.840	1.532	1.508	1.518	**1.547**
-11.830	1.541	1.535	1.568	**1.547**
-11.820	1.507	1.563	1.583	**1.547**
-11.810	1.537	1.530	1.565	**1.547**
-11.800	1.504	1.521	1.579	**1.547**
-11.790	1.507	1.524	1.556	**1.547**
-11.780	1.500	1.556	1.545	**1.547**
-11.770	1.582	1.527	1.589	**1.547**
-11.760	1.571	1.530	1.577	**1.547**
-11.750	1.544	1.560	1.520	**1.547**
-11.740	1.577	1.544	1.578	**1.547**
-11.730	1.506	1.510	1.503	**1.547**
-11.720	1.513	1.544	1.517	**1.547**
-11.710	1.585	1.560	1.592	**1.547**
-11.700	1.566	1.574	1.571	**1.548**
-11.690	1.509	1.520	1.545	**1.548**

-12.120	1.494	1.495	1.586	**1.556**
-12.110	1.550	1.558	1.605	**1.555**
-12.100	1.577	1.558	1.563	**1.554**
-12.090	1.489	1.546	1.592	**1.554**
-12.080	1.514	1.553	1.614	**1.553**
-12.070	1.540	1.615	1.520	**1.552**
-12.060	1.560	1.493	1.476	**1.551**
-12.050	1.576	1.536	1.517	**1.550**
-12.040	1.561	1.555	1.624	**1.549**
-12.030	1.634	1.568	1.613	**1.549**
-12.020	1.525	1.595	1.537	**1.548**
-12.010	1.597	1.572	1.491	**1.547**
-12.000	1.592	1.523	1.557	**1.547**
-11.990	1.499	1.570	1.588	**1.547**
-11.980	1.563	1.565	1.634	**1.547**
-11.970	1.544	1.486	1.573	**1.547**
-11.960	1.638	1.620	1.664	**1.547**
-11.950	1.524	1.495	1.571	**1.548**
-11.940	1.586	1.581	1.566	**1.548**
-11.930	1.538	1.600	1.522	**1.548**
-11.920	1.576	1.537	1.536	**1.549**
-11.910	1.527	1.496	1.602	**1.549**
-11.900	1.503	1.514	1.464	**1.549**
-11.890	1.506	1.520	1.573	**1.549**
-11.880	1.528	1.640	1.586	**1.550**
-11.870	1.572	1.571	1.604	**1.550**
-11.860	1.525	1.491	1.518	**1.550**
-11.850	1.545	1.510	1.540	**1.551**
-11.840	1.554	1.481	1.535	**1.551**
-11.830	1.510	1.575	1.527	**1.551**
-11.820	1.545	1.629	1.524	**1.552**
-11.810	1.616	1.540	1.570	**1.552**

-11.680	1.541	1.581	1.536	**1.548**	-11.800	1.516	1.530	1.539	**1.552**
-11.670	1.506	1.562	1.558	**1.548**	-11.790	1.557	1.537	1.580	**1.553**
-11.660	1.648	1.604	1.572	**1.548**	-11.780	1.549	1.542	1.524	**1.553**
-11.650	1.594	1.554	1.613	**1.549**	-11.770	1.592	1.567	1.485	**1.553**
-11.640	1.589	1.583	1.535	**1.549**	-11.760	1.656	1.547	1.574	**1.553**
-11.630	1.584	1.533	1.560	**1.549**	-11.750	1.520	1.582	1.516	**1.554**
-11.620	1.544	1.534	1.603	**1.550**	-11.740	1.658	1.594	1.558	**1.554**
-11.610	1.523	1.549	1.501	**1.550**	-11.730	1.552	1.524	1.628	**1.554**
-11.600	1.564	1.572	1.574	**1.551**	-11.720	1.557	1.611	1.577	**1.554**
-11.590	1.551	1.576	1.531	**1.551**	-11.710	1.576	1.637	1.586	**1.555**
-11.580	1.565	1.508	1.575	**1.551**	-11.700	1.600	1.516	1.561	**1.555**
-11.570	1.568	1.490	1.539	**1.551**	-11.690	1.558	1.578	1.556	**1.555**
-11.560	1.488	1.509	1.485	**1.551**	-11.680	1.521	1.545	1.503	**1.555**
-11.550	1.566	1.514	1.555	**1.551**	-11.670	1.587	1.541	1.524	**1.556**
-11.540	1.576	1.538	1.576	**1.551**	-11.660	1.602	1.643	1.541	**1.556**
-11.530	1.543	1.540	1.565	**1.551**	-11.650	1.495	1.574	1.512	**1.556**
-11.520	1.556	1.534	1.537	**1.551**	-11.640	1.599	1.663	1.563	**1.556**
-11.510	1.569	1.532	1.556	**1.551**	-11.630	1.554	1.509	1.529	**1.557**
-11.500	1.626	1.558	1.583	**1.551**	-11.620	1.566	1.644	1.541	**1.557**
-11.490	1.579	1.580	1.627	**1.551**	-11.610	1.619	1.629	1.608	**1.557**
-11.480	1.542	1.543	1.505	**1.551**	-11.600	1.533	1.515	1.572	**1.557**
-11.470	1.508	1.560	1.492	**1.551**	-11.590	1.594	1.517	1.628	**1.557**
-11.460	1.589	1.553	1.565	**1.552**	-11.580	1.579	1.546	1.500	**1.557**
-11.450	1.517	1.525	1.555	**1.554**	-11.570	1.504	1.538	1.611	**1.557**
-11.440	1.558	1.512	1.504	**1.555**	-11.560	1.606	1.545	1.579	**1.557**
-11.430	1.527	1.541	1.555	**1.556**	-11.550	1.681	1.579	1.622	**1.556**
-11.420	1.504	1.528	1.537	**1.556**	-11.540	1.498	1.474	1.463	**1.556**
-11.410	1.552	1.598	1.548	**1.557**	-11.530	1.549	1.481	1.524	**1.556**
-11.400	1.619	1.600	1.552	**1.557**	-11.520	1.547	1.560	1.503	**1.556**
-11.390	1.611	1.553	1.565	**1.557**	-11.510	1.592	1.596	1.540	**1.556**
-11.380	1.555	1.539	1.567	**1.556**	-11.500	1.546	1.539	1.559	**1.556**
-11.370	1.566	1.515	1.562	**1.556**	-11.490	1.620	1.533	1.607	**1.556**

-11.360	1.568	1.541	1.548	**1.556**	-11.480	1.496	1.409	1.514	**1.556**
-11.350	1.582	1.534	1.599	**1.555**	-11.470	1.587	1.543	1.593	**1.555**
-11.340	1.565	1.575	1.507	**1.555**	-11.460	1.609	1.603	1.639	**1.555**
-11.330	1.561	1.568	1.500	**1.555**	-11.450	1.531	1.634	1.570	**1.555**
-11.320	1.571	1.548	1.596	**1.556**	-11.440	1.541	1.541	1.484	**1.556**
-11.310	1.552	1.548	1.563	**1.557**	-11.430	1.593	1.702	1.637	**1.556**
-11.300	1.557	1.565	1.510	**1.558**	-11.420	1.575	1.537	1.497	**1.557**
-11.290	1.557	1.573	1.563	**1.559**	-11.410	1.495	1.559	1.555	**1.557**
-11.280	1.556	1.515	1.556	**1.560**	-11.400	1.459	1.548	1.466	**1.557**
-11.270	1.523	1.527	1.553	**1.562**	-11.390	1.616	1.550	1.621	**1.557**
-11.260	1.552	1.604	1.557	**1.563**	-11.380	1.567	1.613	1.580	**1.558**
-11.250	1.583	1.588	1.582	**1.564**	-11.370	1.605	1.504	1.582	**1.558**
-11.240	1.535	1.566	1.534	**1.565**	-11.360	1.547	1.510	1.548	**1.558**
-11.230	1.572	1.549	1.595	**1.567**	-11.350	1.572	1.570	1.578	**1.559**
-11.220	1.560	1.549	1.576	**1.568**	-11.340	1.529	1.557	1.547	**1.560**
-11.210	1.517	1.560	1.522	**1.569**	-11.330	1.515	1.498	1.580	**1.561**
-11.200	1.549	1.613	1.579	**1.570**	-11.320	1.593	1.523	1.526	**1.562**
-11.190	1.554	1.588	1.584	**1.572**	-11.310	1.562	1.534	1.624	**1.564**
-11.180	1.575	1.639	1.571	**1.573**	-11.300	1.605	1.500	1.574	**1.565**
-11.170	1.624	1.599	1.575	**1.575**	-11.290	1.554	1.527	1.564	**1.567**
-11.160	1.513	1.548	1.563	**1.577**	-11.280	1.506	1.571	1.582	**1.568**
-11.150	1.619	1.617	1.591	**1.580**	-11.270	1.631	1.624	1.530	**1.570**
-11.140	1.579	1.603	1.560	**1.582**	-11.260	1.542	1.549	1.552	**1.572**
-11.130	1.579	1.524	1.596	**1.584**	-11.250	1.571	1.574	1.555	**1.574**
-11.120	1.578	1.585	1.635	**1.588**	-11.240	1.552	1.631	1.577	**1.576**
-11.110	1.606	1.610	1.562	**1.593**	-11.230	1.635	1.582	1.521	**1.578**
-11.100	1.598	1.641	1.627	**1.599**	-11.220	1.530	1.613	1.584	**1.580**
-11.090	1.619	1.579	1.587	**1.604**	-11.210	1.620	1.582	1.534	**1.582**
-11.080	1.616	1.664	1.598	**1.609**	-11.200	1.571	1.547	1.549	**1.584**
-11.070	1.622	1.683	1.658	**1.615**	-11.190	1.601	1.625	1.608	**1.587**
-11.060	1.619	1.616	1.565	**1.620**	-11.180	1.487	1.495	1.519	**1.589**
-11.050	1.605	1.591	1.611	**1.625**	-11.170	1.636	1.705	1.628	**1.591**

-11.040	1.676	1.649	1.637	**1.630**	-11.160	1.578	1.571	1.535	**1.593**
-11.030	1.593	1.664	1.605	**1.636**	-11.150	1.643	1.684	1.719	**1.595**
-11.020	1.660	1.586	1.636	**1.641**	-11.140	1.698	1.635	1.644	**1.597**
-11.010	1.696	1.634	1.662	**1.646**	-11.130	1.665	1.606	1.657	**1.599**
-11.000	1.649	1.635	1.662	**1.652**	-11.120	1.585	1.564	1.564	**1.601**
-10.990	1.637	1.680	1.629	**1.657**	-11.110	1.552	1.649	1.571	**1.603**
-10.980	1.630	1.645	1.701	**1.662**	-11.100	1.561	1.642	1.602	**1.605**
-10.970	1.687	1.704	1.754	**1.667**	-11.090	1.610	1.610	1.613	**1.607**
-10.960	1.659	1.651	1.707	**1.673**	-11.080	1.604	1.637	1.587	**1.609**
-10.950	1.671	1.702	1.708	**1.678**	-11.070	1.550	1.550	1.557	**1.612**
-10.940	1.677	1.623	1.658	**1.683**	-11.060	1.674	1.639	1.638	**1.616**
-10.930	1.721	1.745	1.675	**1.689**	-11.050	1.558	1.595	1.551	**1.620**
-10.920	1.655	1.646	1.605	**1.694**	-11.040	1.647	1.615	1.583	**1.624**
-10.910	1.666	1.676	1.736	**1.699**	-11.030	1.699	1.677	1.637	**1.627**
-10.900	1.660	1.695	1.712	**1.705**	-11.020	1.684	1.577	1.609	**1.631**
-10.890	1.717	1.717	1.727	**1.710**	-11.010	1.641	1.617	1.548	**1.635**
-10.880	1.738	1.702	1.672	**1.715**	-11.000	1.675	1.688	1.660	**1.639**
-10.870	1.714	1.666	1.655	**1.720**	-10.990	1.631	1.677	1.604	**1.643**
-10.860	1.698	1.674	1.713	**1.726**	-10.980	1.700	1.754	1.758	**1.647**
-10.850	1.779	1.737	1.746	**1.731**	-10.970	1.641	1.590	1.582	**1.651**
-10.840	1.726	1.691	1.685	**1.735**	-10.960	1.693	1.714	1.689	**1.655**
-10.830	1.712	1.748	1.670	**1.739**	-10.950	1.727	1.656	1.657	**1.659**
-10.820	1.728	1.752	1.760	**1.744**	-10.940	1.590	1.569	1.587	**1.663**
-10.810	1.805	1.766	1.731	**1.748**	-10.930	1.656	1.661	1.619	**1.667**
-10.800	1.722	1.712	1.720	**1.753**	-10.920	1.762	1.688	1.684	**1.671**
-10.790	1.768	1.768	1.824	**1.757**	-10.910	1.611	1.653	1.671	**1.675**
-10.780	1.763	1.782	1.733	**1.761**	-10.900	1.667	1.651	1.722	**1.679**
-10.770	1.781	1.789	1.746	**1.765**	-10.890	1.648	1.641	1.642	**1.683**
-10.760	1.765	1.772	1.709	**1.769**	-10.880	1.640	1.746	1.690	**1.687**
-10.750	1.756	1.759	1.787	**1.772**	-10.870	1.741	1.756	1.698	**1.691**
-10.740	1.830	1.845	1.797	**1.776**	-10.860	1.694	1.676	1.692	**1.694**
-10.730	1.798	1.764	1.797	**1.779**	-10.850	1.716	1.762	1.700	**1.698**

-10.720	1.862	1.802	1.808	**1.783**	-10.840	1.728	1.625	1.657	**1.702**
-10.710	1.757	1.799	1.761	**1.786**	-10.830	1.842	1.736	1.764	**1.707**
-10.700	1.763	1.754	1.776	**1.790**	-10.820	1.709	1.728	1.733	**1.711**
-10.690	1.818	1.759	1.828	**1.794**	-10.810	1.731	1.756	1.753	**1.715**
-10.680	1.850	1.777	1.796	**1.797**	-10.800	1.742	1.729	1.655	**1.720**
-10.670	1.861	1.790	1.824	**1.801**	-10.790	1.764	1.718	1.772	**1.724**
-10.660	1.762	1.802	1.775	**1.804**	-10.780	1.735	1.709	1.688	**1.729**
-10.650	1.852	1.779	1.827	**1.808**	-10.770	1.670	1.703	1.780	**1.733**
-10.640	1.795	1.800	1.792	**1.811**	-10.760	1.736	1.716	1.790	**1.738**
-10.630	1.846	1.823	1.841	**1.815**	-10.750	1.751	1.743	1.766	**1.742**
-10.620	1.822	1.802	1.814	**1.818**	-10.740	1.680	1.744	1.726	**1.747**
-10.610	1.815	1.790	1.757	**1.821**	-10.730	1.752	1.707	1.813	**1.751**
-10.600	1.850	1.849	1.854	**1.824**	-10.720	1.812	1.719	1.801	**1.756**
-10.590	1.860	1.808	1.841	**1.827**	-10.710	1.728	1.764	1.655	**1.760**
-10.580	1.896	1.915	1.891	**1.830**	-10.700	1.721	1.775	1.816	**1.764**
-10.570	1.859	1.815	1.856	**1.832**	-10.690	1.750	1.808	1.766	**1.769**
-10.560	1.810	1.879	1.842	**1.834**	-10.680	1.794	1.881	1.775	**1.773**
-10.550	1.799	1.840	1.872	**1.836**	-10.670	1.863	1.790	1.829	**1.778**
-10.540	1.851	1.870	1.849	**1.838**	-10.660	1.855	1.823	1.827	**1.782**
-10.530	1.824	1.883	1.875	**1.840**	-10.650	1.799	1.850	1.906	**1.787**
-10.520	1.806	1.850	1.827	**1.841**	-10.640	1.788	1.857	1.865	**1.791**
-10.510	1.849	1.797	1.850	**1.842**	-10.630	1.792	1.727	1.828	**1.796**
-10.500	1.838	1.886	1.816	**1.843**	-10.620	1.813	1.712	1.810	**1.800**
-10.490	1.881	1.864	1.915	**1.844**	-10.610	1.819	1.772	1.742	**1.804**
-10.480	1.842	1.900	1.836	**1.845**	-10.600	1.849	1.773	1.880	**1.809**
-10.470	1.848	1.870	1.813	**1.846**	-10.590	1.866	1.767	1.826	**1.813**
-10.460	1.815	1.838	1.777	**1.846**	-10.580	1.832	1.871	1.853	**1.818**
-10.450	1.803	1.848	1.813	**1.846**	-10.570	1.816	1.897	1.836	**1.822**
-10.440	1.860	1.891	1.831	**1.847**	-10.560	1.777	1.765	1.728	**1.826**
-10.430	1.893	1.893	1.918	**1.847**	-10.550	1.898	1.830	1.804	**1.830**
-10.420	1.876	1.844	1.830	**1.847**	-10.540	1.872	1.893	1.843	**1.834**
-10.410	1.838	1.837	1.815	**1.847**	-10.530	1.838	1.785	1.885	**1.838**

-10.400	1.847	1.872	1.844	**1.848**	-10.520	1.755	1.822	1.763	**1.842**
-10.390	1.807	1.824	1.868	**1.848**	-10.510	1.867	1.830	1.788	**1.846**
-10.380	1.878	1.878	1.816	**1.848**	-10.500	1.863	1.824	1.920	**1.850**
-10.370	1.828	1.798	1.833	**1.848**	-10.490	1.860	1.866	1.925	**1.854**
-10.360	1.849	1.858	1.863	**1.849**	-10.480	1.820	1.843	1.883	**1.856**
-10.350	1.889	1.833	1.832	**1.849**	-10.470	1.926	1.985	1.874	**1.858**
-10.340	1.885	1.869	1.911	**1.849**	-10.460	1.868	1.843	1.918	**1.859**
-10.330	1.883	1.955	1.909	**1.849**	-10.450	1.904	1.842	1.858	**1.860**
-10.320	1.795	1.789	1.830	**1.849**	-10.440	1.790	1.809	1.892	**1.861**
-10.310	1.874	1.845	1.883	**1.850**	-10.430	1.911	1.882	1.894	**1.861**
-10.300	1.905	1.837	1.849	**1.850**	-10.420	1.849	1.934	1.870	**1.861**
-10.290	1.857	1.835	1.869	**1.850**	-10.410	1.781	1.806	1.806	**1.861**
-10.280	1.870	1.901	1.854	**1.850**	-10.400	1.798	1.879	1.785	**1.861**
-10.270	1.820	1.847	1.846	**1.850**	-10.390	1.903	1.820	1.817	**1.861**
-10.260	1.851	1.823	1.849	**1.849**	-10.380	1.847	1.856	1.780	**1.861**
-10.250	1.834	1.852	1.859	**1.849**	-10.370	1.898	1.808	1.870	**1.861**
-10.240	1.831	1.863	1.835	**1.849**	-10.360	1.950	1.901	1.877	**1.861**
-10.230	1.845	1.853	1.829	**1.849**	-10.350	1.906	1.874	1.936	**1.861**
-10.220	1.889	1.921	1.846	**1.849**	-10.340	1.830	1.777	1.868	**1.861**
-10.210	1.816	1.817	1.861	**1.848**	-10.330	1.805	1.831	1.851	**1.861**
-10.200	1.842	1.824	1.799	**1.848**	-10.320	1.888	1.928	1.926	**1.861**
-10.190	1.852	1.813	1.817	**1.848**	-10.310	1.826	1.812	1.849	**1.861**
-10.180	1.864	1.899	1.833	**1.848**	-10.300	1.904	1.866	1.838	**1.861**
-10.170	1.854	1.829	1.849	**1.848**	-10.290	1.858	1.894	1.931	**1.861**
-10.160	1.808	1.812	1.832	**1.848**	-10.280	1.897	1.964	1.874	**1.861**
-10.150	1.815	1.846	1.857	**1.848**	-10.270	1.807	1.881	1.892	**1.861**
-10.140	1.874	1.841	1.821	**1.848**	-10.260	1.855	1.886	1.923	**1.862**
-10.130	1.861	1.893	1.815	**1.848**	-10.250	1.774	1.849	1.870	**1.862**
-10.120	1.860	1.894	1.851	**1.848**	-10.240	1.899	1.791	1.862	**1.862**
-10.110	1.858	1.880	1.916	**1.849**	-10.230	1.908	1.827	1.840	**1.863**
-10.100	1.844	1.817	1.847	**1.850**	-10.220	1.831	1.868	1.835	**1.864**
-10.090	1.783	1.811	1.856	**1.850**	-10.210	1.834	1.910	1.809	**1.864**

-10.080	1.844	1.919	1.882	**1.851**	-10.200	1.925	1.924	1.862	**1.865**
-10.070	1.859	1.870	1.865	**1.852**	-10.190	1.893	1.892	1.797	**1.866**
-10.060	1.862	1.851	1.816	**1.852**	-10.180	1.708	1.802	1.793	**1.866**
-10.050	1.884	1.863	1.808	**1.853**	-10.170	1.850	1.878	1.832	**1.866**
-10.040	1.824	1.841	1.816	**1.853**	-10.160	1.839	1.886	1.895	**1.867**
-10.030	1.862	1.858	1.813	**1.854**	-10.150	2.028	1.936	1.935	**1.867**
-10.020	1.816	1.865	1.791	**1.854**	-10.140	1.902	1.893	1.830	**1.867**
-10.010	1.931	1.895	1.935	**1.854**	-10.130	1.906	1.924	1.837	**1.868**
-10.000	1.905	1.866	1.917	**1.855**	-10.120	1.885	1.788	1.832	**1.868**
-9.990	1.857	1.900	1.919	**1.855**	-10.110	1.848	1.901	1.819	**1.869**
-9.980	1.835	1.817	1.847	**1.855**	-10.100	1.891	1.946	1.839	**1.869**
-9.970	1.884	1.849	1.851	**1.855**	-10.090	1.862	1.930	1.842	**1.869**
-9.960	1.885	1.829	1.892	**1.856**	-10.080	1.877	1.881	1.912	**1.869**
-9.950	1.805	1.868	1.809	**1.856**	-10.070	1.862	1.831	1.909	**1.870**
-9.940	1.826	1.856	1.837	**1.856**	-10.060	1.907	1.863	1.916	**1.870**
-9.930	1.793	1.813	1.827	**1.857**	-10.050	1.831	1.907	1.824	**1.870**
-9.920	1.836	1.889	1.890	**1.857**	-10.040	1.944	1.900	1.974	**1.870**
-9.910	1.877	1.818	1.844	**1.857**	-10.030	1.904	1.881	1.884	**1.870**
-9.900	1.882	1.843	1.842	**1.858**	-10.020	1.878	1.832	1.879	**1.871**
-9.890	1.936	1.887	1.872	**1.858**	-10.010	1.813	1.878	1.814	**1.871**
-9.880	1.890	1.885	1.867	**1.859**	-10.000	1.860	1.827	1.931	**1.871**
-9.870	1.784	1.813	1.856	**1.859**	-9.990	1.914	1.804	1.847	**1.871**
-9.860	1.913	1.882	1.879	**1.860**	-9.980	1.874	1.848	1.881	**1.871**
-9.850	1.864	1.861	1.907	**1.861**	-9.970	1.853	1.845	1.881	**1.870**
-9.840	1.882	1.894	1.877	**1.861**	-9.960	1.909	1.919	1.914	**1.870**
-9.830	1.848	1.881	1.887	**1.862**	-9.950	1.835	1.889	1.810	**1.870**
-9.820	1.847	1.875	1.899	**1.863**	-9.940	1.872	1.970	1.915	**1.869**
-9.810	1.831	1.839	1.829	**1.864**	-9.930	1.846	1.886	1.883	**1.869**
-9.800	1.878	1.852	1.848	**1.864**	-9.920	1.903	1.907	1.876	**1.868**
-9.790	1.858	1.894	1.901	**1.864**	-9.910	1.844	1.958	1.895	**1.868**
-9.780	1.833	1.802	1.817	**1.864**	-9.900	1.909	1.929	1.893	**1.867**
-9.770	1.825	1.838	1.814	**1.865**	-9.890	1.925	1.812	1.872	**1.867**

-9.760	1.839	1.874	1.890	**1.865**	-9.880	1.865	1.861	1.932	**1.867**
-9.750	1.867	1.890	1.830	**1.865**	-9.870	1.867	1.804	1.885	**1.867**
-9.740	1.841	1.850	1.833	**1.865**	-9.860	1.904	1.891	1.861	**1.867**
-9.730	1.852	1.860	1.821	**1.866**	-9.850	1.836	1.878	1.810	**1.867**
-9.720	1.939	1.920	1.921	**1.866**	-9.840	1.935	1.839	1.876	**1.867**
-9.710	1.902	1.834	1.882	**1.866**	-9.830	1.891	1.926	1.962	**1.867**
-9.700	1.871	1.886	1.930	**1.866**	-9.820	1.826	1.889	1.784	**1.867**
-9.690	1.844	1.910	1.890	**1.867**	-9.810	1.890	1.859	1.801	**1.867**
-9.680	1.887	1.861	1.815	**1.867**	-9.800	1.821	1.843	1.898	**1.867**
-9.670	1.850	1.831	1.853	**1.867**	-9.790	1.902	1.949	1.929	**1.867**
-9.660	1.895	1.910	1.934	**1.868**	-9.780	1.778	1.872	1.815	**1.867**
-9.650	1.863	1.866	1.918	**1.868**	-9.770	1.890	1.890	1.859	**1.867**
-9.640	1.851	1.851	1.854	**1.869**	-9.760	1.845	1.833	1.911	**1.867**
-9.630	1.870	1.880	1.868	**1.869**	-9.750	1.874	1.926	1.967	**1.867**
-9.620	1.887	1.868	1.928	**1.869**	-9.740	1.952	1.839	1.874	**1.868**
-9.610	1.901	1.861	1.849	**1.870**	-9.730	1.844	1.810	1.902	**1.868**
-9.600	1.844	1.848	1.800	**1.870**	-9.720	1.893	1.869	1.856	**1.868**
-9.590	1.810	1.862	1.843	**1.871**	-9.710	1.882	1.906	1.881	**1.868**
-9.580	1.865	1.883	1.922	**1.871**	-9.700	1.818	1.884	1.816	**1.868**
-9.570	1.927	1.866	1.872	**1.872**	-9.690	1.921	1.889	1.940	**1.868**
-9.560	1.843	1.892	1.832	**1.872**	-9.680	1.969	1.923	1.875	**1.868**
-9.550	1.875	1.909	1.868	**1.873**	-9.670	1.901	1.881	1.847	**1.868**
-9.540	1.871	1.892	1.858	**1.873**	-9.660	1.773	1.792	1.791	**1.868**
-9.530	1.942	1.871	1.877	**1.873**	-9.650	1.847	1.907	1.842	**1.868**
-9.520	1.911	1.895	1.845	**1.874**	-9.640	1.871	1.893	1.828	**1.869**
-9.510	1.838	1.871	1.889	**1.874**	-9.630	1.857	1.940	1.850	**1.869**
-9.500	1.885	1.887	1.946	**1.873**	-9.620	1.865	1.790	1.847	**1.869**
-9.490	1.939	1.923	1.870	**1.873**	-9.610	1.851	1.900	1.945	**1.869**
-9.480	1.831	1.822	1.850	**1.873**	-9.600	1.884	1.842	1.790	**1.869**
-9.470	1.944	1.935	1.889	**1.872**	-9.590	1.865	1.900	1.890	**1.869**
-9.460	1.875	1.869	1.868	**1.872**	-9.580	1.870	1.844	1.881	**1.870**
-9.450	1.947	1.894	1.932	**1.871**	-9.570	1.878	1.896	1.820	**1.870**

-9.440	1.888	1.881	1.941	**1.871**	-9.560	1.851	1.933	1.859	**1.870**
-9.430	1.863	1.858	1.830	**1.871**	-9.550	1.881	1.813	1.872	**1.870**
-9.420	1.849	1.892	1.885	**1.870**	-9.540	1.930	1.936	1.866	**1.871**
-9.410	1.828	1.887	1.852	**1.870**	-9.530	1.869	1.866	1.865	**1.871**
-9.400	1.818	1.817	1.829	**1.869**	-9.520	1.847	1.857	1.811	**1.871**
-9.390	1.949	1.876	1.889	**1.868**	-9.510	1.849	1.873	1.808	**1.871**
-9.380	1.866	1.858	1.844	**1.868**	-9.500	1.875	1.915	1.812	**1.871**
-9.370	1.864	1.903	1.859	**1.867**	-9.490	1.870	1.840	1.906	**1.871**
-9.360	1.954	1.923	1.886	**1.866**	-9.480	1.928	1.930	1.854	**1.871**
-9.350	1.830	1.829	1.875	**1.865**	-9.470	1.859	1.879	1.878	**1.871**
-9.340	1.846	1.877	1.845	**1.864**	-9.460	1.946	1.848	1.916	**1.870**
-9.330	1.888	1.843	1.877	**1.863**	-9.450	1.848	1.770	1.796	**1.870**
-9.320	1.874	1.909	1.904	**1.862**	-9.440	1.860	1.823	1.845	**1.869**
-9.310	1.936	1.886	1.910	**1.862**	-9.430	1.824	1.908	1.910	**1.869**
-9.300	1.885	1.933	1.860	**1.862**	-9.420	1.897	1.859	1.923	**1.869**
-9.290	1.873	1.873	1.873	**1.863**	-9.410	1.826	1.871	1.843	**1.868**
-9.280	1.858	1.837	1.803	**1.864**	-9.400	1.873	1.886	1.849	**1.867**
-9.270	1.899	1.846	1.910	**1.865**	-9.390	1.795	1.898	1.838	**1.867**
-9.260	1.926	1.901	1.899	**1.865**	-9.380	1.871	1.890	1.875	**1.866**
-9.250	1.890	1.885	1.917	**1.865**	-9.370	1.880	1.922	1.843	**1.866**
-9.240	1.918	1.864	1.930	**1.865**	-9.360	1.899	1.842	1.919	**1.865**
-9.230	1.816	1.848	1.825	**1.865**	-9.350	1.900	1.859	1.822	**1.865**
-9.220	1.889	1.854	1.909	**1.865**	-9.340	1.797	1.807	1.836	**1.864**
-9.210	1.852	1.834	1.909	**1.865**	-9.330	1.836	1.830	1.856	**1.863**
-9.200	1.939	1.881	1.879	**1.864**	-9.320	1.942	1.874	1.864	**1.863**
-9.190	1.840	1.828	1.892	**1.864**	-9.310	1.820	1.875	1.829	**1.862**
-9.180	1.879	1.865	1.897	**1.864**	-9.300	1.920	1.873	1.845	**1.862**
-9.170	1.857	1.919	1.879	**1.864**	-9.290	1.780	1.867	1.821	**1.861**
-9.160	1.846	1.804	1.851	**1.864**	-9.280	1.801	1.864	1.830	**1.861**
-9.150	1.826	1.855	1.806	**1.864**	-9.270	1.854	1.772	1.859	**1.860**
-9.140	1.855	1.861	1.857	**1.864**	-9.260	1.840	1.928	1.946	**1.860**
-9.130	1.824	1.837	1.896	**1.864**	-9.250	1.899	1.857	1.848	**1.860**

-9.120	1.875	1.813	1.844	**1.864**
-9.110	1.912	1.921	1.862	**1.864**
-9.100	1.821	1.895	1.838	**1.864**
-9.090	1.875	1.842	1.835	**1.864**
-9.080	1.899	1.846	1.885	**1.864**
-9.070	1.882	1.815	1.858	**1.864**
-9.060	1.817	1.835	1.871	**1.864**
-9.050	1.836	1.822	1.786	**1.864**
-9.040	1.851	1.921	1.918	**1.864**
-9.030	1.855	1.882	1.855	**1.864**
-9.020	1.838	1.875	1.853	**1.864**
-9.010	1.891	1.897	1.852	**1.863**
-9.000	1.837	1.816	1.880	**1.863**
-8.990	1.876	1.883	1.928	**1.863**
-8.980	1.902	1.840	1.884	**1.863**
-8.970	1.888	1.896	1.863	**1.863**
-8.960	1.898	1.870	1.844	**1.863**
-8.950	1.879	1.852	1.874	**1.863**
-8.940	1.842	1.866	1.884	**1.862**
-8.930	1.832	1.865	1.797	**1.862**
-8.920	1.874	1.840	1.862	**1.862**
-8.910	1.856	1.896	1.925	**1.862**
-8.900	1.840	1.865	1.794	**1.862**
-8.890	1.853	1.843	1.878	**1.862**
-8.880	1.848	1.881	1.905	**1.862**
-8.870	1.900	1.870	1.882	**1.862**
-8.860	1.830	1.831	1.801	**1.862**
-8.850	1.876	1.861	1.844	**1.862**
-8.840	1.866	1.833	1.807	**1.863**
-8.830	1.894	1.872	1.851	**1.863**
-8.820	1.864	1.857	1.886	**1.864**
-8.810	1.933	1.894	1.913	**1.865**

-9.240	1.925	1.874	1.878	**1.859**
-9.230	1.817	1.917	1.896	**1.859**
-9.220	1.892	1.928	1.863	**1.858**
-9.210	1.788	1.853	1.869	**1.858**
-9.200	1.892	1.942	1.893	**1.858**
-9.190	1.831	1.898	1.905	**1.858**
-9.180	1.742	1.846	1.828	**1.858**
-9.170	1.858	1.807	1.880	**1.858**
-9.160	1.785	1.889	1.840	**1.858**
-9.150	1.803	1.847	1.844	**1.858**
-9.140	1.893	1.846	1.859	**1.858**
-9.130	1.924	1.845	1.834	**1.858**
-9.120	1.858	1.943	1.958	**1.858**
-9.110	1.846	1.885	1.927	**1.858**
-9.100	1.827	1.885	1.820	**1.858**
-9.090	1.844	1.782	1.842	**1.858**
-9.080	1.829	1.844	1.885	**1.858**
-9.070	1.822	1.779	1.746	**1.858**
-9.060	1.828	1.839	1.856	**1.858**
-9.050	1.906	1.866	1.858	**1.858**
-9.040	1.867	1.872	1.884	**1.858**
-9.030	1.896	1.928	1.858	**1.858**
-9.020	1.891	1.860	1.924	**1.859**
-9.010	1.815	1.783	1.889	**1.859**
-9.000	1.802	1.878	1.855	**1.859**
-8.990	1.857	1.849	1.870	**1.859**
-8.980	1.773	1.783	1.835	**1.859**
-8.970	1.873	1.823	1.878	**1.860**
-8.960	1.877	1.830	1.766	**1.860**
-8.950	1.866	1.803	1.816	**1.860**
-8.940	1.775	1.795	1.858	**1.860**
-8.930	1.865	1.925	1.823	**1.861**

-8.800	1.813	1.881	1.868	**1.865**	-8.920	1.808	1.802	1.843	**1.861**
-8.790	1.859	1.854	1.886	**1.866**	-8.910	1.930	1.835	1.908	**1.861**
-8.780	1.893	1.886	1.892	**1.866**	-8.900	1.859	1.837	1.934	**1.861**
-8.770	1.878	1.851	1.853	**1.867**	-8.890	1.828	1.753	1.841	**1.862**
-8.760	1.866	1.827	1.858	**1.868**	-8.880	1.852	1.860	1.805	**1.862**
-8.750	1.878	1.872	1.855	**1.868**	-8.870	1.877	1.861	1.818	**1.862**
-8.740	1.872	1.849	1.849	**1.869**	-8.860	1.919	1.829	1.885	**1.863**
-8.730	1.867	1.874	1.858	**1.869**	-8.850	1.782	1.804	1.886	**1.863**
-8.720	1.870	1.859	1.816	**1.870**	-8.840	1.849	1.809	1.878	**1.864**
-8.710	1.883	1.894	1.880	**1.871**	-8.830	1.917	1.980	1.887	**1.865**
-8.700	1.889	1.886	1.866	**1.871**	-8.820	1.846	1.834	1.880	**1.865**
-8.690	1.814	1.830	1.862	**1.872**	-8.810	1.855	1.921	1.856	**1.866**
-8.680	1.936	1.907	1.891	**1.873**	-8.800	1.873	1.918	1.839	**1.867**
-8.670	1.878	1.894	1.881	**1.873**	-8.790	1.850	1.858	1.866	**1.867**
-8.660	1.890	1.857	1.885	**1.874**	-8.780	1.844	1.899	1.926	**1.868**
-8.650	1.898	1.865	1.867	**1.875**	-8.770	1.869	1.851	1.888	**1.868**
-8.640	1.909	1.830	1.855	**1.875**	-8.760	1.841	1.874	1.792	**1.869**
-8.630	1.836	1.815	1.848	**1.875**	-8.750	1.893	1.914	1.943	**1.870**
-8.620	1.920	1.883	1.858	**1.875**	-8.740	1.957	1.909	1.958	**1.871**
-8.610	1.921	1.849	1.914	**1.876**	-8.730	1.916	1.904	1.863	**1.871**
-8.600	1.846	1.885	1.906	**1.876**	-8.720	1.876	1.831	1.796	**1.871**
-8.590	1.902	1.856	1.888	**1.876**	-8.710	1.882	1.936	1.850	**1.871**
-8.580	1.872	1.837	1.905	**1.876**	-8.700	1.961	1.865	1.860	**1.871**
-8.570	1.879	1.815	1.868	**1.877**	-8.690	1.945	1.883	1.910	**1.871**
-8.560	1.931	1.908	1.899	**1.877**	-8.680	1.851	1.878	1.911	**1.871**
-8.550	1.898	1.872	1.901	**1.877**	-8.670	1.974	1.942	1.909	**1.871**
-8.540	1.893	1.835	1.860	**1.877**	-8.660	1.879	1.866	1.845	**1.871**
-8.530	1.850	1.896	1.845	**1.877**	-8.650	1.935	1.840	1.831	**1.871**
-8.520	1.879	1.848	1.882	**1.877**	-8.640	1.816	1.872	1.857	**1.870**
-8.510	1.924	1.869	1.925	**1.877**	-8.630	1.862	1.963	1.940	**1.870**
-8.500	1.882	1.818	1.883	**1.877**	-8.620	1.853	1.909	1.890	**1.870**
-8.490	1.841	1.904	1.880	**1.877**	-8.610	1.897	1.947	1.834	**1.870**

-8.480	1.846	1.872	1.885	**1.877**	-8.600	1.854	1.889	1.879	**1.870**
-8.470	1.847	1.834	1.820	**1.877**	-8.590	1.929	1.880	1.921	**1.870**
-8.460	1.871	1.842	1.863	**1.876**	-8.580	1.843	1.931	1.878	**1.870**
-8.450	1.887	1.919	1.924	**1.876**	-8.570	1.882	1.830	1.878	**1.870**
-8.440	1.895	1.886	1.861	**1.875**	-8.560	1.843	1.835	1.862	**1.869**
-8.430	1.907	1.837	1.895	**1.875**	-8.550	1.904	1.817	1.886	**1.869**
-8.420	1.879	1.825	1.867	**1.875**	-8.540	1.937	1.868	1.930	**1.869**
-8.410	1.875	1.869	1.908	**1.875**	-8.530	1.801	1.833	1.832	**1.869**
-8.400	1.855	1.921	1.912	**1.875**	-8.520	1.802	1.765	1.730	**1.868**
-8.390	1.839	1.876	1.873	**1.874**	-8.510	1.919	1.894	1.935	**1.868**
-8.380	1.842	1.870	1.866	**1.874**	-8.500	1.811	1.914	1.847	**1.868**
-8.370	1.874	1.799	1.844	**1.874**	-8.490	1.895	1.873	1.924	**1.868**
-8.360	1.928	1.878	1.892	**1.874**	-8.480	1.909	1.889	1.831	**1.869**
-8.350	1.878	1.904	1.865	**1.874**	-8.470	1.869	1.837	1.861	**1.869**
-8.340	1.840	1.810	1.864	**1.874**	-8.460	1.888	1.922	1.848	**1.869**
-8.330	1.856	1.873	1.898	**1.874**	-8.450	1.820	1.803	1.894	**1.869**
-8.320	1.864	1.873	1.858	**1.873**	-8.440	1.838	1.824	1.804	**1.870**
-8.310	1.833	1.897	1.892	**1.873**	-8.430	1.909	1.846	1.861	**1.870**
-8.300	1.864	1.888	1.853	**1.873**	-8.420	1.826	1.829	1.911	**1.870**
-8.290	1.933	1.901	1.860	**1.873**	-8.410	1.871	1.838	1.807	**1.871**
-8.280	1.842	1.838	1.863	**1.873**	-8.400	1.898	1.905	1.834	**1.871**
-8.270	1.859	1.860	1.846	**1.873**	-8.390	1.848	1.742	1.821	**1.871**
-8.260	1.868	1.825	1.885	**1.873**	-8.380	1.841	1.906	1.873	**1.871**
-8.250	1.933	1.937	1.888	**1.873**	-8.370	1.880	1.892	1.866	**1.871**
-8.240	1.877	1.857	1.880	**1.873**	-8.360	1.805	1.812	1.860	**1.871**
-8.230	1.922	1.870	1.887	**1.873**	-8.350	1.884	1.863	1.862	**1.871**
-8.220	1.871	1.903	1.861	**1.873**	-8.340	1.909	1.861	1.840	**1.871**
-8.210	1.854	1.921	1.912	**1.873**	-8.330	1.820	1.794	1.841	**1.871**
-8.200	1.842	1.868	1.874	**1.873**	-8.320	1.880	1.877	1.818	**1.871**
-8.190	1.885	1.872	1.867	**1.874**	-8.310	1.862	1.884	1.826	**1.871**
-8.180	1.875	1.836	1.868	**1.874**	-8.300	1.834	1.846	1.812	**1.872**
-8.170	1.835	1.892	1.881	**1.874**	-8.290	1.867	1.841	1.915	**1.872**

-8.160	1.910	1.893	1.892	**1.874**
-8.150	1.873	1.851	1.806	**1.875**
-8.140	1.920	1.894	1.896	**1.875**
-8.130	1.918	1.865	1.889	**1.875**
-8.120	1.906	1.885	1.933	**1.876**
-8.110	1.825	1.890	1.835	**1.876**
-8.100	1.861	1.889	1.873	**1.876**
-8.090	1.894	1.898	1.862	**1.876**
-8.080	1.924	1.885	1.882	**1.877**
-8.070	1.845	1.904	1.900	**1.877**
-8.060	1.868	1.849	1.815	**1.877**
-8.050	1.848	1.886	1.872	**1.878**
-8.040	1.808	1.816	1.855	**1.878**
-8.030	1.939	1.860	1.901	**1.878**
-8.020	1.905	1.926	1.911	**1.878**
-8.010	1.903	1.901	1.887	**1.878**
-8.000	1.865	1.933	1.911	**1.879**
-7.990	1.857	1.871	1.842	**1.879**
-7.980	1.863	1.886	1.854	**1.879**
-7.970	1.856	1.837	1.894	**1.879**
-7.960	1.871	1.821	1.898	**1.879**
-7.950	1.907	1.930	1.951	**1.879**
-7.940	1.846	1.823	1.851	**1.878**
-7.930	1.930	1.895	1.852	**1.878**
-7.920	1.833	1.893	1.861	**1.878**
-7.910	1.844	1.882	1.854	**1.877**
-7.900	1.911	1.860	1.930	**1.877**
-7.890	1.892	1.835	1.890	**1.877**
-7.880	1.905	1.850	1.869	**1.876**
-7.870	1.920	1.878	1.911	**1.876**
-7.860	1.856	1.877	1.896	**1.877**
-7.850	1.864	1.840	1.841	**1.877**
-8.280	1.834	1.902	1.867	**1.872**
-8.270	1.886	1.839	1.839	**1.872**
-8.260	1.805	1.900	1.822	**1.872**
-8.250	1.880	1.875	1.887	**1.872**
-8.240	1.862	1.800	1.809	**1.872**
-8.230	1.886	1.872	1.824	**1.872**
-8.220	1.857	1.911	1.890	**1.871**
-8.210	1.866	1.777	1.838	**1.871**
-8.200	1.901	1.871	1.844	**1.870**
-8.190	1.869	1.976	1.932	**1.870**
-8.180	1.834	1.829	1.811	**1.870**
-8.170	1.819	1.752	1.825	**1.869**
-8.160	1.905	1.836	1.951	**1.869**
-8.150	1.789	1.870	1.833	**1.868**
-8.140	1.867	1.855	1.816	**1.868**
-8.130	1.859	1.936	1.830	**1.869**
-8.120	1.859	1.892	1.896	**1.869**
-8.110	1.859	1.906	1.889	**1.870**
-8.100	1.919	1.913	1.904	**1.870**
-8.090	1.964	1.864	1.885	**1.870**
-8.080	1.848	1.889	1.856	**1.871**
-8.070	1.894	1.857	1.854	**1.872**
-8.060	1.945	1.889	1.839	**1.872**
-8.050	1.857	1.869	1.822	**1.873**
-8.040	1.887	1.949	1.864	**1.873**
-8.030	1.841	1.885	1.916	**1.874**
-8.020	1.900	1.910	1.866	**1.874**
-8.010	1.796	1.870	1.885	**1.875**
-8.000	1.882	1.886	1.825	**1.875**
-7.990	1.852	1.855	1.857	**1.876**
-7.980	1.946	1.844	1.854	**1.876**
-7.970	1.964	1.871	1.906	**1.876**

-7.840	1.817	1.871	1.859	**1.878**	-7.960	1.790	1.858	1.836	**1.877**
-7.830	1.860	1.883	1.886	**1.878**	-7.950	1.913	1.897	1.841	**1.877**
-7.820	1.888	1.943	1.921	**1.879**	-7.940	1.875	1.932	1.851	**1.877**
-7.810	1.882	1.845	1.919	**1.879**	-7.930	1.844	1.930	1.890	**1.878**
-7.800	1.890	1.924	1.879	**1.880**	-7.920	1.833	1.802	1.875	**1.878**
-7.790	1.864	1.891	1.869	**1.880**	-7.910	1.917	1.864	1.903	**1.878**
-7.780	1.833	1.884	1.812	**1.881**	-7.900	1.947	1.889	1.914	**1.878**
-7.770	1.941	1.926	1.876	**1.881**	-7.890	1.876	1.912	1.911	**1.878**
-7.760	1.886	1.890	1.858	**1.882**	-7.880	1.849	1.891	1.886	**1.879**
-7.750	1.864	1.915	1.891	**1.882**	-7.870	1.838	1.935	1.833	**1.879**
-7.740	1.912	1.843	1.881	**1.882**	-7.860	1.883	1.905	1.905	**1.879**
-7.730	1.839	1.844	1.898	**1.882**	-7.850	1.932	1.882	1.981	**1.879**
-7.720	1.864	1.851	1.829	**1.882**	-7.840	1.854	1.886	1.952	**1.879**
-7.710	1.899	1.945	1.921	**1.882**	-7.830	1.868	1.866	1.916	**1.879**
-7.700	1.899	1.848	1.850	**1.881**	-7.820	1.849	1.837	1.869	**1.879**
-7.690	1.864	1.804	1.877	**1.881**	-7.810	1.818	1.882	1.905	**1.879**
-7.680	1.838	1.901	1.891	**1.881**	-7.800	1.870	1.908	1.893	**1.879**
-7.670	1.887	1.888	1.876	**1.881**	-7.790	1.908	1.805	1.862	**1.879**
-7.660	1.933	1.936	1.898	**1.881**	-7.780	1.909	1.957	1.902	**1.879**
-7.650	1.842	1.859	1.877	**1.881**	-7.770	1.895	1.846	1.901	**1.879**
-7.640	1.888	1.895	1.849	**1.881**	-7.760	1.838	1.926	1.853	**1.879**
-7.630	1.893	1.856	1.871	**1.881**	-7.750	1.903	1.909	1.842	**1.879**
-7.620	1.924	1.900	1.871	**1.881**	-7.740	1.883	1.891	1.972	**1.878**
-7.610	1.806	1.863	1.854	**1.881**	-7.730	1.839	1.886	1.835	**1.878**
-7.600	1.862	1.900	1.914	**1.880**	-7.720	1.852	1.957	1.889	**1.877**
-7.590	1.925	1.887	1.888	**1.880**	-7.710	1.834	1.831	1.847	**1.877**
-7.580	1.898	1.869	1.921	**1.879**	-7.700	1.809	1.913	1.824	**1.877**
-7.570	1.881	1.824	1.859	**1.879**	-7.690	1.895	1.824	1.896	**1.876**
-7.560	1.904	1.824	1.866	**1.879**	-7.680	1.944	1.855	1.852	**1.876**
-7.550	1.893	1.838	1.848	**1.878**	-7.670	1.866	1.826	1.855	**1.876**
-7.540	1.843	1.883	1.890	**1.878**	-7.660	1.790	1.888	1.883	**1.876**
-7.530	1.808	1.820	1.864	**1.877**	-7.650	1.835	1.870	1.809	**1.876**

-7.520	1.870	1.839	1.878	**1.877**	-7.640	1.951	1.908	1.941	**1.875**
-7.510	1.919	1.886	1.899	**1.876**	-7.630	1.893	1.902	1.842	**1.875**
-7.500	1.872	1.894	1.854	**1.875**	-7.620	1.854	1.749	1.818	**1.875**
-7.490	1.859	1.929	1.879	**1.875**	-7.610	1.818	1.871	1.838	**1.875**
-7.480	1.900	1.913	1.861	**1.874**	-7.600	1.931	1.853	1.843	**1.875**
-7.470	1.920	1.874	1.864	**1.873**	-7.590	1.886	1.847	1.876	**1.875**
-7.460	1.856	1.908	1.919	**1.873**	-7.580	1.844	1.873	1.867	**1.875**
-7.450	1.915	1.870	1.862	**1.872**	-7.570	1.918	1.886	1.857	**1.874**
-7.440	1.915	1.856	1.843	**1.871**	-7.560	1.833	1.884	1.902	**1.874**
-7.430	1.871	1.859	1.895	**1.871**	-7.550	1.811	1.831	1.856	**1.874**
-7.420	1.846	1.855	1.865	**1.870**	-7.540	1.810	1.869	1.885	**1.874**
-7.410	1.856	1.804	1.861	**1.870**	-7.530	1.820	1.880	1.890	**1.873**
-7.400	1.829	1.872	1.877	**1.870**	-7.520	1.892	1.843	1.912	**1.873**
-7.390	1.868	1.859	1.860	**1.870**	-7.510	1.903	1.886	1.844	**1.873**
-7.380	1.841	1.870	1.821	**1.869**	-7.500	1.878	1.832	1.792	**1.873**
-7.370	1.826	1.841	1.900	**1.869**	-7.490	1.878	1.815	1.897	**1.873**
-7.360	1.852	1.869	1.892	**1.869**	-7.480	1.868	1.882	1.823	**1.873**
-7.350	1.895	1.826	1.868	**1.869**	-7.470	1.866	1.860	1.859	**1.873**
-7.340	1.887	1.938	1.892	**1.869**	-7.460	1.903	1.829	1.824	**1.873**
-7.330	1.932	1.943	1.897	**1.869**	-7.450	1.841	1.843	1.798	**1.873**
-7.320	1.903	1.964	1.890	**1.870**	-7.440	1.928	1.856	1.876	**1.873**
-7.310	1.891	1.855	1.853	**1.870**	-7.430	1.790	1.891	1.836	**1.872**
-7.300	1.863	1.818	1.850	**1.870**	-7.420	1.893	1.944	1.911	**1.872**
-7.290	1.842	1.902	1.880	**1.870**	-7.410	1.856	1.866	1.900	**1.872**
-7.280	1.816	1.858	1.867	**1.870**	-7.400	1.925	1.913	1.922	**1.872**
-7.270	1.848	1.903	1.903	**1.871**	-7.390	1.851	1.933	1.862	**1.872**
-7.260	1.887	1.903	1.890	**1.871**	-7.380	1.934	1.880	1.903	**1.871**
-7.250	1.875	1.896	1.881	**1.871**	-7.370	1.851	1.829	1.907	**1.871**
-7.240	1.840	1.861	1.853	**1.871**	-7.360	1.967	1.917	1.918	**1.871**
-7.230	1.883	1.832	1.868	**1.871**	-7.350	1.891	1.855	1.880	**1.871**
-7.220	1.896	1.880	1.826	**1.871**	-7.340	1.775	1.887	1.838	**1.871**
-7.210	1.881	1.895	1.903	**1.871**	-7.330	1.892	1.900	1.868	**1.871**

-7.200	1.841	1.847	1.856	**1.871**	-7.320	1.917	1.867	1.927	**1.871**
-7.190	1.910	1.852	1.849	**1.872**	-7.310	1.850	1.761	1.851	**1.871**
-7.180	1.844	1.866	1.838	**1.872**	-7.300	1.835	1.912	1.920	**1.871**
-7.170	1.876	1.893	1.857	**1.872**	-7.290	1.817	1.820	1.913	**1.870**
-7.160	1.849	1.806	1.885	**1.871**	-7.280	1.822	1.861	1.842	**1.870**
-7.150	1.916	1.886	1.884	**1.871**	-7.270	1.918	1.818	1.875	**1.870**
-7.140	1.870	1.814	1.851	**1.871**	-7.260	1.881	1.871	1.803	**1.870**
-7.130	1.826	1.815	1.882	**1.871**	-7.250	1.799	1.861	1.897	**1.870**
-7.120	1.870	1.870	1.896	**1.871**	-7.240	1.923	1.892	1.957	**1.870**
-7.110	1.893	1.847	1.899	**1.871**	-7.230	1.888	1.851	1.957	**1.870**
-7.100	1.816	1.851	1.846	**1.871**	-7.220	1.887	1.988	1.968	**1.870**
-7.090	1.841	1.837	1.849	**1.871**	-7.210	1.747	1.841	1.741	**1.870**
-7.080	1.914	1.902	1.894	**1.871**	-7.200	1.808	1.911	1.854	**1.869**
-7.070	1.875	1.853	1.851	**1.871**	-7.190	1.916	1.820	1.898	**1.869**
-7.060	1.834	1.839	1.783	**1.871**	-7.180	1.855	1.852	1.929	**1.869**
-7.050	1.896	1.840	1.876	**1.870**	-7.170	1.723	1.802	1.835	**1.869**
-7.040	1.842	1.874	1.900	**1.870**	-7.160	1.866	1.853	1.809	**1.869**
-7.030	1.865	1.879	1.838	**1.870**	-7.150	1.853	1.844	1.896	**1.869**
-7.020	1.861	1.859	1.856	**1.870**	-7.140	1.846	1.853	1.827	**1.869**
-7.010	1.874	1.815	1.836	**1.870**	-7.130	1.785	1.808	1.843	**1.869**
-7.000	1.819	1.865	1.867	**1.870**	-7.120	1.801	1.860	1.868	**1.869**
-6.990	1.887	1.859	1.856	**1.870**	-7.110	1.946	1.848	1.937	**1.868**
-6.980	1.893	1.923	1.909	**1.870**	-7.100	1.872	1.891	1.838	**1.868**
-6.970	1.889	1.868	1.840	**1.870**	-7.090	1.876	1.884	1.847	**1.868**
-6.960	1.866	1.869	1.872	**1.870**	-7.080	1.836	1.894	1.831	**1.868**
-6.950	1.904	1.846	1.914	**1.870**	-7.070	1.875	1.902	1.850	**1.869**
-6.940	1.905	1.841	1.869	**1.870**	-7.060	1.905	1.854	1.854	**1.869**
-6.930	1.845	1.824	1.866	**1.871**	-7.050	1.928	1.895	1.833	**1.869**
-6.920	1.803	1.854	1.870	**1.871**	-7.040	1.893	1.869	1.845	**1.869**
-6.910	1.865	1.885	1.840	**1.871**	-7.030	1.821	1.845	1.893	**1.869**
-6.900	1.925	1.880	1.935	**1.871**	-7.020	1.800	1.887	1.888	**1.869**
-6.890	1.885	1.912	1.936	**1.871**	-7.010	1.890	1.904	1.885	**1.869**

-6.880	1.888	1.883	1.880	**1.871**	-7.000	1.834	1.869	1.796	**1.869**
-6.870	1.887	1.893	1.879	**1.871**	-6.990	1.826	1.886	1.825	**1.869**
-6.860	1.900	1.876	1.890	**1.871**	-6.980	1.858	1.876	1.889	**1.869**
-6.850	1.839	1.822	1.853	**1.871**	-6.970	1.859	1.795	1.902	**1.870**
-6.840	1.831	1.888	1.856	**1.870**	-6.960	1.894	1.875	1.894	**1.870**
-6.830	1.878	1.888	1.894	**1.870**	-6.950	1.855	1.885	1.914	**1.870**
-6.820	1.875	1.863	1.849	**1.870**	-6.940	1.782	1.812	1.882	**1.870**
-6.810	1.804	1.867	1.851	**1.870**	-6.930	1.927	1.848	1.869	**1.870**
-6.800	1.887	1.895	1.849	**1.870**	-6.920	1.818	1.826	1.893	**1.870**
-6.790	1.824	1.888	1.860	**1.869**	-6.910	1.908	1.856	1.871	**1.870**
-6.780	1.924	1.906	1.865	**1.869**	-6.900	1.910	1.920	1.875	**1.870**
-6.770	1.800	1.878	1.841	**1.868**	-6.890	1.831	1.896	1.872	**1.870**
-6.760	1.851	1.845	1.832	**1.868**	-6.880	1.928	1.879	1.987	**1.870**
-6.750	1.815	1.866	1.858	**1.867**	-6.870	1.929	1.852	1.858	**1.871**
-6.740	1.874	1.901	1.919	**1.867**	-6.860	1.838	1.869	1.940	**1.871**
-6.730	1.847	1.854	1.879	**1.867**	-6.850	1.826	1.921	1.921	**1.871**
-6.720	1.865	1.843	1.893	**1.866**	-6.840	1.923	1.842	1.867	**1.872**
-6.710	1.871	1.898	1.858	**1.866**	-6.830	1.876	1.894	1.817	**1.872**
-6.700	1.895	1.894	1.855	**1.865**	-6.820	1.893	1.790	1.795	**1.873**
-6.690	1.813	1.883	1.862	**1.865**	-6.810	1.884	1.840	1.927	**1.873**
-6.680	1.913	1.855	1.881	**1.865**	-6.800	1.828	1.932	1.862	**1.873**
-6.670	1.862	1.841	1.863	**1.864**	-6.790	1.915	1.938	1.957	**1.874**
-6.660	1.882	1.853	1.843	**1.864**	-6.780	1.994	1.941	1.895	**1.874**
-6.650	1.867	1.816	1.802	**1.863**	-6.770	1.924	1.949	1.874	**1.875**
-6.640	1.867	1.885	1.839	**1.863**	-6.760	1.852	1.810	1.850	**1.875**
-6.630	1.880	1.809	1.863	**1.863**	-6.750	1.786	1.880	1.828	**1.876**
-6.620	1.817	1.825	1.826	**1.863**	-6.740	1.929	1.819	1.863	**1.876**
-6.610	1.862	1.876	1.826	**1.863**	-6.730	1.929	1.842	1.842	**1.877**
-6.600	1.846	1.834	1.852	**1.863**	-6.720	1.873	1.854	1.914	**1.877**
-6.590	1.825	1.883	1.849	**1.863**	-6.710	1.916	1.901	1.888	**1.877**
-6.580	1.897	1.852	1.842	**1.863**	-6.700	1.895	1.903	1.920	**1.877**
-6.570	1.909	1.874	1.905	**1.862**	-6.690	1.914	1.962	1.878	**1.877**

-6.560	1.908	1.864	1.875	**1.862**	-6.680	1.831	1.855	1.865	**1.877**
-6.550	1.898	1.909	1.868	**1.863**	-6.670	1.880	1.874	1.846	**1.876**
-6.540	1.840	1.877	1.900	**1.863**	-6.660	1.814	1.844	1.922	**1.876**
-6.530	1.871	1.839	1.816	**1.863**	-6.650	1.891	1.816	1.831	**1.876**
-6.520	1.878	1.815	1.827	**1.863**	-6.640	1.999	1.920	1.895	**1.876**
-6.510	1.872	1.866	1.919	**1.863**	-6.630	1.855	1.895	1.847	**1.876**
-6.500	1.865	1.911	1.897	**1.863**	-6.620	1.878	1.848	1.875	**1.876**
-6.490	1.782	1.858	1.838	**1.864**	-6.610	1.876	1.828	1.908	**1.876**
-6.480	1.846	1.827	1.871	**1.864**	-6.600	1.824	1.858	1.901	**1.876**
-6.470	1.844	1.846	1.842	**1.864**	-6.590	1.849	1.856	1.828	**1.876**
-6.460	1.823	1.794	1.780	**1.864**	-6.580	1.935	1.903	1.867	**1.876**
-6.450	1.917	1.889	1.903	**1.864**	-6.570	1.755	1.860	1.816	**1.875**
-6.440	1.822	1.851	1.861	**1.864**	-6.560	1.923	1.858	1.845	**1.875**
-6.430	1.822	1.882	1.865	**1.864**	-6.550	1.882	1.812	1.823	**1.875**
-6.420	1.892	1.870	1.835	**1.864**	-6.540	1.834	1.848	1.906	**1.875**
-6.410	1.827	1.791	1.818	**1.864**	-6.530	1.854	1.895	1.835	**1.875**
-6.400	1.876	1.836	1.856	**1.864**	-6.520	1.843	1.871	1.896	**1.875**
-6.390	1.849	1.902	1.856	**1.864**	-6.510	1.848	1.794	1.881	**1.874**
-6.380	1.857	1.874	1.878	**1.864**	-6.500	1.866	1.888	1.833	**1.874**
-6.370	1.902	1.835	1.874	**1.864**	-6.490	1.887	1.870	1.894	**1.874**
-6.360	1.896	1.837	1.856	**1.864**	-6.480	1.924	1.891	1.864	**1.874**
-6.350	1.847	1.879	1.878	**1.865**	-6.470	1.882	1.798	1.826	**1.873**
-6.340	1.845	1.827	1.897	**1.865**	-6.460	1.883	1.856	1.891	**1.873**
-6.330	1.862	1.866	1.932	**1.865**	-6.450	1.870	1.776	1.810	**1.873**
-6.320	1.852	1.827	1.842	**1.865**	-6.440	1.882	1.891	1.975	**1.873**
-6.310	1.917	1.880	1.940	**1.866**	-6.430	1.868	1.917	1.878	**1.872**
-6.300	1.912	1.885	1.848	**1.866**	-6.420	1.913	1.850	1.878	**1.872**
-6.290	1.841	1.883	1.845	**1.866**	-6.410	1.857	1.813	1.849	**1.872**
-6.280	1.889	1.918	1.855	**1.866**	-6.400	1.822	1.872	1.835	**1.871**
-6.270	1.885	1.847	1.844	**1.866**	-6.390	1.820	1.920	1.825	**1.871**
-6.260	1.894	1.830	1.859	**1.866**	-6.380	1.878	1.906	1.904	**1.870**
-6.250	1.842	1.862	1.903	**1.867**	-6.370	1.829	1.868	1.878	**1.870**

-6.240	1.862	1.883	1.819	**1.867**	-6.360	1.856	1.901	1.941	**1.870**
-6.230	1.919	1.880	1.916	**1.867**	-6.350	1.858	1.807	1.882	**1.870**
-6.220	1.851	1.906	1.869	**1.867**	-6.340	1.941	1.842	1.885	**1.869**
-6.210	1.918	1.886	1.893	**1.867**	-6.330	1.865	1.891	1.926	**1.869**
-6.200	1.924	1.860	1.885	**1.867**	-6.320	1.856	1.815	1.833	**1.869**
-6.190	1.830	1.873	1.817	**1.867**	-6.310	1.848	1.831	1.855	**1.869**
-6.180	1.845	1.824	1.879	**1.867**	-6.300	1.864	1.917	1.937	**1.869**
-6.170	1.874	1.880	1.865	**1.868**	-6.290	1.922	1.963	1.890	**1.869**
-6.160	1.828	1.864	1.800	**1.868**	-6.280	1.851	1.854	1.950	**1.868**
-6.150	1.841	1.883	1.805	**1.868**	-6.270	1.834	1.832	1.875	**1.868**
-6.140	1.873	1.861	1.827	**1.869**	-6.260	1.854	1.821	1.892	**1.868**
-6.130	1.867	1.875	1.854	**1.869**	-6.250	1.892	1.930	1.948	**1.868**
-6.120	1.860	1.842	1.894	**1.870**	-6.240	1.814	1.781	1.840	**1.867**
-6.110	1.910	1.956	1.908	**1.870**	-6.230	1.915	1.925	1.922	**1.867**
-6.100	1.867	1.853	1.923	**1.870**	-6.220	1.877	1.838	1.950	**1.866**
-6.090	1.842	1.836	1.874	**1.871**	-6.210	1.849	1.927	1.866	**1.866**
-6.080	1.911	1.856	1.835	**1.871**	-6.200	1.910	1.880	1.929	**1.865**
-6.070	1.915	1.875	1.932	**1.871**	-6.190	1.788	1.842	1.814	**1.864**
-6.060	1.892	1.888	1.873	**1.871**	-6.180	1.839	1.895	1.862	**1.864**
-6.050	1.862	1.866	1.803	**1.871**	-6.170	1.916	1.929	1.893	**1.863**
-6.040	1.910	1.870	1.916	**1.872**	-6.160	1.850	1.890	1.956	**1.863**
-6.030	1.847	1.875	1.855	**1.872**	-6.150	1.906	1.880	1.807	**1.863**
-6.020	1.903	1.877	1.853	**1.872**	-6.140	1.821	1.812	1.856	**1.862**
-6.010	1.834	1.802	1.875	**1.872**	-6.130	1.875	1.891	1.909	**1.862**
-6.000	1.917	1.963	1.893	**1.873**	-6.120	1.877	1.937	1.880	**1.862**
-5.990	1.841	1.874	1.894	**1.873**	-6.110	1.922	1.851	1.962	**1.862**
-5.980	1.830	1.879	1.850	**1.873**	-6.100	1.781	1.832	1.844	**1.863**
-5.970	1.859	1.876	1.864	**1.873**	-6.090	1.827	1.836	1.818	**1.863**
-5.960	1.840	1.826	1.848	**1.873**	-6.080	1.896	1.886	1.955	**1.863**
-5.950	1.837	1.898	1.857	**1.873**	-6.070	1.877	1.829	1.940	**1.863**
-5.940	1.901	1.900	1.860	**1.873**	-6.060	1.819	1.836	1.833	**1.864**
-5.930	1.828	1.902	1.839	**1.874**	-6.050	1.824	1.885	1.870	**1.864**

-5.920	1.904	1.878	1.842	**1.874**	-6.040	1.864	1.836	1.878	**1.864**
-5.910	1.904	1.914	1.929	**1.874**	-6.030	1.891	1.820	1.890	**1.864**
-5.900	1.802	1.842	1.878	**1.874**	-6.020	1.862	1.830	1.860	**1.864**
-5.890	1.855	1.880	1.849	**1.875**	-6.010	1.918	1.912	1.830	**1.864**
-5.880	1.903	1.848	1.863	**1.875**	-6.000	1.839	1.913	1.852	**1.864**
-5.870	1.897	1.853	1.837	**1.875**	-5.990	1.881	1.911	1.891	**1.864**
-5.860	1.860	1.855	1.865	**1.875**	-5.980	1.878	1.914	1.823	**1.864**
-5.850	1.885	1.845	1.904	**1.875**	-5.970	1.865	1.802	1.771	**1.864**
-5.840	1.903	1.860	1.888	**1.875**	-5.960	1.953	1.903	1.892	**1.863**
-5.830	1.868	1.896	1.861	**1.875**	-5.950	1.865	1.861	1.863	**1.863**
-5.820	1.890	1.884	1.895	**1.875**	-5.940	1.853	1.874	1.786	**1.863**
-5.810	1.847	1.816	1.882	**1.875**	-5.930	1.856	1.874	1.773	**1.863**
-5.800	1.853	1.837	1.855	**1.875**	-5.920	1.875	1.839	1.903	**1.863**
-5.790	1.883	1.872	1.925	**1.875**	-5.910	1.836	1.863	1.875	**1.863**
-5.780	1.847	1.824	1.864	**1.875**	-5.900	1.766	1.855	1.845	**1.864**
-5.770	1.859	1.818	1.875	**1.875**	-5.890	1.870	1.834	1.855	**1.864**
-5.760	1.886	1.894	1.858	**1.875**	-5.880	1.836	1.842	1.772	**1.864**
-5.750	1.912	1.878	1.918	**1.875**	-5.870	1.871	1.887	1.815	**1.864**
-5.740	1.918	1.880	1.944	**1.875**	-5.860	1.800	1.728	1.805	**1.864**
-5.730	1.893	1.849	1.892	**1.875**	-5.850	1.844	1.856	1.846	**1.865**
-5.720	1.856	1.880	1.862	**1.875**	-5.840	1.933	1.859	1.870	**1.865**
-5.710	1.882	1.931	1.932	**1.875**	-5.830	1.820	1.814	1.835	**1.865**
-5.700	1.913	1.888	1.840	**1.875**	-5.820	1.899	1.924	1.933	**1.865**
-5.690	1.871	1.867	1.891	**1.876**	-5.810	1.856	1.814	1.812	**1.865**
-5.680	1.867	1.887	1.923	**1.876**	-5.800	1.909	1.928	1.827	**1.865**
-5.670	1.833	1.868	1.900	**1.877**	-5.790	1.902	1.897	1.874	**1.865**
-5.660	1.898	1.892	1.831	**1.877**	-5.780	1.871	1.804	1.847	**1.865**
-5.650	1.879	1.871	1.930	**1.878**	-5.770	1.921	1.848	1.840	**1.865**
-5.640	1.854	1.830	1.875	**1.877**	-5.760	1.784	1.836	1.883	**1.866**
-5.630	1.847	1.887	1.909	**1.876**	-5.750	1.846	1.830	1.831	**1.865**
-5.620	1.828	1.899	1.862	**1.876**	-5.740	1.921	1.870	1.906	**1.865**
-5.610	1.906	1.884	1.887	**1.875**	-5.730	1.854	1.957	1.912	**1.865**

-5.600	1.944	1.889	1.892	**1.874**	-5.720	1.832	1.883	1.780	**1.865**
-5.590	1.898	1.840	1.897	**1.874**	-5.710	1.914	1.833	1.871	**1.865**
-5.580	1.909	1.840	1.852	**1.873**	-5.700	1.908	1.933	1.890	**1.865**
-5.570	1.898	1.870	1.890	**1.873**	-5.690	1.836	1.888	1.862	**1.866**
-5.560	1.910	1.912	1.890	**1.872**	-5.680	1.872	1.811	1.775	**1.866**
-5.550	1.881	1.929	1.950	**1.872**	-5.670	1.823	1.855	1.856	**1.866**
-5.540	1.824	1.864	1.856	**1.871**	-5.660	1.850	1.913	1.897	**1.866**
-5.530	1.857	1.888	1.877	**1.871**	-5.650	1.850	1.786	1.851	**1.866**
-5.520	1.830	1.842	1.893	**1.870**	-5.640	1.895	1.883	1.801	**1.867**
-5.510	1.870	1.889	1.826	**1.870**	-5.630	1.966	1.885	1.884	**1.867**
-5.500	1.861	1.853	1.884	**1.870**	-5.620	1.852	1.789	1.888	**1.867**
-5.490	1.883	1.899	1.846	**1.870**	-5.610	1.924	1.835	1.935	**1.867**
-5.480	1.864	1.849	1.885	**1.870**	-5.600	1.815	1.892	1.881	**1.867**
-5.470	1.807	1.810	1.809	**1.871**	-5.590	1.870	1.827	1.892	**1.867**
-5.460	1.876	1.843	1.824	**1.871**	-5.580	1.869	1.807	1.914	**1.867**
-5.450	1.883	1.833	1.839	**1.871**	-5.570	1.906	1.873	1.931	**1.867**
-5.440	1.871	1.876	1.842	**1.871**	-5.560	1.815	1.833	1.878	**1.868**
-5.430	1.844	1.873	1.841	**1.872**	-5.550	1.868	1.896	1.861	**1.868**
-5.420	1.904	1.880	1.922	**1.872**	-5.540	1.931	1.983	1.905	**1.868**
-5.410	1.917	1.914	1.906	**1.873**	-5.530	1.962	1.909	1.987	**1.868**
-5.400	1.812	1.856	1.854	**1.873**	-5.520	1.804	1.879	1.917	**1.869**
-5.390	1.911	1.868	1.841	**1.874**	-5.510	1.847	1.894	1.862	**1.869**
-5.380	1.905	1.834	1.859	**1.874**	-5.500	1.877	1.811	1.853	**1.869**
-5.370	1.850	1.862	1.853	**1.875**	-5.490	1.885	1.827	1.891	**1.869**
-5.360	1.926	1.871	1.872	**1.875**	-5.480	1.863	1.899	1.908	**1.870**
-5.350	1.861	1.876	1.922	**1.876**	-5.470	1.822	1.872	1.878	**1.870**
-5.340	1.844	1.880	1.861	**1.876**	-5.460	1.878	1.852	1.885	**1.870**
-5.330	1.857	1.920	1.921	**1.877**	-5.450	1.904	1.873	1.884	**1.870**
-5.320	1.906	1.851	1.912	**1.877**	-5.440	1.840	1.866	1.758	**1.871**
-5.310	1.880	1.875	1.861	**1.877**	-5.430	1.918	1.958	1.935	**1.871**
-5.300	1.823	1.857	1.858	**1.878**	-5.420	1.845	1.936	1.860	**1.871**
-5.290	1.884	1.916	1.879	**1.878**	-5.410	1.931	1.863	1.906	**1.871**

-5.280	1.889	1.912	1.933	**1.878**	-5.400	1.835	1.881	1.825	**1.872**
-5.270	1.877	1.872	1.918	**1.879**	-5.390	1.910	1.892	1.927	**1.872**
-5.260	1.880	1.897	1.914	**1.879**	-5.380	1.823	1.829	1.811	**1.872**
-5.250	1.858	1.869	1.901	**1.879**	-5.370	1.860	1.843	1.938	**1.872**
-5.240	1.835	1.841	1.896	**1.879**	-5.360	1.834	1.895	1.861	**1.873**
-5.230	1.892	1.914	1.891	**1.879**	-5.350	1.911	1.853	1.907	**1.873**
-5.220	1.897	1.882	1.846	**1.879**	-5.340	1.824	1.828	1.848	**1.873**
-5.210	1.906	1.878	1.878	**1.879**	-5.330	1.867	1.935	1.889	**1.874**
-5.200	1.854	1.803	1.866	**1.879**	-5.320	1.885	1.895	1.801	**1.874**
-5.190	1.886	1.946	1.890	**1.878**	-5.310	1.868	1.910	1.849	**1.875**
-5.180	1.834	1.858	1.786	**1.877**	-5.300	1.835	1.910	1.846	**1.875**
-5.170	1.874	1.918	1.926	**1.877**	-5.290	1.869	1.856	1.930	**1.875**
-5.160	1.846	1.858	1.920	**1.876**	-5.280	1.902	1.857	1.961	**1.876**
-5.150	1.908	1.888	1.915	**1.875**	-5.270	1.919	1.827	1.845	**1.876**
-5.140	1.891	1.885	1.931	**1.874**	-5.260	1.935	1.829	1.864	**1.876**
-5.130	1.884	1.864	1.885	**1.874**	-5.250	1.885	1.889	1.789	**1.877**
-5.120	1.923	1.894	1.889	**1.873**	-5.240	1.786	1.878	1.874	**1.877**
-5.110	1.836	1.854	1.806	**1.872**	-5.230	1.852	1.896	1.895	**1.877**
-5.100	1.905	1.926	1.908	**1.871**	-5.220	1.929	1.877	1.906	**1.876**
-5.090	1.809	1.838	1.883	**1.871**	-5.210	1.909	1.893	1.919	**1.876**
-5.080	1.891	1.898	1.862	**1.871**	-5.200	1.820	1.851	1.918	**1.876**
-5.070	1.887	1.864	1.889	**1.872**	-5.190	1.893	1.893	1.913	**1.876**
-5.060	1.867	1.851	1.868	**1.872**	-5.180	1.854	1.926	1.823	**1.876**
-5.050	1.888	1.942	1.886	**1.872**	-5.170	1.897	1.868	1.894	**1.876**
-5.040	1.824	1.877	1.887	**1.872**	-5.160	1.848	1.850	1.876	**1.876**
-5.030	1.900	1.845	1.839	**1.872**	-5.150	1.819	1.836	1.834	**1.875**
-5.020	1.904	1.829	1.867	**1.873**	-5.140	1.878	1.890	1.868	**1.875**
-5.010	1.884	1.910	1.888	**1.873**	-5.130	1.953	1.876	1.908	**1.875**
-5.000	1.847	1.887	1.904	**1.872**	-5.120	1.893	1.813	1.895	**1.875**
-4.990	1.879	1.935	1.890	**1.872**	-5.110	1.893	1.896	1.908	**1.874**
-4.980	1.869	1.808	1.853	**1.872**	-5.100	1.885	1.989	1.934	**1.874**
-4.970	1.893	1.898	1.892	**1.872**	-5.090	1.825	1.906	1.908	**1.873**

-4.960	1.830	1.843	1.840	**1.872**	-5.080	1.888	1.925	1.913	**1.873**
-4.950	1.854	1.856	1.901	**1.872**	-5.070	1.809	1.894	1.814	**1.873**
-4.940	1.864	1.877	1.882	**1.872**	-5.060	1.831	1.884	1.913	**1.872**
-4.930	1.801	1.791	1.823	**1.872**	-5.050	1.879	1.851	1.843	**1.871**
-4.920	1.877	1.877	1.907	**1.873**	-5.040	1.893	1.880	1.878	**1.871**
-4.910	1.876	1.881	1.824	**1.873**	-5.030	1.833	1.865	1.922	**1.870**
-4.900	1.874	1.917	1.887	**1.873**	-5.020	1.838	1.893	1.877	**1.870**
-4.890	1.848	1.908	1.868	**1.873**	-5.010	1.864	1.899	1.807	**1.869**
-4.880	1.935	1.875	1.921	**1.873**	-5.000	1.867	1.848	1.806	**1.869**
-4.870	1.836	1.876	1.890	**1.873**	-4.990	1.924	1.908	1.884	**1.869**
-4.860	1.825	1.825	1.810	**1.873**	-4.980	1.936	1.994	1.941	**1.868**
-4.850	1.899	1.871	1.881	**1.872**	-4.970	1.856	1.791	1.832	**1.868**
-4.840	1.824	1.863	1.835	**1.872**	-4.960	1.946	1.840	1.902	**1.868**
-4.830	1.877	1.854	1.842	**1.871**	-4.950	1.905	1.924	1.866	**1.868**
-4.820	1.854	1.911	1.896	**1.871**	-4.940	1.988	1.873	1.919	**1.868**
-4.810	1.850	1.887	1.854	**1.870**	-4.930	1.869	1.779	1.825	**1.869**
-4.800	1.861	1.847	1.860	**1.870**	-4.920	1.847	1.900	1.815	**1.869**
-4.790	1.810	1.836	1.831	**1.869**	-4.910	1.871	1.945	1.843	**1.869**
-4.780	1.792	1.836	1.835	**1.868**	-4.900	1.856	1.868	1.956	**1.869**
-4.770	1.915	1.889	1.919	**1.868**	-4.890	1.799	1.876	1.871	**1.870**
-4.760	1.825	1.862	1.821	**1.867**	-4.880	1.882	1.819	1.900	**1.870**
-4.750	1.911	1.850	1.893	**1.867**	-4.870	1.844	1.830	1.772	**1.870**
-4.740	1.894	1.847	1.897	**1.866**	-4.860	1.862	1.893	1.832	**1.871**
-4.730	1.861	1.857	1.819	**1.866**	-4.850	1.870	1.855	1.843	**1.871**
-4.720	1.846	1.852	1.858	**1.865**	-4.840	1.957	1.847	1.927	**1.872**
-4.710	1.819	1.850	1.858	**1.864**	-4.830	1.837	1.843	1.828	**1.872**
-4.700	1.944	1.893	1.942	**1.864**	-4.820	1.930	1.933	1.838	**1.872**
-4.690	1.807	1.856	1.818	**1.863**	-4.810	1.908	1.897	1.830	**1.873**
-4.680	1.858	1.833	1.857	**1.863**	-4.800	1.880	1.889	1.883	**1.873**
-4.670	1.845	1.867	1.884	**1.862**	-4.790	1.859	1.912	1.910	**1.874**
-4.660	1.902	1.848	1.907	**1.862**	-4.780	1.843	1.860	1.905	**1.874**
-4.650	1.818	1.849	1.879	**1.862**	-4.770	1.856	1.910	1.896	**1.874**

-4.640	1.905	1.885	1.875	**1.861**	-4.760	1.861	1.867	1.913	**1.875**
-4.630	1.890	1.924	1.850	**1.861**	-4.750	1.852	1.909	1.849	**1.875**
-4.620	1.856	1.839	1.860	**1.860**	-4.740	1.889	1.867	1.838	**1.875**
-4.610	1.834	1.829	1.834	**1.860**	-4.730	1.858	1.902	1.884	**1.875**
-4.600	1.842	1.823	1.849	**1.859**	-4.720	1.896	1.853	1.874	**1.875**
-4.590	1.805	1.848	1.836	**1.859**	-4.710	1.894	1.878	1.876	**1.875**
-4.580	1.804	1.859	1.858	**1.859**	-4.700	1.905	1.855	1.890	**1.876**
-4.570	1.867	1.890	1.814	**1.858**	-4.690	1.845	1.872	1.919	**1.876**
-4.560	1.820	1.897	1.859	**1.858**	-4.680	1.886	1.906	1.870	**1.876**
-4.550	1.830	1.814	1.884	**1.858**	-4.670	1.909	1.868	1.902	**1.876**
-4.540	1.848	1.860	1.821	**1.857**	-4.660	1.853	1.898	1.931	**1.876**
-4.530	1.926	1.899	1.863	**1.858**	-4.650	1.904	1.946	1.984	**1.876**
-4.520	1.871	1.885	1.906	**1.858**	-4.640	1.833	1.934	1.881	**1.876**
-4.510	1.799	1.798	1.832	**1.858**	-4.630	1.954	1.909	1.894	**1.876**
-4.500	1.862	1.864	1.826	**1.859**	-4.620	1.847	1.929	1.885	**1.876**
-4.490	1.861	1.874	1.915	**1.859**	-4.610	1.868	1.917	1.977	**1.876**
-4.480	1.846	1.858	1.908	**1.859**	-4.600	1.850	1.829	1.887	**1.875**
-4.470	1.776	1.848	1.827	**1.859**	-4.590	1.861	1.939	1.880	**1.875**
-4.460	1.877	1.813	1.840	**1.859**	-4.580	1.861	1.850	1.883	**1.875**
-4.450	1.828	1.884	1.828	**1.860**	-4.570	1.808	1.877	1.876	**1.875**
-4.440	1.840	1.869	1.875	**1.860**	-4.560	1.839	1.903	1.928	**1.874**
-4.430	1.813	1.854	1.837	**1.861**	-4.550	1.898	1.908	1.858	**1.874**
-4.420	1.899	1.850	1.850	**1.861**	-4.540	1.866	1.879	1.803	**1.873**
-4.410	1.839	1.880	1.900	**1.862**	-4.530	1.898	1.807	1.885	**1.873**
-4.400	1.887	1.825	1.818	**1.863**	-4.520	1.843	1.929	1.925	**1.872**
-4.390	1.840	1.878	1.859	**1.863**	-4.510	1.925	1.913	1.856	**1.872**
-4.380	1.888	1.867	1.868	**1.864**	-4.500	1.806	1.855	1.892	**1.871**
-4.370	1.879	1.896	1.830	**1.865**	-4.490	1.784	1.897	1.857	**1.871**
-4.360	1.806	1.867	1.812	**1.865**	-4.480	1.900	1.794	1.849	**1.870**
-4.350	1.846	1.904	1.845	**1.866**	-4.470	1.796	1.828	1.763	**1.869**
-4.340	1.872	1.921	1.864	**1.867**	-4.460	1.873	1.843	1.953	**1.869**
-4.330	1.892	1.885	1.864	**1.867**	-4.450	1.927	1.852	1.913	**1.868**

-4.320	1.890	1.886	1.855	**1.868**	-4.440	1.841	1.874	1.774	**1.868**
-4.310	1.864	1.813	1.887	**1.869**	-4.430	1.918	1.863	1.939	**1.867**
-4.300	1.870	1.865	1.913	**1.869**	-4.420	1.893	1.856	1.828	**1.867**
-4.290	1.877	1.889	1.865	**1.870**	-4.410	1.891	1.914	1.839	**1.867**
-4.280	1.940	1.877	1.873	**1.870**	-4.400	1.839	1.867	1.872	**1.867**
-4.270	1.880	1.875	1.897	**1.871**	-4.390	1.900	1.892	1.915	**1.867**
-4.260	1.850	1.862	1.810	**1.871**	-4.380	1.926	1.898	1.831	**1.867**
-4.250	1.903	1.911	1.950	**1.872**	-4.370	1.932	1.927	1.930	**1.867**
-4.240	1.917	1.866	1.933	**1.872**	-4.360	1.842	1.825	1.853	**1.868**
-4.230	1.842	1.857	1.889	**1.873**	-4.350	1.935	1.870	1.915	**1.868**
-4.220	1.883	1.882	1.843	**1.873**	-4.340	1.848	1.909	1.871	**1.868**
-4.210	1.887	1.886	1.831	**1.873**	-4.330	1.893	1.850	1.781	**1.868**
-4.200	1.876	1.844	1.821	**1.874**	-4.320	1.892	1.908	1.848	**1.868**
-4.190	1.878	1.880	1.890	**1.875**	-4.310	1.865	1.920	1.862	**1.868**
-4.180	1.894	1.910	1.890	**1.875**	-4.300	1.898	1.927	1.964	**1.869**
-4.170	1.787	1.852	1.847	**1.876**	-4.290	1.917	1.890	1.821	**1.869**
-4.160	1.879	1.932	1.882	**1.876**	-4.280	1.909	1.897	1.865	**1.869**
-4.150	1.846	1.854	1.814	**1.877**	-4.270	1.866	1.959	1.918	**1.869**
-4.140	1.857	1.891	1.882	**1.877**	-4.260	1.886	1.883	1.870	**1.870**
-4.130	1.921	1.902	1.913	**1.878**	-4.250	1.871	1.868	1.935	**1.870**
-4.120	1.858	1.881	1.934	**1.878**	-4.240	1.915	1.802	1.854	**1.871**
-4.110	1.901	1.926	1.957	**1.878**	-4.230	1.890	1.885	1.885	**1.871**
-4.100	1.817	1.852	1.835	**1.877**	-4.220	1.982	1.888	1.883	**1.872**
-4.090	1.829	1.899	1.831	**1.877**	-4.210	1.839	1.876	1.891	**1.872**
-4.080	1.870	1.849	1.884	**1.877**	-4.200	1.909	1.912	1.929	**1.872**
-4.070	1.846	1.895	1.854	**1.876**	-4.190	1.801	1.854	1.856	**1.872**
-4.060	1.880	1.801	1.842	**1.876**	-4.180	1.987	1.893	1.924	**1.873**
-4.050	1.897	1.893	1.892	**1.876**	-4.170	1.854	1.907	1.924	**1.873**
-4.040	1.878	1.937	1.887	**1.875**	-4.160	1.923	1.826	1.826	**1.873**
-4.030	1.876	1.865	1.881	**1.875**	-4.150	1.876	1.868	1.813	**1.873**
-4.020	1.874	1.880	1.930	**1.875**	-4.140	1.813	1.758	1.862	**1.873**
-4.010	1.824	1.886	1.892	**1.874**	-4.130	1.912	1.850	1.875	**1.874**

-4.000	1.898	1.916	1.928	**1.874**	-4.120	1.937	1.836	1.854	**1.874**
-3.990	1.865	1.874	1.890	**1.873**	-4.110	1.883	1.830	1.845	**1.874**
-3.980	1.910	1.915	1.921	**1.872**	-4.100	1.927	1.820	1.905	**1.874**
-3.970	1.882	1.863	1.851	**1.872**	-4.090	1.865	1.893	1.811	**1.874**
-3.960	1.865	1.883	1.837	**1.871**	-4.080	1.840	1.879	1.902	**1.874**
-3.950	1.844	1.824	1.871	**1.871**	-4.070	1.868	1.845	1.783	**1.874**
-3.940	1.841	1.794	1.864	**1.870**	-4.060	1.877	1.969	1.963	**1.874**
-3.930	1.895	1.895	1.866	**1.869**	-4.050	1.871	1.871	1.966	**1.874**
-3.920	1.852	1.880	1.879	**1.869**	-4.040	1.804	1.795	1.891	**1.874**
-3.910	1.831	1.845	1.882	**1.869**	-4.030	1.960	1.938	1.890	**1.874**
-3.900	1.843	1.851	1.855	**1.868**	-4.020	1.856	1.858	1.800	**1.874**
-3.890	1.873	1.889	1.874	**1.867**	-4.010	1.897	1.846	1.885	**1.874**
-3.880	1.923	1.911	1.924	**1.866**	-4.000	1.926	1.845	1.892	**1.874**
-3.870	1.825	1.828	1.864	**1.865**	-3.990	1.876	1.853	1.814	**1.874**
-3.860	1.796	1.852	1.866	**1.864**	-3.980	1.925	1.843	1.848	**1.875**
-3.850	1.820	1.859	1.864	**1.864**	-3.970	1.860	1.819	1.906	**1.875**
-3.840	1.875	1.929	1.890	**1.863**	-3.960	1.882	1.925	1.897	**1.875**
-3.830	1.860	1.819	1.835	**1.862**	-3.950	1.928	1.866	1.934	**1.875**
-3.820	1.877	1.890	1.912	**1.862**	-3.940	1.888	1.860	1.900	**1.875**
-3.810	1.846	1.870	1.900	**1.862**	-3.930	1.915	1.863	1.893	**1.875**
-3.800	1.904	1.893	1.903	**1.862**	-3.920	1.890	1.964	1.997	**1.875**
-3.790	1.852	1.890	1.873	**1.863**	-3.910	1.900	1.924	1.875	**1.875**
-3.780	1.832	1.853	1.902	**1.864**	-3.900	1.832	1.855	1.838	**1.875**
-3.770	1.802	1.844	1.871	**1.864**	-3.890	1.961	1.898	1.864	**1.875**
-3.760	1.828	1.850	1.865	**1.865**	-3.880	1.921	1.825	1.839	**1.875**
-3.750	1.836	1.898	1.880	**1.866**	-3.870	1.880	1.949	1.883	**1.875**
-3.740	1.846	1.891	1.886	**1.866**	-3.860	1.844	1.915	1.839	**1.874**
-3.730	1.906	1.900	1.890	**1.867**	-3.850	1.943	1.984	1.938	**1.874**
-3.720	1.840	1.881	1.845	**1.869**	-3.840	1.919	1.921	1.975	**1.874**
-3.710	1.843	1.854	1.861	**1.870**	-3.830	1.909	1.829	1.841	**1.874**
-3.700	1.866	1.864	1.931	**1.871**	-3.820	1.801	1.764	1.794	**1.873**
-3.690	1.887	1.920	1.851	**1.872**	-3.810	1.871	1.864	1.869	**1.873**

-3.680	1.884	1.867	1.909	**1.873**	-3.800	1.897	1.853	1.826	**1.873**
-3.670	1.905	1.900	1.849	**1.875**	-3.790	1.882	1.962	1.906	**1.873**
-3.660	1.891	1.870	1.866	**1.877**	-3.780	1.912	1.851	1.857	**1.874**
-3.650	1.845	1.857	1.916	**1.879**	-3.770	1.835	1.847	1.930	**1.874**
-3.640	1.882	1.871	1.855	**1.883**	-3.760	1.902	1.891	1.933	**1.874**
-3.630	1.844	1.897	1.890	**1.887**	-3.750	1.903	1.847	1.900	**1.874**
-3.620	1.869	1.824	1.882	**1.890**	-3.740	1.891	1.852	1.933	**1.874**
-3.610	1.903	1.839	1.897	**1.894**	-3.730	1.918	1.842	1.947	**1.874**
-3.600	1.951	1.917	1.928	**1.898**	-3.720	1.957	1.865	1.881	**1.876**
-3.590	1.942	1.938	1.939	**1.902**	-3.710	1.788	1.882	1.864	**1.877**
-3.580	1.947	1.885	1.943	**1.906**	-3.700	1.867	1.870	1.933	**1.878**
-3.570	1.922	1.874	1.930	**1.909**	-3.690	1.823	1.892	1.862	**1.879**
-3.560	1.906	1.901	1.855	**1.913**	-3.680	1.858	1.892	1.868	**1.880**
-3.550	1.907	1.968	1.947	**1.917**	-3.670	1.827	1.877	1.811	**1.882**
-3.540	1.911	1.912	1.946	**1.921**	-3.660	1.858	1.896	1.863	**1.883**
-3.530	1.958	1.961	1.912	**1.925**	-3.650	1.837	1.888	1.862	**1.884**
-3.520	1.967	1.976	1.954	**1.928**	-3.640	1.917	1.853	1.876	**1.886**
-3.510	1.906	1.905	1.958	**1.932**	-3.630	1.879	1.854	1.893	**1.889**
-3.500	1.959	1.894	1.889	**1.936**	-3.620	1.938	1.958	1.883	**1.891**
-3.490	1.884	1.837	1.912	**1.940**	-3.610	1.810	1.856	1.824	**1.893**
-3.480	1.914	1.975	1.933	**1.943**	-3.600	1.945	1.859	1.914	**1.896**
-3.470	1.987	1.952	1.917	**1.947**	-3.590	1.791	1.871	1.870	**1.899**
-3.460	1.979	1.909	1.957	**1.951**	-3.580	2.007	1.924	1.987	**1.902**
-3.450	1.926	1.988	1.971	**1.955**	-3.570	1.849	1.805	1.902	**1.906**
-3.440	1.984	1.940	1.949	**1.959**	-3.560	1.885	1.929	1.973	**1.909**
-3.430	1.994	1.999	1.948	**1.962**	-3.550	1.949	1.916	2.025	**1.913**
-3.420	1.946	1.943	1.973	**1.966**	-3.540	1.923	2.023	1.960	**1.916**
-3.410	1.990	2.036	1.964	**1.970**	-3.530	1.967	1.929	1.997	**1.920**
-3.400	1.970	1.917	1.950	**1.974**	-3.520	1.916	1.883	1.875	**1.923**
-3.390	1.963	1.940	1.934	**1.978**	-3.510	1.958	1.955	1.867	**1.926**
-3.380	1.961	2.001	1.936	**1.981**	-3.500	1.966	2.008	2.003	**1.930**
-3.370	2.025	1.972	1.998	**1.985**	-3.490	1.912	1.879	1.867	**1.933**

-3.360	1.982	1.935	2.002	**1.989**	-3.480	1.986	1.932	1.933	**1.937**
-3.350	2.016	2.035	1.987	**1.993**	-3.470	1.890	1.964	1.943	**1.940**
-3.340	1.960	1.990	1.981	**1.996**	-3.460	1.963	1.940	1.927	**1.944**
-3.330	1.997	1.954	1.999	**2.000**	-3.450	2.012	1.936	1.954	**1.947**
-3.320	1.978	2.006	2.008	**2.004**	-3.440	1.890	1.954	1.860	**1.951**
-3.310	2.047	2.021	2.025	**2.008**	-3.430	1.937	1.914	1.919	**1.954**
-3.300	2.050	2.009	1.976	**2.011**	-3.420	1.859	1.929	1.909	**1.958**
-3.290	1.985	2.019	2.025	**2.015**	-3.410	1.928	1.936	1.966	**1.961**
-3.280	2.040	1.993	1.987	**2.019**	-3.400	1.977	1.933	1.929	**1.965**
-3.270	2.000	2.036	2.032	**2.023**	-3.390	2.012	1.931	1.977	**1.968**
-3.260	2.032	1.984	2.043	**2.026**	-3.380	1.998	2.028	2.018	**1.972**
-3.250	2.050	1.995	2.026	**2.030**	-3.370	2.004	2.028	2.006	**1.975**
-3.240	2.034	2.014	2.043	**2.034**	-3.360	2.027	2.009	1.981	**1.979**
-3.230	2.037	2.029	2.068	**2.038**	-3.350	1.997	1.925	2.007	**1.982**
-3.220	2.018	2.071	2.033	**2.041**	-3.340	1.943	2.025	1.978	**1.986**
-3.210	2.047	2.046	2.056	**2.045**	-3.330	2.032	2.021	2.037	**1.990**
-3.200	2.011	1.993	2.056	**2.048**	-3.320	2.030	2.034	2.013	**1.994**
-3.190	2.067	2.054	2.064	**2.052**	-3.310	1.897	1.982	1.986	**1.998**
-3.180	2.060	1.997	2.020	**2.055**	-3.300	1.978	2.023	1.975	**2.001**
-3.170	2.020	2.009	2.021	**2.059**	-3.290	1.970	2.039	1.977	**2.005**
-3.160	2.021	2.042	2.047	**2.062**	-3.280	1.978	2.014	1.940	**2.009**
-3.150	2.089	2.023	2.069	**2.066**	-3.270	2.050	2.106	1.992	**2.013**
-3.140	2.045	2.085	2.019	**2.069**	-3.260	1.962	2.005	2.063	**2.016**
-3.130	2.060	2.091	2.056	**2.073**	-3.250	2.043	1.992	2.055	**2.020**
-3.120	2.055	2.067	2.100	**2.076**	-3.240	2.024	2.017	2.075	**2.024**
-3.110	2.062	2.039	2.023	**2.078**	-3.230	2.019	2.047	2.035	**2.028**
-3.100	2.117	2.114	2.101	**2.080**	-3.220	2.093	2.092	2.098	**2.032**
-3.090	2.070	2.111	2.074	**2.083**	-3.210	2.061	2.031	2.061	**2.035**
-3.080	2.103	2.139	2.112	**2.085**	-3.200	2.024	1.953	2.011	**2.039**
-3.070	2.076	2.133	2.142	**2.087**	-3.190	2.010	2.009	2.069	**2.043**
-3.060	2.096	2.124	2.064	**2.089**	-3.180	2.049	2.015	2.044	**2.047**
-3.050	2.103	2.050	2.072	**2.092**	-3.170	2.087	2.071	2.061	**2.051**

-3.040	2.063	2.101	2.131	**2.094**	-3.160	2.019	2.050	2.027	**2.054**
-3.030	2.090	2.109	2.053	**2.096**	-3.150	1.993	1.977	2.083	**2.058**
-3.020	2.112	2.122	2.129	**2.098**	-3.140	2.056	2.055	2.014	**2.062**
-3.010	2.092	2.140	2.117	**2.100**	-3.130	1.953	1.979	2.001	**2.066**
-3.000	2.060	2.109	2.118	**2.101**	-3.120	2.122	2.098	2.142	**2.069**
-2.990	2.091	2.056	2.096	**2.102**	-3.110	2.049	2.117	2.158	**2.073**
-2.980	2.144	2.120	2.088	**2.102**	-3.100	2.112	2.061	2.112	**2.077**
-2.970	2.137	2.122	2.143	**2.103**	-3.090	2.053	2.069	2.126	**2.080**
-2.960	2.132	2.101	2.116	**2.104**	-3.080	2.118	2.040	2.025	**2.083**
-2.950	2.134	2.096	2.071	**2.104**	-3.070	2.065	2.067	2.072	**2.086**
-2.940	2.138	2.084	2.088	**2.105**	-3.060	2.028	2.019	2.028	**2.089**
-2.930	2.100	2.131	2.090	**2.105**	-3.050	2.116	2.008	2.049	**2.092**
-2.920	2.058	2.115	2.080	**2.106**	-3.040	2.109	2.125	2.119	**2.094**
-2.910	2.067	2.074	2.076	**2.107**	-3.030	2.148	2.164	2.060	**2.096**
-2.900	2.130	2.141	2.102	**2.107**	-3.020	2.097	2.083	2.176	**2.099**
-2.890	2.052	2.059	2.075	**2.108**	-3.010	2.183	2.120	2.134	**2.101**
-2.880	2.102	2.129	2.086	**2.109**	-3.000	2.186	2.117	2.119	**2.102**
-2.870	2.134	2.119	2.084	**2.109**	-2.990	2.076	2.155	2.102	**2.103**
-2.860	2.048	2.118	2.112	**2.110**	-2.980	2.077	2.156	2.111	**2.104**
-2.850	2.131	2.077	2.134	**2.110**	-2.970	2.115	2.104	2.034	**2.105**
-2.840	2.162	2.119	2.166	**2.110**	-2.960	2.113	2.072	2.157	**2.105**
-2.830	2.121	2.095	2.127	**2.110**	-2.950	2.052	2.126	2.073	**2.106**
-2.820	2.060	2.059	2.091	**2.110**	-2.940	2.105	2.071	2.043	**2.107**
-2.810	2.061	2.106	2.087	**2.109**	-2.930	2.101	2.139	2.105	**2.107**
-2.800	2.120	2.083	2.073	**2.109**	-2.920	2.152	2.139	2.151	**2.108**
-2.790	2.096	2.118	2.153	**2.109**	-2.910	2.155	2.180	2.109	**2.109**
-2.780	2.100	2.091	2.119	**2.109**	-2.900	2.125	2.158	2.168	**2.110**
-2.770	2.095	2.053	2.082	**2.109**	-2.890	2.083	2.031	2.060	**2.110**
-2.760	2.148	2.138	2.124	**2.109**	-2.880	2.140	2.153	2.057	**2.111**
-2.750	2.136	2.086	2.097	**2.109**	-2.870	2.149	2.129	2.150	**2.112**
-2.740	2.118	2.091	2.152	**2.109**	-2.860	2.184	2.118	2.192	**2.113**
-2.730	2.086	2.098	2.144	**2.109**	-2.850	2.123	2.062	2.124	**2.114**

-2.720	2.163	2.101	2.155	**2.108**	-2.840	2.107	2.157	2.100	**2.114**
-2.710	2.068	2.093	2.104	**2.108**	-2.830	2.121	2.020	2.088	**2.115**
-2.700	2.099	2.072	2.052	**2.108**	-2.820	2.154	2.117	2.178	**2.116**
-2.690	2.097	2.168	2.151	**2.108**	-2.810	2.142	2.129	2.086	**2.117**
-2.680	2.117	2.146	2.101	**2.108**	-2.800	2.168	2.169	2.072	**2.116**
-2.670	2.118	2.104	2.118	**2.108**	-2.790	2.177	2.134	2.116	**2.116**
-2.660	2.064	2.132	2.108	**2.108**	-2.780	2.091	2.115	2.158	**2.116**
-2.650	2.106	2.049	2.073	**2.108**	-2.770	2.120	2.144	2.068	**2.116**
-2.640	2.106	2.149	2.161	**2.108**	-2.760	2.178	2.123	2.139	**2.116**
-2.630	2.109	2.105	2.080	**2.108**	-2.750	2.160	2.107	2.082	**2.115**
-2.620	2.087	2.145	2.073	**2.108**	-2.740	2.111	2.191	2.155	**2.115**
-2.610	2.080	2.147	2.094	**2.108**	-2.730	2.159	2.083	2.189	**2.115**
-2.600	2.136	2.150	2.108	**2.108**	-2.720	2.068	2.060	2.103	**2.115**
-2.590	2.107	2.138	2.103	**2.108**	-2.710	2.026	2.100	2.099	**2.115**
-2.580	2.136	2.138	2.074	**2.107**	-2.700	2.095	2.099	2.134	**2.114**
-2.570	2.094	2.118	2.120	**2.107**	-2.690	2.062	2.097	2.103	**2.114**
-2.560	2.088	2.106	2.088	**2.107**	-2.680	2.101	2.167	2.097	**2.114**
-2.550	2.066	2.086	2.058	**2.107**	-2.670	2.107	2.160	2.104	**2.114**
-2.540	2.093	2.161	2.112	**2.107**	-2.660	2.110	2.057	2.141	**2.114**
-2.530	2.151	2.106	2.089	**2.106**	-2.650	2.103	2.015	2.098	**2.114**
-2.520	2.108	2.127	2.109	**2.106**	-2.640	2.095	2.096	2.061	**2.115**
-2.510	2.110	2.050	2.115	**2.106**	-2.630	2.157	2.130	2.083	**2.115**
-2.500	2.110	2.101	2.138	**2.106**	-2.620	2.163	2.087	2.079	**2.116**
-2.490	2.127	2.136	2.074	**2.105**	-2.610	2.041	2.106	2.143	**2.116**
-2.480	2.085	2.117	2.075	**2.105**	-2.600	2.091	2.024	2.124	**2.117**
-2.470	2.136	2.153	2.167	**2.105**	-2.590	2.126	2.068	2.035	**2.117**
-2.460	2.067	2.126	2.089	**2.105**	-2.580	2.184	2.080	2.121	**2.117**
-2.450	2.076	2.119	2.075	**2.105**	-2.570	2.101	2.125	2.141	**2.118**
-2.440	2.105	2.111	2.097	**2.105**	-2.560	2.090	2.055	2.141	**2.118**
-2.430	2.108	2.114	2.091	**2.105**	-2.550	2.130	2.086	2.103	**2.118**
-2.420	2.091	2.080	2.124	**2.104**	-2.540	2.079	2.134	2.028	**2.118**
-2.410	2.121	2.128	2.058	**2.105**	-2.530	2.136	2.124	2.152	**2.118**

-2.400	2.084	2.086	2.069	**2.105**	-2.520	2.101	2.189	2.090	**2.118**
-2.390	2.104	2.092	2.053	**2.105**	-2.510	2.126	2.096	2.019	**2.118**
-2.380	2.040	2.063	2.053	**2.105**	-2.500	2.110	2.112	2.135	**2.118**
-2.370	2.078	2.052	2.098	**2.106**	-2.490	2.151	2.091	2.091	**2.118**
-2.360	2.089	2.088	2.124	**2.106**	-2.480	2.092	2.116	2.117	**2.118**
-2.350	2.099	2.095	2.029	**2.106**	-2.470	2.048	2.146	2.116	**2.118**
-2.340	2.077	2.125	2.047	**2.106**	-2.460	2.136	2.183	2.097	**2.118**
-2.330	2.128	2.201	2.150	**2.106**	-2.450	2.011	2.053	1.994	**2.117**
-2.320	2.143	2.121	2.117	**2.106**	-2.440	2.136	2.052	2.102	**2.117**
-2.310	2.083	2.076	2.098	**2.106**	-2.430	2.113	2.032	2.083	**2.117**
-2.300	2.140	2.079	2.112	**2.106**	-2.420	2.079	2.171	2.152	**2.117**
-2.290	2.090	2.072	2.068	**2.106**	-2.410	2.090	2.111	2.131	**2.117**
-2.280	2.076	2.131	2.078	**2.106**	-2.400	2.093	2.078	2.088	**2.118**
-2.270	2.125	2.139	2.065	**2.106**	-2.390	2.227	2.148	2.204	**2.118**
-2.260	2.133	2.101	2.120	**2.106**	-2.380	2.171	2.060	2.106	**2.119**
-2.250	2.062	2.118	2.084	**2.105**	-2.370	2.106	2.117	2.148	**2.119**
-2.240	2.116	2.074	2.047	**2.105**	-2.360	2.163	2.128	2.191	**2.120**
-2.230	2.095	2.073	2.119	**2.105**	-2.350	2.147	2.099	2.056	**2.121**
-2.220	2.086	2.140	2.114	**2.105**	-2.340	2.118	2.097	2.176	**2.121**
-2.210	2.087	2.111	2.117	**2.105**	-2.330	2.094	2.114	2.124	**2.122**
-2.200	2.136	2.137	2.115	**2.105**	-2.320	2.148	2.108	2.147	**2.122**
-2.190	2.081	2.093	2.089	**2.105**	-2.310	2.105	2.109	2.045	**2.123**
-2.180	2.124	2.094	2.072	**2.106**	-2.300	2.159	2.125	2.089	**2.123**
-2.170	2.098	2.076	2.095	**2.106**	-2.290	2.124	2.046	2.142	**2.124**
-2.160	2.045	2.112	2.078	**2.106**	-2.280	2.137	2.099	2.068	**2.124**
-2.150	2.180	2.154	2.129	**2.106**	-2.270	2.098	2.016	2.030	**2.125**
-2.140	2.073	2.093	2.085	**2.106**	-2.260	2.064	2.138	2.159	**2.125**
-2.130	2.147	2.164	2.133	**2.106**	-2.250	2.177	2.140	2.070	**2.126**
-2.120	2.142	2.178	2.117	**2.107**	-2.240	2.056	2.111	2.037	**2.126**
-2.110	2.066	2.093	2.113	**2.107**	-2.230	2.116	2.090	2.160	**2.127**
-2.100	2.107	2.098	2.095	**2.107**	-2.220	2.115	2.106	2.099	**2.127**
-2.090	2.155	2.129	2.101	**2.108**	-2.210	2.196	2.168	2.186	**2.128**

-2.080	2.130	2.118	2.097	**2.108**	-2.200	2.118	2.104	2.029	**2.128**
-2.070	2.092	2.121	2.154	**2.108**	-2.190	2.073	2.154	2.106	**2.128**
-2.060	2.149	2.084	2.150	**2.108**	-2.180	2.131	2.083	2.171	**2.128**
-2.050	2.051	2.093	2.121	**2.109**	-2.170	2.028	2.123	2.071	**2.128**
-2.040	2.043	2.030	2.034	**2.109**	-2.160	2.168	2.135	2.202	**2.128**
-2.030	2.115	2.076	2.133	**2.109**	-2.150	2.159	2.093	2.123	**2.128**
-2.020	2.121	2.155	2.076	**2.109**	-2.140	2.154	2.155	2.056	**2.128**
-2.010	2.116	2.054	2.047	**2.110**	-2.130	2.172	2.224	2.160	**2.129**
-2.000	2.106	2.137	2.079	**2.110**	-2.120	2.048	2.161	2.083	**2.129**
-1.990	2.092	2.076	2.116	**2.110**	-2.110	2.106	2.078	2.167	**2.129**
-1.980	2.108	2.062	2.103	**2.111**	-2.100	2.074	2.169	2.159	**2.129**
-1.970	2.087	2.084	2.129	**2.111**	-2.090	2.160	2.150	2.141	**2.129**
-1.960	2.115	2.114	2.150	**2.111**	-2.080	2.126	2.114	2.130	**2.130**
-1.950	2.128	2.148	2.154	**2.111**	-2.070	2.050	2.104	2.098	**2.130**
-1.940	2.079	2.087	2.040	**2.112**	-2.060	2.075	2.088	2.179	**2.130**
-1.930	2.139	2.183	2.123	**2.112**	-2.050	2.131	2.105	2.098	**2.131**
-1.920	2.116	2.123	2.137	**2.112**	-2.040	2.184	2.161	2.101	**2.131**
-1.910	2.073	2.089	2.088	**2.112**	-2.030	2.128	2.129	2.109	**2.131**
-1.900	2.101	2.150	2.127	**2.113**	-2.020	2.189	2.191	2.110	**2.132**
-1.890	2.078	2.116	2.090	**2.113**	-2.010	2.137	2.172	2.190	**2.132**
-1.880	2.071	2.126	2.144	**2.113**	-2.000	2.082	2.049	2.073	**2.132**
-1.870	2.092	2.156	2.143	**2.113**	-1.990	2.109	2.161	2.174	**2.132**
-1.860	2.076	2.145	2.103	**2.113**	-1.980	2.066	2.165	2.103	**2.132**
-1.850	2.133	2.097	2.068	**2.113**	-1.970	2.129	2.162	2.143	**2.133**
-1.840	2.116	2.084	2.074	**2.113**	-1.960	2.167	2.110	2.187	**2.133**
-1.830	2.089	2.045	2.105	**2.113**	-1.950	2.224	2.149	2.112	**2.133**
-1.820	2.123	2.121	2.124	**2.113**	-1.940	2.137	2.188	2.075	**2.133**
-1.810	2.072	2.064	2.061	**2.112**	-1.930	2.107	2.173	2.173	**2.133**
-1.800	2.099	2.083	2.117	**2.112**	-1.920	2.134	2.145	2.040	**2.134**
-1.790	2.117	2.088	2.059	**2.112**	-1.910	2.187	2.169	2.093	**2.133**
-1.780	2.099	2.121	2.150	**2.111**	-1.900	2.135	2.122	2.151	**2.133**
-1.770	2.086	2.128	2.069	**2.111**	-1.890	2.094	2.060	2.149	**2.132**

-1.760	2.146	2.159	2.091	**2.110**	-1.880	2.128	2.017	2.051	**2.132**
-1.750	2.086	2.057	2.084	**2.110**	-1.870	2.159	2.118	2.148	**2.131**
-1.740	2.140	2.142	2.103	**2.109**	-1.860	2.142	2.090	2.111	**2.130**
-1.730	2.052	2.092	2.090	**2.109**	-1.850	2.161	2.120	2.124	**2.130**
-1.720	2.117	2.192	2.157	**2.108**	-1.840	2.095	2.092	2.099	**2.129**
-1.710	2.012	2.039	2.036	**2.108**	-1.830	2.154	2.142	2.095	**2.128**
-1.700	2.067	2.115	2.119	**2.108**	-1.820	2.089	2.149	2.169	**2.127**
-1.690	2.114	2.104	2.118	**2.108**	-1.810	2.113	2.078	2.133	**2.126**
-1.680	2.152	2.095	2.076	**2.108**	-1.800	2.191	2.111	2.121	**2.125**
-1.670	2.093	2.166	2.137	**2.108**	-1.790	2.121	2.184	2.174	**2.124**
-1.660	2.065	2.124	2.071	**2.108**	-1.780	2.175	2.113	2.085	**2.123**
-1.650	2.140	2.086	2.123	**2.107**	-1.770	2.186	2.108	2.214	**2.122**
-1.640	2.077	2.110	2.048	**2.107**	-1.760	2.223	2.155	2.244	**2.121**
-1.630	2.098	2.096	2.082	**2.107**	-1.750	2.168	2.111	2.193	**2.120**
-1.620	2.109	2.107	2.075	**2.107**	-1.740	2.073	2.098	2.172	**2.119**
-1.610	2.105	2.148	2.144	**2.107**	-1.730	2.093	2.179	2.121	**2.118**
-1.600	2.174	2.140	2.114	**2.107**	-1.720	2.099	2.193	2.116	**2.117**
-1.590	2.101	2.077	2.065	**2.107**	-1.710	2.125	2.082	2.079	**2.116**
-1.580	2.046	2.070	2.018	**2.107**	-1.700	2.119	2.092	2.179	**2.114**
-1.570	2.180	2.114	2.176	**2.106**	-1.690	2.114	2.162	2.091	**2.113**
-1.560	2.105	2.093	2.086	**2.106**	-1.680	2.092	2.043	2.073	**2.112**
-1.550	2.156	2.159	2.154	**2.106**	-1.670	2.045	2.108	2.142	**2.111**
-1.540	2.055	2.110	2.099	**2.105**	-1.660	2.153	2.106	2.115	**2.110**
-1.530	2.124	2.091	2.111	**2.105**	-1.650	2.112	2.085	2.151	**2.109**
-1.520	2.141	2.136	2.174	**2.105**	-1.640	2.097	2.154	2.042	**2.109**
-1.510	2.140	2.134	2.109	**2.105**	-1.630	2.035	2.012	2.113	**2.108**
-1.500	2.072	2.142	2.080	**2.104**	-1.620	2.104	2.117	2.137	**2.107**
-1.490	2.140	2.133	2.120	**2.104**	-1.610	2.142	2.078	2.121	**2.107**
-1.480	2.091	2.133	2.110	**2.104**	-1.600	2.110	2.093	2.154	**2.106**
-1.470	2.095	2.154	2.157	**2.104**	-1.590	2.123	2.141	2.089	**2.105**
-1.460	2.002	2.058	2.066	**2.103**	-1.580	2.147	2.098	2.032	**2.105**
-1.450	2.074	2.133	2.116	**2.103**	-1.570	2.057	2.064	2.132	**2.104**

-1.440	2.122	2.108	2.098	**2.103**	-1.560	2.113	2.114	2.100	**2.103**
-1.430	2.112	2.088	2.151	**2.103**	-1.550	2.056	2.129	2.065	**2.103**
-1.420	2.091	2.121	2.104	**2.102**	-1.540	2.167	2.161	2.102	**2.102**
-1.410	2.119	2.116	2.112	**2.102**	-1.530	2.177	2.171	2.118	**2.102**
-1.400	2.059	2.122	2.092	**2.101**	-1.520	2.085	2.074	2.116	**2.101**
-1.390	2.153	2.120	2.088	**2.101**	-1.510	2.075	2.053	2.101	**2.101**
-1.380	2.110	2.103	2.117	**2.100**	-1.500	2.092	2.043	2.154	**2.100**
-1.370	2.090	2.062	2.111	**2.100**	-1.490	2.105	2.077	2.053	**2.100**
-1.360	2.078	2.112	2.091	**2.100**	-1.480	2.083	2.094	2.087	**2.099**
-1.350	2.072	2.100	2.093	**2.099**	-1.470	2.053	2.132	2.060	**2.099**
-1.340	2.126	2.125	2.084	**2.099**	-1.460	2.059	2.107	2.113	**2.098**
-1.330	2.091	2.098	2.110	**2.099**	-1.450	2.187	2.094	2.092	**2.098**
-1.320	2.066	2.081	2.137	**2.099**	-1.440	2.097	2.175	2.065	**2.097**
-1.310	2.117	2.129	2.117	**2.099**	-1.430	2.081	2.149	2.169	**2.097**
-1.300	2.086	2.143	2.121	**2.099**	-1.420	2.147	2.098	2.124	**2.096**
-1.290	2.074	2.153	2.103	**2.098**	-1.410	2.084	2.020	2.024	**2.095**
-1.280	2.129	2.083	2.085	**2.098**	-1.400	2.156	2.128	2.087	**2.094**
-1.270	2.076	2.104	2.080	**2.098**	-1.390	2.087	2.130	2.100	**2.094**
-1.260	2.124	2.093	2.068	**2.097**	-1.380	2.077	2.054	2.042	**2.093**
-1.250	2.107	2.124	2.126	**2.097**	-1.370	2.169	2.116	2.101	**2.092**
-1.240	2.158	2.137	2.086	**2.097**	-1.360	2.159	2.051	2.099	**2.092**
-1.230	2.107	2.119	2.049	**2.096**	-1.350	2.056	2.051	2.095	**2.091**
-1.220	2.072	2.081	2.123	**2.096**	-1.340	2.116	2.136	2.115	**2.091**
-1.210	2.106	2.120	2.157	**2.096**	-1.330	2.142	2.091	2.073	**2.090**
-1.200	2.094	2.092	2.076	**2.096**	-1.320	2.097	2.042	2.122	**2.090**
-1.190	2.071	2.111	2.120	**2.095**	-1.310	2.066	2.020	2.104	**2.089**
-1.180	2.080	2.089	2.082	**2.095**	-1.300	2.041	2.107	2.147	**2.089**
-1.170	2.095	2.068	2.084	**2.095**	-1.290	2.171	2.140	2.113	**2.089**
-1.160	2.142	2.109	2.130	**2.094**	-1.280	2.100	2.080	2.076	**2.089**
-1.150	2.144	2.168	2.096	**2.094**	-1.270	2.144	2.049	2.062	**2.089**
-1.140	2.117	2.080	2.099	**2.094**	-1.260	2.064	2.111	2.030	**2.090**
-1.130	2.161	2.125	2.123	**2.094**	-1.250	1.999	2.074	2.051	**2.090**

-1.120	2.143	2.078	2.084	**2.093**	-1.240	2.092	2.156	2.091	**2.090**
-1.110	2.073	2.135	2.127	**2.093**	-1.230	2.066	2.098	2.124	**2.090**
-1.100	2.062	2.029	2.083	**2.093**	-1.220	2.013	2.056	2.092	**2.090**
-1.090	2.083	2.123	2.132	**2.094**	-1.210	2.064	2.135	2.159	**2.090**
-1.080	2.056	2.104	2.079	**2.094**	-1.200	2.111	2.076	2.028	**2.090**
-1.070	2.129	2.127	2.157	**2.095**	-1.190	2.116	2.066	2.096	**2.090**
-1.060	2.084	2.143	2.090	**2.096**	-1.180	2.079	2.064	2.103	**2.090**
-1.050	2.121	2.103	2.086	**2.097**	-1.170	2.137	2.083	2.070	**2.090**
-1.040	2.058	2.098	2.103	**2.098**	-1.160	2.082	2.071	2.124	**2.090**
-1.030	2.100	2.099	2.089	**2.098**	-1.150	2.148	2.071	2.169	**2.090**
-1.020	2.122	2.129	2.133	**2.099**	-1.140	2.141	2.092	2.111	**2.090**
-1.010	2.092	2.082	2.069	**2.099**	-1.130	2.116	2.068	2.123	**2.090**
-1.000	2.095	2.102	2.084	**2.100**	-1.120	2.026	2.108	2.040	**2.090**
-0.990	2.096	2.051	2.074	**2.100**	-1.110	2.038	2.063	2.023	**2.090**
-0.980	2.077	2.064	2.058	**2.100**	-1.100	2.154	2.158	2.067	**2.090**
-0.970	2.123	2.110	2.107	**2.101**	-1.090	2.136	2.104	2.047	**2.090**
-0.960	2.080	2.127	2.137	**2.101**	-1.080	2.119	2.123	2.092	**2.090**
-0.950	2.091	2.136	2.168	**2.101**	-1.070	2.088	2.050	2.018	**2.091**
-0.940	2.069	2.066	2.054	**2.101**	-1.060	2.125	2.071	2.015	**2.091**
-0.930	2.092	2.080	2.110	**2.101**	-1.050	2.072	2.024	1.965	**2.091**
-0.920	2.059	2.089	2.107	**2.101**	-1.040	2.196	2.108	2.150	**2.092**
-0.910	2.090	2.153	2.097	**2.101**	-1.030	2.109	2.109	2.089	**2.093**
-0.900	2.155	2.114	2.123	**2.101**	-1.020	2.088	2.058	1.997	**2.093**
-0.890	2.048	2.061	2.090	**2.101**	-1.010	2.072	2.042	2.024	**2.094**
-0.880	2.091	2.093	2.146	**2.101**	-1.000	2.107	2.037	2.076	**2.094**
-0.870	2.071	2.127	2.057	**2.101**	-0.990	2.097	2.036	2.099	**2.095**
-0.860	2.089	2.060	2.120	**2.101**	-0.980	2.094	2.188	2.106	**2.095**
-0.850	2.065	2.100	2.072	**2.101**	-0.970	2.066	2.049	2.118	**2.096**
-0.840	2.031	2.078	2.093	**2.101**	-0.960	2.028	2.061	1.982	**2.096**
-0.830	2.118	2.127	2.081	**2.101**	-0.950	2.098	2.131	2.078	**2.096**
-0.820	2.109	2.085	2.106	**2.101**	-0.940	1.991	2.058	2.102	**2.097**
-0.810	2.046	2.123	2.096	**2.101**	-0.930	2.106	2.159	2.094	**2.097**

-0.800	2.094	2.131	2.055	**2.101**	-0.920	2.070	2.185	2.130	**2.098**
-0.790	2.132	2.058	2.113	**2.101**	-0.910	2.029	2.130	2.118	**2.098**
-0.780	2.092	2.083	2.127	**2.101**	-0.900	2.086	2.119	2.089	**2.098**
-0.770	2.141	2.122	2.109	**2.101**	-0.890	2.151	2.090	2.147	**2.099**
-0.760	2.095	2.027	2.053	**2.101**	-0.880	2.186	2.098	2.201	**2.099**
-0.750	2.144	2.097	2.093	**2.101**	-0.870	2.104	2.124	2.092	**2.099**
-0.740	2.101	2.108	2.095	**2.101**	-0.860	2.108	2.140	2.122	**2.099**
-0.730	2.131	2.085	2.052	**2.101**	-0.850	2.117	2.046	2.006	**2.100**
-0.720	2.138	2.094	2.101	**2.101**	-0.840	2.054	2.139	2.060	**2.100**
-0.710	2.117	2.080	2.124	**2.101**	-0.830	2.075	2.054	2.080	**2.100**
-0.700	2.079	2.087	2.111	**2.101**	-0.820	2.136	2.136	2.121	**2.101**
-0.690	2.150	2.097	2.133	**2.101**	-0.810	2.089	2.148	2.141	**2.101**
-0.680	2.086	2.087	2.060	**2.101**	-0.800	2.108	2.062	2.007	**2.101**
-0.670	2.075	2.121	2.067	**2.101**	-0.790	1.987	2.053	2.064	**2.101**
-0.660	2.082	2.085	2.143	**2.101**	-0.780	2.092	2.121	2.096	**2.101**
-0.650	2.067	2.124	2.126	**2.101**	-0.770	2.138	2.131	2.105	**2.101**
-0.640	2.113	2.119	2.079	**2.101**	-0.760	2.207	2.113	2.142	**2.101**
-0.630	2.138	2.133	2.116	**2.101**	-0.750	2.049	2.069	2.044	**2.101**
-0.620	2.022	2.076	2.078	**2.101**	-0.740	2.074	2.099	2.047	**2.102**
-0.610	2.154	2.142	2.086	**2.101**	-0.730	2.048	2.130	2.096	**2.102**
-0.600	2.162	2.110	2.128	**2.101**	-0.720	2.125	2.083	2.061	**2.102**
-0.590	2.012	2.076	2.071	**2.100**	-0.710	2.084	2.097	2.020	**2.102**
-0.580	2.096	2.114	2.104	**2.100**	-0.700	2.115	2.113	2.091	**2.102**
-0.570	2.120	2.151	2.090	**2.100**	-0.690	2.076	2.093	1.989	**2.102**
-0.560	2.099	2.161	2.153	**2.100**	-0.680	2.175	2.131	2.086	**2.102**
-0.550	2.080	2.091	2.080	**2.100**	-0.670	2.114	2.119	2.180	**2.102**
-0.540	2.151	2.124	2.134	**2.100**	-0.660	2.037	2.071	2.098	**2.102**
-0.530	2.100	2.081	2.080	**2.100**	-0.650	2.062	2.121	2.109	**2.102**
-0.520	2.079	2.098	2.117	**2.100**	-0.640	2.118	2.145	2.171	**2.102**
-0.510	2.072	2.123	2.102	**2.100**	-0.630	2.054	2.149	2.119	**2.102**
-0.500	2.077	2.098	2.128	**2.100**	-0.620	2.053	2.162	2.130	**2.102**
-0.490	2.063	2.106	2.104	**2.100**	-0.610	2.136	2.095	2.049	**2.102**

-0.480	2.130	2.065	2.092	**2.100**
-0.470	2.068	2.064	2.063	**2.100**
-0.460	2.117	2.108	2.047	**2.100**
-0.450	2.093	2.076	2.068	**2.100**
-0.440	2.100	2.095	2.102	**2.100**
-0.430	2.084	2.059	2.026	**2.100**
-0.420	2.106	2.083	2.065	**2.100**
-0.410	2.089	2.117	2.100	**2.100**
-0.400	2.129	2.124	2.131	**2.100**
-0.390	2.061	1.984	2.010	**2.100**
-0.380	2.072	2.114	2.144	**2.100**
-0.370	2.125	2.146	2.114	**2.100**
-0.360	2.115	2.111	2.109	**2.100**
-0.350	2.130	2.104	2.113	**2.100**
-0.340	2.183	2.112	2.117	**2.100**
-0.330	2.105	2.122	2.087	**2.100**

-0.600	2.152	2.109	2.194	**2.102**
-0.590	2.042	2.047	2.124	**2.102**
-0.580	2.112	2.114	2.021	**2.102**
-0.570	2.025	2.051	2.094	**2.102**
-0.560	2.096	2.110	2.007	**2.102**
-0.550	2.052	2.039	2.087	**2.102**
-0.540	2.098	2.178	2.113	**2.102**
-0.530	2.095	2.200	2.132	**2.102**
-0.520	2.014	1.950	2.039	**2.102**
-0.510	2.086	2.112	2.057	**2.102**
-0.500	2.135	2.169	2.096	**2.102**
-0.490	2.008	2.042	2.118	**2.102**
-0.480	2.146	2.118	2.155	**2.102**
-0.470	2.157	2.198	2.127	**2.102**
-0.460	2.129	2.064	2.115	**2.102**
-0.450	2.086	2.088	2.139	**2.102**
-0.440	2.189	2.142	2.101	**2.102**
-0.430	2.110	2.056	2.104	**2.102**
-0.420	2.019	2.058	2.016	**2.102**
-0.410	2.097	2.101	2.196	**2.102**
-0.400	2.003	2.094	2.088	**2.102**
-0.390	2.087	2.194	2.138	**2.102**
-0.380	2.112	2.133	2.059	**2.102**
-0.370	1.956	2.058	2.027	**2.102**
-0.360	2.127	2.045	2.125	**2.102**
-0.350	2.122	2.174	2.085	**2.102**
-0.340	2.062	2.116	2.103	**2.102**
-0.330	2.132	2.170	2.193	**2.102**
-0.320	2.086	2.043	2.142	**2.102**
-0.310	2.107	2.052	2.126	**2.102**
-0.300	2.052	2.077	2.126	**2.102**
-0.290	2.228	2.180	2.211	**2.102**

-0.280	2.075	2.165	2.120	**2.102**
-0.270	2.063	2.165	2.077	**2.102**
-0.260	2.065	2.092	2.114	**2.102**
-0.250	2.035	2.071	2.065	**2.102**
-0.24	2.18	2.14	2.07	**2.10**
-0.23	2.07	2.04	2.08	**2.10**
-0.22	2.03	2.06	1.96	**2.10**

Protocol	AT			
Hole nr.	40			
Hole depth (mm)	Meas_1 Radius (mm)	Meas_2 Radius (mm)	Meas_3 Radius (mm)	**Mean Radius (mm)**
-13.950	0.434	0.470	0.393	**0.422**
-13.940	0.416	0.442	0.402	**0.454**
-13.930	0.456	0.493	0.478	**0.486**
-13.920	0.508	0.524	0.498	**0.518**
-13.910	0.558	0.527	0.559	**0.550**
-13.900	0.542	0.570	0.577	**0.581**
-13.890	0.626	0.645	0.698	**0.612**
-13.880	0.611	0.652	0.619	**0.642**
-13.870	0.685	0.643	0.683	**0.672**
-13.860	0.668	0.686	0.722	**0.701**
-13.850	0.704	0.683	0.750	**0.729**
-13.840	0.777	0.793	0.766	**0.758**
-13.830	0.765	0.770	0.800	**0.788**
-13.820	0.822	0.837	0.868	**0.819**
-13.810	0.818	0.834	0.845	**0.850**
-13.800	0.820	0.844	0.850	**0.870**
-13.790	0.823	0.870	0.825	**0.884**
-13.780	0.887	0.873	0.905	**0.898**
-13.770	0.832	0.839	0.898	**0.911**
-13.760	0.908	0.947	0.911	**0.922**
-13.750	0.961	0.912	0.912	**0.932**
-13.740	0.957	0.954	0.935	**0.943**
-13.730	0.977	1.013	0.975	**0.954**
-13.720	0.949	0.918	0.913	**0.964**
-13.710	0.989	0.966	1.012	**0.975**
-13.700	0.987	0.971	0.938	**0.986**
-13.690	1.033	1.079	1.064	**0.996**

Protocol	AT			
Hole nr.	50			
Hole depth (mm)	Meas_1 Radius (mm)	Meas_2 Radius (mm)	Meas_3 Radius (mm)	**Mean Radius (mm)**
-13.990	0.264	0.263	0.302	**0.277**
-13.980	0.340	0.420	0.329	**0.313**
-13.970	0.340	0.331	0.373	**0.339**
-13.960	0.382	0.420	0.436	**0.371**
-13.950	0.404	0.384	0.424	**0.406**
-13.940	0.424	0.350	0.409	**0.433**
-13.930	0.481	0.516	0.414	**0.459**
-13.920	0.467	0.384	0.427	**0.487**
-13.910	0.542	0.553	0.494	**0.514**
-13.900	0.497	0.550	0.549	**0.540**
-13.890	0.555	0.611	0.516	**0.565**
-13.880	0.659	0.591	0.568	**0.591**
-13.870	0.557	0.639	0.571	**0.620**
-13.860	0.645	0.659	0.635	**0.650**
-13.850	0.756	0.672	0.710	**0.679**
-13.840	0.719	0.722	0.728	**0.710**
-13.830	0.757	0.723	0.760	**0.740**
-13.820	0.749	0.760	0.691	**0.769**
-13.810	0.773	0.797	0.843	**0.792**
-13.800	0.808	0.809	0.788	**0.814**
-13.790	0.801	0.849	0.838	**0.830**
-13.780	0.856	0.807	0.900	**0.847**
-13.770	0.814	0.813	0.882	**0.863**
-13.760	0.862	0.824	0.879	**0.877**
-13.750	0.870	0.954	0.893	**0.891**
-13.740	0.903	0.942	0.876	**0.904**
-13.730	0.960	0.928	0.957	**0.916**

-13.680	0.984	0.987	0.960	**1.007**	-13.720	0.954	0.876	0.924	**0.928**
-13.670	1.010	0.953	0.964	**1.018**	-13.710	0.977	0.938	0.908	**0.941**
-13.660	1.054	1.066	1.027	**1.028**	-13.700	0.919	0.963	0.911	**0.952**
-13.650	1.055	1.085	1.038	**1.038**	-13.690	0.929	1.010	0.942	**0.962**
-13.640	1.055	1.090	1.074	**1.048**	-13.680	0.884	0.969	0.942	**0.972**
-13.630	1.065	1.053	1.040	**1.057**	-13.670	0.999	0.958	1.037	**0.982**
-13.620	1.080	1.071	1.037	**1.067**	-13.660	0.931	0.982	0.965	**0.991**
-13.610	1.043	1.084	1.024	**1.076**	-13.650	1.033	1.012	1.002	**1.001**
-13.600	1.106	1.082	1.040	**1.086**	-13.640	0.970	1.014	1.020	**1.010**
-13.590	1.021	1.057	1.019	**1.095**	-13.630	1.012	1.026	1.078	**1.020**
-13.580	1.047	1.101	1.074	**1.105**	-13.620	1.039	0.973	1.033	**1.029**
-13.570	1.140	1.149	1.082	**1.114**	-13.610	1.032	1.059	1.076	**1.039**
-13.560	1.117	1.172	1.109	**1.124**	-13.600	1.099	1.140	1.111	**1.048**
-13.550	1.121	1.100	1.171	**1.133**	-13.590	1.156	1.099	1.083	**1.058**
-13.540	1.133	1.118	1.149	**1.143**	-13.580	1.063	1.011	0.963	**1.067**
-13.530	1.149	1.165	1.160	**1.152**	-13.570	1.121	1.210	1.153	**1.077**
-13.520	1.134	1.161	1.176	**1.161**	-13.560	1.033	1.085	0.986	**1.086**
-13.510	1.121	1.130	1.166	**1.170**	-13.550	1.140	1.129	1.095	**1.095**
-13.500	1.211	1.169	1.239	**1.178**	-13.540	1.118	1.130	1.038	**1.104**
-13.490	1.139	1.177	1.125	**1.187**	-13.530	1.157	1.097	1.097	**1.113**
-13.480	1.243	1.226	1.247	**1.195**	-13.520	1.101	1.088	1.116	**1.122**
-13.470	1.201	1.146	1.162	**1.204**	-13.510	1.057	1.101	1.134	**1.131**
-13.460	1.219	1.176	1.212	**1.212**	-13.500	1.104	1.042	1.098	**1.140**
-13.450	1.165	1.173	1.203	**1.221**	-13.490	1.107	1.171	1.187	**1.149**
-13.440	1.240	1.226	1.269	**1.229**	-13.480	1.202	1.243	1.205	**1.158**
-13.430	1.254	1.215	1.233	**1.238**	-13.470	1.117	1.072	1.165	**1.167**
-13.420	1.249	1.324	1.274	**1.246**	-13.460	1.209	1.146	1.191	**1.176**
-13.410	1.237	1.220	1.231	**1.255**	-13.450	1.288	1.229	1.177	**1.185**
-13.400	1.254	1.254	1.299	**1.263**	-13.440	1.218	1.173	1.191	**1.194**
-13.390	1.268	1.280	1.230	**1.272**	-13.430	1.208	1.174	1.200	**1.203**
-13.380	1.265	1.249	1.223	**1.280**	-13.420	1.269	1.248	1.185	**1.212**
-13.370	1.281	1.240	1.285	**1.289**	-13.410	1.147	1.194	1.165	**1.221**

-13.360	1.312	1.329	1.302	**1.297**	-13.400	1.243	1.260	1.322	**1.230**
-13.350	1.283	1.321	1.351	**1.306**	-13.390	1.211	1.225	1.189	**1.239**
-13.340	1.258	1.302	1.333	**1.314**	-13.380	1.175	1.193	1.272	**1.248**
-13.330	1.331	1.300	1.287	**1.323**	-13.370	1.178	1.214	1.243	**1.257**
-13.320	1.358	1.342	1.319	**1.331**	-13.360	1.266	1.183	1.254	**1.266**
-13.310	1.318	1.349	1.334	**1.340**	-13.350	1.282	1.233	1.255	**1.275**
-13.300	1.318	1.390	1.358	**1.348**	-13.340	1.283	1.273	1.222	**1.284**
-13.290	1.335	1.329	1.333	**1.357**	-13.330	1.268	1.330	1.335	**1.293**
-13.280	1.347	1.366	1.350	**1.365**	-13.320	1.249	1.309	1.332	**1.302**
-13.270	1.333	1.363	1.393	**1.373**	-13.310	1.274	1.271	1.286	**1.311**
-13.260	1.344	1.343	1.299	**1.382**	-13.300	1.295	1.377	1.325	**1.320**
-13.250	1.421	1.424	1.389	**1.390**	-13.290	1.374	1.296	1.300	**1.329**
-13.240	1.394	1.403	1.377	**1.398**	-13.280	1.329	1.262	1.312	**1.337**
-13.230	1.422	1.393	1.432	**1.406**	-13.270	1.290	1.298	1.314	**1.344**
-13.220	1.419	1.386	1.401	**1.414**	-13.260	1.260	1.321	1.350	**1.350**
-13.210	1.437	1.406	1.436	**1.422**	-13.250	1.345	1.424	1.371	**1.356**
-13.200	1.472	1.463	1.417	**1.430**	-13.240	1.298	1.341	1.344	**1.363**
-13.190	1.450	1.452	1.401	**1.436**	-13.230	1.414	1.437	1.374	**1.369**
-13.180	1.409	1.444	1.426	**1.442**	-13.220	1.376	1.438	1.356	**1.375**
-13.170	1.470	1.494	1.541	**1.448**	-13.210	1.350	1.392	1.437	**1.381**
-13.160	1.437	1.458	1.466	**1.454**	-13.200	1.388	1.351	1.351	**1.388**
-13.150	1.450	1.473	1.478	**1.459**	-13.190	1.325	1.410	1.430	**1.394**
-13.140	1.447	1.496	1.442	**1.465**	-13.180	1.417	1.439	1.407	**1.400**
-13.130	1.439	1.440	1.465	**1.471**	-13.170	1.452	1.463	1.403	**1.407**
-13.120	1.424	1.446	1.464	**1.476**	-13.160	1.381	1.411	1.478	**1.413**
-13.110	1.457	1.460	1.483	**1.480**	-13.150	1.427	1.525	1.462	**1.419**
-13.100	1.534	1.552	1.502	**1.485**	-13.140	1.400	1.442	1.379	**1.425**
-13.090	1.532	1.523	1.507	**1.489**	-13.130	1.435	1.360	1.456	**1.432**
-13.080	1.469	1.502	1.511	**1.494**	-13.120	1.471	1.435	1.425	**1.438**
-13.070	1.488	1.501	1.499	**1.498**	-13.110	1.401	1.435	1.404	**1.444**
-13.060	1.509	1.486	1.547	**1.502**	-13.100	1.504	1.426	1.486	**1.451**
-13.050	1.506	1.489	1.513	**1.506**	-13.090	1.480	1.515	1.460	**1.456**

-13.040	1.467	1.441	1.515	**1.509**	-13.080	1.412	1.467	1.512	**1.462**
-13.030	1.495	1.493	1.543	**1.512**	-13.070	1.515	1.493	1.509	**1.468**
-13.020	1.545	1.545	1.512	**1.515**	-13.060	1.520	1.521	1.517	**1.474**
-13.010	1.513	1.526	1.513	**1.518**	-13.050	1.532	1.516	1.517	**1.479**
-13.000	1.481	1.485	1.521	**1.521**	-13.040	1.493	1.484	1.487	**1.483**
-12.990	1.510	1.512	1.574	**1.524**	-13.030	1.450	1.532	1.459	**1.488**
-12.980	1.540	1.543	1.538	**1.527**	-13.020	1.486	1.534	1.534	**1.493**
-12.970	1.589	1.526	1.528	**1.529**	-13.010	1.566	1.487	1.535	**1.498**
-12.960	1.532	1.516	1.518	**1.531**	-13.000	1.447	1.438	1.515	**1.502**
-12.950	1.551	1.541	1.548	**1.532**	-12.990	1.504	1.470	1.501	**1.506**
-12.940	1.510	1.536	1.574	**1.533**	-12.980	1.592	1.542	1.605	**1.510**
-12.930	1.554	1.586	1.536	**1.534**	-12.970	1.501	1.545	1.564	**1.514**
-12.920	1.527	1.567	1.597	**1.535**	-12.960	1.573	1.520	1.538	**1.517**
-12.910	1.553	1.534	1.514	**1.536**	-12.950	1.471	1.534	1.544	**1.521**
-12.900	1.536	1.570	1.517	**1.538**	-12.940	1.545	1.589	1.535	**1.524**
-12.890	1.588	1.583	1.540	**1.539**	-12.930	1.445	1.551	1.512	**1.527**
-12.880	1.553	1.549	1.561	**1.540**	-12.920	1.543	1.552	1.457	**1.530**
-12.870	1.546	1.536	1.585	**1.541**	-12.910	1.557	1.526	1.514	**1.533**
-12.860	1.580	1.535	1.568	**1.542**	-12.900	1.446	1.551	1.528	**1.536**
-12.850	1.534	1.596	1.525	**1.543**	-12.890	1.511	1.562	1.602	**1.538**
-12.840	1.502	1.553	1.527	**1.544**	-12.880	1.518	1.532	1.475	**1.540**
-12.830	1.498	1.505	1.518	**1.545**	-12.870	1.574	1.569	1.551	**1.542**
-12.820	1.531	1.556	1.554	**1.546**	-12.860	1.590	1.543	1.598	**1.544**
-12.810	1.527	1.537	1.491	**1.547**	-12.850	1.598	1.637	1.541	**1.546**
-12.800	1.586	1.553	1.574	**1.549**	-12.840	1.496	1.464	1.524	**1.547**
-12.790	1.514	1.523	1.550	**1.550**	-12.830	1.559	1.512	1.527	**1.549**
-12.780	1.541	1.480	1.517	**1.551**	-12.820	1.496	1.593	1.582	**1.551**
-12.770	1.517	1.508	1.572	**1.551**	-12.810	1.589	1.488	1.556	**1.553**
-12.760	1.536	1.599	1.536	**1.552**	-12.800	1.564	1.504	1.549	**1.554**
-12.750	1.479	1.522	1.546	**1.552**	-12.790	1.520	1.545	1.547	**1.556**
-12.740	1.549	1.542	1.546	**1.552**	-12.780	1.519	1.626	1.586	**1.557**
-12.730	1.618	1.554	1.567	**1.552**	-12.770	1.536	1.541	1.483	**1.558**

-12.720	1.515	1.500	1.532	**1.552**	-12.760	1.563	1.544	1.495	**1.559**
-12.710	1.534	1.490	1.513	**1.552**	-12.750	1.525	1.499	1.595	**1.560**
-12.700	1.562	1.531	1.525	**1.552**	-12.740	1.621	1.602	1.626	**1.560**
-12.690	1.568	1.540	1.551	**1.552**	-12.730	1.588	1.652	1.563	**1.560**
-12.680	1.516	1.456	1.478	**1.551**	-12.720	1.537	1.543	1.582	**1.559**
-12.670	1.576	1.586	1.576	**1.551**	-12.710	1.599	1.541	1.549	**1.559**
-12.660	1.552	1.540	1.596	**1.551**	-12.700	1.573	1.535	1.493	**1.559**
-12.650	1.542	1.538	1.580	**1.551**	-12.690	1.586	1.501	1.582	**1.559**
-12.640	1.540	1.468	1.508	**1.550**	-12.680	1.601	1.554	1.599	**1.558**
-12.630	1.555	1.504	1.568	**1.550**	-12.670	1.497	1.599	1.516	**1.558**
-12.620	1.581	1.513	1.536	**1.550**	-12.660	1.598	1.486	1.547	**1.558**
-12.610	1.578	1.562	1.531	**1.550**	-12.650	1.555	1.494	1.579	**1.557**
-12.600	1.583	1.571	1.560	**1.550**	-12.640	1.596	1.584	1.612	**1.557**
-12.590	1.579	1.522	1.572	**1.549**	-12.630	1.588	1.568	1.590	**1.557**
-12.580	1.542	1.531	1.521	**1.549**	-12.620	1.591	1.579	1.502	**1.556**
-12.570	1.532	1.556	1.542	**1.549**	-12.610	1.577	1.506	1.609	**1.556**
-12.560	1.517	1.577	1.577	**1.549**	-12.600	1.596	1.562	1.639	**1.555**
-12.550	1.557	1.607	1.558	**1.549**	-12.590	1.511	1.483	1.535	**1.555**
-12.540	1.538	1.570	1.528	**1.549**	-12.580	1.492	1.549	1.562	**1.554**
-12.530	1.515	1.564	1.569	**1.548**	-12.570	1.576	1.603	1.522	**1.554**
-12.520	1.524	1.537	1.555	**1.548**	-12.560	1.501	1.569	1.603	**1.553**
-12.510	1.615	1.602	1.558	**1.548**	-12.550	1.476	1.580	1.512	**1.553**
-12.500	1.502	1.553	1.526	**1.548**	-12.540	1.519	1.581	1.559	**1.553**
-12.490	1.560	1.582	1.549	**1.548**	-12.530	1.594	1.543	1.499	**1.553**
-12.480	1.545	1.505	1.580	**1.548**	-12.520	1.521	1.531	1.576	**1.553**
-12.470	1.572	1.537	1.497	**1.548**	-12.510	1.477	1.526	1.518	**1.553**
-12.460	1.580	1.571	1.547	**1.548**	-12.500	1.483	1.518	1.517	**1.553**
-12.450	1.528	1.546	1.572	**1.548**	-12.490	1.552	1.579	1.633	**1.553**
-12.440	1.547	1.587	1.530	**1.547**	-12.480	1.487	1.575	1.583	**1.554**
-12.430	1.583	1.603	1.536	**1.547**	-12.470	1.520	1.581	1.572	**1.554**
-12.420	1.565	1.519	1.559	**1.547**	-12.460	1.605	1.634	1.548	**1.554**
-12.410	1.555	1.496	1.507	**1.548**	-12.450	1.507	1.569	1.570	**1.554**

-12.400	1.560	1.512	1.516	**1.548**	-12.440	1.608	1.585	1.564	**1.554**
-12.390	1.600	1.589	1.601	**1.549**	-12.430	1.546	1.593	1.494	**1.554**
-12.380	1.522	1.563	1.583	**1.549**	-12.420	1.582	1.594	1.558	**1.554**
-12.370	1.571	1.496	1.545	**1.550**	-12.410	1.571	1.528	1.595	**1.553**
-12.360	1.503	1.552	1.573	**1.550**	-12.400	1.573	1.523	1.493	**1.553**
-12.350	1.479	1.526	1.486	**1.551**	-12.390	1.577	1.625	1.574	**1.553**
-12.340	1.559	1.549	1.571	**1.551**	-12.380	1.520	1.527	1.585	**1.552**
-12.330	1.553	1.543	1.579	**1.552**	-12.370	1.602	1.549	1.573	**1.552**
-12.320	1.564	1.574	1.560	**1.552**	-12.360	1.596	1.512	1.601	**1.552**
-12.310	1.602	1.532	1.588	**1.553**	-12.350	1.494	1.563	1.487	**1.552**
-12.300	1.580	1.510	1.535	**1.553**	-12.340	1.614	1.606	1.617	**1.552**
-12.290	1.577	1.580	1.549	**1.554**	-12.330	1.577	1.613	1.557	**1.552**
-12.280	1.558	1.586	1.512	**1.555**	-12.320	1.493	1.593	1.560	**1.551**
-12.270	1.533	1.506	1.580	**1.555**	-12.310	1.507	1.541	1.581	**1.551**
-12.260	1.577	1.534	1.540	**1.555**	-12.300	1.632	1.521	1.562	**1.550**
-12.250	1.577	1.514	1.545	**1.555**	-12.290	1.493	1.547	1.525	**1.550**
-12.240	1.531	1.547	1.550	**1.555**	-12.280	1.529	1.431	1.510	**1.550**
-12.230	1.525	1.534	1.532	**1.555**	-12.270	1.587	1.600	1.560	**1.549**
-12.220	1.543	1.541	1.541	**1.556**	-12.260	1.526	1.532	1.567	**1.549**
-12.210	1.528	1.491	1.558	**1.556**	-12.250	1.558	1.627	1.655	**1.549**
-12.200	1.560	1.532	1.489	**1.556**	-12.240	1.521	1.519	1.589	**1.549**
-12.190	1.573	1.553	1.513	**1.556**	-12.230	1.516	1.526	1.562	**1.549**
-12.180	1.565	1.550	1.524	**1.555**	-12.220	1.552	1.556	1.604	**1.549**
-12.170	1.563	1.587	1.518	**1.555**	-12.210	1.483	1.511	1.535	**1.549**
-12.160	1.593	1.528	1.572	**1.555**	-12.200	1.435	1.540	1.509	**1.549**
-12.150	1.584	1.525	1.556	**1.555**	-12.190	1.519	1.577	1.596	**1.549**
-12.140	1.552	1.561	1.524	**1.555**	-12.180	1.505	1.539	1.555	**1.549**
-12.130	1.565	1.528	1.587	**1.554**	-12.170	1.548	1.576	1.546	**1.550**
-12.120	1.610	1.581	1.557	**1.554**	-12.160	1.550	1.497	1.585	**1.550**
-12.110	1.595	1.588	1.552	**1.554**	-12.150	1.549	1.550	1.546	**1.550**
-12.100	1.498	1.534	1.550	**1.553**	-12.140	1.573	1.531	1.560	**1.550**
-12.090	1.580	1.598	1.567	**1.553**	-12.130	1.559	1.558	1.553	**1.551**

-12.080	1.547	1.609	1.540	**1.552**	-12.120	1.590	1.609	1.603	**1.551**
-12.070	1.509	1.533	1.565	**1.552**	-12.110	1.533	1.577	1.502	**1.551**
-12.060	1.569	1.510	1.513	**1.552**	-12.100	1.572	1.498	1.535	**1.551**
-12.050	1.594	1.585	1.525	**1.551**	-12.090	1.577	1.617	1.579	**1.551**
-12.040	1.551	1.601	1.587	**1.551**	-12.080	1.544	1.513	1.443	**1.551**
-12.030	1.594	1.529	1.551	**1.550**	-12.070	1.575	1.644	1.536	**1.551**
-12.020	1.554	1.593	1.599	**1.550**	-12.060	1.520	1.589	1.530	**1.551**
-12.010	1.591	1.539	1.546	**1.551**	-12.050	1.576	1.554	1.637	**1.552**
-12.000	1.492	1.542	1.516	**1.551**	-12.040	1.572	1.598	1.597	**1.552**
-11.990	1.579	1.520	1.546	**1.551**	-12.030	1.537	1.577	1.518	**1.552**
-11.980	1.516	1.574	1.505	**1.552**	-12.020	1.587	1.579	1.572	**1.552**
-11.970	1.527	1.554	1.566	**1.553**	-12.010	1.537	1.589	1.547	**1.552**
-11.960	1.508	1.534	1.572	**1.553**	-12.000	1.576	1.547	1.543	**1.552**
-11.950	1.557	1.586	1.550	**1.554**	-11.990	1.530	1.539	1.573	**1.552**
-11.940	1.609	1.602	1.552	**1.554**	-11.980	1.571	1.588	1.622	**1.552**
-11.930	1.528	1.541	1.535	**1.555**	-11.970	1.529	1.561	1.571	**1.551**
-11.920	1.566	1.574	1.580	**1.555**	-11.960	1.468	1.557	1.548	**1.551**
-11.910	1.566	1.630	1.562	**1.555**	-11.950	1.522	1.543	1.536	**1.551**
-11.900	1.573	1.556	1.547	**1.556**	-11.940	1.609	1.500	1.559	**1.551**
-11.890	1.557	1.573	1.560	**1.556**	-11.930	1.618	1.625	1.587	**1.551**
-11.880	1.624	1.568	1.580	**1.556**	-11.920	1.541	1.570	1.498	**1.551**
-11.870	1.594	1.581	1.530	**1.556**	-11.910	1.565	1.589	1.533	**1.551**
-11.860	1.586	1.534	1.569	**1.556**	-11.900	1.551	1.542	1.525	**1.551**
-11.850	1.516	1.516	1.518	**1.557**	-11.890	1.444	1.555	1.486	**1.551**
-11.840	1.560	1.610	1.601	**1.557**	-11.880	1.517	1.491	1.509	**1.551**
-11.830	1.534	1.598	1.568	**1.557**	-11.870	1.487	1.533	1.558	**1.551**
-11.820	1.525	1.573	1.525	**1.557**	-11.860	1.558	1.484	1.503	**1.551**
-11.810	1.609	1.595	1.578	**1.557**	-11.850	1.548	1.571	1.513	**1.551**
-11.800	1.539	1.585	1.530	**1.557**	-11.840	1.592	1.540	1.513	**1.551**
-11.790	1.603	1.573	1.564	**1.557**	-11.830	1.520	1.596	1.516	**1.551**
-11.780	1.550	1.580	1.541	**1.557**	-11.820	1.534	1.641	1.595	**1.551**
-11.770	1.539	1.548	1.593	**1.557**	-11.810	1.538	1.481	1.510	**1.551**

-11.760	1.571	1.564	1.515	**1.557**	-11.800	1.581	1.523	1.630	**1.552**
-11.750	1.559	1.543	1.550	**1.557**	-11.790	1.505	1.585	1.574	**1.552**
-11.740	1.519	1.566	1.580	**1.557**	-11.780	1.593	1.586	1.675	**1.553**
-11.730	1.535	1.578	1.530	**1.557**	-11.770	1.428	1.486	1.522	**1.553**
-11.720	1.541	1.586	1.574	**1.557**	-11.760	1.535	1.605	1.615	**1.553**
-11.710	1.518	1.569	1.567	**1.557**	-11.750	1.638	1.544	1.550	**1.553**
-11.700	1.530	1.570	1.525	**1.556**	-11.740	1.567	1.649	1.554	**1.553**
-11.690	1.620	1.556	1.585	**1.556**	-11.730	1.562	1.525	1.560	**1.553**
-11.680	1.537	1.534	1.557	**1.556**	-11.720	1.567	1.549	1.511	**1.553**
-11.670	1.521	1.557	1.546	**1.556**	-11.710	1.553	1.570	1.643	**1.553**
-11.660	1.578	1.618	1.549	**1.556**	-11.700	1.504	1.541	1.600	**1.553**
-11.650	1.493	1.566	1.548	**1.556**	-11.690	1.566	1.556	1.612	**1.553**
-11.640	1.546	1.598	1.572	**1.555**	-11.680	1.504	1.601	1.569	**1.553**
-11.630	1.601	1.559	1.531	**1.555**	-11.670	1.516	1.432	1.451	**1.553**
-11.620	1.505	1.555	1.512	**1.555**	-11.660	1.549	1.625	1.647	**1.553**
-11.610	1.543	1.520	1.572	**1.555**	-11.650	1.559	1.615	1.534	**1.553**
-11.600	1.551	1.547	1.561	**1.555**	-11.640	1.505	1.591	1.572	**1.553**
-11.590	1.572	1.580	1.520	**1.555**	-11.630	1.522	1.543	1.508	**1.553**
-11.580	1.587	1.540	1.526	**1.555**	-11.620	1.563	1.563	1.544	**1.553**
-11.570	1.569	1.564	1.561	**1.556**	-11.610	1.532	1.567	1.470	**1.553**
-11.560	1.538	1.594	1.546	**1.556**	-11.600	1.568	1.550	1.545	**1.553**
-11.550	1.511	1.506	1.478	**1.556**	-11.590	1.559	1.524	1.593	**1.553**
-11.540	1.518	1.589	1.540	**1.556**	-11.580	1.543	1.518	1.494	**1.553**
-11.530	1.546	1.534	1.502	**1.556**	-11.570	1.612	1.592	1.620	**1.553**
-11.520	1.538	1.557	1.569	**1.555**	-11.560	1.621	1.579	1.525	**1.554**
-11.510	1.556	1.532	1.586	**1.555**	-11.550	1.534	1.513	1.554	**1.554**
-11.500	1.548	1.526	1.602	**1.555**	-11.540	1.629	1.567	1.628	**1.554**
-11.490	1.595	1.550	1.623	**1.555**	-11.530	1.514	1.617	1.570	**1.554**
-11.480	1.584	1.559	1.529	**1.555**	-11.520	1.607	1.629	1.558	**1.554**
-11.470	1.594	1.550	1.557	**1.555**	-11.510	1.619	1.586	1.581	**1.554**
-11.460	1.623	1.657	1.583	**1.554**	-11.500	1.566	1.546	1.502	**1.554**
-11.450	1.567	1.556	1.510	**1.554**	-11.490	1.587	1.611	1.540	**1.554**

-11.440	1.513	1.506	1.568	**1.553**	-11.480	1.601	1.581	1.582	**1.554**
-11.430	1.537	1.540	1.524	**1.553**	-11.470	1.510	1.543	1.577	**1.554**
-11.420	1.544	1.595	1.556	**1.553**	-11.460	1.541	1.549	1.520	**1.554**
-11.410	1.567	1.610	1.535	**1.553**	-11.450	1.628	1.582	1.531	**1.554**
-11.400	1.516	1.584	1.580	**1.553**	-11.440	1.566	1.493	1.479	**1.553**
-11.390	1.602	1.544	1.547	**1.553**	-11.430	1.559	1.588	1.558	**1.553**
-11.380	1.542	1.536	1.552	**1.553**	-11.420	1.557	1.540	1.550	**1.553**
-11.370	1.527	1.586	1.567	**1.553**	-11.410	1.563	1.514	1.581	**1.552**
-11.360	1.549	1.523	1.515	**1.554**	-11.400	1.632	1.552	1.538	**1.552**
-11.350	1.562	1.512	1.543	**1.554**	-11.390	1.596	1.510	1.525	**1.551**
-11.340	1.534	1.555	1.585	**1.554**	-11.380	1.570	1.533	1.510	**1.551**
-11.330	1.542	1.530	1.589	**1.555**	-11.370	1.499	1.549	1.541	**1.550**
-11.320	1.532	1.474	1.527	**1.555**	-11.360	1.633	1.549	1.555	**1.550**
-11.310	1.597	1.590	1.590	**1.556**	-11.350	1.509	1.572	1.561	**1.549**
-11.300	1.521	1.572	1.523	**1.556**	-11.340	1.443	1.514	1.511	**1.549**
-11.290	1.575	1.586	1.559	**1.557**	-11.330	1.551	1.475	1.495	**1.549**
-11.280	1.555	1.566	1.543	**1.559**	-11.320	1.565	1.562	1.573	**1.549**
-11.270	1.577	1.633	1.621	**1.560**	-11.310	1.608	1.600	1.618	**1.550**
-11.260	1.591	1.568	1.574	**1.562**	-11.300	1.545	1.489	1.596	**1.550**
-11.250	1.583	1.548	1.572	**1.564**	-11.290	1.706	1.646	1.603	**1.550**
-11.240	1.523	1.548	1.551	**1.565**	-11.280	1.575	1.471	1.545	**1.550**
-11.230	1.605	1.558	1.599	**1.567**	-11.270	1.665	1.583	1.565	**1.551**
-11.220	1.575	1.578	1.606	**1.569**	-11.260	1.486	1.566	1.536	**1.551**
-11.210	1.557	1.618	1.554	**1.573**	-11.250	1.543	1.503	1.604	**1.552**
-11.200	1.601	1.629	1.621	**1.576**	-11.240	1.518	1.584	1.506	**1.552**
-11.190	1.579	1.556	1.584	**1.579**	-11.230	1.504	1.503	1.465	**1.553**
-11.180	1.600	1.587	1.623	**1.581**	-11.220	1.529	1.496	1.526	**1.555**
-11.170	1.619	1.662	1.619	**1.584**	-11.210	1.557	1.565	1.534	**1.556**
-11.160	1.588	1.565	1.565	**1.587**	-11.200	1.612	1.638	1.594	**1.559**
-11.150	1.602	1.654	1.610	**1.590**	-11.190	1.518	1.516	1.545	**1.561**
-11.140	1.555	1.539	1.526	**1.593**	-11.180	1.616	1.562	1.543	**1.564**
-11.130	1.626	1.591	1.600	**1.596**	-11.170	1.645	1.581	1.542	**1.567**

-11.120	1.587	1.578	1.613	**1.599**	-11.160	1.549	1.572	1.543	**1.571**
-11.110	1.576	1.592	1.615	**1.602**	-11.150	1.605	1.538	1.534	**1.575**
-11.100	1.637	1.599	1.577	**1.605**	-11.140	1.535	1.561	1.474	**1.579**
-11.090	1.625	1.603	1.587	**1.608**	-11.130	1.571	1.628	1.613	**1.583**
-11.080	1.575	1.562	1.580	**1.611**	-11.120	1.602	1.562	1.620	**1.587**
-11.070	1.577	1.622	1.629	**1.614**	-11.110	1.595	1.623	1.625	**1.591**
-11.060	1.649	1.644	1.592	**1.617**	-11.100	1.586	1.664	1.568	**1.595**
-11.050	1.601	1.662	1.599	**1.620**	-11.090	1.632	1.584	1.658	**1.599**
-11.040	1.638	1.612	1.607	**1.623**	-11.080	1.589	1.556	1.540	**1.603**
-11.030	1.618	1.603	1.628	**1.625**	-11.070	1.564	1.653	1.667	**1.607**
-11.020	1.637	1.672	1.667	**1.628**	-11.060	1.642	1.672	1.644	**1.611**
-11.010	1.633	1.640	1.606	**1.631**	-11.050	1.590	1.677	1.576	**1.614**
-11.000	1.687	1.646	1.634	**1.634**	-11.040	1.623	1.606	1.621	**1.618**
-10.990	1.654	1.589	1.640	**1.638**	-11.030	1.672	1.654	1.607	**1.622**
-10.980	1.657	1.640	1.607	**1.641**	-11.020	1.588	1.618	1.650	**1.626**
-10.970	1.684	1.682	1.640	**1.645**	-11.010	1.636	1.585	1.680	**1.630**
-10.960	1.659	1.673	1.662	**1.649**	-11.000	1.568	1.626	1.613	**1.634**
-10.950	1.664	1.682	1.645	**1.652**	-10.990	1.631	1.582	1.572	**1.638**
-10.940	1.656	1.677	1.661	**1.656**	-10.980	1.635	1.640	1.675	**1.642**
-10.930	1.688	1.676	1.690	**1.660**	-10.970	1.655	1.608	1.619	**1.646**
-10.920	1.685	1.668	1.676	**1.665**	-10.960	1.692	1.684	1.750	**1.650**
-10.910	1.692	1.699	1.630	**1.670**	-10.950	1.673	1.624	1.590	**1.654**
-10.900	1.662	1.680	1.665	**1.676**	-10.940	1.693	1.721	1.614	**1.658**
-10.890	1.639	1.678	1.700	**1.681**	-10.930	1.656	1.674	1.586	**1.662**
-10.880	1.674	1.728	1.690	**1.686**	-10.920	1.647	1.671	1.659	**1.665**
-10.870	1.687	1.647	1.660	**1.691**	-10.910	1.665	1.692	1.660	**1.669**
-10.860	1.674	1.675	1.728	**1.696**	-10.900	1.692	1.666	1.709	**1.673**
-10.850	1.701	1.655	1.679	**1.701**	-10.890	1.625	1.720	1.651	**1.677**
-10.840	1.665	1.706	1.653	**1.706**	-10.880	1.636	1.564	1.633	**1.681**
-10.830	1.735	1.725	1.691	**1.711**	-10.870	1.620	1.627	1.698	**1.685**
-10.820	1.675	1.683	1.684	**1.715**	-10.860	1.747	1.647	1.695	**1.689**
-10.810	1.703	1.715	1.767	**1.720**	-10.850	1.695	1.794	1.781	**1.693**

-10.800	1.763	1.735	1.703	**1.724**	-10.840	1.653	1.668	1.687	**1.698**
-10.790	1.794	1.741	1.766	**1.729**	-10.830	1.665	1.619	1.593	**1.702**
-10.780	1.726	1.737	1.732	**1.733**	-10.820	1.715	1.804	1.765	**1.706**
-10.770	1.713	1.733	1.687	**1.738**	-10.810	1.684	1.756	1.791	**1.710**
-10.760	1.744	1.724	1.763	**1.742**	-10.800	1.798	1.751	1.713	**1.714**
-10.750	1.752	1.741	1.782	**1.747**	-10.790	1.706	1.716	1.707	**1.718**
-10.740	1.746	1.739	1.692	**1.751**	-10.780	1.655	1.719	1.658	**1.722**
-10.730	1.707	1.752	1.697	**1.756**	-10.770	1.690	1.675	1.720	**1.726**
-10.720	1.803	1.764	1.796	**1.760**	-10.760	1.773	1.764	1.729	**1.730**
-10.710	1.791	1.749	1.725	**1.765**	-10.750	1.651	1.725	1.687	**1.734**
-10.700	1.751	1.720	1.743	**1.769**	-10.740	1.773	1.739	1.748	**1.739**
-10.690	1.766	1.740	1.771	**1.774**	-10.730	1.821	1.748	1.804	**1.743**
-10.680	1.810	1.740	1.779	**1.778**	-10.720	1.741	1.775	1.766	**1.747**
-10.670	1.752	1.751	1.777	**1.783**	-10.710	1.760	1.812	1.744	**1.752**
-10.660	1.792	1.736	1.810	**1.787**	-10.700	1.775	1.789	1.812	**1.756**
-10.650	1.816	1.802	1.813	**1.791**	-10.690	1.868	1.765	1.852	**1.760**
-10.640	1.753	1.778	1.778	**1.794**	-10.680	1.763	1.826	1.731	**1.765**
-10.630	1.831	1.811	1.774	**1.798**	-10.670	1.705	1.800	1.760	**1.769**
-10.620	1.817	1.813	1.830	**1.801**	-10.660	1.759	1.744	1.686	**1.773**
-10.610	1.824	1.830	1.783	**1.805**	-10.650	1.781	1.770	1.714	**1.777**
-10.600	1.781	1.800	1.836	**1.808**	-10.640	1.692	1.702	1.759	**1.780**
-10.590	1.884	1.853	1.817	**1.812**	-10.630	1.819	1.812	1.845	**1.784**
-10.580	1.832	1.835	1.834	**1.815**	-10.620	1.749	1.807	1.843	**1.788**
-10.570	1.782	1.776	1.750	**1.819**	-10.610	1.841	1.778	1.861	**1.791**
-10.560	1.832	1.829	1.860	**1.822**	-10.600	1.812	1.719	1.783	**1.795**
-10.550	1.797	1.795	1.845	**1.826**	-10.590	1.829	1.779	1.791	**1.798**
-10.540	1.829	1.803	1.788	**1.829**	-10.580	1.814	1.859	1.808	**1.802**
-10.530	1.819	1.792	1.809	**1.833**	-10.570	1.765	1.853	1.818	**1.805**
-10.520	1.819	1.814	1.817	**1.836**	-10.560	1.740	1.795	1.696	**1.809**
-10.510	1.836	1.859	1.822	**1.839**	-10.550	1.747	1.820	1.799	**1.812**
-10.500	1.828	1.827	1.819	**1.841**	-10.540	1.797	1.778	1.809	**1.816**
-10.490	1.860	1.843	1.849	**1.844**	-10.530	1.791	1.836	1.888	**1.820**

-10.480	1.873	1.840	1.860	**1.845**	-10.520	1.768	1.845	1.873	**1.823**
-10.470	1.844	1.869	1.815	**1.846**	-10.510	1.880	1.808	1.859	**1.827**
-10.460	1.893	1.843	1.833	**1.848**	-10.500	1.768	1.855	1.822	**1.830**
-10.450	1.888	1.820	1.879	**1.849**	-10.490	1.836	1.841	1.843	**1.834**
-10.440	1.888	1.852	1.815	**1.850**	-10.480	1.796	1.834	1.898	**1.837**
-10.430	1.861	1.874	1.822	**1.851**	-10.470	1.841	1.851	1.771	**1.840**
-10.420	1.845	1.884	1.866	**1.853**	-10.460	1.873	1.905	1.821	**1.842**
-10.410	1.784	1.814	1.857	**1.854**	-10.450	1.778	1.811	1.852	**1.844**
-10.400	1.848	1.879	1.875	**1.855**	-10.440	1.892	1.931	1.933	**1.846**
-10.390	1.889	1.870	1.821	**1.856**	-10.430	1.873	1.814	1.892	**1.848**
-10.380	1.916	1.914	1.879	**1.857**	-10.420	1.869	1.841	1.896	**1.850**
-10.370	1.862	1.821	1.863	**1.858**	-10.410	1.786	1.816	1.780	**1.852**
-10.360	1.830	1.783	1.832	**1.859**	-10.400	1.773	1.839	1.838	**1.853**
-10.350	1.844	1.812	1.821	**1.860**	-10.390	1.905	1.884	1.877	**1.855**
-10.340	1.812	1.801	1.833	**1.860**	-10.380	1.841	1.835	1.858	**1.855**
-10.330	1.885	1.879	1.852	**1.860**	-10.370	1.899	1.874	1.933	**1.856**
-10.320	1.844	1.843	1.821	**1.860**	-10.360	1.860	1.832	1.798	**1.856**
-10.310	1.834	1.810	1.817	**1.860**	-10.350	1.864	1.900	1.872	**1.856**
-10.300	1.804	1.858	1.863	**1.860**	-10.340	1.839	1.827	1.848	**1.856**
-10.290	1.834	1.869	1.838	**1.860**	-10.330	1.877	1.851	1.831	**1.857**
-10.280	1.839	1.843	1.855	**1.860**	-10.320	1.883	1.780	1.796	**1.857**
-10.270	1.818	1.870	1.846	**1.860**	-10.310	1.866	1.933	1.835	**1.857**
-10.260	1.870	1.856	1.819	**1.860**	-10.300	1.803	1.787	1.817	**1.857**
-10.250	1.873	1.855	1.883	**1.861**	-10.290	1.831	1.864	1.765	**1.857**
-10.240	1.868	1.868	1.837	**1.861**	-10.280	1.908	1.856	1.814	**1.857**
-10.230	1.838	1.798	1.848	**1.861**	-10.270	1.885	1.853	1.891	**1.857**
-10.220	1.919	1.862	1.850	**1.861**	-10.260	1.866	1.797	1.866	**1.857**
-10.210	1.888	1.863	1.833	**1.861**	-10.250	1.852	1.837	1.874	**1.857**
-10.200	1.856	1.802	1.843	**1.862**	-10.240	1.754	1.834	1.839	**1.857**
-10.190	1.867	1.916	1.839	**1.862**	-10.230	1.919	1.853	1.824	**1.857**
-10.180	1.898	1.833	1.894	**1.862**	-10.220	1.856	1.865	1.928	**1.857**
-10.170	1.912	1.844	1.873	**1.862**	-10.210	1.905	1.859	1.871	**1.857**

-10.160	1.846	1.841	1.875	**1.862**	-10.200	1.853	1.849	1.801	**1.857**
-10.150	1.871	1.845	1.801	**1.862**	-10.190	1.835	1.846	1.819	**1.857**
-10.140	1.873	1.859	1.917	**1.862**	-10.180	1.751	1.840	1.852	**1.857**
-10.130	1.845	1.914	1.864	**1.862**	-10.170	1.852	1.831	1.925	**1.857**
-10.120	1.899	1.827	1.840	**1.862**	-10.160	1.936	1.853	1.858	**1.857**
-10.110	1.816	1.834	1.869	**1.862**	-10.150	1.759	1.865	1.800	**1.857**
-10.100	1.848	1.867	1.812	**1.862**	-10.140	1.872	1.885	1.868	**1.857**
-10.090	1.856	1.883	1.827	**1.862**	-10.130	1.852	1.850	1.892	**1.857**
-10.080	1.871	1.857	1.827	**1.862**	-10.120	1.742	1.811	1.789	**1.856**
-10.070	1.898	1.871	1.828	**1.862**	-10.110	1.893	1.820	1.787	**1.856**
-10.060	1.833	1.871	1.870	**1.862**	-10.100	1.877	1.903	1.874	**1.856**
-10.050	1.875	1.846	1.882	**1.863**	-10.090	1.937	1.900	1.868	**1.856**
-10.040	1.845	1.884	1.811	**1.863**	-10.080	1.888	1.909	1.820	**1.856**
-10.030	1.825	1.853	1.869	**1.863**	-10.070	1.773	1.851	1.796	**1.856**
-10.020	1.839	1.819	1.784	**1.862**	-10.060	1.845	1.883	1.884	**1.856**
-10.010	1.868	1.861	1.927	**1.862**	-10.050	1.856	1.936	1.833	**1.856**
-10.000	1.883	1.872	1.872	**1.863**	-10.040	1.846	1.850	1.873	**1.856**
-9.990	1.861	1.885	1.856	**1.863**	-10.030	1.852	1.831	1.873	**1.857**
-9.980	1.833	1.866	1.861	**1.863**	-10.020	1.821	1.809	1.849	**1.857**
-9.970	1.819	1.881	1.843	**1.863**	-10.010	1.839	1.794	1.848	**1.857**
-9.960	1.873	1.928	1.890	**1.863**	-10.000	1.887	1.888	1.915	**1.857**
-9.950	1.824	1.834	1.843	**1.863**	-9.990	1.869	1.854	1.801	**1.857**
-9.940	1.870	1.888	1.836	**1.863**	-9.980	1.881	1.861	1.882	**1.857**
-9.930	1.872	1.875	1.929	**1.863**	-9.970	1.800	1.887	1.863	**1.857**
-9.920	1.855	1.913	1.927	**1.864**	-9.960	1.840	1.763	1.863	**1.857**
-9.910	1.834	1.894	1.891	**1.864**	-9.950	1.821	1.807	1.782	**1.857**
-9.900	1.841	1.876	1.817	**1.864**	-9.940	1.926	1.908	1.916	**1.857**
-9.890	1.870	1.865	1.910	**1.863**	-9.930	1.854	1.886	1.843	**1.857**
-9.880	1.835	1.857	1.870	**1.864**	-9.920	1.903	1.901	1.888	**1.857**
-9.870	1.849	1.907	1.850	**1.864**	-9.910	1.864	1.880	1.940	**1.857**
-9.860	1.877	1.847	1.838	**1.864**	-9.900	1.881	1.792	1.864	**1.857**
-9.850	1.851	1.810	1.873	**1.864**	-9.890	1.859	1.843	1.810	**1.857**

-9.840	1.859	1.882	1.841	**1.864**	-9.880	1.870	1.822	1.835	**1.856**
-9.830	1.851	1.906	1.842	**1.864**	-9.870	1.858	1.905	1.927	**1.856**
-9.820	1.884	1.842	1.832	**1.864**	-9.860	1.765	1.834	1.815	**1.856**
-9.810	1.889	1.910	1.906	**1.864**	-9.850	1.782	1.866	1.862	**1.856**
-9.800	1.892	1.879	1.846	**1.864**	-9.840	1.820	1.810	1.840	**1.856**
-9.790	1.848	1.864	1.800	**1.864**	-9.830	1.772	1.846	1.864	**1.857**
-9.780	1.851	1.852	1.840	**1.864**	-9.820	1.915	1.907	1.840	**1.857**
-9.770	1.880	1.833	1.893	**1.864**	-9.810	1.820	1.915	1.902	**1.857**
-9.760	1.888	1.844	1.835	**1.864**	-9.800	1.826	1.823	1.844	**1.857**
-9.750	1.851	1.907	1.891	**1.863**	-9.790	1.858	1.882	1.879	**1.857**
-9.740	1.896	1.837	1.857	**1.863**	-9.780	1.785	1.824	1.890	**1.857**
-9.730	1.841	1.825	1.885	**1.863**	-9.770	1.832	1.816	1.853	**1.857**
-9.720	1.817	1.862	1.893	**1.863**	-9.760	1.905	1.825	1.875	**1.857**
-9.710	1.861	1.896	1.819	**1.863**	-9.750	1.821	1.882	1.851	**1.857**
-9.700	1.846	1.894	1.888	**1.863**	-9.740	1.856	1.887	1.829	**1.857**
-9.690	1.892	1.874	1.869	**1.864**	-9.730	1.845	1.822	1.883	**1.858**
-9.680	1.814	1.859	1.840	**1.864**	-9.720	1.886	1.860	1.851	**1.858**
-9.670	1.892	1.832	1.893	**1.864**	-9.710	1.888	1.867	1.920	**1.858**
-9.660	1.883	1.831	1.870	**1.864**	-9.700	1.777	1.815	1.882	**1.858**
-9.650	1.831	1.813	1.818	**1.865**	-9.690	1.791	1.768	1.761	**1.858**
-9.640	1.829	1.853	1.835	**1.865**	-9.680	1.785	1.862	1.864	**1.858**
-9.630	1.874	1.864	1.868	**1.866**	-9.670	1.826	1.850	1.889	**1.859**
-9.620	1.856	1.822	1.824	**1.866**	-9.660	1.845	1.858	1.817	**1.859**
-9.610	1.862	1.855	1.892	**1.866**	-9.650	1.805	1.831	1.877	**1.859**
-9.600	1.905	1.878	1.830	**1.866**	-9.640	1.872	1.814	1.906	**1.860**
-9.590	1.849	1.865	1.876	**1.866**	-9.630	1.790	1.860	1.768	**1.860**
-9.580	1.868	1.900	1.871	**1.866**	-9.620	1.854	1.908	1.849	**1.860**
-9.570	1.888	1.962	1.910	**1.866**	-9.610	1.828	1.937	1.869	**1.861**
-9.560	1.847	1.854	1.848	**1.866**	-9.600	1.894	1.947	1.882	**1.861**
-9.550	1.871	1.903	1.920	**1.866**	-9.590	1.856	1.829	1.791	**1.861**
-9.540	1.835	1.845	1.836	**1.866**	-9.580	1.801	1.888	1.833	**1.862**
-9.530	1.926	1.921	1.876	**1.866**	-9.570	1.827	1.857	1.824	**1.862**

-9.520	1.856	1.828	1.784	**1.866**	-9.560	1.863	1.956	1.856	**1.862**
-9.510	1.880	1.899	1.903	**1.866**	-9.550	1.890	1.899	1.810	**1.862**
-9.500	1.869	1.879	1.913	**1.866**	-9.540	1.919	1.820	1.852	**1.863**
-9.490	1.844	1.904	1.886	**1.865**	-9.530	1.895	1.860	1.797	**1.863**
-9.480	1.838	1.827	1.806	**1.865**	-9.520	1.849	1.779	1.817	**1.863**
-9.470	1.870	1.859	1.835	**1.864**	-9.510	1.827	1.837	1.818	**1.863**
-9.460	1.911	1.868	1.888	**1.864**	-9.500	1.875	1.889	1.938	**1.863**
-9.450	1.841	1.858	1.893	**1.864**	-9.490	1.910	1.873	1.862	**1.863**
-9.440	1.867	1.867	1.817	**1.863**	-9.480	1.823	1.888	1.857	**1.864**
-9.430	1.871	1.868	1.907	**1.863**	-9.470	1.897	1.890	1.858	**1.864**
-9.420	1.881	1.943	1.888	**1.862**	-9.460	1.851	1.913	1.892	**1.864**
-9.410	1.853	1.808	1.833	**1.862**	-9.450	1.886	1.844	1.825	**1.864**
-9.400	1.867	1.841	1.876	**1.862**	-9.440	1.910	1.837	1.826	**1.864**
-9.390	1.879	1.868	1.863	**1.861**	-9.430	1.882	1.808	1.864	**1.864**
-9.380	1.861	1.876	1.862	**1.861**	-9.420	1.910	1.860	1.871	**1.864**
-9.370	1.843	1.876	1.829	**1.861**	-9.410	1.958	1.891	1.935	**1.864**
-9.360	1.811	1.888	1.850	**1.861**	-9.400	1.907	1.867	1.801	**1.864**
-9.350	1.840	1.904	1.907	**1.861**	-9.390	1.837	1.891	1.869	**1.864**
-9.340	1.839	1.859	1.818	**1.861**	-9.380	1.832	1.810	1.890	**1.863**
-9.330	1.811	1.846	1.814	**1.861**	-9.370	1.851	1.856	1.851	**1.863**
-9.320	1.866	1.895	1.905	**1.861**	-9.360	1.837	1.831	1.815	**1.863**
-9.310	1.862	1.864	1.865	**1.861**	-9.350	1.807	1.877	1.850	**1.863**
-9.300	1.853	1.876	1.828	**1.861**	-9.340	1.898	1.876	1.895	**1.863**
-9.290	1.863	1.847	1.862	**1.861**	-9.330	1.879	1.875	1.957	**1.863**
-9.280	1.813	1.853	1.830	**1.861**	-9.320	1.857	1.873	1.805	**1.863**
-9.270	1.812	1.795	1.848	**1.861**	-9.310	1.885	1.832	1.889	**1.862**
-9.260	1.902	1.874	1.858	**1.862**	-9.300	1.878	1.919	1.872	**1.862**
-9.250	1.811	1.866	1.864	**1.862**	-9.290	1.806	1.837	1.877	**1.862**
-9.240	1.872	1.862	1.871	**1.862**	-9.280	1.835	1.890	1.921	**1.862**
-9.230	1.821	1.852	1.793	**1.862**	-9.270	1.971	1.925	1.877	**1.861**
-9.220	1.848	1.883	1.866	**1.862**	-9.260	1.863	1.882	1.806	**1.861**
-9.210	1.844	1.839	1.844	**1.863**	-9.250	1.869	1.920	1.881	**1.861**

-9.200	1.882	1.900	1.937	**1.863**	-9.240	1.807	1.836	1.860	**1.861**
-9.190	1.807	1.807	1.813	**1.863**	-9.230	1.833	1.902	1.834	**1.860**
-9.180	1.842	1.913	1.877	**1.864**	-9.220	1.909	1.853	1.807	**1.860**
-9.170	1.880	1.865	1.864	**1.864**	-9.210	1.936	1.963	1.867	**1.860**
-9.160	1.830	1.877	1.812	**1.864**	-9.200	1.862	1.893	1.848	**1.860**
-9.150	1.808	1.819	1.842	**1.864**	-9.190	1.962	1.892	1.869	**1.860**
-9.140	1.851	1.827	1.792	**1.864**	-9.180	1.944	1.836	1.879	**1.860**
-9.130	1.854	1.889	1.841	**1.864**	-9.170	1.857	1.835	1.858	**1.860**
-9.120	1.935	1.910	1.894	**1.864**	-9.160	1.923	1.910	1.891	**1.859**
-9.110	1.855	1.861	1.883	**1.864**	-9.150	1.862	1.792	1.885	**1.859**
-9.100	1.853	1.895	1.885	**1.864**	-9.140	1.894	1.901	1.829	**1.859**
-9.090	1.823	1.841	1.869	**1.864**	-9.130	1.899	1.863	1.808	**1.859**
-9.080	1.852	1.869	1.849	**1.864**	-9.120	1.802	1.862	1.766	**1.859**
-9.070	1.845	1.893	1.867	**1.864**	-9.110	1.854	1.857	1.902	**1.859**
-9.060	1.819	1.861	1.839	**1.864**	-9.100	1.851	1.851	1.850	**1.859**
-9.050	1.832	1.849	1.858	**1.864**	-9.090	1.907	1.952	1.900	**1.859**
-9.040	1.866	1.869	1.829	**1.864**	-9.080	1.916	1.906	1.829	**1.859**
-9.030	1.866	1.865	1.898	**1.864**	-9.070	1.890	1.912	1.907	**1.860**
-9.020	1.924	1.915	1.882	**1.865**	-9.060	1.930	1.911	1.917	**1.860**
-9.010	1.885	1.857	1.860	**1.865**	-9.050	1.836	1.815	1.902	**1.860**
-9.000	1.864	1.861	1.879	**1.865**	-9.040	1.860	1.899	1.921	**1.860**
-8.990	1.894	1.885	1.912	**1.865**	-9.030	1.874	1.882	1.796	**1.860**
-8.980	1.883	1.822	1.866	**1.866**	-9.020	1.947	1.870	1.852	**1.860**
-8.970	1.851	1.913	1.901	**1.866**	-9.010	1.902	1.862	1.935	**1.860**
-8.960	1.797	1.831	1.824	**1.866**	-9.000	1.867	1.805	1.838	**1.860**
-8.950	1.873	1.876	1.896	**1.866**	-8.990	1.853	1.788	1.830	**1.860**
-8.940	1.913	1.871	1.880	**1.866**	-8.980	1.947	1.902	1.919	**1.860**
-8.930	1.806	1.846	1.862	**1.867**	-8.970	1.892	1.833	1.888	**1.860**
-8.920	1.895	1.896	1.845	**1.867**	-8.960	1.802	1.874	1.843	**1.860**
-8.910	1.799	1.853	1.864	**1.867**	-8.950	1.856	1.838	1.892	**1.860**
-8.900	1.842	1.885	1.857	**1.867**	-8.940	1.834	1.908	1.890	**1.860**
-8.890	1.834	1.893	1.855	**1.867**	-8.930	1.873	1.887	1.941	**1.860**

-8.880	1.894	1.879	1.822	**1.867**	-8.920	1.826	1.783	1.835	**1.860**
-8.870	1.877	1.863	1.867	**1.868**	-8.910	1.810	1.876	1.845	**1.860**
-8.860	1.851	1.897	1.875	**1.868**	-8.900	1.852	1.916	1.922	**1.860**
-8.850	1.856	1.849	1.909	**1.868**	-8.890	1.931	1.839	1.916	**1.860**
-8.840	1.893	1.867	1.899	**1.868**	-8.880	1.883	1.901	1.875	**1.860**
-8.830	1.850	1.871	1.868	**1.868**	-8.870	1.816	1.813	1.826	**1.860**
-8.820	1.939	1.883	1.903	**1.868**	-8.860	1.954	1.929	1.855	**1.860**
-8.810	1.884	1.897	1.844	**1.868**	-8.850	1.808	1.901	1.828	**1.860**
-8.800	1.903	1.915	1.879	**1.868**	-8.840	1.865	1.885	1.865	**1.860**
-8.790	1.903	1.831	1.890	**1.868**	-8.830	1.949	1.863	1.951	**1.860**
-8.780	1.907	1.864	1.878	**1.868**	-8.820	1.934	1.897	1.958	**1.860**
-8.770	1.844	1.899	1.838	**1.868**	-8.810	1.877	1.877	1.969	**1.860**
-8.760	1.846	1.855	1.792	**1.869**	-8.800	1.906	1.902	1.856	**1.861**
-8.750	1.878	1.834	1.830	**1.869**	-8.790	1.937	1.870	1.850	**1.861**
-8.740	1.888	1.905	1.914	**1.869**	-8.780	1.797	1.895	1.858	**1.862**
-8.730	1.872	1.867	1.831	**1.869**	-8.770	1.868	1.899	1.854	**1.862**
-8.720	1.850	1.849	1.876	**1.870**	-8.760	1.867	1.914	1.882	**1.863**
-8.710	1.900	1.865	1.932	**1.870**	-8.750	1.816	1.905	1.868	**1.864**
-8.700	1.898	1.909	1.844	**1.870**	-8.740	1.792	1.901	1.871	**1.865**
-8.690	1.890	1.905	1.836	**1.870**	-8.730	1.787	1.752	1.815	**1.865**
-8.680	1.857	1.900	1.888	**1.871**	-8.720	1.835	1.926	1.851	**1.866**
-8.670	1.837	1.896	1.908	**1.871**	-8.710	1.881	1.899	1.816	**1.866**
-8.660	1.869	1.899	1.897	**1.871**	-8.700	1.871	1.937	1.845	**1.867**
-8.650	1.869	1.873	1.879	**1.872**	-8.690	1.971	1.859	1.919	**1.868**
-8.640	1.821	1.889	1.873	**1.872**	-8.680	1.858	1.866	1.925	**1.868**
-8.630	1.876	1.849	1.836	**1.872**	-8.670	1.831	1.872	1.886	**1.868**
-8.620	1.879	1.838	1.868	**1.872**	-8.660	1.895	1.877	1.887	**1.869**
-8.610	1.836	1.868	1.910	**1.872**	-8.650	1.843	1.861	1.838	**1.869**
-8.600	1.829	1.884	1.896	**1.872**	-8.640	1.859	1.902	1.848	**1.869**
-8.590	1.873	1.835	1.843	**1.872**	-8.630	1.889	1.848	1.810	**1.869**
-8.580	1.855	1.842	1.856	**1.872**	-8.620	1.874	1.854	1.903	**1.870**
-8.570	1.876	1.862	1.877	**1.872**	-8.610	1.909	1.903	1.816	**1.870**

-8.560	1.878	1.832	1.908	**1.871**	-8.600	1.857	1.838	1.862	**1.870**
-8.550	1.881	1.838	1.856	**1.871**	-8.590	1.813	1.794	1.859	**1.870**
-8.540	1.898	1.825	1.878	**1.871**	-8.580	1.848	1.833	1.839	**1.870**
-8.530	1.853	1.856	1.873	**1.871**	-8.570	1.862	1.929	1.873	**1.870**
-8.520	1.854	1.835	1.806	**1.871**	-8.560	1.843	1.858	1.919	**1.870**
-8.510	1.837	1.840	1.853	**1.871**	-8.550	1.850	1.914	1.933	**1.871**
-8.500	1.888	1.822	1.862	**1.871**	-8.540	1.946	1.885	1.911	**1.871**
-8.490	1.888	1.853	1.879	**1.871**	-8.530	1.881	1.896	1.879	**1.871**
-8.480	1.874	1.850	1.872	**1.870**	-8.520	1.933	1.920	1.894	**1.871**
-8.470	1.944	1.914	1.914	**1.870**	-8.510	1.799	1.756	1.808	**1.872**
-8.460	1.862	1.856	1.879	**1.870**	-8.500	1.931	1.894	1.843	**1.872**
-8.450	1.894	1.854	1.865	**1.870**	-8.490	1.856	1.856	1.766	**1.872**
-8.440	1.864	1.827	1.862	**1.870**	-8.480	1.871	1.866	1.885	**1.872**
-8.430	1.857	1.861	1.866	**1.870**	-8.470	1.856	1.913	1.876	**1.871**
-8.420	1.900	1.832	1.843	**1.870**	-8.460	1.914	1.942	1.959	**1.871**
-8.410	1.897	1.831	1.892	**1.870**	-8.450	1.911	1.947	1.905	**1.871**
-8.400	1.902	1.924	1.937	**1.870**	-8.440	1.846	1.913	1.918	**1.871**
-8.390	1.836	1.810	1.873	**1.869**	-8.430	1.934	1.862	1.955	**1.871**
-8.380	1.871	1.878	1.836	**1.869**	-8.420	1.852	1.865	1.770	**1.871**
-8.370	1.863	1.903	1.848	**1.870**	-8.410	1.822	1.820	1.842	**1.871**
-8.360	1.853	1.864	1.823	**1.870**	-8.400	1.817	1.831	1.781	**1.871**
-8.350	1.870	1.911	1.864	**1.871**	-8.390	1.824	1.827	1.749	**1.871**
-8.340	1.901	1.865	1.861	**1.871**	-8.380	1.913	1.831	1.878	**1.870**
-8.330	1.871	1.867	1.895	**1.872**	-8.370	1.889	1.792	1.852	**1.870**
-8.320	1.870	1.872	1.879	**1.872**	-8.360	1.882	1.885	1.937	**1.870**
-8.310	1.879	1.871	1.918	**1.873**	-8.350	1.895	1.861	1.910	**1.869**
-8.300	1.877	1.937	1.886	**1.873**	-8.340	1.854	1.893	1.790	**1.869**
-8.290	1.877	1.922	1.861	**1.874**	-8.330	1.844	1.905	1.941	**1.869**
-8.280	1.837	1.804	1.792	**1.874**	-8.320	1.883	1.849	1.867	**1.868**
-8.270	1.852	1.897	1.916	**1.874**	-8.310	1.892	1.859	1.888	**1.867**
-8.260	1.886	1.913	1.894	**1.875**	-8.300	1.828	1.856	1.843	**1.867**
-8.250	1.867	1.867	1.917	**1.875**	-8.290	1.935	1.919	1.846	**1.866**

-8.240	1.903	1.936	1.955	**1.875**	-8.280	1.791	1.896	1.836	**1.865**
-8.230	1.857	1.879	1.887	**1.875**	-8.270	1.849	1.908	1.867	**1.865**
-8.220	1.845	1.859	1.845	**1.875**	-8.260	1.868	1.890	1.924	**1.864**
-8.210	1.815	1.870	1.856	**1.875**	-8.250	1.913	1.861	1.962	**1.863**
-8.200	1.871	1.851	1.921	**1.874**	-8.240	1.806	1.837	1.890	**1.862**
-8.190	1.908	1.885	1.871	**1.874**	-8.230	1.880	1.818	1.825	**1.861**
-8.180	1.866	1.857	1.861	**1.874**	-8.220	1.909	1.895	1.902	**1.861**
-8.170	1.847	1.882	1.866	**1.874**	-8.210	1.858	1.883	1.910	**1.860**
-8.160	1.848	1.920	1.910	**1.874**	-8.200	1.836	1.765	1.810	**1.860**
-8.150	1.920	1.856	1.877	**1.874**	-8.190	1.907	1.807	1.868	**1.860**
-8.140	1.848	1.835	1.822	**1.874**	-8.180	1.892	1.853	1.910	**1.860**
-8.130	1.854	1.900	1.856	**1.874**	-8.170	1.855	1.763	1.782	**1.860**
-8.120	1.921	1.878	1.867	**1.873**	-8.160	1.851	1.852	1.820	**1.860**
-8.110	1.816	1.778	1.837	**1.873**	-8.150	1.925	1.949	1.844	**1.860**
-8.100	1.866	1.941	1.887	**1.872**	-8.140	1.796	1.803	1.856	**1.860**
-8.090	1.902	1.942	1.887	**1.871**	-8.130	1.945	1.890	1.926	**1.860**
-8.080	1.888	1.852	1.831	**1.871**	-8.120	1.786	1.868	1.856	**1.860**
-8.070	1.840	1.892	1.862	**1.871**	-8.110	1.909	1.903	1.927	**1.860**
-8.060	1.825	1.882	1.863	**1.871**	-8.100	1.882	1.952	1.904	**1.860**
-8.050	1.903	1.898	1.915	**1.870**	-8.090	1.918	1.900	1.893	**1.860**
-8.040	1.858	1.815	1.811	**1.871**	-8.080	1.874	1.792	1.865	**1.860**
-8.030	1.882	1.857	1.929	**1.871**	-8.070	1.898	1.887	1.819	**1.860**
-8.020	1.841	1.891	1.882	**1.871**	-8.060	1.860	1.847	1.923	**1.860**
-8.010	1.834	1.812	1.876	**1.871**	-8.050	1.894	1.839	1.877	**1.860**
-8.000	1.811	1.882	1.865	**1.871**	-8.040	1.835	1.876	1.815	**1.860**
-7.990	1.832	1.851	1.865	**1.871**	-8.030	1.894	1.830	1.882	**1.860**
-7.980	1.935	1.857	1.895	**1.872**	-8.020	1.848	1.779	1.832	**1.861**
-7.970	1.870	1.873	1.828	**1.872**	-8.010	1.782	1.827	1.837	**1.861**
-7.960	1.891	1.875	1.871	**1.872**	-8.000	1.762	1.816	1.861	**1.861**
-7.950	1.826	1.875	1.877	**1.872**	-7.990	1.824	1.840	1.886	**1.861**
-7.940	1.878	1.873	1.916	**1.872**	-7.980	1.869	1.781	1.847	**1.861**
-7.930	1.874	1.885	1.915	**1.872**	-7.970	1.899	1.893	1.809	**1.861**

-7.920	1.903	1.843	1.882	**1.872**	-7.960	1.798	1.815	1.851	**1.861**
-7.910	1.914	1.891	1.885	**1.873**	-7.950	1.852	1.843	1.826	**1.861**
-7.900	1.902	1.883	1.871	**1.873**	-7.940	1.836	1.835	1.829	**1.861**
-7.890	1.858	1.834	1.850	**1.873**	-7.930	1.853	1.962	1.923	**1.861**
-7.880	1.835	1.864	1.834	**1.873**	-7.920	1.888	1.908	1.888	**1.861**
-7.870	1.871	1.839	1.865	**1.873**	-7.910	1.944	1.903	1.867	**1.861**
-7.860	1.856	1.895	1.861	**1.874**	-7.900	1.858	1.828	1.859	**1.861**
-7.850	1.813	1.856	1.858	**1.874**	-7.890	1.857	1.881	1.802	**1.861**
-7.840	1.861	1.827	1.904	**1.874**	-7.880	1.941	1.888	1.867	**1.861**
-7.830	1.842	1.863	1.856	**1.874**	-7.870	1.930	1.880	1.855	**1.861**
-7.820	1.917	1.882	1.843	**1.874**	-7.860	1.755	1.846	1.764	**1.861**
-7.810	1.853	1.878	1.923	**1.874**	-7.850	1.877	1.943	1.918	**1.861**
-7.800	1.915	1.854	1.912	**1.874**	-7.840	1.913	1.826	1.847	**1.861**
-7.790	1.861	1.825	1.894	**1.874**	-7.830	1.790	1.847	1.892	**1.861**
-7.780	1.896	1.867	1.909	**1.874**	-7.820	1.742	1.794	1.814	**1.861**
-7.770	1.879	1.905	1.906	**1.874**	-7.810	1.846	1.847	1.876	**1.862**
-7.760	1.843	1.840	1.853	**1.875**	-7.800	1.832	1.896	1.929	**1.862**
-7.750	1.896	1.832	1.844	**1.875**	-7.790	1.909	1.934	1.871	**1.862**
-7.740	1.866	1.857	1.874	**1.875**	-7.780	1.860	1.923	1.817	**1.862**
-7.730	1.938	1.893	1.888	**1.875**	-7.770	1.910	1.825	1.859	**1.862**
-7.720	1.906	1.876	1.884	**1.875**	-7.760	1.825	1.920	1.894	**1.862**
-7.710	1.893	1.838	1.901	**1.875**	-7.750	1.830	1.815	1.865	**1.862**
-7.700	1.853	1.898	1.862	**1.875**	-7.740	1.865	1.901	1.835	**1.862**
-7.690	1.879	1.885	1.843	**1.875**	-7.730	1.901	1.870	1.860	**1.862**
-7.680	1.876	1.897	1.880	**1.875**	-7.720	1.833	1.865	1.826	**1.862**
-7.670	1.879	1.805	1.832	**1.875**	-7.710	1.853	1.854	1.821	**1.863**
-7.660	1.858	1.902	1.836	**1.874**	-7.700	1.815	1.911	1.888	**1.863**
-7.650	1.861	1.840	1.872	**1.874**	-7.690	1.915	1.872	1.909	**1.863**
-7.640	1.864	1.828	1.834	**1.874**	-7.680	1.818	1.854	1.826	**1.864**
-7.630	1.860	1.853	1.877	**1.874**	-7.670	1.903	1.814	1.842	**1.864**
-7.620	1.868	1.882	1.825	**1.874**	-7.660	1.863	1.839	1.846	**1.864**
-7.610	1.891	1.840	1.888	**1.874**	-7.650	1.885	1.824	1.899	**1.865**

-7.600	1.928	1.894	1.879	**1.874**	-7.640	1.841	1.932	1.892	**1.865**
-7.590	1.853	1.853	1.890	**1.874**	-7.630	1.772	1.851	1.831	**1.865**
-7.580	1.832	1.902	1.882	**1.874**	-7.620	1.846	1.820	1.835	**1.866**
-7.570	1.847	1.898	1.909	**1.874**	-7.610	1.892	1.872	1.899	**1.866**
-7.560	1.856	1.850	1.847	**1.874**	-7.600	1.869	1.800	1.866	**1.866**
-7.550	1.907	1.850	1.868	**1.874**	-7.590	1.840	1.781	1.878	**1.867**
-7.540	1.871	1.856	1.820	**1.874**	-7.580	1.888	1.841	1.791	**1.867**
-7.530	1.885	1.879	1.884	**1.874**	-7.570	1.870	1.865	1.890	**1.868**
-7.520	1.917	1.878	1.855	**1.874**	-7.560	1.927	1.916	1.926	**1.868**
-7.510	1.840	1.915	1.891	**1.874**	-7.550	1.875	1.922	1.946	**1.868**
-7.500	1.874	1.915	1.838	**1.874**	-7.540	1.898	1.879	1.902	**1.869**
-7.490	1.882	1.911	1.843	**1.873**	-7.530	1.805	1.881	1.795	**1.869**
-7.480	1.856	1.831	1.874	**1.873**	-7.520	1.871	1.874	1.882	**1.869**
-7.470	1.853	1.880	1.810	**1.873**	-7.510	1.887	1.857	1.949	**1.869**
-7.460	1.833	1.831	1.854	**1.873**	-7.500	1.928	1.819	1.885	**1.870**
-7.450	1.836	1.885	1.889	**1.873**	-7.490	1.771	1.814	1.805	**1.870**
-7.440	1.924	1.908	1.856	**1.873**	-7.480	1.825	1.915	1.913	**1.870**
-7.430	1.894	1.831	1.880	**1.872**	-7.470	1.839	1.809	1.855	**1.870**
-7.420	1.847	1.897	1.855	**1.872**	-7.460	1.803	1.827	1.872	**1.870**
-7.410	1.878	1.886	1.856	**1.872**	-7.450	1.910	1.835	1.821	**1.870**
-7.400	1.911	1.878	1.839	**1.871**	-7.440	1.850	1.817	1.914	**1.870**
-7.390	1.859	1.831	1.824	**1.871**	-7.430	1.814	1.870	1.825	**1.870**
-7.380	1.889	1.856	1.818	**1.871**	-7.420	1.862	1.913	1.895	**1.870**
-7.370	1.856	1.878	1.875	**1.871**	-7.410	1.981	1.909	1.935	**1.870**
-7.360	1.863	1.860	1.830	**1.871**	-7.400	1.897	1.869	1.913	**1.870**
-7.350	1.924	1.889	1.903	**1.870**	-7.390	1.814	1.784	1.736	**1.870**
-7.340	1.839	1.821	1.840	**1.870**	-7.380	1.901	1.901	1.990	**1.871**
-7.330	1.880	1.887	1.937	**1.870**	-7.370	1.839	1.821	1.831	**1.871**
-7.320	1.869	1.888	1.870	**1.870**	-7.360	1.914	1.882	1.922	**1.871**
-7.310	1.855	1.840	1.819	**1.869**	-7.350	1.847	1.862	1.948	**1.871**
-7.300	1.927	1.856	1.868	**1.869**	-7.340	1.854	1.855	1.816	**1.872**
-7.290	1.849	1.874	1.864	**1.869**	-7.330	1.885	1.868	1.787	**1.872**

-7.280	1.903	1.897	1.876	**1.869**	-7.320	1.908	1.930	1.876	**1.872**
-7.270	1.936	1.907	1.903	**1.869**	-7.310	1.874	1.827	1.869	**1.873**
-7.260	1.911	1.899	1.910	**1.869**	-7.300	1.926	1.832	1.829	**1.873**
-7.250	1.912	1.920	1.930	**1.869**	-7.290	1.890	1.906	1.878	**1.873**
-7.240	1.861	1.867	1.863	**1.868**	-7.280	1.761	1.757	1.847	**1.873**
-7.230	1.940	1.870	1.933	**1.868**	-7.270	1.845	1.840	1.825	**1.873**
-7.220	1.890	1.831	1.879	**1.868**	-7.260	1.882	1.870	1.814	**1.873**
-7.210	1.909	1.910	1.856	**1.868**	-7.250	1.836	1.866	1.917	**1.872**
-7.200	1.863	1.873	1.909	**1.868**	-7.240	1.839	1.801	1.910	**1.872**
-7.190	1.900	1.870	1.830	**1.867**	-7.230	1.859	1.875	1.876	**1.872**
-7.180	1.876	1.854	1.909	**1.867**	-7.220	1.855	1.788	1.880	**1.872**
-7.170	1.898	1.822	1.874	**1.867**	-7.210	1.807	1.821	1.897	**1.872**
-7.160	1.851	1.853	1.823	**1.867**	-7.200	1.906	1.876	1.801	**1.872**
-7.150	1.801	1.865	1.837	**1.866**	-7.190	1.884	1.881	1.886	**1.871**
-7.140	1.842	1.917	1.890	**1.866**	-7.180	1.830	1.886	1.868	**1.871**
-7.130	1.924	1.864	1.925	**1.866**	-7.170	1.797	1.859	1.894	**1.871**
-7.120	1.888	1.864	1.821	**1.866**	-7.160	1.915	1.906	1.894	**1.870**
-7.110	1.893	1.851	1.827	**1.866**	-7.150	1.772	1.794	1.780	**1.870**
-7.100	1.897	1.872	1.907	**1.866**	-7.140	1.826	1.914	1.908	**1.870**
-7.090	1.837	1.885	1.874	**1.866**	-7.130	1.815	1.846	1.923	**1.869**
-7.080	1.841	1.839	1.831	**1.865**	-7.120	1.900	1.948	1.972	**1.869**
-7.070	1.864	1.910	1.903	**1.865**	-7.110	1.844	1.887	1.845	**1.868**
-7.060	1.854	1.845	1.908	**1.865**	-7.100	1.890	1.979	1.945	**1.868**
-7.050	1.861	1.865	1.860	**1.865**	-7.090	1.913	1.820	1.840	**1.867**
-7.040	1.857	1.835	1.844	**1.865**	-7.080	1.846	1.935	1.868	**1.867**
-7.030	1.869	1.885	1.842	**1.865**	-7.070	1.910	1.837	1.812	**1.867**
-7.020	1.851	1.874	1.871	**1.866**	-7.060	1.874	1.934	1.850	**1.867**
-7.010	1.896	1.911	1.855	**1.866**	-7.050	1.841	1.817	1.808	**1.867**
-7.000	1.879	1.884	1.925	**1.867**	-7.040	1.874	1.911	1.932	**1.867**
-6.990	1.900	1.909	1.865	**1.867**	-7.030	1.944	1.864	1.868	**1.867**
-6.980	1.832	1.864	1.849	**1.867**	-7.020	1.933	1.923	1.888	**1.867**
-6.970	1.886	1.951	1.883	**1.868**	-7.010	1.860	1.816	1.840	**1.867**

-6.960	1.880	1.878	1.853	**1.868**	-7.000	1.887	1.864	1.847	**1.867**
-6.950	1.881	1.883	1.824	**1.868**	-6.990	1.754	1.861	1.829	**1.867**
-6.940	1.908	1.870	1.837	**1.868**	-6.980	1.913	1.870	1.804	**1.867**
-6.930	1.883	1.815	1.880	**1.869**	-6.970	1.913	1.825	1.836	**1.867**
-6.920	1.877	1.855	1.830	**1.869**	-6.960	1.877	1.802	1.801	**1.867**
-6.910	1.890	1.891	1.900	**1.869**	-6.950	1.914	1.888	1.807	**1.867**
-6.900	1.890	1.874	1.850	**1.869**	-6.940	1.907	1.844	1.897	**1.866**
-6.890	1.880	1.859	1.807	**1.869**	-6.930	1.902	1.866	1.826	**1.866**
-6.880	1.893	1.904	1.879	**1.869**	-6.920	1.826	1.841	1.870	**1.866**
-6.870	1.860	1.901	1.928	**1.869**	-6.910	1.768	1.777	1.860	**1.866**
-6.860	1.885	1.859	1.904	**1.869**	-6.900	1.863	1.831	1.864	**1.867**
-6.850	1.895	1.879	1.904	**1.868**	-6.890	1.875	1.852	1.888	**1.867**
-6.840	1.859	1.877	1.822	**1.868**	-6.880	1.847	1.903	1.901	**1.867**
-6.830	1.832	1.857	1.793	**1.868**	-6.870	1.861	1.807	1.846	**1.867**
-6.820	1.901	1.853	1.867	**1.868**	-6.860	1.799	1.891	1.815	**1.867**
-6.810	1.863	1.889	1.845	**1.868**	-6.850	1.893	1.834	1.831	**1.867**
-6.800	1.885	1.882	1.944	**1.868**	-6.840	1.827	1.875	1.865	**1.867**
-6.790	1.841	1.871	1.794	**1.867**	-6.830	1.835	1.815	1.851	**1.868**
-6.780	1.855	1.814	1.890	**1.867**	-6.820	1.919	1.872	1.905	**1.868**
-6.770	1.903	1.861	1.890	**1.867**	-6.810	1.863	1.910	1.855	**1.868**
-6.760	1.894	1.905	1.957	**1.867**	-6.800	1.855	1.872	1.820	**1.869**
-6.750	1.858	1.865	1.865	**1.867**	-6.790	1.900	1.905	1.918	**1.869**
-6.740	1.854	1.905	1.853	**1.866**	-6.780	1.833	1.886	1.863	**1.869**
-6.730	1.853	1.867	1.841	**1.866**	-6.770	1.842	1.824	1.928	**1.869**
-6.720	1.897	1.887	1.833	**1.866**	-6.760	1.896	1.935	1.990	**1.869**
-6.710	1.914	1.878	1.872	**1.866**	-6.750	1.887	1.880	1.856	**1.870**
-6.700	1.872	1.861	1.852	**1.866**	-6.740	1.863	1.830	1.895	**1.870**
-6.690	1.879	1.872	1.881	**1.866**	-6.730	1.841	1.843	1.896	**1.870**
-6.680	1.831	1.808	1.821	**1.865**	-6.720	1.838	1.786	1.758	**1.871**
-6.670	1.841	1.869	1.830	**1.865**	-6.710	1.935	1.849	1.939	**1.871**
-6.660	1.861	1.905	1.854	**1.865**	-6.700	1.809	1.862	1.868	**1.871**
-6.650	1.884	1.858	1.914	**1.866**	-6.690	1.889	1.824	1.827	**1.871**

-6.640	1.847	1.836	1.864	**1.866**	-6.680	1.877	1.839	1.945	**1.871**
-6.630	1.830	1.887	1.846	**1.866**	-6.670	1.874	1.925	1.863	**1.872**
-6.620	1.864	1.837	1.871	**1.866**	-6.660	1.865	1.847	1.845	**1.872**
-6.610	1.875	1.853	1.908	**1.866**	-6.650	1.806	1.757	1.846	**1.872**
-6.600	1.868	1.882	1.865	**1.866**	-6.640	1.862	1.822	1.900	**1.872**
-6.590	1.820	1.883	1.848	**1.866**	-6.630	1.876	1.797	1.820	**1.872**
-6.580	1.822	1.841	1.868	**1.866**	-6.620	1.871	1.844	1.887	**1.872**
-6.570	1.904	1.846	1.885	**1.866**	-6.610	1.847	1.867	1.763	**1.872**
-6.560	1.864	1.868	1.845	**1.866**	-6.600	1.829	1.860	1.937	**1.872**
-6.550	1.852	1.869	1.873	**1.867**	-6.590	1.906	1.878	1.886	**1.872**
-6.540	1.938	1.898	1.900	**1.867**	-6.580	1.843	1.817	1.822	**1.872**
-6.530	1.858	1.850	1.885	**1.867**	-6.570	1.917	1.896	1.914	**1.872**
-6.520	1.885	1.871	1.933	**1.867**	-6.560	1.834	1.871	1.817	**1.871**
-6.510	1.888	1.869	1.863	**1.867**	-6.550	1.882	1.804	1.882	**1.871**
-6.500	1.914	1.897	1.872	**1.867**	-6.540	1.837	1.807	1.751	**1.871**
-6.490	1.816	1.827	1.862	**1.867**	-6.530	1.939	1.924	1.928	**1.871**
-6.480	1.863	1.896	1.921	**1.867**	-6.520	1.874	1.906	1.899	**1.871**
-6.470	1.892	1.893	1.858	**1.867**	-6.510	1.875	1.871	1.835	**1.871**
-6.460	1.908	1.916	1.900	**1.867**	-6.500	1.830	1.888	1.796	**1.871**
-6.450	1.889	1.852	1.849	**1.867**	-6.490	1.886	1.829	1.904	**1.871**
-6.440	1.906	1.860	1.913	**1.867**	-6.480	1.956	1.945	1.892	**1.870**
-6.430	1.892	1.854	1.869	**1.867**	-6.470	1.883	1.861	1.923	**1.870**
-6.420	1.893	1.858	1.912	**1.867**	-6.460	1.842	1.761	1.844	**1.869**
-6.410	1.885	1.886	1.930	**1.867**	-6.450	1.924	1.913	1.834	**1.869**
-6.400	1.887	1.879	1.900	**1.867**	-6.440	1.893	1.921	1.884	**1.869**
-6.390	1.863	1.843	1.837	**1.867**	-6.430	1.815	1.820	1.862	**1.868**
-6.380	1.842	1.886	1.836	**1.867**	-6.420	1.854	1.834	1.810	**1.868**
-6.370	1.843	1.879	1.861	**1.867**	-6.410	1.880	1.864	1.816	**1.867**
-6.360	1.856	1.910	1.864	**1.867**	-6.400	1.784	1.843	1.753	**1.867**
-6.350	1.865	1.928	1.865	**1.867**	-6.390	1.895	1.895	1.867	**1.867**
-6.340	1.860	1.868	1.867	**1.867**	-6.380	1.850	1.818	1.894	**1.867**
-6.330	1.907	1.833	1.855	**1.867**	-6.370	1.894	1.914	1.906	**1.867**

-6.320	1.863	1.864	1.849	**1.867**	-6.360	1.901	1.841	1.849	**1.867**
-6.310	1.842	1.909	1.873	**1.867**	-6.350	1.788	1.863	1.850	**1.867**
-6.300	1.827	1.844	1.854	**1.867**	-6.340	1.930	1.873	1.897	**1.867**
-6.290	1.835	1.880	1.819	**1.867**	-6.330	1.883	1.881	1.860	**1.867**
-6.280	1.890	1.822	1.877	**1.867**	-6.320	1.859	1.782	1.854	**1.867**
-6.270	1.881	1.861	1.807	**1.866**	-6.310	1.855	1.935	1.836	**1.867**
-6.260	1.892	1.910	1.871	**1.866**	-6.300	1.825	1.826	1.885	**1.867**
-6.250	1.866	1.887	1.859	**1.866**	-6.290	1.912	1.900	1.886	**1.866**
-6.240	1.891	1.848	1.919	**1.865**	-6.280	1.852	1.910	1.896	**1.866**
-6.230	1.869	1.839	1.860	**1.865**	-6.270	1.894	1.818	1.829	**1.866**
-6.220	1.835	1.877	1.868	**1.864**	-6.260	1.908	1.849	1.892	**1.866**
-6.210	1.810	1.852	1.834	**1.864**	-6.250	1.891	1.871	1.818	**1.866**
-6.200	1.898	1.852	1.853	**1.864**	-6.240	1.859	1.929	1.902	**1.866**
-6.190	1.908	1.848	1.883	**1.863**	-6.230	1.876	1.955	1.974	**1.866**
-6.180	1.840	1.832	1.861	**1.863**	-6.220	1.820	1.861	1.887	**1.866**
-6.170	1.900	1.853	1.847	**1.863**	-6.210	1.909	1.951	1.883	**1.866**
-6.160	1.887	1.857	1.905	**1.863**	-6.200	1.829	1.890	1.898	**1.866**
-6.150	1.879	1.912	1.893	**1.862**	-6.190	1.835	1.837	1.815	**1.866**
-6.140	1.885	1.908	1.850	**1.863**	-6.180	1.816	1.888	1.856	**1.866**
-6.130	1.859	1.879	1.809	**1.863**	-6.170	1.875	1.937	1.974	**1.866**
-6.120	1.916	1.852	1.901	**1.863**	-6.160	1.926	1.899	1.962	**1.866**
-6.110	1.861	1.844	1.837	**1.863**	-6.150	1.894	1.910	1.855	**1.866**
-6.100	1.872	1.850	1.879	**1.864**	-6.140	1.828	1.923	1.889	**1.866**
-6.090	1.894	1.850	1.844	**1.864**	-6.130	1.849	1.823	1.880	**1.866**
-6.080	1.843	1.847	1.821	**1.865**	-6.120	1.771	1.865	1.779	**1.866**
-6.070	1.832	1.837	1.893	**1.866**	-6.110	1.796	1.896	1.857	**1.866**
-6.060	1.817	1.868	1.877	**1.866**	-6.100	1.870	1.908	1.819	**1.866**
-6.050	1.866	1.810	1.868	**1.867**	-6.090	1.873	1.881	1.817	**1.866**
-6.040	1.851	1.849	1.836	**1.867**	-6.080	1.847	1.922	1.858	**1.866**
-6.030	1.882	1.849	1.854	**1.868**	-6.070	1.854	1.925	1.875	**1.867**
-6.020	1.909	1.916	1.895	**1.869**	-6.060	1.849	1.846	1.843	**1.867**
-6.010	1.860	1.884	1.839	**1.869**	-6.050	1.921	1.873	1.854	**1.867**

-6.000	1.851	1.820	1.843	**1.870**	-6.040	1.834	1.868	1.862	**1.867**
-5.990	1.831	1.826	1.860	**1.870**	-6.030	1.818	1.870	1.820	**1.867**
-5.980	1.920	1.852	1.914	**1.870**	-6.020	1.764	1.809	1.804	**1.867**
-5.970	1.889	1.872	1.861	**1.870**	-6.010	1.839	1.873	1.907	**1.868**
-5.960	1.898	1.912	1.865	**1.870**	-6.000	1.868	1.802	1.809	**1.868**
-5.950	1.931	1.893	1.878	**1.870**	-5.990	1.758	1.837	1.776	**1.868**
-5.940	1.827	1.843	1.865	**1.870**	-5.980	1.833	1.832	1.924	**1.868**
-5.930	1.847	1.837	1.865	**1.870**	-5.970	1.879	1.837	1.830	**1.868**
-5.920	1.861	1.873	1.859	**1.871**	-5.960	1.801	1.863	1.763	**1.868**
-5.910	1.908	1.909	1.880	**1.871**	-5.950	1.824	1.819	1.857	**1.868**
-5.900	1.866	1.902	1.927	**1.871**	-5.940	1.890	1.905	1.835	**1.868**
-5.890	1.849	1.834	1.811	**1.871**	-5.930	1.847	1.914	1.913	**1.868**
-5.880	1.853	1.833	1.871	**1.871**	-5.920	1.840	1.800	1.802	**1.868**
-5.870	1.853	1.905	1.878	**1.871**	-5.910	1.897	1.863	1.912	**1.868**
-5.860	1.895	1.826	1.858	**1.871**	-5.900	1.905	1.855	1.920	**1.868**
-5.850	1.864	1.840	1.865	**1.871**	-5.890	1.848	1.874	1.844	**1.869**
-5.840	1.883	1.871	1.921	**1.871**	-5.880	1.843	1.856	1.912	**1.869**
-5.830	1.844	1.860	1.867	**1.871**	-5.870	1.887	1.844	1.844	**1.868**
-5.820	1.824	1.886	1.886	**1.871**	-5.860	1.870	1.908	1.926	**1.868**
-5.810	1.889	1.869	1.929	**1.871**	-5.850	1.885	1.940	1.880	**1.868**
-5.800	1.831	1.903	1.880	**1.871**	-5.840	1.846	1.806	1.846	**1.868**
-5.790	1.863	1.899	1.937	**1.871**	-5.830	1.847	1.888	1.882	**1.868**
-5.780	1.894	1.837	1.877	**1.871**	-5.820	1.917	1.858	1.888	**1.868**
-5.770	1.894	1.840	1.866	**1.871**	-5.810	1.878	1.882	1.909	**1.869**
-5.760	1.907	1.869	1.902	**1.870**	-5.800	1.984	1.916	1.973	**1.869**
-5.750	1.889	1.859	1.898	**1.870**	-5.790	1.831	1.826	1.849	**1.870**
-5.740	1.837	1.836	1.818	**1.869**	-5.780	1.822	1.918	1.851	**1.870**
-5.730	1.848	1.885	1.841	**1.869**	-5.770	1.838	1.859	1.875	**1.871**
-5.720	1.838	1.877	1.847	**1.868**	-5.760	1.803	1.802	1.800	**1.871**
-5.710	1.835	1.835	1.817	**1.868**	-5.750	1.823	1.826	1.882	**1.872**
-5.700	1.870	1.899	1.881	**1.867**	-5.740	1.810	1.863	1.871	**1.872**
-5.690	1.830	1.825	1.846	**1.867**	-5.730	1.840	1.865	1.828	**1.873**

-5.680	1.866	1.878	1.840	**1.866**	-5.720	1.825	1.910	1.879	**1.873**
-5.670	1.794	1.855	1.862	**1.865**	-5.710	1.824	1.869	1.834	**1.874**
-5.660	1.903	1.871	1.829	**1.865**	-5.700	1.855	1.869	1.814	**1.874**
-5.650	1.924	1.856	1.879	**1.864**	-5.690	1.811	1.868	1.848	**1.874**
-5.640	1.812	1.863	1.830	**1.864**	-5.680	1.871	1.818	1.806	**1.875**
-5.630	1.854	1.833	1.866	**1.864**	-5.670	1.840	1.946	1.918	**1.875**
-5.620	1.879	1.867	1.940	**1.864**	-5.660	1.886	1.893	1.930	**1.875**
-5.610	1.917	1.912	1.847	**1.864**	-5.650	1.910	1.887	1.852	**1.876**
-5.600	1.819	1.872	1.875	**1.864**	-5.640	1.850	1.768	1.853	**1.876**
-5.590	1.853	1.910	1.859	**1.865**	-5.630	1.912	1.858	1.862	**1.876**
-5.580	1.875	1.828	1.879	**1.865**	-5.620	1.883	1.832	1.886	**1.876**
-5.570	1.864	1.888	1.888	**1.865**	-5.610	1.802	1.894	1.864	**1.876**
-5.560	1.834	1.857	1.887	**1.866**	-5.600	1.855	1.882	1.902	**1.876**
-5.550	1.811	1.872	1.853	**1.867**	-5.590	1.842	1.869	1.892	**1.876**
-5.540	1.860	1.863	1.804	**1.867**	-5.580	1.881	1.964	1.910	**1.876**
-5.530	1.809	1.880	1.854	**1.868**	-5.570	1.822	1.838	1.910	**1.876**
-5.520	1.889	1.863	1.857	**1.868**	-5.560	1.931	1.886	1.901	**1.876**
-5.510	1.919	1.881	1.858	**1.869**	-5.550	1.840	1.861	1.881	**1.876**
-5.500	1.941	1.888	1.868	**1.869**	-5.540	1.929	1.862	1.879	**1.876**
-5.490	1.877	1.873	1.921	**1.870**	-5.530	1.831	1.808	1.907	**1.876**
-5.480	1.893	1.861	1.829	**1.870**	-5.520	1.919	1.893	1.888	**1.876**
-5.470	1.869	1.825	1.861	**1.870**	-5.510	1.855	1.810	1.874	**1.875**
-5.460	1.889	1.859	1.900	**1.870**	-5.500	1.893	1.865	1.858	**1.875**
-5.450	1.898	1.886	1.867	**1.871**	-5.490	1.917	1.861	1.829	**1.875**
-5.440	1.816	1.833	1.852	**1.871**	-5.480	1.859	1.885	1.818	**1.875**
-5.430	1.855	1.910	1.869	**1.871**	-5.470	1.889	1.970	1.899	**1.874**
-5.420	1.895	1.871	1.881	**1.872**	-5.460	1.819	1.820	1.905	**1.874**
-5.410	1.835	1.823	1.813	**1.872**	-5.450	1.794	1.888	1.827	**1.874**
-5.400	1.838	1.871	1.838	**1.872**	-5.440	1.978	1.876	1.891	**1.874**
-5.390	1.856	1.894	1.897	**1.872**	-5.430	1.766	1.827	1.823	**1.873**
-5.380	1.919	1.854	1.909	**1.872**	-5.420	1.915	1.941	1.864	**1.873**
-5.370	1.844	1.906	1.846	**1.872**	-5.410	1.890	1.866	1.870	**1.873**

-5.360	1.852	1.894	1.879	**1.872**	-5.400	1.840	1.815	1.890	**1.873**
-5.350	1.877	1.816	1.875	**1.871**	-5.390	1.901	1.913	1.892	**1.872**
-5.340	1.877	1.858	1.915	**1.871**	-5.380	1.924	1.843	1.851	**1.872**
-5.330	1.931	1.879	1.899	**1.871**	-5.370	1.869	1.872	1.875	**1.872**
-5.320	1.879	1.878	1.892	**1.870**	-5.360	1.795	1.833	1.868	**1.872**
-5.310	1.819	1.855	1.868	**1.870**	-5.350	1.809	1.790	1.866	**1.871**
-5.300	1.835	1.905	1.874	**1.870**	-5.340	1.855	1.861	1.812	**1.871**
-5.290	1.826	1.824	1.816	**1.869**	-5.330	1.891	1.964	1.876	**1.871**
-5.280	1.833	1.850	1.863	**1.869**	-5.320	1.803	1.829	1.866	**1.871**
-5.270	1.877	1.915	1.847	**1.869**	-5.310	1.908	1.956	1.885	**1.871**
-5.260	1.838	1.869	1.856	**1.869**	-5.300	1.887	1.938	1.876	**1.871**
-5.250	1.880	1.925	1.859	**1.868**	-5.290	1.882	1.901	1.961	**1.870**
-5.240	1.850	1.859	1.872	**1.868**	-5.280	1.943	1.869	1.837	**1.870**
-5.230	1.879	1.869	1.873	**1.868**	-5.270	1.920	1.844	1.826	**1.870**
-5.220	1.890	1.866	1.871	**1.868**	-5.260	1.871	1.897	1.914	**1.870**
-5.210	1.889	1.831	1.845	**1.867**	-5.250	1.797	1.796	1.794	**1.869**
-5.200	1.852	1.910	1.854	**1.867**	-5.240	1.775	1.864	1.876	**1.869**
-5.190	1.860	1.897	1.854	**1.867**	-5.230	1.833	1.784	1.891	**1.869**
-5.180	1.903	1.882	1.883	**1.867**	-5.220	1.874	1.798	1.795	**1.868**
-5.170	1.825	1.811	1.784	**1.867**	-5.210	1.868	1.900	1.868	**1.868**
-5.160	1.864	1.838	1.898	**1.867**	-5.200	1.870	1.868	1.925	**1.868**
-5.150	1.902	1.913	1.953	**1.866**	-5.190	1.846	1.838	1.888	**1.867**
-5.140	1.928	1.898	1.865	**1.866**	-5.180	1.842	1.873	1.915	**1.868**
-5.130	1.811	1.848	1.806	**1.866**	-5.170	1.870	1.887	1.930	**1.868**
-5.120	1.858	1.867	1.895	**1.866**	-5.160	1.873	1.823	1.836	**1.868**
-5.110	1.879	1.829	1.896	**1.866**	-5.150	1.797	1.781	1.850	**1.868**
-5.100	1.840	1.830	1.840	**1.865**	-5.140	1.902	1.881	1.893	**1.868**
-5.090	1.837	1.877	1.816	**1.865**	-5.130	1.912	1.904	1.920	**1.869**
-5.080	1.842	1.835	1.861	**1.865**	-5.120	1.894	1.833	1.788	**1.869**
-5.070	1.886	1.899	1.874	**1.865**	-5.110	1.824	1.851	1.924	**1.869**
-5.060	1.889	1.949	1.879	**1.865**	-5.100	1.876	1.853	1.864	**1.869**
-5.050	1.852	1.868	1.850	**1.866**	-5.090	1.872	1.886	1.807	**1.869**

-5.040	1.891	1.905	1.888	**1.866**	-5.080	1.812	1.818	1.840	**1.869**
-5.030	1.871	1.836	1.835	**1.867**	-5.070	1.952	1.921	1.909	**1.869**
-5.020	1.843	1.898	1.889	**1.867**	-5.060	1.867	1.878	1.828	**1.869**
-5.010	1.874	1.815	1.863	**1.867**	-5.050	1.913	1.965	1.894	**1.868**
-5.000	1.840	1.820	1.826	**1.867**	-5.040	1.820	1.876	1.878	**1.868**
-4.990	1.839	1.870	1.815	**1.868**	-5.030	1.894	1.898	1.872	**1.868**
-4.980	1.865	1.867	1.909	**1.868**	-5.020	1.880	1.855	1.801	**1.868**
-4.970	1.873	1.907	1.881	**1.868**	-5.010	1.902	1.863	1.933	**1.868**
-4.960	1.828	1.897	1.858	**1.869**	-5.000	1.873	1.808	1.887	**1.867**
-4.950	1.884	1.831	1.875	**1.869**	-4.990	1.810	1.897	1.837	**1.867**
-4.940	1.887	1.836	1.860	**1.869**	-4.980	1.950	1.849	1.885	**1.867**
-4.930	1.864	1.913	1.863	**1.869**	-4.970	1.883	1.864	1.964	**1.866**
-4.920	1.893	1.830	1.848	**1.870**	-4.960	1.848	1.789	1.812	**1.866**
-4.910	1.866	1.884	1.811	**1.870**	-4.950	1.848	1.898	1.818	**1.866**
-4.900	1.901	1.876	1.926	**1.870**	-4.940	1.854	1.847	1.813	**1.865**
-4.890	1.846	1.906	1.847	**1.870**	-4.930	1.862	1.905	1.805	**1.865**
-4.880	1.865	1.862	1.912	**1.870**	-4.920	1.777	1.880	1.820	**1.865**
-4.870	1.838	1.877	1.860	**1.870**	-4.910	1.821	1.863	1.931	**1.864**
-4.860	1.870	1.863	1.819	**1.871**	-4.900	1.884	1.838	1.878	**1.864**
-4.850	1.936	1.915	1.911	**1.871**	-4.890	1.932	1.835	1.870	**1.864**
-4.840	1.916	1.953	1.887	**1.871**	-4.880	1.932	1.850	1.899	**1.863**
-4.830	1.872	1.903	1.846	**1.872**	-4.870	1.940	1.895	1.869	**1.863**
-4.820	1.859	1.822	1.879	**1.872**	-4.860	1.863	1.888	1.858	**1.862**
-4.810	1.862	1.919	1.926	**1.873**	-4.850	1.886	1.887	1.837	**1.862**
-4.800	1.845	1.838	1.876	**1.873**	-4.840	1.867	1.847	1.878	**1.862**
-4.790	1.881	1.838	1.848	**1.873**	-4.830	1.841	1.887	1.908	**1.862**
-4.780	1.878	1.869	1.882	**1.874**	-4.820	1.854	1.938	1.858	**1.862**
-4.770	1.858	1.873	1.868	**1.874**	-4.810	1.849	1.888	1.878	**1.862**
-4.760	1.888	1.897	1.860	**1.874**	-4.800	2.022	1.927	1.938	**1.862**
-4.750	1.859	1.813	1.885	**1.875**	-4.790	1.785	1.839	1.793	**1.862**
-4.740	1.829	1.849	1.866	**1.875**	-4.780	1.909	1.880	1.924	**1.862**
-4.730	1.898	1.879	1.887	**1.875**	-4.770	1.919	1.909	1.878	**1.862**

-4.720	1.922	1.893	1.869	**1.875**	-4.760	1.885	1.815	1.802	**1.862**
-4.710	1.870	1.908	1.933	**1.875**	-4.750	1.879	1.973	1.951	**1.862**
-4.700	1.819	1.845	1.864	**1.876**	-4.740	1.875	1.918	1.877	**1.862**
-4.690	1.849	1.829	1.879	**1.876**	-4.730	1.813	1.768	1.762	**1.862**
-4.680	1.857	1.851	1.877	**1.876**	-4.720	1.886	1.815	1.856	**1.862**
-4.670	1.887	1.841	1.871	**1.876**	-4.710	1.860	1.835	1.844	**1.862**
-4.660	1.842	1.867	1.893	**1.876**	-4.700	1.878	1.869	1.854	**1.862**
-4.650	1.872	1.856	1.809	**1.877**	-4.690	1.808	1.854	1.866	**1.863**
-4.640	1.880	1.860	1.885	**1.877**	-4.680	1.878	1.898	1.915	**1.863**
-4.630	1.841	1.885	1.847	**1.877**	-4.670	1.863	1.838	1.854	**1.864**
-4.620	1.879	1.840	1.896	**1.877**	-4.660	1.822	1.867	1.824	**1.864**
-4.610	1.859	1.859	1.794	**1.877**	-4.650	1.834	1.942	1.864	**1.864**
-4.600	1.861	1.882	1.874	**1.878**	-4.640	1.891	1.862	1.967	**1.865**
-4.590	1.918	1.893	1.908	**1.878**	-4.630	1.870	1.853	1.792	**1.865**
-4.580	1.894	1.921	1.900	**1.878**	-4.620	1.793	1.792	1.881	**1.865**
-4.570	1.888	1.912	1.876	**1.878**	-4.610	1.812	1.802	1.854	**1.865**
-4.560	1.865	1.920	1.856	**1.878**	-4.600	1.743	1.797	1.826	**1.866**
-4.550	1.882	1.919	1.915	**1.878**	-4.590	1.844	1.865	1.821	**1.866**
-4.540	1.894	1.898	1.880	**1.878**	-4.580	1.901	1.875	1.904	**1.866**
-4.530	1.887	1.920	1.924	**1.878**	-4.570	1.786	1.835	1.835	**1.867**
-4.520	1.874	1.933	1.892	**1.877**	-4.560	1.869	1.926	1.913	**1.867**
-4.510	1.891	1.863	1.826	**1.877**	-4.550	1.807	1.802	1.778	**1.867**
-4.500	1.843	1.904	1.844	**1.877**	-4.540	1.919	2.001	1.897	**1.868**
-4.490	1.924	1.909	1.862	**1.876**	-4.530	1.914	1.867	1.892	**1.868**
-4.480	1.842	1.885	1.877	**1.876**	-4.520	1.915	1.867	1.817	**1.868**
-4.470	1.877	1.923	1.895	**1.876**	-4.510	1.810	1.850	1.797	**1.868**
-4.460	1.839	1.885	1.871	**1.875**	-4.500	1.862	1.891	1.873	**1.868**
-4.450	1.919	1.912	1.900	**1.875**	-4.490	1.938	1.845	1.884	**1.869**
-4.440	1.838	1.847	1.861	**1.875**	-4.480	1.895	1.837	1.847	**1.869**
-4.430	1.832	1.819	1.833	**1.874**	-4.470	1.898	1.885	1.870	**1.869**
-4.420	1.840	1.895	1.888	**1.874**	-4.460	1.858	1.899	1.944	**1.869**
-4.410	1.851	1.834	1.871	**1.874**	-4.450	1.831	1.788	1.882	**1.869**

-4.400	1.865	1.837	1.857	**1.874**	-4.440	1.882	1.850	1.827	**1.869**
-4.390	1.910	1.900	1.882	**1.874**	-4.430	1.834	1.906	1.916	**1.868**
-4.380	1.813	1.838	1.874	**1.874**	-4.420	1.889	1.841	1.915	**1.868**
-4.370	1.910	1.896	1.894	**1.874**	-4.410	1.856	1.882	1.882	**1.868**
-4.360	1.849	1.832	1.883	**1.874**	-4.400	1.882	1.792	1.859	**1.867**
-4.350	1.847	1.842	1.850	**1.873**	-4.390	1.915	1.861	1.864	**1.867**
-4.340	1.918	1.906	1.875	**1.873**	-4.380	1.845	1.860	1.933	**1.866**
-4.330	1.872	1.895	1.881	**1.873**	-4.370	1.933	1.908	1.873	**1.866**
-4.320	1.837	1.839	1.861	**1.873**	-4.360	1.863	1.855	1.938	**1.865**
-4.310	1.930	1.871	1.890	**1.873**	-4.350	1.881	1.802	1.876	**1.865**
-4.300	1.860	1.909	1.886	**1.872**	-4.340	1.873	1.864	1.854	**1.864**
-4.290	1.849	1.922	1.875	**1.872**	-4.330	1.865	1.860	1.884	**1.863**
-4.280	1.874	1.846	1.896	**1.872**	-4.320	1.882	1.859	1.852	**1.863**
-4.270	1.854	1.869	1.895	**1.871**	-4.310	1.820	1.883	1.836	**1.862**
-4.260	1.851	1.867	1.807	**1.871**	-4.300	1.819	1.885	1.859	**1.861**
-4.250	1.909	1.871	1.901	**1.871**	-4.290	1.894	1.935	1.905	**1.861**
-4.240	1.851	1.911	1.907	**1.870**	-4.280	1.829	1.804	1.837	**1.860**
-4.230	1.880	1.884	1.876	**1.870**	-4.270	1.824	1.889	1.839	**1.859**
-4.220	1.876	1.855	1.902	**1.870**	-4.260	1.901	1.834	1.806	**1.859**
-4.210	1.848	1.877	1.845	**1.869**	-4.250	1.859	1.898	1.904	**1.859**
-4.200	1.880	1.879	1.887	**1.869**	-4.240	1.829	1.934	1.875	**1.858**
-4.190	1.847	1.838	1.873	**1.869**	-4.230	1.839	1.909	1.832	**1.858**
-4.180	1.875	1.854	1.865	**1.869**	-4.220	1.824	1.815	1.809	**1.858**
-4.170	1.892	1.884	1.908	**1.868**	-4.210	1.827	1.892	1.845	**1.858**
-4.160	1.809	1.838	1.885	**1.868**	-4.200	1.881	1.870	1.923	**1.858**
-4.150	1.819	1.889	1.843	**1.868**	-4.190	1.766	1.842	1.800	**1.859**
-4.140	1.847	1.886	1.843	**1.868**	-4.180	1.846	1.884	1.791	**1.859**
-4.130	1.896	1.843	1.911	**1.867**	-4.170	1.847	1.852	1.804	**1.859**
-4.120	1.929	1.879	1.865	**1.867**	-4.160	1.796	1.859	1.838	**1.860**
-4.110	1.846	1.833	1.873	**1.866**	-4.150	1.855	1.791	1.864	**1.860**
-4.100	1.894	1.861	1.885	**1.866**	-4.140	1.868	1.867	1.879	**1.860**
-4.090	1.842	1.883	1.890	**1.865**	-4.130	1.877	1.906	1.825	**1.861**

-4.080	1.802	1.829	1.829	**1.865**	-4.120	1.881	1.892	1.877	**1.861**
-4.070	1.875	1.871	1.889	**1.865**	-4.110	1.825	1.877	1.858	**1.861**
-4.060	1.887	1.923	1.863	**1.865**	-4.100	1.916	1.859	1.854	**1.861**
-4.050	1.866	1.859	1.877	**1.865**	-4.090	1.827	1.887	1.937	**1.862**
-4.040	1.825	1.838	1.882	**1.865**	-4.080	1.864	1.853	1.877	**1.862**
-4.030	1.863	1.845	1.832	**1.865**	-4.070	1.931	1.964	1.892	**1.862**
-4.020	1.869	1.861	1.866	**1.865**	-4.060	1.907	1.929	1.942	**1.863**
-4.010	1.867	1.844	1.828	**1.866**	-4.050	1.857	1.956	1.938	**1.863**
-4.000	1.843	1.813	1.797	**1.866**	-4.040	1.832	1.906	1.848	**1.863**
-3.990	1.862	1.830	1.800	**1.866**	-4.030	1.828	1.848	1.757	**1.863**
-3.980	1.893	1.856	1.851	**1.867**	-4.020	1.787	1.842	1.829	**1.863**
-3.970	1.921	1.917	1.887	**1.867**	-4.010	1.846	1.813	1.859	**1.863**
-3.960	1.824	1.819	1.878	**1.867**	-4.000	1.828	1.832	1.808	**1.863**
-3.950	1.794	1.872	1.826	**1.867**	-3.990	1.861	1.862	1.834	**1.862**
-3.940	1.801	1.863	1.873	**1.867**	-3.980	1.836	1.897	1.836	**1.862**
-3.930	1.910	1.886	1.854	**1.867**	-3.970	1.883	1.828	1.872	**1.862**
-3.920	1.826	1.875	1.856	**1.867**	-3.960	1.965	1.891	1.895	**1.861**
-3.910	1.902	1.915	1.864	**1.867**	-3.950	1.840	1.850	1.906	**1.861**
-3.900	1.836	1.876	1.870	**1.866**	-3.940	1.890	1.904	1.816	**1.861**
-3.890	1.875	1.875	1.905	**1.866**	-3.930	1.909	1.852	1.869	**1.861**
-3.880	1.855	1.903	1.891	**1.866**	-3.920	1.960	1.933	1.951	**1.861**
-3.870	1.837	1.902	1.850	**1.865**	-3.910	1.825	1.813	1.862	**1.860**
-3.860	1.850	1.826	1.881	**1.865**	-3.900	1.862	1.877	1.817	**1.860**
-3.850	1.867	1.823	1.843	**1.865**	-3.890	1.842	1.879	1.872	**1.860**
-3.840	1.826	1.824	1.866	**1.864**	-3.880	1.902	1.905	1.845	**1.859**
-3.830	1.883	1.926	1.896	**1.864**	-3.870	1.877	1.867	1.913	**1.859**
-3.820	1.858	1.808	1.804	**1.864**	-3.860	1.841	1.828	1.802	**1.858**
-3.810	1.913	1.862	1.853	**1.863**	-3.850	1.874	1.861	1.831	**1.858**
-3.800	1.846	1.870	1.848	**1.863**	-3.840	1.840	1.880	1.911	**1.858**
-3.790	1.832	1.865	1.843	**1.864**	-3.830	1.855	1.852	1.885	**1.858**
-3.780	1.908	1.889	1.912	**1.864**	-3.820	1.862	1.864	1.866	**1.857**
-3.770	1.850	1.851	1.884	**1.865**	-3.810	1.819	1.848	1.857	**1.857**

-3.760	1.858	1.843	1.796	**1.866**	-3.800	1.901	1.892	1.934	**1.857**
-3.750	1.894	1.887	1.837	**1.868**	-3.790	1.865	1.876	1.855	**1.857**
-3.740	1.868	1.914	1.870	**1.869**	-3.780	1.833	1.795	1.879	**1.857**
-3.730	1.865	1.873	1.918	**1.871**	-3.770	1.819	1.833	1.833	**1.857**
-3.720	1.923	1.874	1.862	**1.873**	-3.760	1.890	1.852	1.806	**1.858**
-3.710	1.900	1.868	1.898	**1.877**	-3.750	1.801	1.867	1.805	**1.858**
-3.700	1.909	1.843	1.882	**1.881**	-3.740	1.812	1.823	1.804	**1.858**
-3.690	1.906	1.967	1.921	**1.885**	-3.730	1.870	1.787	1.820	**1.859**
-3.680	1.882	1.849	1.920	**1.888**	-3.720	1.909	1.855	1.932	**1.859**
-3.670	1.827	1.893	1.882	**1.892**	-3.710	1.794	1.898	1.832	**1.860**
-3.660	1.869	1.902	1.841	**1.895**	-3.700	1.926	1.840	1.899	**1.861**
-3.650	1.869	1.851	1.867	**1.899**	-3.690	1.838	1.824	1.785	**1.862**
-3.640	1.904	1.912	1.930	**1.902**	-3.680	1.831	1.812	1.822	**1.862**
-3.630	1.911	1.867	1.910	**1.906**	-3.670	1.767	1.828	1.816	**1.863**
-3.620	1.928	1.913	1.921	**1.909**	-3.660	1.806	1.890	1.828	**1.864**
-3.610	1.877	1.888	1.947	**1.913**	-3.650	1.887	1.852	1.888	**1.865**
-3.600	1.864	1.899	1.908	**1.916**	-3.640	1.857	1.814	1.879	**1.866**
-3.590	1.894	1.905	1.877	**1.920**	-3.630	1.925	1.854	1.952	**1.868**
-3.580	1.935	1.937	1.895	**1.923**	-3.620	1.887	1.838	1.837	**1.869**
-3.570	1.970	1.967	1.908	**1.927**	-3.610	1.830	1.903	1.820	**1.870**
-3.560	1.929	1.885	1.913	**1.930**	-3.600	1.858	1.822	1.831	**1.872**
-3.550	1.847	1.837	1.895	**1.934**	-3.590	1.841	1.846	1.827	**1.874**
-3.540	1.906	1.972	1.903	**1.937**	-3.580	1.890	1.817	1.890	**1.876**
-3.530	1.913	1.912	1.879	**1.941**	-3.570	1.829	1.870	1.887	**1.878**
-3.520	2.000	1.953	1.958	**1.944**	-3.560	1.849	1.888	1.807	**1.880**
-3.510	1.980	1.937	1.903	**1.948**	-3.550	1.876	1.876	1.842	**1.883**
-3.500	1.990	1.942	1.945	**1.951**	-3.540	1.902	1.911	1.878	**1.886**
-3.490	1.960	1.933	1.931	**1.955**	-3.530	1.960	1.938	1.866	**1.888**
-3.480	1.987	2.001	1.991	**1.958**	-3.520	1.914	1.876	1.893	**1.892**
-3.470	1.986	1.971	1.944	**1.962**	-3.510	1.893	1.893	1.801	**1.895**
-3.460	1.966	2.014	1.954	**1.965**	-3.500	1.852	1.852	1.900	**1.899**
-3.450	2.036	1.990	1.976	**1.969**	-3.490	1.938	1.893	1.950	**1.902**

-3.440	1.965	1.980	1.951	**1.972**	-3.480	1.848	1.865	1.772	**1.905**
-3.430	1.980	1.943	1.990	**1.976**	-3.470	1.903	1.895	1.886	**1.909**
-3.420	1.959	1.953	1.981	**1.979**	-3.460	1.896	1.846	1.881	**1.912**
-3.410	1.952	1.980	2.029	**1.983**	-3.450	1.949	1.883	1.866	**1.916**
-3.400	2.010	1.954	1.985	**1.986**	-3.440	1.890	1.931	1.977	**1.919**
-3.390	2.019	1.964	2.017	**1.990**	-3.430	1.983	1.947	1.917	**1.923**
-3.380	2.001	2.012	2.017	**1.993**	-3.420	1.945	2.018	1.919	**1.926**
-3.370	2.018	1.956	2.003	**1.996**	-3.410	1.929	1.979	2.017	**1.929**
-3.360	1.993	2.014	2.004	**2.000**	-3.400	1.972	1.955	1.919	**1.933**
-3.350	2.029	2.015	2.008	**2.003**	-3.390	1.848	1.928	1.898	**1.936**
-3.340	1.965	2.011	2.028	**2.007**	-3.380	1.928	1.985	1.910	**1.940**
-3.330	1.962	2.006	1.968	**2.010**	-3.370	1.942	2.006	2.001	**1.943**
-3.320	2.025	1.996	1.974	**2.014**	-3.360	2.036	1.975	1.985	**1.947**
-3.310	2.080	2.011	2.059	**2.017**	-3.350	2.019	1.918	1.944	**1.950**
-3.300	2.008	2.073	2.022	**2.021**	-3.340	1.902	1.975	1.982	**1.953**
-3.290	2.001	2.017	2.047	**2.024**	-3.330	1.964	1.994	1.912	**1.957**
-3.280	2.024	2.050	2.018	**2.028**	-3.320	1.909	1.922	1.970	**1.961**
-3.270	2.073	2.052	2.035	**2.031**	-3.310	2.042	2.017	2.021	**1.965**
-3.260	2.012	2.008	2.059	**2.035**	-3.300	1.992	2.081	1.989	**1.968**
-3.250	2.047	2.017	1.973	**2.038**	-3.290	1.982	2.010	1.934	**1.972**
-3.240	2.045	2.018	2.076	**2.041**	-3.280	1.982	1.926	1.998	**1.976**
-3.230	2.025	2.005	2.037	**2.044**	-3.270	1.984	1.919	1.914	**1.980**
-3.220	2.042	2.024	2.059	**2.047**	-3.260	1.900	1.904	1.972	**1.984**
-3.210	2.062	2.046	2.037	**2.050**	-3.250	1.973	1.942	1.985	**1.987**
-3.200	2.072	2.075	2.103	**2.053**	-3.240	1.918	1.902	1.941	**1.991**
-3.190	2.101	2.081	2.060	**2.056**	-3.230	2.084	1.977	2.009	**1.995**
-3.180	1.954	2.006	1.988	**2.058**	-3.220	2.112	2.014	2.034	**1.999**
-3.170	2.065	2.092	2.113	**2.061**	-3.210	1.996	1.977	2.025	**2.002**
-3.160	2.129	2.079	2.140	**2.064**	-3.200	1.946	1.946	1.909	**2.006**
-3.150	2.070	2.028	2.071	**2.067**	-3.190	2.077	2.089	2.046	**2.010**
-3.140	2.098	2.079	2.055	**2.070**	-3.180	2.043	2.034	2.000	**2.014**
-3.130	2.081	2.073	2.047	**2.073**	-3.170	2.022	1.991	1.932	**2.017**

-3.120	2.137	2.107	2.137	**2.076**	-3.160	2.073	2.071	1.992	**2.021**
-3.110	2.094	2.101	2.038	**2.079**	-3.150	2.019	2.031	1.997	**2.025**
-3.100	2.006	2.036	1.990	**2.082**	-3.140	1.995	1.998	2.072	**2.029**
-3.090	2.110	2.065	2.093	**2.085**	-3.130	2.047	1.947	2.034	**2.033**
-3.080	2.033	2.092	2.035	**2.088**	-3.120	2.055	2.032	2.037	**2.036**
-3.070	2.168	2.130	2.098	**2.091**	-3.110	2.098	1.999	2.062	**2.040**
-3.060	2.161	2.097	2.091	**2.092**	-3.100	1.963	2.000	2.006	**2.044**
-3.050	2.113	2.118	2.052	**2.094**	-3.090	2.022	2.007	2.094	**2.047**
-3.040	2.126	2.063	2.058	**2.096**	-3.080	2.049	2.026	2.035	**2.050**
-3.030	2.130	2.124	2.106	**2.098**	-3.070	2.055	1.968	2.065	**2.054**
-3.020	2.116	2.089	2.117	**2.100**	-3.060	2.069	2.061	2.037	**2.057**
-3.010	2.035	2.053	2.034	**2.101**	-3.050	2.054	1.970	2.058	**2.061**
-3.000	2.144	2.122	2.079	**2.103**	-3.040	2.027	2.025	2.044	**2.064**
-2.990	2.132	2.064	2.122	**2.104**	-3.030	2.052	2.086	2.007	**2.067**
-2.980	2.105	2.082	2.038	**2.105**	-3.020	2.090	2.112	2.058	**2.070**
-2.970	2.103	2.036	2.095	**2.106**	-3.010	1.977	2.076	2.030	**2.073**
-2.960	2.142	2.102	2.132	**2.107**	-3.000	2.104	2.054	1.995	**2.076**
-2.950	2.104	2.126	2.155	**2.108**	-2.990	2.023	2.037	2.032	**2.078**
-2.940	2.163	2.103	2.103	**2.109**	-2.980	2.101	2.069	2.171	**2.081**
-2.930	2.122	2.085	2.152	**2.109**	-2.970	2.182	2.078	2.104	**2.084**
-2.920	2.130	2.117	2.124	**2.109**	-2.960	2.103	2.046	2.086	**2.087**
-2.910	2.135	2.089	2.110	**2.110**	-2.950	2.157	2.112	2.085	**2.089**
-2.900	2.150	2.160	2.132	**2.110**	-2.940	2.039	2.072	2.023	**2.092**
-2.890	2.164	2.146	2.150	**2.111**	-2.930	2.123	2.103	2.132	**2.095**
-2.880	2.125	2.085	2.125	**2.111**	-2.920	2.050	2.158	2.082	**2.097**
-2.870	2.101	2.127	2.079	**2.112**	-2.910	2.052	2.133	2.083	**2.100**
-2.860	2.161	2.111	2.093	**2.112**	-2.900	2.102	2.154	2.140	**2.102**
-2.850	2.052	2.118	2.119	**2.113**	-2.890	2.092	2.039	2.098	**2.103**
-2.840	2.065	2.132	2.087	**2.113**	-2.880	2.089	2.154	2.057	**2.104**
-2.830	2.145	2.071	2.117	**2.114**	-2.870	2.109	2.072	2.080	**2.104**
-2.820	2.058	2.116	2.115	**2.115**	-2.860	2.114	2.059	2.137	**2.104**
-2.810	2.120	2.159	2.177	**2.115**	-2.850	2.071	2.027	2.071	**2.105**

-2.800	2.126	2.159	2.131	**2.116**	-2.840	2.102	2.170	2.191	**2.105**
-2.790	2.108	2.101	2.164	**2.116**	-2.830	2.023	2.132	2.061	**2.106**
-2.780	2.123	2.142	2.100	**2.116**	-2.820	2.109	2.077	2.107	**2.106**
-2.770	2.148	2.120	2.156	**2.116**	-2.810	2.106	2.141	2.039	**2.107**
-2.760	2.130	2.110	2.099	**2.116**	-2.800	2.161	2.140	2.193	**2.107**
-2.750	2.116	2.073	2.085	**2.116**	-2.790	2.139	2.046	2.116	**2.107**
-2.740	2.081	2.117	2.115	**2.117**	-2.780	2.073	2.140	2.094	**2.108**
-2.730	2.100	2.094	2.112	**2.117**	-2.770	2.100	2.119	2.092	**2.108**
-2.720	2.115	2.137	2.110	**2.117**	-2.760	2.088	2.141	2.189	**2.109**
-2.710	2.099	2.063	2.138	**2.117**	-2.750	2.196	2.109	2.122	**2.109**
-2.700	2.115	2.142	2.119	**2.117**	-2.740	2.051	2.029	2.073	**2.110**
-2.690	2.143	2.119	2.093	**2.117**	-2.730	2.088	2.060	2.050	**2.110**
-2.680	2.112	2.094	2.054	**2.117**	-2.720	2.109	2.214	2.190	**2.110**
-2.670	2.104	2.090	2.056	**2.116**	-2.710	2.114	2.138	2.136	**2.111**
-2.660	2.131	2.136	2.135	**2.116**	-2.700	2.095	2.074	2.046	**2.111**
-2.650	2.117	2.108	2.115	**2.116**	-2.690	2.149	2.143	2.141	**2.111**
-2.640	2.098	2.130	2.168	**2.116**	-2.680	2.137	2.161	2.175	**2.111**
-2.630	2.144	2.137	2.151	**2.116**	-2.670	2.194	2.184	2.113	**2.111**
-2.620	2.099	2.095	2.123	**2.115**	-2.660	2.142	2.152	2.120	**2.111**
-2.610	2.111	2.120	2.108	**2.115**	-2.650	2.059	2.073	2.083	**2.111**
-2.600	2.131	2.131	2.152	**2.115**	-2.640	2.130	2.115	2.155	**2.111**
-2.590	2.134	2.142	2.137	**2.114**	-2.630	2.042	2.070	2.046	**2.111**
-2.580	2.119	2.146	2.101	**2.114**	-2.620	2.198	2.149	2.102	**2.111**
-2.570	2.114	2.129	2.096	**2.113**	-2.610	2.070	2.144	2.074	**2.111**
-2.560	2.121	2.057	2.060	**2.113**	-2.600	2.071	2.055	2.126	**2.111**
-2.550	2.125	2.091	2.063	**2.112**	-2.590	2.130	2.142	2.088	**2.111**
-2.540	2.078	2.109	2.138	**2.112**	-2.580	2.118	2.067	2.113	**2.111**
-2.530	2.090	2.070	2.080	**2.112**	-2.570	2.158	2.135	2.148	**2.111**
-2.520	2.099	2.096	2.100	**2.112**	-2.560	2.157	2.130	2.097	**2.111**
-2.510	2.132	2.091	2.130	**2.111**	-2.550	2.060	2.004	2.105	**2.111**
-2.500	2.089	2.048	2.124	**2.111**	-2.540	2.072	2.074	2.065	**2.111**
-2.490	2.143	2.098	2.112	**2.111**	-2.530	2.111	2.056	2.107	**2.110**

-2.480	2.066	2.083	2.101	**2.111**	-2.520	2.191	2.119	2.218	**2.110**
-2.470	2.109	2.119	2.074	**2.111**	-2.510	2.063	2.041	2.125	**2.110**
-2.460	2.097	2.106	2.126	**2.111**	-2.500	2.092	2.081	2.084	**2.110**
-2.450	2.096	2.090	2.113	**2.111**	-2.490	2.141	2.062	2.112	**2.110**
-2.440	2.139	2.174	2.143	**2.111**	-2.480	2.068	2.063	2.111	**2.109**
-2.430	2.184	2.130	2.167	**2.111**	-2.470	2.144	2.104	2.147	**2.109**
-2.420	2.061	2.133	2.103	**2.111**	-2.460	2.200	2.165	2.134	**2.109**
-2.410	2.066	2.043	2.095	**2.111**	-2.450	2.091	2.119	2.042	**2.109**
-2.400	2.103	2.096	2.099	**2.111**	-2.440	2.109	2.163	2.123	**2.109**
-2.390	2.120	2.103	2.126	**2.111**	-2.430	2.133	2.177	2.067	**2.109**
-2.380	2.145	2.141	2.108	**2.111**	-2.420	2.142	2.197	2.093	**2.109**
-2.370	2.109	2.111	2.099	**2.111**	-2.410	2.060	2.074	2.093	**2.108**
-2.360	2.129	2.059	2.077	**2.111**	-2.400	2.151	2.156	2.086	**2.108**
-2.350	2.077	2.102	2.131	**2.110**	-2.390	2.093	2.189	2.102	**2.108**
-2.340	2.164	2.107	2.134	**2.110**	-2.380	2.016	2.052	2.100	**2.108**
-2.330	2.149	2.168	2.126	**2.110**	-2.370	2.077	2.078	2.128	**2.108**
-2.320	2.095	2.119	2.107	**2.110**	-2.360	2.106	2.111	2.195	**2.108**
-2.310	2.069	2.131	2.079	**2.110**	-2.350	2.173	2.069	2.140	**2.108**
-2.300	2.134	2.165	2.138	**2.110**	-2.340	2.101	2.050	2.145	**2.108**
-2.290	2.114	2.149	2.111	**2.111**	-2.330	2.139	2.155	2.064	**2.108**
-2.280	2.101	2.111	2.114	**2.111**	-2.320	2.055	2.095	2.114	**2.108**
-2.270	2.156	2.096	2.129	**2.111**	-2.310	2.159	2.078	2.112	**2.108**
-2.260	2.120	2.087	2.082	**2.111**	-2.300	2.064	2.047	2.046	**2.108**
-2.250	2.064	2.113	2.140	**2.111**	-2.290	2.187	2.121	2.158	**2.108**
-2.240	2.107	2.046	2.055	**2.111**	-2.280	2.082	2.068	2.127	**2.108**
-2.230	2.047	2.081	2.123	**2.111**	-2.270	2.128	2.077	2.145	**2.107**
-2.220	2.098	2.042	2.091	**2.111**	-2.260	2.055	2.123	2.032	**2.107**
-2.210	2.115	2.163	2.160	**2.111**	-2.250	2.153	2.066	2.119	**2.107**
-2.200	2.053	2.094	2.082	**2.111**	-2.240	2.143	2.167	2.208	**2.107**
-2.190	2.145	2.160	2.106	**2.110**	-2.230	2.120	2.092	2.048	**2.107**
-2.180	2.084	2.096	2.109	**2.110**	-2.220	2.095	2.057	2.058	**2.108**
-2.170	2.057	2.121	2.098	**2.110**	-2.210	2.171	2.073	2.138	**2.108**

-2.160	2.156	2.090	2.123	**2.110**	-2.200	2.049	2.103	2.128	**2.108**
-2.150	2.124	2.120	2.164	**2.110**	-2.190	2.122	2.050	2.158	**2.108**
-2.140	2.119	2.094	2.114	**2.109**	-2.180	2.095	2.113	2.064	**2.108**
-2.130	2.150	2.102	2.104	**2.109**	-2.170	2.131	2.137	2.149	**2.108**
-2.120	2.113	2.065	2.064	**2.109**	-2.160	2.140	2.052	2.138	**2.108**
-2.110	2.067	2.082	2.066	**2.109**	-2.150	2.171	2.123	2.081	**2.108**
-2.100	2.073	2.127	2.142	**2.109**	-2.140	2.046	2.143	2.121	**2.108**
-2.090	2.154	2.141	2.130	**2.109**	-2.130	2.059	2.131	2.066	**2.109**
-2.080	2.178	2.106	2.146	**2.109**	-2.120	2.159	2.088	2.144	**2.109**
-2.070	2.157	2.124	2.079	**2.109**	-2.110	2.076	2.088	2.148	**2.109**
-2.060	2.117	2.097	2.123	**2.109**	-2.100	2.122	2.116	2.115	**2.109**
-2.050	2.127	2.083	2.101	**2.109**	-2.090	2.145	2.099	2.123	**2.110**
-2.040	2.134	2.171	2.146	**2.110**	-2.080	2.096	2.127	2.158	**2.110**
-2.030	2.076	2.116	2.125	**2.110**	-2.070	2.114	2.065	2.147	**2.110**
-2.020	2.119	2.094	2.068	**2.110**	-2.060	2.140	2.112	2.086	**2.111**
-2.010	2.090	2.125	2.116	**2.110**	-2.050	2.155	2.175	2.172	**2.111**
-2.000	2.065	2.061	2.091	**2.111**	-2.040	2.137	2.132	2.113	**2.111**
-1.990	2.093	2.165	2.131	**2.111**	-2.030	2.131	2.084	2.171	**2.112**
-1.980	2.093	2.111	2.115	**2.111**	-2.020	2.067	2.122	2.096	**2.112**
-1.970	2.125	2.094	2.151	**2.111**	-2.010	2.088	2.144	2.088	**2.112**
-1.960	2.136	2.181	2.110	**2.111**	-2.000	2.114	2.053	2.034	**2.112**
-1.950	2.114	2.151	2.105	**2.111**	-1.990	2.105	2.121	2.031	**2.113**
-1.940	2.104	2.105	2.152	**2.112**	-1.980	2.184	2.170	2.218	**2.113**
-1.930	2.113	2.136	2.077	**2.112**	-1.970	2.131	2.092	2.055	**2.113**
-1.920	2.132	2.101	2.163	**2.112**	-1.960	2.111	2.213	2.145	**2.113**
-1.910	2.124	2.118	2.140	**2.112**	-1.950	2.192	2.151	2.158	**2.113**
-1.900	2.200	2.172	2.130	**2.112**	-1.940	2.098	2.087	2.096	**2.113**
-1.890	2.119	2.138	2.131	**2.113**	-1.930	2.097	2.092	2.102	**2.113**
-1.880	2.099	2.114	2.104	**2.113**	-1.920	2.125	2.108	2.132	**2.113**
-1.870	2.095	2.049	2.057	**2.113**	-1.910	2.030	2.029	2.126	**2.113**
-1.860	2.142	2.097	2.108	**2.113**	-1.900	2.071	2.095	2.092	**2.113**
-1.850	2.096	2.120	2.110	**2.113**	-1.890	2.067	2.112	2.021	**2.112**

-1.840	2.113	2.065	2.082	**2.113**	-1.880	2.092	2.079	2.109	**2.112**
-1.830	2.048	2.104	2.045	**2.113**	-1.870	2.111	2.106	2.173	**2.112**
-1.820	2.131	2.094	2.159	**2.113**	-1.860	2.117	2.169	2.088	**2.112**
-1.810	2.075	2.116	2.088	**2.112**	-1.850	2.063	2.143	2.115	**2.112**
-1.800	2.150	2.183	2.127	**2.112**	-1.840	2.086	2.104	2.038	**2.112**
-1.790	2.132	2.126	2.091	**2.112**	-1.830	2.176	2.190	2.205	**2.112**
-1.780	2.147	2.092	2.124	**2.112**	-1.820	2.156	2.146	2.090	**2.112**
-1.770	2.090	2.061	2.133	**2.111**	-1.810	2.012	2.055	2.113	**2.112**
-1.760	2.111	2.068	2.082	**2.111**	-1.800	2.116	2.131	2.098	**2.111**
-1.750	2.132	2.076	2.141	**2.111**	-1.790	2.060	1.973	2.047	**2.111**
-1.740	2.098	2.127	2.054	**2.111**	-1.780	2.154	2.154	2.153	**2.111**
-1.730	2.103	2.104	2.148	**2.111**	-1.770	2.059	2.126	2.125	**2.111**
-1.720	2.113	2.124	2.085	**2.110**	-1.760	2.138	2.203	2.135	**2.111**
-1.710	2.174	2.117	2.147	**2.110**	-1.750	2.120	2.164	2.106	**2.111**
-1.700	2.106	2.109	2.081	**2.110**	-1.740	2.136	2.134	2.076	**2.111**
-1.690	2.102	2.169	2.148	**2.110**	-1.730	2.034	2.134	2.122	**2.111**
-1.680	2.118	2.131	2.114	**2.110**	-1.720	2.083	2.094	2.103	**2.111**
-1.670	2.059	2.072	2.104	**2.109**	-1.710	2.102	2.110	2.161	**2.111**
-1.660	2.104	2.112	2.171	**2.109**	-1.700	2.141	2.220	2.191	**2.110**
-1.650	2.108	2.124	2.094	**2.109**	-1.690	2.024	2.072	2.107	**2.110**
-1.640	2.115	2.132	2.093	**2.109**	-1.680	2.101	2.071	1.995	**2.110**
-1.630	2.084	2.050	2.075	**2.108**	-1.670	2.175	2.196	2.104	**2.110**
-1.620	2.084	2.092	2.065	**2.108**	-1.660	2.061	2.094	2.085	**2.110**
-1.610	2.147	2.111	2.146	**2.108**	-1.650	2.087	2.084	2.051	**2.109**
-1.600	2.104	2.080	2.102	**2.108**	-1.640	2.159	2.130	2.147	**2.109**
-1.590	2.095	2.153	2.102	**2.108**	-1.630	2.175	2.090	2.077	**2.108**
-1.580	2.131	2.105	2.066	**2.107**	-1.620	2.106	2.174	2.105	**2.108**
-1.570	2.120	2.050	2.081	**2.107**	-1.610	2.039	2.007	2.043	**2.108**
-1.560	2.122	2.099	2.065	**2.107**	-1.600	2.073	2.101	2.166	**2.108**
-1.550	2.099	2.080	2.091	**2.106**	-1.590	2.160	2.087	2.173	**2.107**
-1.540	2.109	2.080	2.149	**2.106**	-1.580	2.142	2.117	2.143	**2.107**
-1.530	2.033	2.092	2.076	**2.106**	-1.570	2.088	2.126	2.096	**2.106**

-1.520	2.101	2.086	2.142	**2.105**	-1.560	2.084	2.166	2.137	**2.106**
-1.510	2.105	2.077	2.126	**2.105**	-1.550	2.168	2.118	2.181	**2.105**
-1.500	2.102	2.173	2.127	**2.104**	-1.540	2.018	2.058	2.127	**2.105**
-1.490	2.126	2.118	2.098	**2.103**	-1.530	2.073	2.078	2.089	**2.104**
-1.480	2.114	2.087	2.075	**2.103**	-1.520	2.057	2.034	2.075	**2.104**
-1.470	2.138	2.148	2.118	**2.102**	-1.510	2.114	2.108	2.147	**2.103**
-1.460	2.094	2.132	2.135	**2.101**	-1.500	2.026	2.016	2.013	**2.103**
-1.450	2.147	2.100	2.137	**2.101**	-1.490	2.057	2.124	2.075	**2.103**
-1.440	2.134	2.128	2.083	**2.100**	-1.480	2.136	2.175	2.099	**2.102**
-1.430	2.123	2.089	2.051	**2.099**	-1.470	2.034	2.063	2.090	**2.102**
-1.420	2.129	2.094	2.065	**2.099**	-1.460	2.126	2.116	2.102	**2.102**
-1.410	2.091	2.118	2.100	**2.098**	-1.450	2.069	2.108	2.138	**2.102**
-1.400	2.073	2.086	2.062	**2.097**	-1.440	2.113	2.178	2.122	**2.101**
-1.390	2.113	2.081	2.115	**2.097**	-1.430	2.172	2.146	2.129	**2.101**
-1.380	2.075	2.125	2.140	**2.096**	-1.420	2.015	2.118	2.091	**2.101**
-1.370	2.140	2.193	2.169	**2.095**	-1.410	2.145	2.069	2.167	**2.100**
-1.360	2.088	2.045	2.090	**2.095**	-1.400	2.033	2.125	2.108	**2.099**
-1.350	2.093	2.091	2.063	**2.094**	-1.390	2.107	2.107	2.164	**2.099**
-1.340	2.068	2.079	2.082	**2.094**	-1.380	2.049	2.109	2.097	**2.098**
-1.330	2.067	2.103	2.111	**2.093**	-1.370	2.159	2.049	2.120	**2.097**
-1.320	2.064	2.035	2.093	**2.093**	-1.360	2.112	2.115	2.204	**2.097**
-1.310	2.096	2.110	2.148	**2.092**	-1.350	2.085	2.067	2.120	**2.096**
-1.300	2.049	2.050	2.071	**2.092**	-1.340	2.063	2.128	2.157	**2.096**
-1.290	2.137	2.111	2.076	**2.091**	-1.330	2.093	2.072	2.074	**2.095**
-1.280	2.120	2.108	2.073	**2.090**	-1.320	2.091	2.102	2.031	**2.095**
-1.270	2.058	2.029	2.038	**2.090**	-1.310	2.077	2.042	2.114	**2.094**
-1.260	2.139	2.080	2.078	**2.089**	-1.300	2.113	2.021	2.076	**2.093**
-1.250	2.095	2.123	2.087	**2.088**	-1.290	2.079	2.105	2.106	**2.093**
-1.240	2.076	2.061	2.116	**2.088**	-1.280	2.065	2.079	2.149	**2.092**
-1.230	2.058	2.118	2.082	**2.087**	-1.270	2.022	2.035	2.040	**2.092**
-1.220	2.098	2.112	2.107	**2.087**	-1.260	2.121	2.101	2.040	**2.091**
-1.210	2.100	2.161	2.149	**2.087**	-1.250	2.080	2.119	2.158	**2.091**

-1.200	2.103	2.104	2.067	**2.086**	-1.240	2.144	2.136	2.059	**2.090**
-1.190	2.054	2.075	2.095	**2.086**	-1.230	2.128	2.028	2.055	**2.089**
-1.180	2.106	2.130	2.070	**2.086**	-1.220	2.048	2.010	2.047	**2.089**
-1.170	2.071	2.039	2.093	**2.086**	-1.210	2.068	2.032	2.129	**2.088**
-1.160	2.044	2.038	2.103	**2.086**	-1.200	2.072	2.015	2.065	**2.087**
-1.150	2.067	2.127	2.085	**2.086**	-1.190	2.117	2.060	2.140	**2.086**
-1.140	2.075	2.108	2.070	**2.086**	-1.180	1.998	2.060	2.044	**2.086**
-1.130	2.137	2.071	2.085	**2.087**	-1.170	2.079	2.067	2.131	**2.085**
-1.120	2.055	2.072	2.039	**2.088**	-1.160	2.076	2.063	2.080	**2.085**
-1.110	2.067	2.070	2.111	**2.088**	-1.150	2.036	2.120	2.110	**2.085**
-1.100	2.085	2.103	2.126	**2.089**	-1.140	2.141	2.074	2.038	**2.085**
-1.090	2.129	2.086	2.069	**2.090**	-1.130	2.219	2.157	2.108	**2.085**
-1.080	2.099	2.102	2.060	**2.091**	-1.120	2.174	2.175	2.123	**2.085**
-1.070	2.159	2.124	2.087	**2.092**	-1.110	2.097	2.097	2.113	**2.086**
-1.060	2.118	2.092	2.120	**2.093**	-1.100	2.097	2.186	2.180	**2.086**
-1.050	2.089	2.064	2.117	**2.094**	-1.090	2.100	2.065	2.095	**2.087**
-1.040	2.117	2.095	2.103	**2.095**	-1.080	2.039	2.146	2.116	**2.087**
-1.030	2.068	2.066	2.122	**2.097**	-1.070	2.100	2.003	2.014	**2.088**
-1.020	2.059	2.104	2.054	**2.098**	-1.060	2.107	2.208	2.118	**2.089**
-1.010	2.047	2.092	2.123	**2.098**	-1.050	2.055	2.157	2.097	**2.089**
-1.000	2.068	2.129	2.075	**2.099**	-1.040	2.097	2.151	2.070	**2.089**
-0.990	2.125	2.090	2.088	**2.100**	-1.030	2.141	2.053	2.108	**2.090**
-0.980	2.139	2.114	2.109	**2.100**	-1.020	2.091	2.046	2.084	**2.090**
-0.970	2.067	2.137	2.076	**2.101**	-1.010	2.087	2.177	2.120	**2.090**
-0.960	2.080	2.107	2.120	**2.101**	-1.000	2.118	2.071	2.168	**2.091**
-0.950	2.046	2.102	2.112	**2.101**	-0.990	2.026	2.103	2.113	**2.091**
-0.940	2.141	2.125	2.097	**2.102**	-0.980	2.086	2.030	2.069	**2.091**
-0.930	2.042	2.095	2.054	**2.102**	-0.970	2.099	2.159	2.093	**2.091**
-0.920	2.110	2.083	2.101	**2.102**	-0.960	2.039	2.073	2.089	**2.092**
-0.910	2.184	2.126	2.125	**2.102**	-0.950	2.064	2.087	2.039	**2.093**
-0.900	2.091	2.088	2.137	**2.103**	-0.940	2.072	2.104	2.074	**2.094**
-0.890	2.093	2.087	2.087	**2.103**	-0.930	2.168	2.143	2.062	**2.094**

-0.880	2.085	2.104	2.152	**2.103**	-0.920	2.126	2.060	2.057	**2.095**
-0.870	2.060	2.108	2.103	**2.103**	-0.910	2.002	2.091	2.076	**2.095**
-0.860	2.133	2.092	2.061	**2.103**	-0.900	2.062	2.128	2.021	**2.096**
-0.850	2.125	2.128	2.106	**2.103**	-0.890	2.097	2.126	2.079	**2.096**
-0.840	2.108	2.067	2.141	**2.103**	-0.880	2.035	2.064	2.064	**2.097**
-0.830	2.110	2.112	2.159	**2.103**	-0.870	2.100	2.059	2.122	**2.097**
-0.820	2.065	2.105	2.109	**2.103**	-0.860	2.171	2.092	2.145	**2.098**
-0.810	2.126	2.069	2.123	**2.104**	-0.850	2.109	2.064	2.133	**2.098**
-0.800	2.045	2.078	2.110	**2.104**	-0.840	2.106	2.141	2.147	**2.099**
-0.790	2.109	2.102	2.069	**2.104**	-0.830	2.082	2.135	2.108	**2.100**
-0.780	2.085	2.045	2.054	**2.104**	-0.820	2.054	2.107	2.082	**2.100**
-0.770	2.113	2.057	2.116	**2.104**	-0.810	2.107	2.074	2.155	**2.101**
-0.760	2.068	2.096	2.051	**2.104**	-0.800	2.026	2.127	2.089	**2.101**
-0.750	2.106	2.128	2.130	**2.104**	-0.790	2.098	2.141	2.153	**2.101**
-0.740	2.060	2.114	2.083	**2.103**	-0.780	2.141	2.063	2.145	**2.102**
-0.730	2.045	2.057	2.105	**2.103**	-0.770	2.029	2.134	2.067	**2.102**
-0.720	2.092	2.059	2.125	**2.103**	-0.760	2.124	2.146	2.127	**2.103**
-0.710	2.089	2.138	2.112	**2.103**	-0.750	2.044	2.046	2.104	**2.103**
-0.700	2.097	2.120	2.093	**2.103**	-0.740	2.151	2.165	2.139	**2.103**
-0.690	2.078	2.114	2.104	**2.103**	-0.730	2.144	2.128	2.140	**2.103**
-0.680	2.115	2.054	2.055	**2.103**	-0.720	2.072	2.100	2.055	**2.103**
-0.670	2.109	2.087	2.085	**2.102**	-0.710	2.061	2.139	2.036	**2.103**
-0.660	2.090	2.015	2.038	**2.102**	-0.700	2.115	2.198	2.122	**2.104**
-0.650	2.050	2.122	2.099	**2.102**	-0.690	2.090	2.048	2.088	**2.104**
-0.640	2.118	2.147	2.096	**2.102**	-0.680	2.205	2.134	2.095	**2.103**
-0.630	2.147	2.110	2.150	**2.102**	-0.670	2.126	2.214	2.152	**2.103**
-0.620	2.074	2.078	2.120	**2.102**	-0.660	2.024	2.044	2.060	**2.103**
-0.610	2.151	2.108	2.146	**2.102**	-0.650	2.106	2.069	2.115	**2.103**
-0.600	2.101	2.034	2.053	**2.103**	-0.640	2.124	2.045	2.136	**2.102**
-0.590	2.130	2.133	2.102	**2.103**	-0.630	2.136	2.141	2.136	**2.102**
-0.580	2.092	2.122	2.074	**2.103**	-0.620	2.057	2.037	2.119	**2.102**
-0.570	2.103	2.053	2.118	**2.103**	-0.610	2.075	2.049	2.146	**2.102**

-0.560	2.132	2.079	2.073	**2.103**
-0.550	2.116	2.112	2.118	**2.103**
-0.540	2.139	2.119	2.176	**2.103**
-0.530	2.115	2.123	2.133	**2.103**
-0.520	2.093	2.056	2.123	**2.103**
-0.510	2.112	2.103	2.089	**2.103**
-0.500	2.074	2.067	2.096	**2.102**
-0.490	2.075	2.017	2.053	**2.102**
-0.480	2.080	2.050	2.122	**2.102**
-0.470	2.078	2.125	2.071	**2.101**

-0.600	2.078	2.093	2.055	**2.101**
-0.590	2.074	2.112	2.116	**2.101**
-0.580	2.039	2.081	2.122	**2.101**
-0.570	2.142	2.085	2.094	**2.101**
-0.560	2.090	2.063	2.138	**2.101**
-0.550	2.103	2.136	2.060	**2.100**
-0.540	2.082	2.120	2.150	**2.100**
-0.530	2.098	2.028	2.129	**2.100**
-0.520	2.105	2.088	2.001	**2.100**
-0.510	2.113	2.146	2.142	**2.100**
-0.500	2.124	2.100	2.057	**2.100**
-0.490	2.168	2.103	2.084	**2.099**
-0.480	2.080	2.078	2.071	**2.099**
-0.470	2.127	2.052	2.143	**2.099**
-0.460	2.094	2.126	2.037	**2.099**
-0.450	2.059	2.113	2.104	**2.099**
-0.440	2.104	2.022	2.109	**2.099**
-0.430	2.039	2.140	2.090	**2.099**
-0.420	2.128	2.102	2.050	**2.099**
-0.410	2.186	2.114	2.140	**2.099**
-0.400	2.046	2.055	2.101	**2.099**
-0.390	2.186	2.108	2.136	**2.099**
-0.380	2.135	2.119	2.107	**2.099**
