# Supplementary material for: Impact of drill bit wear on temperature increase in dental implant osteotomy: an in vitro study
Source: PLoS One. 2025 Mar 19;20(3):e0319492. doi: 10.1371/journal.pone.0319492 (PMC11922234; doi:10.1371/journal.pone.0319492)
Supplement: S6 Table — In this table, the average temperature increment during insertion for the AT protocol is presented. (PDF) [file pone.0319492.s006.pdf]

Protocol	AT					
Hole number	1	10	20	30	40	50
Time (s)	ΔT_avg (K)	ΔT_avg (K)	ΔT_avg (K)	ΔT_avg (K)	ΔT_avg (K)	ΔT_avg (K)
0.00	-0.0305	-0.0004	0.0004	0.0196	-0.0021	0.0090
0.25	-0.0305	0.0298	0.0005	0.0196	-0.0021	0.0090
0.50	0.0423	0.0053	0.0005	-0.0073	0.0106	0.0166
0.75	0.0423	0.0053	-0.0295	0.0211	0.0106	0.0166
1.00	-0.0008	-0.0027	-0.0258	0.0211	0.0049	-0.0128
1.25	-0.0237	-0.0027	-0.0258	-0.0044	0.0049	-0.0128
1.50	-0.0237	0.0393	0.0089	-0.0044	0.0025	0.0428
1.75	0.0169	-0.0300	0.0089	0.0067	-0.0153	-0.0268
2.00	0.0169	-0.0300	-0.0069	-0.0062	-0.0153	-0.0268
2.25	0.0047	-0.0086	-0.0354	-0.0062	0.0021	0.0079
2.50	0.0047	-0.0086	-0.0354	0.0023	0.0021	0.0079
2.75	-0.0089	-0.0017	0.0002	0.0023	0.0069	-0.0117
3.00	0.0241	-0.0017	0.0002	-0.0014	-0.0232	0.0234
3.25	0.0241	-0.0139	-0.0294	-0.0014	-0.0232	0.0234
3.50	0.0116	-0.0044	-0.0294	0.0004	-0.0083	-0.0105
3.75	0.0116	-0.0044	-0.0391	-0.0259	-0.0083	-0.0105
4.00	0.0260	0.0022	-0.0391	-0.0259	-0.0179	-0.0004
4.25	0.0235	0.0022	-0.0391	0.0032	-0.0179	-0.0004
4.50	0.0235	0.0073	-0.0169	0.0032	-0.0159	-0.0270
4.75	-0.0244	0.0135	-0.0169	0.0088	-0.0229	-0.0013
5.00	-0.0244	0.0135	-0.0473	0.0088	-0.0229	-0.0013
5.25	0.0227	-0.0177	-0.0473	-0.0129	-0.0044	-0.0323
5.50	0.0227	-0.0177	-0.0528	0.0172	-0.0044	-0.0323
5.75	0.0095	-0.0324	-0.0677	0.0172	-0.0268	-0.0342
6.00	-0.0053	-0.0324	-0.0677	-0.0302	-0.0268	-0.0330
6.25	-0.0053	-0.0338	-0.0284	-0.0302	-0.0621	-0.0330
6.50	0.0472	-0.0283	-0.0284	-0.0037	-0.0199	-0.0539
6.75	0.0472	-0.0283	-0.0027	-0.0216	-0.0199	-0.0539
7.00	0.0145	-0.0324	-0.0227	-0.0216	-0.0724	-0.0186
7.25	0.0145	-0.0324	-0.0227	-0.0085	-0.0724	-0.0186
7.50	0.0070	-0.0395	-0.0223	-0.0085	-0.0301	-0.0224
7.75	0.0277	-0.0323	-0.0223	-0.0297	-0.0170	-0.0152
8.00	0.0277	-0.0323	0.0050	-0.0297	-0.0170	-0.0152
8.25	0.0347	-0.0179	0.0050	-0.0180	-0.0179	-0.0131
8.50	0.0347	-0.0179	0.0094	-0.0211	-0.0179	-0.0131
8.75	0.0637	0.0098	-0.0015	-0.0211	-0.0272	0.0263
9.00	0.0545	0.0098	-0.0015	-0.0388	-0.0272	0.0189
9.25	0.0545	0.0113	0.0393	-0.0388	0.0256	0.0189
9.50	0.0607	-0.0318	0.0393	-0.0072	0.0344	-0.0029
9.75	0.0607	-0.0318	0.0400	0.0223	0.0344	-0.0029
10.00	0.0891	0.0515	0.0884	0.0223	0.0655	0.0624
10.25	0.0891	0.0515	0.0884	0.0508	0.0655	0.0624
10.50	0.1215	0.0445	0.0753	0.0508	0.0918	0.0607
10.75	0.1255	0.0445	0.0753	0.0611	0.0968	0.0978
11.00	0.1255	0.0490	0.0942	0.0611	0.0968	0.0978
11.25	0.1492	0.0638	0.0942	0.1028	0.0942	0.1186
11.50	0.1492	0.0638	0.1367	0.1264	0.1367	0.1186
11.75	0.1526	0.0561	0.1453	0.1264	0.1453	0.1387

12.00	0.1904	0.0561	0.1453	0.1509	0.1453	0.1387
12.25	0.1904	0.0464	0.1518	0.1509	0.1518	0.1315
12.50	0.2160	0.0762	0.1518	0.1850	0.1518	0.2063
12.75	0.2160	0.0762	0.1950	0.2222	0.1950	0.2063
13.00	0.2349	0.1158	0.2284	0.2222	0.2284	0.2195
13.25	0.2349	0.1158	0.2284	0.2450	0.2284	0.2195
13.50	0.2791	0.1403	0.2454	0.2450	0.2454	0.2590
13.75	0.2771	0.1403	0.2454	0.2375	0.2454	0.2875
14.00	0.2771	0.1424	0.2857	0.2375	0.2857	0.2875
14.25	0.3096	0.1813	0.2857	0.3306	0.2857	0.3322
14.50	0.3096	0.1813	0.2866	0.3513	0.2866	0.3322
14.75	0.3535	0.1866	0.3580	0.3513	0.3580	0.3606
15.00	0.4052	0.1866	0.3580	0.3752	0.3580	0.3606
15.25	0.4052	0.2087	0.3771	0.3752	0.3771	0.4369
15.50	0.3742	0.2176	0.3771	0.4190	0.3771	0.4480
15.75	0.3742	0.2176	0.3915	0.4190	0.3915	0.4480
16.00	0.4128	0.2976	0.3915	0.4636	0.3915	0.4665
16.25	0.4128	0.2976	0.4041	0.4757	0.4041	0.4665
16.50	0.4497	0.2918	0.4502	0.4757	0.4502	0.4782
16.75	0.4847	0.2918	0.4502	0.5367	0.4502	0.5314
17.00	0.4847	0.3345	0.4838	0.5367	0.4838	0.5314
17.25	0.5026	0.2998	0.4838	0.5263	0.4838	0.5650
17.50	0.5026	0.2998	0.5515	0.5573	0.5515	0.5650
17.75	0.5024	0.3234	0.5991	0.5573	0.5991	0.6077
18.00	0.5024	0.3234	0.5991	0.6351	0.5991	0.6077
18.25	0.5698	0.4067	0.6301	0.6351	0.6301	0.6687
18.50	0.6149	0.4226	0.6301	0.6913	0.6301	0.6958
18.75	0.6149	0.4226	0.6913	0.6913	0.6913	0.6958
19.00	0.6453	0.4122	0.6913	0.7165	0.6913	0.7732
19.25	0.6453	0.4122	0.7285	0.7759	0.7285	0.7732
19.50	0.7077	0.4750	0.7972	0.7759	0.7972	0.7963
19.75	0.7330	0.4750	0.7972	0.8303	0.7972	0.8841
20.00	0.7330	0.5168	0.8622	0.8303	0.8622	0.8841
20.25	0.7990	0.5149	0.8622	0.8514	0.8622	0.9341
20.50	0.7990	0.5149	0.8968	0.9270	0.8968	0.9341
20.75	0.8194	0.5925	0.9372	0.9270	0.9372	1.0142
21.00	0.8194	0.5925	0.9372	0.9770	0.9372	1.0142
21.25	0.8666	0.5980	1.0109	0.9770	1.0109	1.0676
21.50	0.9419	0.5980	1.0109	1.0344	1.0109	1.1915
21.75	0.9419	0.6598	1.0915	1.0344	1.0915	1.1915
22.00	0.9669	0.6542	1.0915	1.0860	1.0915	1.2315
22.25	0.9669	0.6542	1.1017	1.1665	1.1017	1.2315
22.50	1.0293	0.7316	1.2239	1.1665	1.2239	1.3087
22.75	1.0832	0.7316	1.2239	1.2660	1.2239	1.3087
23.00	1.0832	0.7800	1.2550	1.2660	1.2550	1.3664
23.25	1.1426	0.7946	1.2550	1.3498	1.2550	1.5069
23.50	1.1426	0.7946	1.3336	1.4034	1.3336	1.5069
23.75	1.2002	0.8344	1.4011	1.4034	1.4011	1.5607
24.00	1.2002	0.8344	1.4011	1.4748	1.4011	1.5607
24.25	1.2366	0.9222	1.4139	1.4748	1.4139	1.6263
24.50	1.2733	0.9222	1.4139	1.5522	1.4139	1.7048
24.75	1.2733	0.9342	1.4997	1.5522	1.4997	1.7048

25.00	1.2829	0.9873	1.4997	1.6196	1.4997	1.8026
25.25	1.2829	0.9873	1.5120	1.6784	1.5120	1.8026
25.50	1.3564	1.0177	1.5859	1.6784	1.5859	1.8483
25.75	1.4052	1.0177	1.5859	1.7561	1.5859	1.8483
26.00	1.4052	1.0575	1.5861	1.7561	1.5861	1.9054
26.25	1.4343	1.1269	1.5861	1.8087	1.5861	1.9870
26.50	1.4343	1.1269	1.6544	1.8087	1.6544	1.9870
26.75	1.4769	1.1332	1.6544	1.8683	1.6544	2.0432
27.00	1.4769	1.1332	1.6877	1.8853	1.6877	2.0432
27.25	1.4948	1.1570	1.7398	1.8853	1.7398	2.0678
27.50	1.5365	1.1570	1.7398	1.9700	1.7398	2.1042
27.75	1.5365	1.2139	1.7428	1.9700	1.7428	2.1042
28.00	1.5431	1.2478	1.7428	1.9697	1.7428	2.1576
28.25	1.5431	1.2478	1.7661	2.0438	1.7661	2.1576
28.50	1.5652	1.2856	1.8291	2.0438	1.8291	2.1860
28.75	1.5652	1.2856	1.8291	2.0393	1.8291	2.1860
29.00	1.6191	1.3308	1.7973	2.0393	1.7973	2.2324
29.25	1.6216	1.3545	1.7973	2.0857	1.7973	2.2706
29.50	1.6216	1.3545	1.8532	2.0857	1.8532	2.2706
29.75	1.6568	1.3800	1.8532	2.1199	1.8532	2.3063
30.00	1.6568	1.3800	1.9002	2.1698	1.9002	2.3063
30.25	1.7058	1.4241	1.9291	2.1698	1.9291	2.3537
30.50	1.7449	1.4241	1.9291	2.1687	1.9291	2.3858
30.75	1.7449	1.4351	1.9668	2.1687	1.9668	2.3858
31.00	1.7203	1.4590	1.9668	2.1939	1.9668	2.3929
31.25	1.7203	1.4590	1.9758	2.2174	1.9758	2.3929
31.50	1.7338	1.4649	1.9727	2.2174	1.9727	2.4637
31.75	1.7338	1.4649	1.9727	2.2670	1.9727	2.4637
32.00	1.7680	1.4791	2.0177	2.2670	2.0177	2.4590
32.25	1.7602	1.4791	2.0177	2.2770	2.0177	2.4829
32.50	1.7602	1.5337	1.9996	2.2770	1.9996	2.4829
32.75	1.8053	1.5178	1.9996	2.2931	1.9996	2.4565
33.00	1.8053	1.5178	2.0326	2.3257	2.0326	2.4565
33.25	1.7942	1.5533	2.0629	2.3257	2.0629	2.5195
33.50	1.8459	1.5533	2.0629	2.3354	2.0629	2.5195
33.75	1.8459	1.5271	2.0805	2.3354	2.0805	2.5179
34.00	1.8176	1.5940	2.0805	2.3664	2.0805	2.5142
34.25	1.8176	1.5940	2.0519	2.3339	2.0519	2.5142
34.50	1.8321	1.6216	2.0921	2.3339	2.0921	2.5919
34.75	1.8321	1.6216	2.0921	2.3156	2.0921	2.5919
35.00	1.8501	1.6305	2.1184	2.3156	2.1184	2.5735
35.25	1.8699	1.6305	2.1184	2.3934	2.1184	2.5920
35.50	1.8699	1.6395	2.1360	2.3934	2.1360	2.5920
35.75	1.8934	1.6240	2.1360	2.3752	2.1360	2.5704
36.00	1.8934	1.6240	2.1626	2.4072	2.1626	2.5704
36.25	1.8623	1.6450	2.1271	2.4072	2.1271	2.5833
36.50	1.9065	1.6450	2.1271	2.4114	2.1271	2.5833
36.75	1.9065	1.6692	2.1480	2.4114	2.1480	2.5985
37.00	1.9124	1.6741	2.1480	2.3934	2.1480	2.6103
37.25	1.9124	1.6741	2.1784	2.3934	2.1784	2.6103
37.50	1.8755	1.7173	2.1784	2.4431	2.1784	2.6644
37.75	1.8755	1.7173	2.1717	2.4288	2.1717	2.6644

38.00	1.9140	1.6908	2.1631	2.4288	2.1631	2.6327
38.25	1.9360	1.6908	2.1631	2.3888	2.1631	2.6472
38.50	1.9360	1.7117	2.1728	2.3888	2.1728	2.6472
38.75	1.9109	1.7294	2.1728	2.4084	2.1728	2.6670
39.00	1.9109	1.7294	2.2090	2.4186	2.2090	2.6670
39.25	1.9128	1.7211	2.2164	2.4186	2.2164	2.6614
39.50	1.9128	1.7211	2.2164	2.4351	2.2164	2.6614
39.75	1.9257	1.7344	2.1889	2.4351	2.1889	2.6961
40.00	1.9336	1.7475	2.1889	2.4472	2.1889	2.6458
40.25	1.9336	1.7475	2.2097	2.4472	2.2097	2.6458
40.50	1.9271	1.7598	2.2097	2.4434	2.2097	2.6676
40.75	1.9271	1.7598	2.2250	2.4169	2.2250	2.6676
41.00	1.9263	1.7466	2.2172	2.4169	2.2172	2.6642
41.25	1.9566	1.7466	2.2172	2.4363	2.2172	2.6697
41.50	1.9566	1.7761	2.2356	2.4363	2.2356	2.6697
41.75	1.9879	1.7824	2.2356	2.4357	2.2356	2.6623
42.00	1.9879	1.7824	2.2095	2.4453	2.2095	2.6623
42.25	1.9670	1.7779	2.1770	2.4453	2.1770	2.6326
42.50	1.9670	1.7779	2.1770	2.4447	2.1770	2.6326
42.75	1.9427	1.8097	2.2305	2.4447	2.2305	2.6557
43.00	1.9556	1.8097	2.2305	2.4423	2.2305	2.6460
43.25	1.9556	1.8042	2.2239	2.4423	2.2239	2.6460
43.50	1.9569	1.8019	2.2239	2.4171	2.2239	2.6551
43.75	1.9569	1.8019	2.2313	2.4586	2.2313	2.6551
44.00	1.9499	1.7948	2.2049	2.4586	2.2049	2.6833
44.25	1.9576	1.7948	2.2049	2.4377	2.2049	2.6833
44.50	1.9576	1.8073	2.2081	2.4377	2.2081	2.6473
44.75	1.9428	1.8101	2.2081	2.4588	2.2081	2.6497
45.00	1.9428	1.8101	2.1826	2.4283	2.1826	2.6497
45.25	1.9587	1.7999	2.1742	2.4283	2.1742	2.6432
45.50	1.9587	1.7999	2.1742	2.4166	2.1742	2.6432
45.75	1.9591	1.8085	2.2248	2.4166	2.2248	2.6846
46.00	1.9649	1.8085	2.2248	2.4220	2.2248	2.6566
46.25	1.9649	1.8118	2.2072	2.4220	2.2072	2.6566
46.50	1.9546	1.8178	2.2072	2.4154	2.2072	2.6541
46.75	1.9546	1.8178	2.2135	2.4050	2.2135	2.6541
47.00	1.9677	1.8140	2.2163	2.4050	2.2163	2.6338
47.25	1.9848	1.8140	2.2163	2.3654	2.2163	2.6338
47.50	1.9848	1.8166	2.1819	2.3654	2.1819	2.6581
47.75	1.9263	1.8054	2.1819	2.3869	2.1819	2.6298
48.00	1.9263	1.8054	2.1907	2.3869	2.1907	2.6298
48.25	1.9408	1.8046	2.1907	2.4004	2.1907	2.6659
48.50	1.9408	1.8046	2.1586	2.3733	2.1586	2.6659
48.75	1.9110	1.7928	2.1932	2.3733	2.1932	2.6240
49.00	1.9416	1.7928	2.1932	2.4165	2.1932	2.6204
49.25	1.9416	1.8124	2.2068	2.4165	2.2068	2.6204
49.50	1.9467	1.8177	2.2068	2.3815	2.2068	2.6453
49.75	1.9467	1.8177	2.2017	2.3932	2.2017	2.6453
50.00	1.9636	1.7932	2.1579	2.3932	2.1579	2.6069
50.25	1.9636	1.7932	2.1579	2.3669	2.1579	2.6069
50.50	1.9606	1.8205	2.1552	2.3669	2.1552	2.6305
50.75	1.9404	1.8263	2.1552	2.3649	2.1552	2.6380

51.00	1.9404	1.8263	2.1883	2.3649	2.1883	2.6380
51.25	1.9398	1.8181	2.1883	2.3788	2.1883	2.6032
51.50	1.9398	1.8181	2.1718	2.3797	2.1718	2.6032
51.75	1.9517	1.8213	2.1494	2.3797	2.1494	2.5935
52.00	1.9453	1.8213	2.1494	2.3832	2.1494	2.6020
52.25	1.9453	1.7988	2.1679	2.3832	2.1679	2.6020
52.50	1.9225	1.7884	2.1679	2.3594	2.1679	2.6026
52.75	1.9225	1.7884	2.2042	2.3728	2.2042	2.6026
53.00	1.9179	1.8047	2.1491	2.3728	2.1491	2.5616
53.25	1.9179	1.8047	2.1491	2.3307	2.1491	2.5616
53.50	1.9140	1.7913	2.1545	2.3307	2.1545	2.5962
53.75	1.9447	1.7913	2.1545	2.3383	2.1545	2.5701
54.00	1.9447	1.7869	2.1249	2.3383	2.1249	2.5701
54.25	1.9301	1.7816	2.1249	2.3181	2.1249	2.5669
54.50	1.9301	1.7816	2.1413	2.3317	2.1413	2.5669
54.75	1.9082	1.7960	2.1181	2.3317	2.1181	2.5410
55.00	1.9143	1.7960	2.1181	2.2830	2.1181	2.5410
55.25	1.9143	1.7996	2.1254	2.2830	2.1254	2.5525
55.50	1.9229	1.7893	2.1254	2.3144	2.1254	2.5663
55.75	1.9229	1.7893	2.1269	2.3298	2.1269	2.5663
56.00	1.9226	1.8118	2.1438	2.3298	2.1438	2.5486
56.25	1.9226	1.8118	2.1438	2.3356	2.1438	2.5486
56.50	1.8959	1.7782	2.1166	2.3356	2.1166	2.5204
56.75	1.8918	1.7782	2.1166	2.2873	2.1166	2.5214
57.00	1.8918	1.7837	2.0979	2.2873	2.0979	2.5214
57.25	1.8648	1.7795	2.0979	2.2609	2.0979	2.5220
57.50	1.8648	1.7795	2.0957	2.2803	2.0957	2.5220
57.75	1.8924	1.8070	2.1077	2.2803	2.1077	2.4926
58.00	1.8632	1.8070	2.1077	2.2872	2.1077	2.4926
58.25	1.8632	1.7921	2.0597	2.2872	2.0597	2.4999
58.50	1.8943	1.7790	2.0597	2.2677	2.0597	2.4843
58.75	1.8943	1.7790	2.0854	2.2677	2.0854	2.4843
59.00	1.8837	1.7534	2.0854	2.2661	2.0854	2.4816
59.25	1.8837	1.7534	2.0673	2.2533	2.0673	2.4816
59.50	1.8771	1.7569	2.0745	2.2533	2.0745	2.4799
59.75	1.8519	1.7569	2.0745	2.2230	2.0745	2.4574
60.00	1.8519	1.7605	2.0654	2.2230	2.0654	2.4574
60.25	1.8667	1.7442	2.0654	2.2177	2.0654	2.4378
60.50	1.8667	1.7442	2.0591	2.1958	2.0591	2.4378
60.75	1.8463	1.7384	2.0607	2.1958	2.0607	2.4479
61.00	1.8463	1.7384	2.0607	2.2270	2.0607	2.4479
61.25	1.8682	1.7307	2.0673	2.2270	2.0673	2.4223
61.50	1.8387	1.7420	2.0673	2.2192	2.0673	2.4488
61.75	1.8387	1.7420	2.0308	2.2192	2.0308	2.4488
62.00	1.8309	1.7242	2.0308	2.2017	2.0308	2.4372
62.25	1.8309	1.7242	2.0275	2.1672	2.0275	2.4372
62.50	1.8454	1.7310	2.0244	2.1672	2.0244	2.4223
62.75	1.8593	1.7310	2.0244	2.2267	2.0244	2.3886
63.00	1.8593	1.7430	2.0257	2.2267	2.0257	2.3886
63.25	1.8453	1.7644	2.0257	2.1527	2.0257	2.3946
63.50	1.8453	1.7644	2.0635	2.1787	2.0635	2.3946
63.75	1.7973	1.7391	2.0215	2.1787	2.0215	2.3921

64.00	1.7973	1.7391	2.0215	2.1311	2.0215	2.3921
64.25	1.8102	1.7173	1.9868	2.1311	1.9868	2.3592
64.50	1.8221	1.7173	1.9868	2.1677	1.9868	2.4071
64.75	1.8221	1.7192	1.9961	2.1677	1.9961	2.4071
65.00	1.8289	1.6978	1.9961	2.1328	1.9961	2.3708
65.25	1.8289	1.6978	1.9832	2.1471	1.9832	2.3708
65.50	1.7905	1.7050	1.9961	2.1471	1.9961	2.3508
65.75	1.8056	1.7050	1.9961	2.1332	1.9961	2.3508
66.00	1.8056	1.6798	1.9694	2.1332	1.9694	2.3592
66.25	1.7775	1.6900	1.9694	2.1271	1.9694	2.3631
66.50	1.7775	1.6900	1.9856	2.1239	1.9856	2.3631
66.75	1.8178	1.7092	1.9734	2.1239	1.9734	2.3307
67.00	1.8178	1.7092	1.9734	2.1192	1.9734	2.3307
67.25	1.8017	1.6630	1.9765	2.1192	1.9765	2.3083
67.50	1.7776	1.6630	1.9765	2.0822	1.9765	2.3210
67.75	1.7776	1.6673	1.9527	2.0822	1.9527	2.3210
68.00	1.7940	1.6481	1.9527	2.0802	1.9527	2.2902
68.25	1.7940	1.6481	1.9409	2.0719	1.9409	2.2902
68.50	1.7924	1.6779	1.9491	2.0719	1.9491	2.2912
68.75	1.8141	1.6779	1.9491	2.0568	1.9491	2.2912
69.00	1.8141	1.6583	1.9060	2.0568	1.9060	2.2835
69.25	1.7901	1.6533	1.9060	2.0353	1.9060	2.2707
69.50	1.7901	1.6533	1.9137	2.0353	1.9137	2.2707
69.75	1.7644	1.6663	1.9137	2.0727	1.9137	2.2351
70.00	1.7644	1.6663	1.9069	2.0342	1.9069	2.2351
70.25	1.7341	1.6089	1.9268	2.0342	1.9268	2.2396
70.50	1.7910	1.6089	1.9268	2.0302	1.9268	2.2410
70.75	1.7910	1.6558	1.9274	2.0302	1.9274	2.2410
71.00	1.7626	1.6213	1.9274	2.0075	1.9274	2.2520
71.25	1.7626	1.6213	1.9005	2.0202	1.9005	2.2520
71.50	1.7287	1.6073	1.8842	2.0202	1.8842	2.2099
71.75	1.7287	1.6073	1.8842	2.0130	1.8842	2.2099
72.00	1.7631	1.6255	1.8864	2.0130	1.8864	2.1998
72.25	1.7430	1.6310	1.8864	2.0108	1.8864	2.2106
72.50	1.7430	1.6310	1.8625	2.0108	1.8625	2.2106
72.75	1.7667	1.6259	1.8625	2.0212	1.8625	2.1541
73.00	1.7667	1.6259	1.8469	1.9982	1.8469	2.1541
73.25	1.7229	1.6120	1.8446	1.9982	1.8446	2.1595
73.50	1.7314	1.6120	1.8446	1.9941	1.8446	2.1689
73.75	1.7314	1.6130	1.8468	1.9941	1.8468	2.1689
74.00	1.7122	1.6227	1.8468	1.9647	1.8468	2.1476
74.25	1.7122	1.6227	1.8323	1.9572	1.8323	2.1476
74.50	1.7024	1.6256	1.8321	1.9572	1.8321	2.1142
74.75	1.7024	1.6256	1.8321	1.9464	1.8321	2.1142
75.00	1.7143	1.6214	1.8041	1.9464	1.8041	2.1389
75.25	1.7214	1.6214	1.8041	1.9389	1.8041	2.1118
75.50	1.7214	1.6064	1.8405	1.9389	1.8405	2.1118
75.75	1.7007	1.5680	1.8405	1.9457	1.8405	2.1119
76.00	1.7007	1.5680	1.8272	1.9193	1.8272	2.1119
76.25	1.6807	1.5687	1.8023	1.9193	1.8023	2.0954
76.50	1.7071	1.5687	1.8023	1.9386	1.8023	2.0954
76.75	1.7071	1.5879	1.7984	1.9386	1.7984	2.0658

77.00	1.6934	1.5787	1.7984	1.8917	1.7984	2.0699
77.25	1.6934	1.5787	1.7840	1.8853	1.7840	2.0699
77.50	1.6800	1.5342	1.7864	1.8853	1.7864	2.0852
77.75	1.6800	1.5342	1.7864	1.8947	1.7864	2.0852
78.00	1.6636	1.5814	1.7857	1.8947	1.7857	2.0560
78.25	1.6718	1.5814	1.7857	1.8519	1.7857	2.0721
78.50	1.6718	1.5550	1.7751	1.8519	1.7751	2.0721
78.75	1.6501	1.5540	1.7751	1.8789	1.7751	2.0306
79.00	1.6501	1.5540	1.7571	1.8665	1.7571	2.0306
79.25	1.6660	1.5498	1.7700	1.8665	1.7700	2.0376
79.50	1.6702	1.5498	1.7700	1.8524	1.7700	2.0376
79.75	1.6702	1.5608	1.7360	1.8524	1.7360	2.0433
80.00	1.6689	1.5217	1.7360	1.8495	1.7360	2.0319
80.25	1.6689	1.5217	1.7098	1.8495	1.7098	2.0319
80.50	1.6073	1.5495	1.7098	1.8371	1.7098	2.0236
80.75	1.6073	1.5495	1.7160	1.8429	1.7160	2.0236
81.00	1.6296	1.5348	1.7151	1.8429	1.7151	2.0107
81.25	1.6547	1.5348	1.7151	1.8318	1.7151	1.9773
81.50	1.6547	1.5424	1.7457	1.8318	1.7457	1.9773
81.75	1.6288	1.4858	1.7457	1.8146	1.7457	1.9815
82.00	1.6288	1.4858	1.7253	1.8199	1.7253	1.9815
82.25	1.6388	1.5253	1.7207	1.8199	1.7207	1.9849
82.50	1.6388	1.5253	1.7207	1.8113	1.7207	1.9849
82.75	1.6088	1.5008	1.7114	1.8113	1.7114	2.0143
83.00	1.6400	1.4794	1.7114	1.7486	1.7114	2.0132
83.25	1.6400	1.4794	1.6822	1.7486	1.6822	2.0132
83.50	1.6128	1.5131	1.6822	1.7765	1.6822	1.9389
83.75	1.6128	1.5131	1.6960	1.7781	1.6960	1.9389
84.00	1.5921	1.4628	1.6957	1.7781	1.6957	1.9215
84.25	1.5946	1.4628	1.6957	1.7725	1.6957	1.9435
84.50	1.5946	1.4788	1.6765	1.7725	1.6765	1.9435
84.75	1.5755	1.4778	1.6765	1.7549	1.6765	1.8952
85.00	1.5755	1.4778	1.6897	1.7521	1.6897	1.8952
85.25	1.5768	1.5064	1.6677	1.7521	1.6677	1.9815
85.50	1.5768	1.5064	1.6677	1.7194	1.6677	1.9815
85.75	1.5604	1.4680	1.6597	1.7194	1.6597	1.9075
86.00	1.5538	1.4680	1.6597	1.7635	1.6597	1.8978
86.25	1.5538	1.4724	1.6686	1.7635	1.6686	1.8978
86.50	1.5713	1.4658	1.6686	1.7288	1.6686	1.8663
86.75	1.5713	1.4658	1.6558	1.7028	1.6558	1.8663
87.00	1.5802	1.4557	1.6422	1.7028	1.6422	1.8945
87.25	1.5620	1.4557	1.6422	1.6660	1.6422	1.8945
87.50	1.5620	1.4204	1.6198	1.6660	1.6198	1.8717
87.75	1.5297	1.4586	1.6198	1.7124	1.6198	1.8605
88.00	1.5297	1.4586	1.6369	1.7023	1.6369	1.8605
88.25	1.5733	1.4426	1.6279	1.7023	1.6279	1.8610
88.50	1.5733	1.4426	1.6279	1.6726	1.6279	1.8610
88.75	1.5408	1.4288	1.6046	1.6726	1.6046	1.8369
89.00	1.5118	1.4288	1.6046	1.6752	1.6046	1.8410
89.25	1.5118	1.4466	1.6202	1.6752	1.6202	1.8410
89.50	1.5602	1.4189	1.6202	1.6478	1.6202	1.8328
89.75	1.5602	1.4189	1.5826	1.6435	1.5826	1.8328

90.00	1.5520	1.4128	1.5782	1.6435	1.5782	1.7997
90.25	1.5078	1.4128	1.5782	1.6465	1.5782	1.7997
90.50	1.5078	1.4516	1.5879	1.6465	1.5879	1.8004
90.75	1.5225	1.3909	1.5879	1.6416	1.5879	1.8082
91.00	1.5225	1.3909	1.5677	1.6416	1.5677	1.8082
91.25	1.5237	1.3792	1.5677	1.6296	1.5677	1.8103
91.50	1.5237	1.3792	1.5653	1.6201	1.5653	1.8103
91.75	1.5078	1.3961	1.5503	1.6201	1.5503	1.7529
92.00	1.5136	1.3961	1.5503	1.6539	1.5503	1.8005
92.25	1.5136	1.4123	1.5495	1.6539	1.5495	1.8005
92.50	1.4907	1.3938	1.5495	1.5798	1.5495	1.7734
92.75	1.4907	1.3938	1.5340	1.6106	1.5340	1.7734
93.00	1.4997	1.3871	1.5181	1.6106	1.5181	1.7498
93.25	1.4997	1.3871	1.5181	1.5820	1.5181	1.7498
93.50	1.5164	1.3709	1.5469	1.5820	1.5469	1.7195
93.75	1.5091	1.3661	1.5469	1.6008	1.5469	1.7705
94.00	1.5091	1.3661	1.5751	1.6008	1.5751	1.7705
94.25	1.4676	1.3653	1.5751	1.5988	1.5751	1.7545
94.50	1.4676	1.3653	1.5128	1.5946	1.5128	1.7545
94.75	1.4750	1.3437	1.5327	1.5946	1.5327	1.7208
95.00	1.5095	1.3437	1.5327	1.6150	1.5327	1.7541
95.25	1.5095	1.3684	1.5093	1.6150	1.5093	1.7541
95.50	1.4631	1.3643	1.5093	1.5841	1.5093	1.7098
95.75	1.4631	1.3643	1.5439	1.5396	1.5439	1.7098
96.00	1.4570	1.3304	1.4982	1.5396	1.4982	1.7192
96.25	1.4570	1.3304	1.4982	1.5755	1.4982	1.7192
96.50	1.4800	1.3383	1.4945	1.5755	1.4945	1.7109
96.75	1.4455	1.3383	1.4945	1.5206	1.4945	1.6542
97.00	1.4455	1.3592	1.4921	1.5206	1.4921	1.6542
97.25	1.4478	1.3146	1.4921	1.5245	1.4921	1.6796
97.50	1.4478	1.3146	1.4916	1.5252	1.4916	1.6796
97.75	1.4575	1.3192	1.4782	1.5252	1.4782	1.6372
98.00	1.4696	1.3192	1.4782	1.5419	1.4782	1.6372
98.25	1.4696	1.3425	1.4568	1.5419	1.4568	1.6626
98.50	1.4561	1.3077	1.4568	1.5118	1.4568	1.6822
98.75	1.4561	1.3077	1.4557	1.5211	1.4557	1.6822
99.00	1.4455	1.3177	1.4507	1.5211	1.4507	1.6224
99.25	1.4455	1.3177	1.4507	1.5175	1.4507	1.6224
99.50	1.3855	1.3244	1.4579	1.5175	1.4579	1.6536
99.75	1.4385	1.3244	1.4579	1.4994	1.4579	1.6136
100.00	1.4385	1.2974	1.4108	1.4994	1.4108	1.6136
100.25	1.4193	1.2743	1.4108	1.4897	1.4108	1.6341
100.50	1.4193	1.2743	1.4489	1.4837	1.4489	1.6341
100.75	1.4332	1.2789	1.4370	1.4837	1.4370	1.6294
101.00	1.4300	1.2789	1.4370	1.4711	1.4370	1.6294
101.25	1.4300	1.2940	1.4391	1.4711	1.4391	1.5980
101.50	1.3925	1.3178	1.4391	1.4782	1.4391	1.5969
101.75	1.3925	1.3178	1.4281	1.4782	1.4281	1.5969
102.00	1.4243	1.2698	1.4281	1.4618	1.4281	1.5813
102.25	1.4243	1.2698	1.4377	1.4576	1.4377	1.5813
102.50	1.4065	1.3071	1.4071	1.4576	1.4071	1.5477
102.75	1.4213	1.3071	1.4071	1.4728	1.4071	1.5785

103.00	1.4213	1.2630	1.3863	1.4728	1.3863	1.5785
103.25	1.3918	1.2563	1.3863	1.4551	1.3863	1.5802
103.50	1.3918	1.2563	1.3830	1.4584	1.3830	1.5802
103.75	1.4180	1.2437	1.3961	1.4584	1.3961	1.5648
104.00	1.4180	1.2437	1.3961	1.4082	1.3961	1.5648
104.25	1.3840	1.2525	1.3724	1.4082	1.3724	1.5766
104.50	1.3714	1.2544	1.3724	1.4303	1.3724	1.5614
104.75	1.3714	1.2544	1.4064	1.4303	1.4064	1.5614
105.00	1.3842	1.2129	1.4064	1.4386	1.4064	1.5219
105.25	1.3842	1.2129	1.3815	1.4408	1.3815	1.5219
105.50	1.3625	1.2126	1.3766	1.4408	1.3766	1.5255
105.75	1.3608	1.2126	1.3766	1.4337	1.3766	1.5470
106.00	1.3608	1.2509	1.3678	1.4337	1.3678	1.5470
106.25	1.3768	1.2559	1.3678	1.3986	1.3678	1.5488
106.50	1.3768	1.2559	1.3149	1.3631	1.3149	1.5488
106.75	1.3699	1.2526	1.3410	1.3631	1.3410	1.5143
107.00	1.3699	1.2526	1.3410	1.3909	1.3410	1.5143
107.25	1.3601	1.2450	1.3475	1.3909	1.3475	1.4917
107.50	1.3599	1.2450	1.3475	1.3847	1.3475	1.4863
107.75	1.3599	1.2087	1.3146	1.3847	1.3146	1.4863
108.00	1.3438	1.2355	1.3146	1.3864	1.3146	1.4938
108.25	1.3438	1.2355	1.3564	1.3500	1.3564	1.4938
108.50	1.3399	1.2286	1.3297	1.3500	1.3297	1.5141
108.75	1.3190	1.2286	1.3297	1.3628	1.3297	1.5141
109.00	1.3190	1.2393	1.3393	1.3628	1.3393	1.4806
109.25	1.3435	1.2076	1.3393	1.3980	1.3393	1.4871
109.50	1.3435	1.2076	1.3126	1.3661	1.3126	1.4871
109.75	1.3264	1.1843	1.3559	1.3661	1.3559	1.4963
110.00	1.3264	1.1843	1.3559	1.3397	1.3559	1.4963
110.25	1.3011	1.1828	1.3107	1.3397	1.3107	1.4876
110.50	1.3703	1.1828	1.3107	1.3314	1.3107	1.4687
110.75	1.3703	1.1784	1.3128	1.3314	1.3128	1.4687
111.00	1.3209	1.1695	1.3128	1.3352	1.3128	1.4594
111.25	1.3209	1.1695	1.2843	1.3450	1.2843	1.4594
111.50	1.3204	1.1964	1.2693	1.3450	1.2693	1.4303
111.75	1.3069	1.1964	1.2693	1.3401	1.2693	1.4303
112.00	1.3069	1.1492	1.2785	1.3401	1.2785	1.4250
112.25	1.3309	1.1941	1.2785	1.2940	1.2785	1.4136
112.50	1.3309	1.1941	1.2812	1.2940	1.2812	1.4136
112.75	1.3040	1.1612	1.2812	1.3408	1.2812	1.4345
113.00	1.3040	1.1612	1.2687	1.3267	1.2687	1.4345
113.25	1.3080	1.1590	1.2802	1.3267	1.2802	1.4332
