# Supplementary material for: Impact of drill bit wear on temperature increase in dental implant osteotomy: an in vitro study
Source: PLoS One. 2025 Mar 19;20(3):e0319492. doi: 10.1371/journal.pone.0319492 (PMC11922234; doi:10.1371/journal.pone.0319492)
Supplement: S7 Table — In this table, the average temperature increment during insertion for the PT protocol is presented. (PDF) [file pone.0319492.s007.pdf]

Protocol	PT					
Hole number	1	10	20	30	40	50
Time (s)	ΔT_avg (K)	ΔT_avg (K)	ΔT_avg (K)	ΔT_avg (K)	ΔT_avg (K)	ΔT_avg (K)
0.00	-0.013	0.019	-0.013	-0.014	0.001	-0.025
0.25	-0.020	0.032	-0.013	0.017	0.001	-0.025
0.50	-0.032	0.032	-0.016	-0.024	-0.002	-0.029
0.75	-0.032	-0.005	0.000	-0.024	-0.002	-0.029
1.00	-0.008	-0.005	0.000	-0.024	-0.004	-0.070
1.25	-0.008	0.002	-0.015	-0.024	-0.008	-0.070
1.50	-0.017	0.002	-0.015	-0.009	-0.008	-0.071
1.75	-0.017	-0.020	0.004	-0.009	-0.018	-0.054
2.00	0.001	-0.023	0.004	-0.036	-0.018	-0.054
2.25	-0.017	-0.023	-0.023	-0.014	0.002	-0.043
2.50	-0.017	-0.008	0.003	-0.014	-0.036	-0.043
2.75	-0.003	-0.008	0.003	-0.033	-0.036	-0.070
3.00	-0.003	-0.001	-0.007	-0.033	-0.027	-0.070
3.25	-0.036	-0.017	-0.007	-0.008	-0.027	-0.048
3.50	-0.020	-0.017	-0.049	-0.031	-0.005	-0.026
3.75	-0.020	-0.035	-0.015	-0.031	-0.005	-0.026
4.00	-0.008	-0.035	-0.015	-0.057	-0.041	-0.031
4.25	-0.008	-0.016	-0.035	-0.057	-0.010	-0.031
4.50	0.003	-0.016	-0.035	-0.037	-0.010	-0.069
4.75	0.003	-0.022	-0.026	-0.037	-0.050	-0.065
5.00	0.004	-0.040	-0.026	-0.029	-0.050	-0.065
5.25	0.020	-0.040	-0.037	-0.038	-0.018	-0.040
5.50	0.020	-0.048	-0.029	-0.038	-0.018	-0.040
5.75	0.011	-0.048	-0.029	-0.028	-0.018	-0.042
6.00	0.011	-0.045	0.000	-0.028	-0.003	-0.042
6.25	0.012	-0.017	0.000	-0.045	-0.003	-0.037
6.50	0.012	-0.017	-0.027	-0.018	-0.019	-0.056
6.75	0.010	-0.026	-0.001	-0.018	-0.019	-0.056
7.00	0.028	-0.026	-0.001	-0.015	-0.026	-0.044
7.25	0.028	-0.022	-0.016	-0.015	-0.032	-0.044
7.50	0.022	-0.022	-0.016	-0.048	-0.032	-0.028
7.75	0.022	-0.049	-0.021	-0.048	-0.045	-0.034
8.00	0.006	-0.063	-0.021	-0.015	-0.045	-0.034
8.25	0.033	-0.063	0.007	-0.040	-0.019	-0.056
8.50	0.033	-0.073	-0.019	-0.040	-0.019	-0.056
8.75	0.031	-0.073	-0.019	-0.009	-0.018	-0.058
9.00	0.031	-0.054	0.030	-0.009	-0.010	-0.058
9.25	0.024	-0.054	0.030	0.020	-0.010	0.018
9.50	0.024	-0.013	0.042	0.020	0.043	0.000
9.75	0.044	0.017	0.042	0.051	0.043	0.000
10.00	0.079	0.017	0.086	0.093	0.071	0.034
10.25	0.079	0.097	0.184	0.093	0.156	0.034
10.50	0.104	0.097	0.184	0.147	0.156	0.107
10.75	0.104	0.152	0.217	0.147	0.169	0.146
11.00	0.139	0.295	0.217	0.227	0.169	0.146
11.25	0.228	0.295	0.301	0.273	0.293	0.241
11.50	0.228	0.389	0.392	0.273	0.293	0.241
11.75	0.301	0.389	0.392	0.390	0.404	0.305

12.00	0.301	0.548	0.480	0.390	0.528	0.305
12.25	0.422	0.548	0.480	0.457	0.528	0.388
12.50	0.422	0.694	0.594	0.457	0.651	0.478
12.75	0.527	0.865	0.594	0.583	0.651	0.478
13.00	0.719	0.865	0.693	0.652	0.763	0.562
13.25	0.719	0.991	0.803	0.652	0.861	0.562
13.50	0.823	0.991	0.803	0.803	0.861	0.672
13.75	0.823	1.130	0.967	0.803	0.957	0.672
14.00	0.948	1.244	0.967	0.896	0.957	0.790
14.25	1.064	1.244	1.050	1.020	1.105	0.836
14.50	1.064	1.382	1.148	1.020	1.105	0.836
14.75	1.168	1.382	1.148	1.158	1.186	0.925
15.00	1.168	1.553	1.247	1.158	1.312	0.925
15.25	1.271	1.553	1.247	1.275	1.312	1.024
15.50	1.271	1.612	1.360	1.275	1.383	1.105
15.75	1.357	1.745	1.360	1.391	1.383	1.105
16.00	1.484	1.745	1.483	1.496	1.508	1.206
16.25	1.484	1.870	1.542	1.496	1.588	1.206
16.50	1.531	1.870	1.542	1.603	1.588	1.257
16.75	1.531	1.964	1.619	1.603	1.697	1.257
17.00	1.664	2.041	1.619	1.672	1.697	1.349
17.25	1.664	2.041	1.727	1.780	1.734	1.438
17.50	1.745	2.149	1.784	1.780	1.734	1.438
17.75	1.845	2.149	1.784	1.858	1.852	1.533
18.00	1.845	2.193	1.856	1.858	1.896	1.533
18.25	1.872	2.193	1.856	1.937	1.896	1.546
18.50	1.872	2.278	1.913	1.937	2.007	1.613
18.75	1.962	2.342	1.913	1.967	2.007	1.613
19.00	2.011	2.342	1.985	2.031	2.057	1.658
19.25	2.011	2.419	2.025	2.031	2.057	1.658
19.50	2.065	2.419	2.025	2.099	2.112	1.716
19.75	2.065	2.487	2.090	2.099	2.181	1.716
20.00	2.129	2.487	2.090	2.160	2.181	1.782
20.25	2.129	2.570	2.123	2.160	2.227	1.837
20.50	2.195	2.579	2.123	2.217	2.227	1.837
20.75	2.251	2.579	2.154	2.244	2.264	1.910
21.00	2.251	2.597	2.217	2.244	2.345	1.910
21.25	2.258	2.597	2.217	2.327	2.345	1.929
21.50	2.258	2.671	2.250	2.327	2.350	1.978
21.75	2.320	2.729	2.250	2.346	2.350	1.978
22.00	2.328	2.729	2.268	2.402	2.410	2.010
22.25	2.328	2.732	2.301	2.402	2.410	2.010
22.50	2.379	2.732	2.301	2.464	2.425	2.056
22.75	2.379	2.804	2.351	2.464	2.453	2.056
23.00	2.408	2.804	2.351	2.470	2.453	2.104
23.25	2.408	2.784	2.391	2.470	2.471	2.126
23.50	2.433	2.853	2.391	2.494	2.471	2.126
23.75	2.453	2.853	2.396	2.574	2.548	2.177
24.00	2.453	2.849	2.401	2.574	2.567	2.177
24.25	2.477	2.849	2.401	2.566	2.567	2.178
24.50	2.477	2.897	2.458	2.566	2.510	2.178
24.75	2.505	2.867	2.458	2.593	2.510	2.219

25.00	2.505	2.867	2.502	2.586	2.614	2.240
25.25	2.505	2.949	2.493	2.586	2.614	2.240
25.50	2.551	2.949	2.493	2.645	2.597	2.248
25.75	2.551	2.950	2.490	2.645	2.666	2.248
26.00	2.562	2.950	2.490	2.662	2.666	2.286
26.25	2.562	2.981	2.537	2.662	2.658	2.301
26.50	2.580	2.997	2.537	2.661	2.658	2.301
26.75	2.604	2.997	2.580	2.693	2.671	2.310
27.00	2.604	2.991	2.562	2.693	2.686	2.310
27.25	2.616	2.991	2.562	2.716	2.686	2.359
27.50	2.616	3.014	2.556	2.716	2.717	2.359
27.75	2.654	3.013	2.556	2.708	2.717	2.372
28.00	2.654	3.013	2.580	2.744	2.732	2.346
28.25	2.604	3.026	2.638	2.744	2.732	2.346
28.50	2.654	3.026	2.638	2.757	2.744	2.389
28.75	2.654	3.055	2.625	2.757	2.745	2.389
29.00	2.606	3.055	2.625	2.724	2.745	2.420
29.25	2.606	3.047	2.595	2.724	2.731	2.408
29.50	2.696	3.032	2.595	2.778	2.731	2.408
29.75	2.673	3.032	2.627	2.751	2.766	2.404
30.00	2.673	3.081	2.628	2.751	2.766	2.404
30.25	2.689	3.081	2.628	2.783	2.769	2.389
30.50	2.689	3.078	2.617	2.783	2.761	2.389
30.75	2.676	3.078	2.617	2.761	2.761	2.402
31.00	2.676	3.095	2.623	2.761	2.767	2.419
31.25	2.712	3.105	2.623	2.769	2.767	2.419
31.50	2.688	3.105	2.643	2.792	2.785	2.444
31.75	2.688	3.060	2.656	2.792	2.764	2.444
32.00	2.715	3.060	2.656	2.791	2.764	2.438
32.25	2.715	3.078	2.634	2.791	2.784	2.429
32.50	2.672	3.060	2.634	2.793	2.784	2.429
32.75	2.691	3.060	2.649	2.771	2.798	2.460
33.00	2.691	3.077	2.652	2.771	2.798	2.460
33.25	2.694	3.077	2.652	2.812	2.807	2.446
33.50	2.694	3.053	2.659	2.812	2.799	2.446
33.75	2.676	3.053	2.659	2.796	2.799	2.444
34.00	2.676	3.079	2.669	2.796	2.767	2.468
34.25	2.723	3.056	2.669	2.800	2.767	2.468
34.50	2.697	3.056	2.650	2.808	2.801	2.462
34.75	2.697	3.053	2.646	2.808	2.813	2.462
35.00	2.710	3.053	2.646	2.807	2.813	2.492
35.25	2.710	3.050	2.659	2.807	2.775	2.492
35.50	2.677	3.072	2.659	2.780	2.775	2.461
35.75	2.660	3.072	2.660	2.776	2.810	2.481
36.00	2.660	3.033	2.630	2.776	2.810	2.481
36.25	2.690	3.033	2.630	2.788	2.809	2.482
36.50	2.690	3.060	2.591	2.788	2.772	2.482
36.75	2.678	3.060	2.591	2.779	2.772	2.489
37.00	2.678	3.045	2.618	2.779	2.773	2.481
37.25	2.696	3.037	2.618	2.803	2.773	2.481
37.50	2.702	3.037	2.633	2.777	2.794	2.484
37.75	2.702	3.023	2.644	2.777	2.827	2.484

38.00	2.665	3.023	2.644	2.740	2.827	2.463
38.25	2.665	3.037	2.623	2.740	2.745	2.463
38.50	2.665	3.017	2.623	2.773	2.745	2.483
38.75	2.665	3.017	2.609	2.750	2.757	2.468
39.00	2.644	3.001	2.609	2.750	2.757	2.468
39.25	2.649	3.001	2.609	2.777	2.755	2.455
39.50	2.649	3.006	2.589	2.777	2.731	2.455
39.75	2.652	3.006	2.589	2.754	2.731	2.435
40.00	2.652	2.989	2.562	2.754	2.772	2.440
40.25	2.633	2.967	2.562	2.748	2.772	2.440
40.50	2.617	2.967	2.612	2.708	2.756	2.465
40.75	2.617	2.978	2.604	2.708	2.756	2.465
41.00	2.600	2.978	2.604	2.706	2.729	2.449
41.25	2.600	2.978	2.598	2.706	2.748	2.449
41.50	2.626	2.978	2.598	2.711	2.748	2.475
41.75	2.626	2.959	2.594	2.711	2.704	2.456
42.00	2.630	2.939	2.594	2.707	2.704	2.456
42.25	2.609	2.939	2.547	2.708	2.735	2.429
42.50	2.609	2.974	2.524	2.708	2.702	2.429
42.75	2.628	2.974	2.524	2.696	2.702	2.430
43.00	2.628	2.929	2.538	2.696	2.708	2.418
43.25	2.588	2.926	2.538	2.694	2.708	2.418
43.50	2.592	2.926	2.572	2.705	2.674	2.414
43.75	2.592	2.920	2.537	2.705	2.674	2.414
44.00	2.583	2.920	2.537	2.658	2.660	2.395
44.25	2.583	2.914	2.517	2.658	2.650	2.395
44.50	2.552	2.914	2.517	2.647	2.650	2.409
44.75	2.552	2.889	2.540	2.647	2.653	2.370
45.00	2.566	2.894	2.540	2.656	2.653	2.370
45.25	2.537	2.894	2.525	2.655	2.639	2.386
45.50	2.537	2.867	2.513	2.655	2.611	2.386
45.75	2.530	2.867	2.513	2.636	2.611	2.354
46.00	2.530	2.865	2.499	2.636	2.613	2.354
46.25	2.570	2.848	2.499	2.627	2.613	2.378
46.50	2.487	2.848	2.484	2.629	2.640	2.350
46.75	2.487	2.860	2.468	2.629	2.640	2.350
47.00	2.519	2.860	2.468	2.627	2.603	2.347
47.25	2.519	2.842	2.467	2.627	2.576	2.347
47.50	2.533	2.842	2.467	2.607	2.576	2.335
47.75	2.533	2.858	2.475	2.607	2.580	2.330
48.00	2.501	2.818	2.475	2.567	2.580	2.330
48.25	2.469	2.818	2.458	2.587	2.566	2.335
48.50	2.469	2.808	2.461	2.587	2.542	2.335
48.75	2.496	2.808	2.461	2.564	2.542	2.288
49.00	2.496	2.808	2.418	2.564	2.559	2.288
49.25	2.445	2.772	2.418	2.538	2.559	2.301
49.50	2.445	2.772	2.428	2.520	2.537	2.287
49.75	2.444	2.801	2.417	2.520	2.537	2.287
50.00	2.450	2.801	2.417	2.497	2.535	2.278
50.25	2.450	2.756	2.403	2.497	2.527	2.278
50.50	2.455	2.756	2.403	2.502	2.527	2.270
50.75	2.455	2.759	2.409	2.502	2.529	2.270

51.00	2.430	2.739	2.409	2.518	2.529	2.270
51.25	2.425	2.739	2.375	2.485	2.498	2.233
51.50	2.425	2.749	2.377	2.485	2.498	2.233
51.75	2.403	2.749	2.377	2.489	2.481	2.261
52.00	2.403	2.723	2.361	2.489	2.494	2.261
52.25	2.447	2.723	2.361	2.498	2.494	2.236
52.50	2.447	2.715	2.383	2.498	2.466	2.235
52.75	2.415	2.708	2.383	2.452	2.466	2.235
53.00	2.386	2.708	2.383	2.452	2.433	2.224
53.25	2.386	2.628	2.348	2.452	2.448	2.224
53.50	2.384	2.628	2.348	2.437	2.448	2.218
53.75	2.384	2.654	2.315	2.437	2.428	2.225
54.00	2.377	2.647	2.315	2.421	2.428	2.225
54.25	2.349	2.647	2.291	2.398	2.412	2.184
54.50	2.349	2.612	2.314	2.398	2.412	2.184
54.75	2.321	2.612	2.314	2.390	2.410	2.164
55.00	2.321	2.595	2.285	2.390	2.417	2.164
55.25	2.307	2.595	2.285	2.415	2.417	2.157
55.50	2.307	2.582	2.292	2.415	2.381	2.131
55.75	2.311	2.623	2.292	2.402	2.381	2.131
56.00	2.322	2.623	2.302	2.364	2.374	2.154
56.25	2.322	2.592	2.253	2.364	2.349	2.154
56.50	2.296	2.592	2.253	2.396	2.349	2.130
56.75	2.296	2.601	2.241	2.396	2.367	2.130
57.00	2.251	2.568	2.241	2.344	2.367	2.131
57.25	2.274	2.568	2.253	2.339	2.349	2.116
57.50	2.274	2.524	2.242	2.339	2.349	2.116
57.75	2.272	2.524	2.242	2.359	2.348	2.110
58.00	2.272	2.533	2.235	2.359	2.333	2.110
58.25	2.255	2.533	2.235	2.323	2.333	2.119
58.50	2.255	2.523	2.244	2.323	2.328	2.108
58.75	2.203	2.516	2.244	2.344	2.328	2.108
59.00	2.213	2.516	2.202	2.302	2.315	2.074
59.25	2.213	2.476	2.212	2.302	2.284	2.074
59.50	2.193	2.476	2.212	2.312	2.284	2.084
59.75	2.193	2.478	2.167	2.312	2.261	2.084
60.00	2.220	2.474	2.167	2.290	2.261	2.060
60.25	2.220	2.474	2.202	2.298	2.245	2.048
60.50	2.217	2.475	2.196	2.298	2.245	2.048
60.75	2.211	2.475	2.196	2.281	2.267	2.044
61.00	2.211	2.437	2.153	2.281	2.230	2.044
61.25	2.184	2.437	2.153	2.263	2.230	2.026
61.50	2.184	2.447	2.132	2.263	2.197	2.002
61.75	2.170	2.418	2.132	2.249	2.197	2.002
62.00	2.124	2.418	2.126	2.245	2.209	2.012
62.25	2.124	2.384	2.126	2.245	2.209	2.012
62.50	2.119	2.384	2.126	2.215	2.201	1.998
62.75	2.119	2.353	2.091	2.215	2.192	1.998
63.00	2.131	2.353	2.091	2.220	2.192	1.993
63.25	2.131	2.408	2.115	2.220	2.207	1.981
63.50	2.134	2.377	2.115	2.185	2.207	1.981
63.75	2.118	2.377	2.093	2.160	2.159	1.984

64.00	2.118	2.378	2.054	2.160	2.170	1.984
64.25	2.082	2.378	2.054	2.190	2.170	1.961
64.50	2.082	2.345	2.064	2.190	2.130	1.965
64.75	2.064	2.321	2.064	2.157	2.130	1.965
65.00	2.082	2.321	2.053	2.145	2.142	1.947
65.25	2.082	2.328	2.070	2.145	2.142	1.947
65.50	2.062	2.328	2.070	2.129	2.122	1.911
65.75	2.062	2.345	2.047	2.129	2.089	1.911
66.00	2.061	2.345	2.047	2.106	2.089	1.927
66.25	2.061	2.299	2.017	2.106	2.086	1.924
66.50	2.044	2.295	2.017	2.135	2.086	1.924
66.75	2.033	2.295	1.992	2.117	2.075	1.906
67.00	2.033	2.304	2.007	2.117	2.072	1.906
67.25	2.029	2.304	2.007	2.086	2.072	1.874
67.50	2.029	2.271	1.994	2.086	2.047	1.874
67.75	1.961	2.279	1.994	2.094	2.047	1.896
68.00	1.990	2.279	1.964	2.072	2.042	1.857
68.25	1.990	2.271	1.960	2.072	2.042	1.857
68.50	1.989	2.271	1.960	2.064	2.018	1.873
68.75	1.989	2.260	1.958	2.064	2.027	1.873
69.00	1.970	2.260	1.958	2.035	2.027	1.855
69.25	1.970	2.232	1.976	2.035	2.041	1.851
69.50	1.937	2.247	1.976	2.047	2.041	1.851
69.75	1.975	2.247	1.907	2.046	1.950	1.830
70.00	1.975	2.229	1.911	2.046	1.987	1.830
70.25	1.939	2.229	1.911	2.030	1.987	1.812
70.50	1.939	2.181	1.916	2.030	1.961	1.812
70.75	1.961	2.211	1.916	1.997	1.961	1.806
71.00	1.961	2.211	1.886	1.988	1.980	1.812
71.25	1.953	2.156	1.892	1.988	1.980	1.812
71.50	1.941	2.156	1.892	1.989	1.946	1.773
71.75	1.941	2.183	1.862	1.989	1.948	1.773
72.00	1.903	2.183	1.862	1.992	1.948	1.781
72.25	1.903	2.170	1.852	1.992	1.959	1.747
72.50	1.879	2.148	1.852	1.984	1.959	1.747
72.75	1.942	2.148	1.878	1.931	1.916	1.769
73.00	1.942	2.125	1.857	1.931	1.916	1.769
73.25	1.875	2.125	1.857	1.923	1.933	1.751
73.50	1.875	2.106	1.858	1.923	1.902	1.751
73.75	1.875	2.106	1.858	1.925	1.902	1.758
74.00	1.875	2.105	1.843	1.925	1.884	1.724
74.25	1.848	2.090	1.843	1.914	1.884	1.724
74.50	1.862	2.090	1.842	1.895	1.875	1.711
74.75	1.862	2.096	1.805	1.895	1.851	1.711
75.00	1.847	2.096	1.805	1.898	1.851	1.711
75.25	1.847	2.087	1.814	1.898	1.866	1.688
75.50	1.830	2.109	1.814	1.864	1.866	1.688
75.75	1.825	2.109	1.789	1.900	1.853	1.688
76.00	1.825	2.058	1.796	1.900	1.853	1.688
76.25	1.803	2.058	1.796	1.859	1.837	1.705
76.50	1.803	2.047	1.781	1.859	1.836	1.705
76.75	1.802	2.047	1.781	1.836	1.836	1.677

77.00	1.802	2.055	1.759	1.836	1.833	1.640
77.25	1.789	2.032	1.759	1.859	1.833	1.640
77.50	1.752	2.032	1.765	1.814	1.818	1.647
77.75	1.752	2.040	1.777	1.814	1.791	1.647
78.00	1.754	2.040	1.777	1.838	1.791	1.646
78.25	1.754	2.027	1.765	1.838	1.785	1.646
78.50	1.742	1.993	1.765	1.807	1.785	1.630
78.75	1.751	1.993	1.781	1.833	1.795	1.621
79.00	1.751	1.965	1.745	1.833	1.795	1.621
79.25	1.732	1.965	1.745	1.796	1.764	1.616
79.50	1.732	1.958	1.730	1.796	1.751	1.616
79.75	1.699	1.958	1.730	1.819	1.751	1.625
80.00	1.699	1.967	1.713	1.819	1.760	1.610
80.25	1.702	1.940	1.713	1.795	1.760	1.610
80.50	1.698	1.940	1.712	1.788	1.760	1.589
80.75	1.698	1.959	1.697	1.788	1.737	1.589
81.00	1.724	1.959	1.697	1.773	1.737	1.582
81.25	1.724	1.917	1.673	1.773	1.710	1.582
81.50	1.719	1.928	1.673	1.759	1.710	1.597
81.75	1.719	1.928	1.682	1.716	1.707	1.548
82.00	1.665	1.946	1.660	1.716	1.707	1.548
82.25	1.686	1.946	1.660	1.724	1.699	1.542
82.50	1.686	1.906	1.669	1.724	1.712	1.542
82.75	1.671	1.906	1.669	1.720	1.712	1.547
83.00	1.671	1.884	1.631	1.720	1.701	1.541
83.25	1.682	1.895	1.631	1.729	1.701	1.541
83.50	1.685	1.895	1.659	1.729	1.681	1.546
83.75	1.685	1.886	1.636	1.729	1.681	1.546
84.00	1.634	1.886	1.636	1.717	1.663	1.499
84.25	1.634	1.872	1.626	1.717	1.676	1.499
84.50	1.674	1.872	1.626	1.692	1.676	1.517
84.75	1.674	1.850	1.626	1.692	1.639	1.494
85.00	1.662	1.834	1.626	1.672	1.639	1.494
85.25	1.634	1.834	1.596	1.684	1.615	1.507
85.50	1.634	1.849	1.611	1.684	1.608	1.507
85.75	1.627	1.849	1.611	1.652	1.608	1.503
86.00	1.627	1.841	1.594	1.652	1.632	1.494
86.25	1.609	1.825	1.594	1.636	1.632	1.494
86.50	1.595	1.825	1.597	1.615	1.613	1.463
86.75	1.595	1.840	1.587	1.615	1.613	1.463
87.00	1.581	1.840	1.587	1.620	1.609	1.451
87.25	1.581	1.810	1.576	1.620	1.567	1.451
87.50	1.599	1.810	1.576	1.627	1.567	1.455
87.75	1.599	1.774	1.581	1.627	1.585	1.437
88.00	1.577	1.786	1.581	1.589	1.585	1.437
88.25	1.561	1.786	1.565	1.591	1.582	1.434
88.50	1.561	1.791	1.551	1.591	1.562	1.434
88.75	1.565	1.791	1.551	1.578	1.562	1.424
89.00	1.565	1.758	1.552	1.578	1.544	1.424
89.25	1.528	1.740	1.552	1.581	1.544	1.395
89.50	1.548	1.740	1.531	1.581	1.538	1.452
89.75	1.548	1.742	1.523	1.581	1.538	1.452

90.00	1.545	1.742	1.523	1.557	1.537	1.386
90.25	1.545	1.735	1.516	1.557	1.512	1.386
90.50	1.545	1.735	1.516	1.573	1.512	1.387
90.75	1.545	1.734	1.490	1.573	1.503	1.393
91.00	1.564	1.692	1.490	1.544	1.503	1.393
91.25	1.509	1.692	1.492	1.518	1.516	1.375
91.50	1.509	1.704	1.506	1.518	1.455	1.375
91.75	1.508	1.704	1.506	1.528	1.455	1.386
92.00	1.508	1.700	1.473	1.528	1.473	1.386
92.25	1.497	1.685	1.473	1.519	1.473	1.342
92.50	1.497	1.685	1.454	1.533	1.510	1.337
92.75	1.501	1.678	1.449	1.533	1.510	1.337
93.00	1.476	1.678	1.449	1.484	1.465	1.342
93.25	1.476	1.687	1.474	1.484	1.478	1.342
93.50	1.467	1.687	1.474	1.476	1.478	1.335
93.75	1.467	1.666	1.449	1.476	1.420	1.339
94.00	1.499	1.668	1.449	1.466	1.420	1.339
94.25	1.460	1.668	1.437	1.486	1.445	1.304
94.50	1.460	1.670	1.398	1.486	1.445	1.304
94.75	1.471	1.670	1.398	1.495	1.442	1.293
95.00	1.471	1.639	1.404	1.495	1.429	1.293
95.25	1.460	1.639	1.404	1.469	1.429	1.300
95.50	1.460	1.676	1.415	1.469	1.419	1.293
95.75	1.449	1.601	1.415	1.433	1.419	1.293
96.00	1.431	1.601	1.414	1.436	1.416	1.261
96.25	1.431	1.630	1.413	1.436	1.375	1.261
96.50	1.439	1.630	1.413	1.430	1.375	1.250
96.75	1.439	1.603	1.375	1.430	1.370	1.280
97.00	1.445	1.590	1.375	1.425	1.370	1.280
97.25	1.417	1.590	1.372	1.410	1.370	1.271
97.50	1.417	1.612	1.372	1.410	1.370	1.271
97.75	1.422	1.612	1.372	1.425	1.380	1.261
98.00	1.422	1.605	1.407	1.425	1.367	1.261
98.25	1.421	1.605	1.407	1.379	1.367	1.235
98.50	1.421	1.559	1.373	1.379	1.352	1.253
98.75	1.389	1.559	1.373	1.394	1.352	1.253
99.00	1.380	1.559	1.387	1.401	1.359	1.228
99.25	1.380	1.551	1.358	1.401	1.359	1.228
99.50	1.418	1.551	1.358	1.365	1.359	1.250
99.75	1.418	1.535	1.351	1.365	1.326	1.250
100.00	1.391	1.544	1.351	1.338	1.326	1.233
100.25	1.375	1.544	1.364	1.357	1.301	1.213
100.50	1.375	1.527	1.337	1.357	1.301	1.213
100.75	1.373	1.527	1.337	1.366	1.299	1.207
101.00	1.373	1.522	1.323	1.366	1.310	1.207
101.25	1.373	1.522	1.323	1.335	1.310	1.189
101.50	1.373	1.493	1.317	1.335	1.311	1.175
101.75	1.350	1.522	1.317	1.318	1.311	1.175
102.00	1.340	1.522	1.328	1.339	1.298	1.204
102.25	1.340	1.511	1.314	1.339	1.300	1.204
102.50	1.364	1.511	1.314	1.300	1.300	1.165
102.75	1.364	1.496	1.294	1.300	1.288	1.165

103.00	1.336	1.472	1.294	1.296	1.288	1.145
103.25	1.336	1.472	1.287	1.273	1.242	1.153
103.50	1.328	1.509	1.277	1.273	1.242	1.153
103.75	1.350	1.509	1.277	1.280	1.254	1.145
104.00	1.350	1.471	1.255	1.280	1.268	1.145
104.25	1.298	1.471	1.255	1.264	1.268	1.165
104.50	1.298	1.454	1.271	1.264	1.254	1.180
104.75	1.318	1.464	1.271	1.251	1.254	1.180
105.00	1.312	1.464	1.243	1.243	1.244	1.125
105.25	1.312	1.472	1.230	1.243	1.244	1.125
105.50	1.302	1.472	1.230	1.249	1.224	1.133
105.75	1.302	1.486	1.219	1.249	1.205	1.133
106.00	1.310	1.486	1.219	1.243	1.205	1.134
106.25	1.310	1.447	1.213	1.243	1.212	1.124
106.50	1.274	1.443	1.213	1.235	1.212	1.124
106.75	1.277	1.443	1.203	1.249	1.209	1.107
107.00	1.277	1.434	1.220	1.249	1.178	1.107
107.25	1.259	1.434	1.220	1.228	1.178	1.104
107.50	1.259	1.431	1.196	1.228	1.212	1.099
107.75	1.250	1.427	1.196	1.228	1.212	1.099
108.00	1.223	1.427	1.192	1.196	1.187	1.085
108.25	1.223	1.426	1.213	1.196	1.187	1.085
108.50	1.231	1.426	1.213	1.213	1.151	1.100
108.75	1.231	1.396	1.184	1.213	1.182	1.100
109.00	1.237	1.396	1.184	1.195	1.182	1.072
109.25	1.237	1.388	1.166	1.195	1.174	1.091
109.50	1.235	1.374	1.166	1.199	1.174	1.091
109.75	1.214	1.374	1.151	1.187	1.158	1.086
110.00	1.214	1.394	1.158	1.187	1.170	1.086
110.25	1.244	1.394	1.158	1.152	1.170	1.054
110.50	1.244	1.387	1.153	1.152	1.152	1.054
110.75	1.232	1.385	1.153	1.166	1.152	1.057
111.00	1.227	1.385	1.145	1.164	1.151	1.056
111.25	1.227	1.376	1.131	1.164	1.151	1.056
111.50	1.206	1.376	1.131	1.138	1.119	1.047
111.75	1.206	1.360	1.137	1.138	1.095	1.047
112.00	1.192	1.360	1.137	1.163	1.095	1.022
112.25	1.192	1.349	1.141	1.163	1.103	1.005
112.50	1.202	1.340	1.141	1.130	1.103	1.005
112.75	1.166	1.340	1.077	1.131	1.117	1.023
113.00	1.166	1.328	1.094	1.131	1.107	1.023
113.25	1.163	1.328	1.094	1.136	1.107	1.044
113.50	1.163	1.314	1.105	1.136	1.093	1.044
113.75	1.166	1.311	1.105	1.139	1.093	1.030
114.00	1.166	1.311	1.079	1.116	1.098	1.024
114.25	1.181	1.327	1.083	1.116	1.098	1.024
114.50	1.162	1.327	1.083	1.114	1.049	1.022
114.75	1.162	1.307	1.103	1.114	1.081	1.022
115.00	1.152	1.307	1.103	1.124	1.081	0.998
115.25	1.152	1.346	1.086	1.124	1.079	1.009
115.50	1.137	1.307	1.086	1.094	1.079	1.009
115.75	1.182	1.307	1.086	1.098	1.036	0.978

116.00	1.182	1.280	1.082	1.098	1.036	0.978
116.25	1.136	1.280	1.082	1.079	1.077	1.015
116.50	1.136	1.286	1.069	1.079	1.050	1.015
