# Supplementary material for: Impact of drill bit wear on temperature increase in dental implant osteotomy: an in vitro study
Source: PLoS One. 2025 Mar 19;20(3):e0319492. doi: 10.1371/journal.pone.0319492 (PMC11922234; doi:10.1371/journal.pone.0319492)
Supplement: S8 Table — In this table, the average torque values during insertion for the AT protocol are presented (PDF) [file pone.0319492.s008.pdf]

Protocol	AT					
Hole numb	1	10	20	30	40	50
Time (s)	Torque_avg (Nmm)	Torque_avg (Nmm)	Torque_avg (Nmm)	Torque_avg (Nmm)	Torque_avg (Nmm)	Torque_avg (Nmm)
0.00	14.00	14.00	14.00	15.00	16.00	18.00
0.10	14.00	13.00	16.00	16.00	20.00	15.00
0.20	15.00	14.00	16.00	15.00	22.00	13.00
0.30	17.00	15.00	17.00	16.00	23.00	13.00
0.40	19.00	18.00	19.00	19.00	22.00	12.00
0.50	20.00	18.00	21.00	17.00	25.00	14.00
0.60	21.00	18.00	20.00	16.00	25.00	14.00
0.70	24.00	21.00	25.00	20.00	24.00	12.00
0.80	27.00	20.00	26.00	24.00	25.00	13.00
0.90	26.00	18.00	28.00	20.00	28.00	18.00
1.00	28.00	22.00	28.00	22.00	29.00	23.00
1.10	29.00	23.00	29.00	26.00	30.00	25.00
1.20	32.00	24.00	33.00	26.00	32.00	25.00
1.30	35.00	24.00	30.00	24.00	32.00	28.00
1.40	33.00	26.00	30.00	27.00	33.00	35.00
1.50	32.00	28.00	30.00	33.00	35.00	42.00
1.60	33.00	28.00	34.00	31.00	37.00	43.00
1.70	32.00	31.00	34.00	36.00	38.00	40.00
1.80	33.00	32.00	36.00	37.00	38.00	41.00
1.90	33.00	31.00	35.00	37.00	39.00	38.00
2.00	36.00	33.00	37.00	36.00	47.00	39.00
2.10	38.00	36.00	36.00	35.00	40.00	32.00
2.20	35.00	35.00	37.00	36.00	39.00	35.00
2.30	37.00	34.00	36.00	39.00	40.00	36.00
2.40	39.00	33.00	40.00	38.00	45.00	42.00
2.50	41.00	36.00	36.00	38.00	41.00	45.00
2.60	42.00	37.00	43.00	41.00	42.00	41.00
2.70	38.00	38.00	40.00	39.00	42.00	43.00
2.80	43.00	38.00	41.00	40.00	43.00	42.00
2.90	43.00	42.00	39.00	39.00	44.00	42.00
3.00	42.00	44.00	43.00	42.00	46.00	47.00
3.10	40.00	46.00	43.00	42.00	45.00	46.00
3.20	42.00	41.00	44.00	48.00	47.00	51.00
3.30	44.00	44.00	47.00	48.00	49.00	50.00
3.40	46.00	41.00	46.00	49.00	45.00	51.00
3.50	43.00	44.00	44.00	46.00	49.00	53.00
3.60	46.00	44.00	47.00	50.00	46.00	47.00
3.70	46.00	43.00	46.00	52.00	48.00	55.00
3.80	47.00	46.00	44.00	58.00	48.00	52.00
3.90	42.00	45.00	47.00	54.00	47.00	53.00
4.00	43.00	45.00	47.00	58.00	51.00	51.00
4.10	48.00	44.00	50.00	57.00	46.00	55.00
4.20	44.00	44.00	47.00	49.00	50.00	61.00
4.30	45.00	49.00	48.00	45.00	47.00	56.00
4.40	48.00	47.00	45.00	52.00	53.00	49.00
4.50	51.00	49.00	48.00	49.00	50.00	52.00
4.60	51.00	48.00	45.00	50.00	51.00	52.00
4.70	49.00	46.00	49.00	48.00	55.00	53.00

4.80	46.00	48.00	52.00	52.00	48.00	50.00
4.90	52.00	47.00	53.00	52.00	47.00	53.00
5.00	49.00	51.00	48.00	47.00	52.00	59.00
5.10	51.00	53.00	51.00	51.00	51.00	52.00
5.20	49.00	51.00	53.00	49.00	52.00	56.00
5.30	52.00	54.00	51.00	50.00	51.00	56.00
5.40	50.00	52.00	50.00	51.00	54.00	64.00
5.50	46.00	48.00	54.00	52.00	56.00	63.00
5.60	47.00	49.00	57.00	52.00	52.00	62.00
5.70	54.00	53.00	54.00	54.00	54.00	61.00
5.80	54.00	50.00	56.00	49.00	57.00	60.00
5.90	53.00	52.00	52.00	57.00	59.00	53.00
6.00	54.00	50.00	56.00	49.00	56.00	59.00
6.10	55.00	54.00	52.00	55.00	58.00	58.00
6.20	58.00	51.00	56.00	54.00	56.00	58.00
6.30	47.00	50.00	54.00	60.00	54.00	59.00
6.40	53.00	50.00	58.00	57.00	58.00	61.00
6.50	52.00	53.00	62.00	53.00	58.00	64.00
6.60	56.00	51.00	62.00	56.00	54.00	60.00
6.70	54.00	54.00	58.00	55.00	57.00	64.00
6.80	54.00	54.00	56.00	58.00	59.00	65.00
6.90	57.00	57.00	60.00	57.00	63.00	66.00
7.00	58.00	52.00	63.00	58.00	58.00	67.00
7.10	52.00	53.00	59.00	59.00	60.00	64.00
7.20	55.00	58.00	57.00	61.00	56.00	57.00
7.30	54.00	54.00	61.00	55.00	56.00	63.00
7.40	55.00	55.00	57.00	59.00	56.00	54.00
7.50	56.00	55.00	57.00	56.00	58.00	60.00
7.60	53.00	59.00	54.00	64.00	60.00	58.00
7.70	58.00	56.00	63.00	62.00	61.00	60.00
7.80	53.00	55.00	60.00	62.00	60.00	62.00
7.90	56.00	55.00	62.00	61.00	63.00	60.00
8.00	53.00	55.00	64.00	59.00	61.00	67.00
8.10	56.00	52.00	66.00	59.00	58.00	64.00
8.20	59.00	56.00	64.00	56.00	62.00	61.00
8.30	60.00	55.00	63.00	57.00	65.00	61.00
8.40	59.00	57.00	61.00	59.00	67.00	61.00
8.50	61.00	57.00	60.00	60.00	61.00	61.00
8.60	58.00	57.00	58.00	58.00	63.00	61.00
8.70	59.00	60.00	60.00	65.00	61.00	61.00
8.80	58.00	56.00	63.00	64.00	59.00	67.00
8.90	58.00	58.00	62.00	65.00	58.00	59.00
9.00	59.00	63.00	68.00	61.00	64.00	64.00
9.10	57.00	64.00	66.00	64.00	63.00	62.00
9.20	60.00	61.00	63.00	61.00	63.00	68.00
9.30	61.00	63.00	63.00	63.00	65.00	70.00
9.40	57.00	55.00	67.00	62.00	66.00	70.00
9.50	63.00	59.00	70.00	65.00	66.00	63.00
9.60	59.00	54.00	70.00	64.00	63.00	68.00
9.70	61.00	62.00	66.00	61.00	60.00	58.00
9.80	63.00	60.00	68.00	61.00	65.00	58.00
9.90	59.00	59.00	67.00	62.00	64.00	57.00

10.00	65.00	62.00	62.00	62.00	63.00	63.00
10.10	59.00	66.00	67.00	63.00	71.00	69.00
10.20	64.00	68.00	66.00	67.00	66.00	56.00
10.30	63.00	63.00	73.00	66.00	70.00	62.00
10.40	61.00	65.00	66.00	66.00	63.00	59.00
10.50	63.00	68.00	75.00	67.00	69.00	58.00
10.60	65.00	67.00	74.00	62.00	65.00	62.00
10.70	64.00	62.00	71.00	65.00	70.00	61.00
10.80	68.00	68.00	67.00	66.00	71.00	61.00
10.90	60.00	60.00	72.00	67.00	73.00	60.00
11.00	67.00	61.00	72.00	68.00	72.00	60.00
11.10	62.00	61.00	72.00	64.00	74.00	66.00
11.20	65.00	68.00	68.00	69.00	66.00	60.00
11.30	66.00	65.00	74.00	68.00	71.00	60.00
11.40	68.00	68.00	76.00	69.00	72.00	61.00
11.50	70.00	74.00	73.00	69.00	72.00	64.00
11.60	73.00	74.00	77.00	69.00	74.00	64.00
11.70	72.00	69.00	72.00	74.00	70.00	67.00
11.80	71.00	68.00	77.00	74.00	77.00	69.00
11.90	79.00	70.00	78.00	72.00	74.00	71.00
12.00	76.00	74.00	82.00	71.00	74.00	60.00
12.10	79.00	72.00	78.00	70.00	76.00	67.00
12.20	77.00	74.00	77.00	74.00	79.00	67.00
12.30	88.00	80.00	80.00	78.00	80.00	68.00
12.40	68.00	72.00	80.00	82.00	85.00	67.00
