# Supplementary material for: Impact of drill bit wear on temperature increase in dental implant osteotomy: an in vitro study
Source: PLoS One. 2025 Mar 19;20(3):e0319492. doi: 10.1371/journal.pone.0319492 (PMC11922234; doi:10.1371/journal.pone.0319492)
Supplement: S9 Table — In this table, the average torque values during insertion for the PT protocol are presented. (PDF) [file pone.0319492.s009.pdf]

Protocol	PT					
Hole number	1	10	20	30	40	50
Time (s)	Torque_avg (Nmm)	Torque_avg (Nmm)	Torque_avg (Nmm)	Torque_avg (Nmm)	Torque_avg (Nmm)	Torque_avg (Nmm)
0.00	14.00	14.00	14.00	14.00	14.00	14.00
0.10	14.00	13.00	14.00	14.00	14.00	16.00
0.20	16.00	15.00	16.00	17.00	18.00	17.00
0.30	18.00	16.00	17.00	18.00	18.00	17.00
0.40	19.00	18.00	19.00	19.00	20.00	19.00
0.50	20.00	17.00	21.00	18.00	25.00	20.00
0.60	21.00	18.00	24.00	19.00	25.00	26.00
0.70	24.00	24.00	27.00	20.00	23.00	24.00
0.80	28.00	27.00	29.00	22.00	29.00	26.00
0.90	30.00	27.00	34.00	27.00	28.00	26.00
1.00	30.00	29.00	33.00	32.00	32.00	29.00
1.10	34.00	30.00	39.00	34.00	33.00	33.00
1.20	37.00	31.00	39.00	36.00	38.00	33.00
1.30	35.00	36.00	42.00	42.00	39.00	36.00
1.40	42.00	36.00	46.00	41.00	46.00	39.00
1.50	45.00	44.00	48.00	44.00	47.00	45.00
1.60	52.00	41.00	51.00	42.00	49.00	48.00
1.70	57.00	47.00	50.00	48.00	55.00	49.00
1.80	67.00	53.00	45.00	48.00	65.00	50.00
1.90	76.00	53.00	54.00	55.00	74.00	57.00
2.00	73.00	59.00	54.00	58.00	87.00	62.00
2.10	74.00	71.00	61.00	59.00	85.00	67.00
2.20	77.00	76.00	66.00	58.00	91.00	67.00
2.30	83.00	80.00	73.00	62.00	92.00	75.00
2.40	93.00	84.00	77.00	66.00	99.00	79.00
2.50	91.00	89.00	68.00	79.00	103.00	78.00
2.60	105.00	96.00	76.00	76.00	107.00	82.00
2.70	99.00	103.00	80.00	78.00	117.00	97.00
2.80	108.00	112.00	80.00	87.00	115.00	95.00
2.90	109.00	114.00	89.00	89.00	122.00	103.00
3.00	126.00	118.00	87.00	91.00	108.00	98.00
3.10	128.00	109.00	93.00	83.00	123.00	104.00
3.20	124.00	118.00	90.00	97.00	129.00	106.00
3.30	129.00	116.00	96.00	101.00	151.00	110.00
3.40	130.00	134.00	98.00	104.00	139.00	113.00
3.50	125.00	130.00	108.00	107.00	149.00	122.00
3.60	135.00	132.00	108.00	108.00	136.00	134.00
3.70	147.00	133.00	120.00	108.00	149.00	131.00
3.80	143.00	138.00	114.00	113.00	145.00	132.00
3.90	148.00	137.00	117.00	112.00	166.00	125.00
4.00	149.00	143.00	116.00	126.00	171.00	131.00
4.10	154.00	138.00	126.00	123.00	170.00	148.00
4.20	155.00	157.00	129.00	129.00	162.00	143.00
4.30	165.00	155.00	133.00	124.00	169.00	153.00
4.40	163.00	160.00	134.00	136.00	158.00	146.00
4.50	179.00	153.00	144.00	140.00	177.00	149.00
4.60	179.00	158.00	141.00	146.00	177.00	146.00
4.70	190.00	146.00	141.00	152.00	176.00	153.00

4.80	176.00	163.00	158.00	147.00	171.00	155.00
4.90	180.00	169.00	158.00	158.00	179.00	168.00
5.00	175.00	175.00	166.00	167.00	180.00	177.00
5.10	198.00	181.00	167.00	157.00	205.00	165.00
5.20	179.00	186.00	172.00	166.00	206.00	160.00
5.30	189.00	179.00	166.00	175.00	201.00	176.00
5.40	172.00	185.00	183.00	178.00	205.00	190.00
5.50	173.00	192.00	183.00	173.00	194.00	193.00
5.60	179.00	191.00	198.00	183.00	189.00	212.00
5.70	186.00	194.00	185.00	186.00	208.00	197.00
5.80	192.00	185.00	202.00	193.00	218.00	192.00
5.90	194.00	196.00	179.00	191.00	225.00	204.00
